# Implementing performance improvement in New Zealand emergency departments: the six hour time target policy national research project protocol

**DOI:** 10.1186/1472-6963-12-45

**Published:** 2012-02-21

**Authors:** Peter Jones, Linda Chalmers, Susan Wells, Shanthi Ameratunga, Peter Carswell, Toni Ashton, Elana Curtis, Papaarangi Reid, Joanna Stewart, Alana Harper, Tim Tenbensel

**Affiliations:** 1 Adult Emergency Department, Auckland City Hospital, Park Road, Grafton, Auckland, New Zealand; 2 Department of Epidemiology and Biostatistics, School of Population Health, Faculty of Medical and Health Sciences, University of Auckland, Auckland, New Zealand; 3 Health Systems, School of Population Health, Faculty of Medical and Health Sciences, University of Auckland, Auckland, New Zealand; 4 Te Kupenga Hauora Māori, School of Population Health, Faculty of Medical and Health Sciences, University of Auckland, Auckland, New Zealand

**Keywords:** Health Policy, Emergency Medicine, Health Services Research, Quality of Health Care, Models, Theoretical, Inequalities

## Abstract

**Background:**

In May 2009, the New Zealand government announced a new policy aimed at improving the quality of Emergency Department care and whole hospital performance. Governments have increasingly looked to time targets as a mechanism for improving hospital performance and from a whole system perspective, using the Emergency Department waiting time as a performance measure has the potential to see improvements in the wider health system. However, the imposition of targets may have significant adverse consequences. There is little empirical work examining how the performance of the wider hospital system is affected by such a target. This project aims to answer the following questions: How has the introduction of the target affected broader hospital performance over time, and what accounts for these changes? Which initiatives and strategies have been successful in moving hospitals towards the target without compromising the quality of other care processes and patient outcomes? Is there a difference in outcomes between different ethnic and age groups? Which initiatives and strategies have the greatest potential to be transferred across organisational contexts?

**Methods/design:**

The study design is mixed methods; combining qualitative research into the behaviour and practices of specific case study hospitals with quantitative data on clinical outcomes and process measures of performance over the period 2006-2012. All research activity is guided by a Kaupapa Māori Research methodological approach. A dynamic systems model of acute patient flows was created to frame the study. Consequences of the target (positive and negative) will be explored by integrating analyses and insights gained from the quantitative and qualitative streams of the study.

**Discussion:**

At the time of submission of this protocol, the project has been underway for 12 months. This time was necessary to finalise both the case study sites and the secondary outcomes through key stakeholder consultation. We believe that this is an appropriate juncture to publish the protocol, now that the sites and final outcomes to be measured have been determined.

## Background

In May 2009, the New Zealand government announced a new policy aimed at improving the quality of Emergency Department (ED) care and whole hospital performance. The 'Shorter Stays in ED' target stipulates that "95% of patients will be admitted, discharged or transferred from an ED within six hours by July 2009" [1]. This target was supported by the National ED Advisory Group, made up of senior ED clinicians and managers [2]. They advised the policy initiative based on observational studies from overseas showing that overcrowding or long waits in ED were associated with poorer outcomes for patients such as increased mortality [3-5]. Similar ED time targets have been recommended by health reformers in Australia [6] and follow the lead of the English National Health Service which introduced time targets for ED in 2001 [7]. In the UK, official monitoring indicated that these targets were successfully met at the national level by 2004 [8]. This contrasted with the lack of improvement in ED waiting times in Scotland, Wales and Northern Ireland over the same time period, where such targets were not introduced [9].

Nevertheless, there has been widespread debate about whether targets in general, and the English ED targets in particular 'do more harm than good' [10]. Advocates of a target approach claim that ED targets can act as an effective catalyst for quality improvement across the whole hospital, and even the wider health system. Taking a systems view of a hospital, the ED is only one part of the patient journey. The pathway for ED patients into in-patient beds is determined by bed availability, inpatient care delivery and discharge practices. These have been shown to improve through attention to quality initiatives [11]. Therefore, from a whole system perspective, using the ED waiting time as a performance measure has the potential to see improvements in parts of the health system beyond ED. This has been demonstrated via application of dynamic system model simulations [12,13] and such simulations have become a cogent influence on policy makers.

However, instead of acting as a catalyst for stimulating and improving broader health system performance, the imposition of targets may have significant adverse consequences. Two types of adverse consequences are typically identified. The first is gaming; or reactive subversion, such as 'hitting the target but missing the point'. Examples are ambulances being made to wait outside the ED to delay arrival time, [14] or re-designating patients as short stay admissions at or around the target time to avoid 'breaching' the target [15,16]. The second is substitution effects; the potential diversion of attention from other important clinical areas and possible distortion of clinical and management priorities. Organisations, in their efforts to meet targets, turn resources and attention away from other important dimensions of performance and quality, and in doing so, shift systemic problems to other parts of the system or organisation [17]. For example, the pressure of admission from ED to an already stretched hospital service with close to 100% bed occupancy may serve to precipitate inappropriate discharge of patients. It is also possible that pressure to meet time targets for patient discharge may exacerbate inequities of care due to clinicians implicit biases [18].

As such, it is possible that the introduction of targets may ultimately detract from health service quality of care. Relevant measures of quality and performance include mortality, hospital length of stay (LOS), readmission rates, and more specific, condition-related measures such as time to reperfusion for those with acute myocardial infarction [19]. Some of these outcomes have been associated with ED overcrowding, [5,20,21] however the associations are not consistent [22-24] and there is little evidence to support the assumption that introduction of an explicit 'ED stay' performance measure will improve clinical markers of quality of care [25].

Whatever relationships there may be between the use of targets and broader clinical and hospital outcomes, these are almost certainly shaped by organisational factors. The literature on organisational studies has demonstrated that the application of performance targets drives organisational attention and resources towards achieving these targets. This has been illustrated in manufacturing, [26,27] retail, [28] and education [29]. Relevant factors include the motivational role of the Chief Executive Officer, the structure of the senior management team and elements of organisational culture. However, it is not at all clear how targets affect organisational behaviour within the complex and complicated context of a health system. Much of the organisational studies literature has been based in contexts that are relatively less complex, more likely to have an agreed organisational purpose, and where the value system of employees is more aligned [30,31].

The evidence regarding organisational responses to the English ED target does not yet add up to a coherent picture. A report commissioned by the UK Health Commission, identified a number of organisational factors contributing to delays in patient care, including the level of clinician involvement in ED and hospital management, levels of nurse absenteeism and lower proportion of non-salary expenditure [32]. This study, however, did not link ED performance with broader clinical outcomes. Another recent study sought to investigate the presence and extent of dysfunctional organisational response to the ED time target [17]. The authors undertook an aggregated analysis of the effects of the ED targets on English National Health Service trusts between 2002 and 2007. They concluded that there was no evidence of dysfunctional effects, and that if anything, the impact on quality was beneficial. However, this research has significant limitations. Firstly, only mortality and inpatient admission (itself a rather ambiguous indicator) were included in the study, and the results conflicted with other reports using the same dataset [33,34]. Secondly, it is doubtful that an exclusively quantitative study of this type would be capable of detecting gaming and effort substitution. Gaming behaviour may be undetectable or statistically insignificant at the aggregate level because the nature and strategies of gaming most likely vary across health care organisations due to differences in organisational context.

The New Zealand health care system is divided into 20 regional District Health Boards (DHB), who manage an extensive system of publicly-owned hospitals that are directly subject to government policy priorities. This provides a context in which it is possible to investigate the effect of an explicit policy directive on the performance of EDs, hospitals and wider health systems.

The introduction of the ED target in New Zealand raises some fundamental questions which can be adequately answered only by undertaking a thorough investigation of implementation processes and impact on the quality of health care delivered. There is very little empirical work that examines how the performance of the wider hospital system is affected by the introduction of a performance target within ED. Both positive and negative consequences of time targets can be best detected by combining qualitative, case-study research into the behaviour and practices of specific hospitals with quantitative data on clinical outcomes and other dimensions of performance.

A priority for this research is to contribute to health equity in New Zealand Emergency Departments. Health inequalities in New Zealand are well documented. In particular, health inequalities by ethnicity have been described as "the most consistent and compelling of health inequalities in New Zealand" [35]. Importantly, evidence also suggests that changes in the political and economic environment, including setting of health targets, may have "unequal impact" and can increase disparities rather than reduce them [36].

Key results of the 2006/7 New Zealand Health Survey [37] describe current inequalities in ED use in New Zealand by ethnicity, gender, age and neighbourhood deprivation. We will therefore consider the impact on the Target via these variables, where data is adequate.

This research therefore aims to investigate the following questions:

• *How has the introduction of the ED target affected broader hospital performance over time, and what accounts for these changes?*

• *Which initiatives and strategies have been successful in moving hospitals towards the target without compromising other quality and hospital outcomes?*

• *Is there a difference in change in outcomes between different ethnic age and deprivation groups?*

• *Which initiatives and strategies have the greatest potential to be transferred across organisational contexts?*

## Methods/design

**Figure 1:**
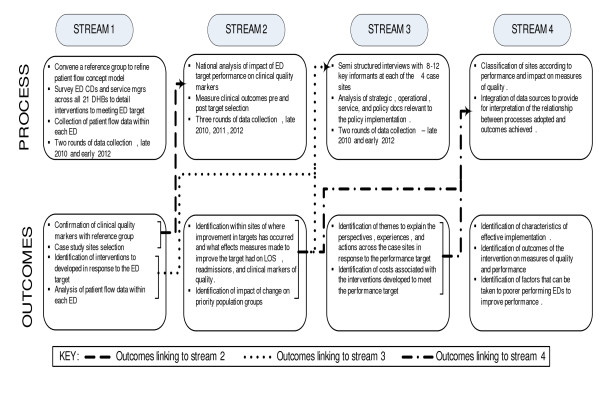
**Content and relationships of the different study streams**.

This is a mixed methods study with three interdependent research streams. The streams employ a range of quantitative and qualitative methods to address a number of research questions. Each stream is conducted consecutively and concurrently over the study time frame and outputs from each inform the others. This material is united into a final, fourth stream to address the overall aims of the study (Figure 1).

### Methodological approach with respect to health inequalities

This research project has considerable potential to contribute to the health needs of Māori. Robust examination of both clinical outcomes and the policy implementation process can reveal critical insights on how this policy will affect Māori patients receiving acute emergency care. Research findings will provide empirical support for identifying both the positive and negative effects of the policy for Māori health, and potentiate Māori health gain. Understanding whether the setting of ED targets introduces disparities for Māori (or vice versa) is a specific area of investigation for this research.

Given this context, this research is being guided by a Kaupapa Māori Research (KMR) methodology [38,39]. Within the context of this study, the KMR framework includes senior Māori research expertise throughout all stages and streams of the research process to: ensure utility and cultural safety of the research process for Māori; Māori research kaitiakitanga (guardianship and protection) of Māori data; review and approval of Māori data collection, analysis and interpretation; analysis and interpretation of Māori data and comparisons with non-Māori and review of any manuscripts involving Māori data prior to submission for publication. Such an approach requires the researchers to aim for equal explanatory and analytical power for Māori compared to non-Māori, a rigorous process to the collection, evaluation and reporting of ethnicity data from available datasets, and interpretation of the data from a non-victim blame or cultural deficit positioning [40,41]. The research team has agreed to conduct all research activity in accordance with the Tōmaiora Māori Health Research Centre research protocols [42].

### Stream one

#### Research aims

The aim of this research stream is to identify initiatives that have been implemented in response to the performance target across the 20 DHBs, to explore the impact of these initiatives on patient flow into and out of EDs, and to identify four case study sites for in-depth analysis. The selection of hospitals was based on initial target performance results from the first quarter of 2009, and by geographic location and population density.

#### Design/method and rationale

The core research question for this stream is 'What impact do different initiatives aimed at the ED performance target have on measures of patient flow? We used a dynamic system modelling approach similar to that used in other studies internationally that have attempted to model the dynamics of an ED [12,43,44] to frame this stream. This approach had four sequential steps [45].

1. An initial mapping of patient flow.

2. Refining the initial model with an expert reference group.

3. Using the refined concept model to inform a survey of clinical directors (CD) and service managers within each ED that is subject to the national performance time-target. This survey was designed to collect information on the initiatives that have been developed in response to the target and where these initiatives sit on the concept model of patient flow (Additional file 1). Respondents were be asked to identify any changes in resource use (especially staff changes) associated with the interventions and to estimate any additional departmental expenditure associated with the intervention. Information from January 2006 onwards of measures of patient flow (ED admissions/discharges, bed occupancy, LOS elective admissions and elective surgical cancellations) were collected.

4. Survey data collected from each site will subsequently be used to develop a working system dynamic model of patient flow into and out of ED, and illustrate the impact of initiatives put in place in response to the performance target.

#### Recruitment and data collection

##### Reference group

This was convened in December 2010 and consisted of the representatives of expert groups relevant to this study, including members of: the national ED advisory group to the Ministry of Health, the Australasian College for Emergency Medicine (ACEM), the College of Emergency Nurses of New Zealand (CENNZ), the Royal New Zealand College of General Practitioners (RNZCGP), Māori representatives, Pacific peoples representatives, representatives from inpatient specialties covering medicine, surgery and paediatrics, representatives of inpatient and community older people's health and the CD and service managers from each case site.

##### Survey

The CD and/or service manager in each ED completed the survey in mid 2011. Follow-up will occur at the end of 2012 to capture any further interventions.

##### Data analysis

Further analysis of the data will follow the usual conventions for system modelling [45]. The concept model will be entered into a specialised software package, iThink [46] and differential equations will be calculated to estimate the patient flow, and impact of the interventions. This will allow us to examine whether the intervention has simply moved the 'access block' to other parts of the hospital. It will also allow us to identify any cost-shifting to other services and to make some estimates of any changes in down-stream costs (such as changes in LOS). Survey results will also be analysed to determine the areas of cost related to the interventions, and the perceived impacts the interventions have had on performance.

### Stream two

#### Research aims and questions

This research stream aims to investigate the impact of the target on objective markers of quality of care. The core research questions for this stream are; 'Was there a change in clinically relevant outcomes after the target was introduced?' and 'Were there differential impacts of the target in at risk ethnic, age and deprivation groups?'

#### Design/method

We will investigate whether the rate of change of quality of care markers differs over time, pre and post introduction of the ED time targets in July 2009. The study will compare outcomes of interest using data collected from three years prior to and after the introduction of the target (1st January 2006 to 31st December 2008 and 1st January 2010 to 31st December 2012). A one year 'target settling' period six months either side of the target introduction in July 2009 will be excluded from analysis. The primary outcomes will be:

• ED and Hospital length of stay (LOS) [47]

• Re-attendance rates within 48 hours of discharge [48,49]

• Differences in these outcomes in different ethnic, age and deprivation groups

The secondary outcomes are other clinical process measures and clinical outcomes that reflect the quality of care delivery both in ED and wider hospital. The candidate indicators were determined by a systematic literature review and the final set was confirmed by an expert reference group (see Stream one, above).

The secondary outcomes will be;

• all cause mortality [2,3,50,51]

• time to reperfusion for ST elevation myocardial infarction (STEMI) [49]

• time to analgesia in ED [52]

• time to theatre for fractured neck of femur [53]

• time to antibiotics for severe infections [54]

• time to treatment in acute asthma [55,56]

• proportion of patients who leave without being seen [48,49]

• time to CT for traumatic brain injury [57,58]

• time to appendectomy for acute appendicitis [59,60]

• appropriateness of discharge information provided to General Practitioners from ED [61,62]

#### Outcome measures: definition, rationale and data collection

##### Primary

LOS starts from date/time of arrival and ends at time/date of discharge. Lack of capacity for acute admissions is a key factor driving the length of time spent in ED [63-65]. Increasing capacity by reducing LOS may have drawbacks. NZ has a shorter average LOS than both Australia and the UK [66] and LOS for Māori is two days shorter than for non-Māori [67,68], with personal experiences in hospital a contributing factor to this [67]. A small change in LOS, for example a decrease of 0.25 days, is important, as MOH data suggests this would result in approximately 125000 more bed-days available per year nationwide. The distribution of times that patients spend in ED should be a smooth curve with a right skew when plotted graphically (most patients leave within a short time, some stay much longer). We will examine the distribution of ED LOS times pre and post target introduction, looking for a 'spike' of admissions or discharges at or near the target time (which may reflect gaming or patients moved elsewhere prior to completion of their care) [69,70].

Re-attendance is defined as re-admission to hospital or representation to ED within 48 hours. This outcome is considered an adverse event [71,72] and may result from inappropriate early discharge. Data on LOS and re-attendance, along with age, ethnicity, gender and NZ deprivation score is available from routinely collected hospital data and will be collected for all 20 DHBs. Ethnicity will be sourced from the National Health Index (a unique patient identifier in NZ) and reported according to the Ethnicity Data Protocols for the Health and Disability Sector, with appropriate adjustments made to account for known discrepancies in NHI data [73].

##### Secondary

While hospital mortality will be available from national datasets for all patients within the time periods, the other secondary outcomes occur within a subgroup of patients. Using the four case study sites selected in Stream One, a series of chart audits from patient hospital records, each with specific sampling frame and sample size calculations will be conducted. These records will be chosen at random throughout the time periods of interest to gain an in-depth understanding of within hospital variation before and after the introduction of the time target policy. The records will be identified by ICD-10 codes appropriate to each clinical outcome.

##### Statistical analyses

The data for each of the outcomes will be recorded by hospital by twelve month periods (to avoid modeling seasonal change). For continuous outcomes, in order to compare the rate of change in the measures pre to post intervention and whether any change is influenced by age, ethnicity or hospital, a general linear mixed model will be used with outcome transformed as necessary to overcome correlation of mean and variance in variables such as LOS. A random coefficients model will be used to allow the change to be modeled within hospital. Explanatory variables will be year within pre- and post-intervention time periods, hospital, age category, ethnicity and pre or post intervention. Appropriate interactions of year, intervention, ethnicity, age and hospital will be investigated with higher order interactions being removed if not important and the analysis being split where non-ignorable interactions exist. Binary outcomes such as readmission will be analysed similarly using a generalised linear mixed model. Estimates of least square mean values of outcomes, with 95% confidence intervals, will be calculated, within important subgroups. Data will be analysed using STATA version 10, StataCorp LP, 4905 Lakeway Drive, College Station, TX 77845 USA.

##### Sample size calculations

All sample size estimations below are based on having 80% power to demonstrate a difference at the five percent level of significance using the models described above. Expected distributions used for estimating variance were based on one tertiary hospital's data, which was the only data available to the researchers at that stage of the study. Expected proportions in each ethnic and age category were based on New Zealand hospital admission data. Māori proportions ranged from five to 18% in the different age groups. For outcomes retrieved from routine electronic data there are very large numbers (almost one million ED attendances annually) and it is possible to look for disparities in change in Māori and other at risk ethnic and age groups and differences in change within hospitals.

The sample size calculations for the primary outcomes are based on detecting interactions. The real difference in LOS considered important to be powered to detect is 0.25 days. To detect a difference in change for Māori compared to European/other, within age groups (< 65 and ≥ 65) requires a total sample of 12,000 in those aged < 65, 76,000 in ≥ 65 (or 4,000 in ≥ 65 if there was no ethnic interaction). These numbers would be present within individual hospitals except for investigating ethnic differences in change in the older age group, where Māori numbers are less. This will be investigated at the national level.

The re-attendance rate at the index hospital was approximately five percent, somewhat higher than reported elsewhere [30,63,65,66]. To detect a real difference in change of one percent in re-attendance in Māori, or other at risk ethnic groups, compared to European/other, within age groups would require approximately 10,000 in the smallest group. Nationwide, there are about 9,000 Māori people older than 65 years admitted annually. Therefore, with three years data after the target was introduced we will have greater than 80% power to investigate this difference.

For secondary outcomes that require manual data extraction, random samples of records of the appropriate size from the six years of the study will be drawn. A sample size calculation for time to reperfusion is given here as this represents the smallest number of clinical events, is clinically very important and health outcomes are associated with disparities by age and ethnicity [74]. Across NZ approximately 1,500 patients per year with STEMI receive reperfusion therapy [74]. In order to detect a five minute change in time to reperfusion, based on a mean (SD) of the log transformed time of 3.61(.58) would require 160 patients per year over the time period of the study or 50 per year to detect a 10 minute change. For each confirmed clinical outcome, a power calculation to determine an appropriate sample size for our research question will be undertaken.

### Stream three

#### Research aim and question

The specific aim of this research stream is to explore how organisations respond to the target by investigating the perspectives, experiences and actions of front line clinical and management personnel in the ED and wider hospital. A second and important aim is to identify the variations in organisational responses between case study sites and different informants. The core research question for this steam is 'How is the ED time target policy implemented?'

#### Design/method and rationale

This stream involves qualitative research into the four case study hospitals. Key strengths of qualitative research in the field of health service and policy research include the ability to enhance understanding of complex phenomenon in dynamic organizational contexts, and interpretation of experience from a variety of actors [75]. Qualitative design and methods have been recently applied to investigate the effect of performance improvement initiatives in the UK from the perspective of front line personnel, for example the emergency nursing experience of the 4 hour ED time target [76], the experience of pay for performance in primary health care providers [77,78] and factors affecting the adoption of a "see and treat" model in emergency care [79]. The design for this research stream is qualitative multiple case study, using semi-structured interviews (Additional file 2) and policy documents within the case study sites to collect data. Comparison and contrast of findings can be made within and across different cases and different informant groups (clinical and management, inside the ED and in the wider hospital).

#### Recruitment and data collection

The first of two rounds of semi-structured interviews across the four case study hospitals, was conducted in early 2011, the second will be in mid 2012. These interviews explore the organisational response to the ED target and associated interventions. Two rounds of data collection will ensure that any changes over time can be captured. The choice of four case studies is based on achieving richness of data, variance in context and comparison of performance, but also recognises resource and time constraints within the research project overall. Between ten and twelve research informants at each site were purposively selected to achieve theoretical saturation, ensure spread across informant groups and requisite experience of policy implementation. Semi-structured interviews ensure focus and structure in the interview, whilst enabling flexibility to probe informant responses for detail, clarification of meaning or examples. Interviews were conducted face to face and audio taped. Strategic, operational, service and policy documents relevant to policy implementation, such as memoranda, guidelines and procedures, will also be collected from case study sites.

#### Data analysis

Transcribed interview data will be analysed using a general thematic inductive approach to identify the range of perspectives, experiences and actions across the hospitals and the respective groups of policy implementers. Documentary data will also be analysed using thematic inductive techniques. Interview data analysis will be analysed using MAXQDA (version 10), Verbi GmbH, Berlin, Germany.

### Stream four

As indicated in Figure 1 above, this stream will draw together information from the other streams. In essence, Stream Two will provide a picture of the patterns of clinical and organisational performance over time (2006-2012), while Streams One and Three will provide the means to interpret and explain such patterns and provide information on costs and resources used to meet the target.

#### Data analysis

Using data from Stream Two we will classify the case study organisations according to degree of success in meeting ED target with/without adverse clinical and hospital outcomes. Themes developed in the analysis of Stream One and Stream Three data will be mapped against Stream Two results to construct explanations of success/failure. For example, information collected in Stream Two may raise a suspicion of gaming in a particular site. A 'spike' of admissions or discharges at or near the target time may reflect gaming; or that patients are moved out of ED prior to completion of their care. Such consequences may show up in data on clinical outcomes. The data collected in Stream Three will provide a more detailed interpretation of this phenomenon and its broader consequences, especially when such information is incorporated into Stream Three interview schedules.

There are some important precedents for using a mixed methods approach in order to account for ED and hospital responses to targets. Mason et al. were able to attribute some variation in ED mean waiting times to the management style of lead clinicians, with data on management style initially collected through in-depth interviews [32]. Mannion et al., using a multiple case-study approach, identified key attributes of organisational culture and management style that accounted for differences between 'high performing' from 'low performing' trusts according to official performance measures [80].

The multi-case study approach should allow the research to distinguish common organisational factors affecting quality and performance from factors that are highly context-specific. As such, this research can help to distinguish between general strategies for quality and performance improvement (strategies that appear to have been successful in more than one setting), and future initiatives that will need to be highly tailored to specific organisational contexts.

## Discussion

### Current stage of research

At the time of submission of this protocol, the project has been underway for 12 months. This time was used to finalise both the case study sites for Stream Three and the secondary outcomes for Stream Two, for which a comprehensive dictionary of data definitions has been completed (Additional file 3). We believe that this is an appropriate juncture to publish the protocol and data dictionary, now that the sites and outcomes to be measured have been determined. During this time the Stream One surveys have been completed, as have the first round of interviews in Stream Three. The analysis of the data from these streams will be undertaken in 2012, in parallel with the quantitative data collection and analysis for Stream Two.

There are other important related questions this project is not able to explore. One such question is whether patient experiences of ED treatment has changed as a consequence of implementation of the target. Although we have attempted to address areas of timeliness of care previously identified as being of concern to patients in our clinical outcomes, we recognise that this is only a surrogate for the patient's perspective in assessing the quality of healthcare. Another question not addressed by the current project is the effect of implementation of the target on the experiences of ED staff [76]. We are currently exploring how such questions can be better captured in the context of our research.

## Ethical approval

Ethical approval for this study was granted by the New Zealand Multi-Region Ethics Committee (MEC 10/06/060) on 1st July 2010.

## Competing interests

The authors declare that they have no competing interests.

## Authors' contributions

PC, PJ, AH and TA developed Stream One of the research. PJ, SW, SA, JS and AH developed Stream Two, PJ and AH reviewed the literature to determine initial candidate markers of care, AH drafted the data dictionary. JS conducted the sample size calculation and will undertake statistical analysis. LC, TT and PC designed Stream Three. PR and EC were included at all stages of research design for all streams. TT has overall responsibility for Stream Four. PJ and AH drafted this manuscript, which was reviewed and revised by the other authors. All authors read and approved the final manuscript.

## Authors' information

The Shorter Stays in Emergency Departments National Research Project team is a collaboration of researchers from the School of Population Health (Faculty of Medical and Health Sciences, University of Auckland) and the Emergency Department Research Team at the Auckland District Health Board. The lead investigators are Peter Jones and Linda Chalmers. The investigators are Shanthi Ameratunga, Toni Ashton, Peter Carswell, Elana Curtis, Papaarangi Reid, Joanna Stewart, Tim Tenbensel and Susan Wells. The research fellows are Alana Harper and James LeFevre, the data manager is Denish Kumar and the research administrator is Jayshree Ramesh-Sukha

## Pre-publication history

The pre-publication history for this paper can be accessed here:

http://www.biomedcentral.com/1472-6963/12/45/prepub

## Supplementary Material

Additional file 1 **Survey of Initiatives Made and Resources Used to Help Meet the Shorter Stays in Emergency Departments Target (Stream One)**. Click here for file

Additional file 2 **Interview Schedule for Qualitative Interviews (Stream Three)**. Click here for file

Additional file 3 **Data Dictionary for Quantitative Outcomes (Stream Two)**. Click here for file
