# Supplementary material for: Implementing performance improvement in New Zealand emergency departments: the six hour time target policy national research project protocol
Source: BMC Health Serv Res. 2012 Feb 21;12:45. doi: 10.1186/1472-6963-12-45 (PMC3311075; doi:10.1186/1472-6963-12-45)
Supplement: Additional file 1 — Survey of Initiatives Made and Resources Used to Help Meet the Shorter Stays in Emergency Departments Target (Stream One). [file 1472-6963-12-45-S1.DOC]

**Appendix 1**

**Shorter Stays in Emergency Departments National Research Project**

**SURVEY**

(Emergency Department Clinical Directors and Service Managers, DHB Management)

**NAME OF RESEARCHERS: Dr Peter Jones, Dr Peter Carswell, A Prof Toni Ashton, Ms Linda Chalmers, Dr Susan Wells, Dr Tim Tenbensel, A Prof. Papaarangi Reid, Dr Elana Curtis, Ms Joanna Stewart, Prof Shanthi Ameratunga.**

Thank you for helping with this research, which aims to investigate how the ‘Shorter Stays in Emergency Departments’ policy was implemented across New Zealand. The survey is in 4 sections. Section A covers changes made in ED, section B covers changes within the rest of the hospital and section C covers changes made in the wider community. Section D relates to DHB funding changes.

Please complete all sections that you believe apply to your role, or that you have knowledge of. If the section does not apply to you, please indicate the person in your DHB that you think would best be able to answer that section in the space provided. If there is insufficient space for you to complete your answer, please continue on a separate sheet.

**Shorter Stays in ED Survey**

1. **Site and Participant Information**
   1. DHB________________________________________________________________________
   2. Site________________________________________________________________________
   3. Role: ED Clinical Director ED Service Manager Other State____________________________________________________

**The Following Questions Relate to The Introduction of the Shorter Stays in ED (SSED) Health Target in NZ in July 2009. Please tick the box that applies or enter free text where appropriate**

1. **Who were the SSED Target Champion(s) in your Organisation?** **(Tick all that apply)**

No Champion CEO ED Clinical Director ED Service Manager

ED Nurse Manager ED SMO Other Clinical Director Other Manager

Other Nurse Manager Other SMO

State which department if not ED___________________________________________________

1. **When did your DHB start implementing changes to meet the SSED Target?__________________**
2. **CHANGES in the EMERGENCY DEPARTMENT**
   1. **Did the Physical Structure of your ED Change in order to help meet the SSED Target?**

Yes No Go to A.4

- - 1. **If Yes, please how did the physical structure of your ED change?**

| **Physical Change** | **Tick if Applies** | **Number**  **of Beds** | **Estimated**  **Cost ($NZ)** | **Date Change Occurred**  **(Month / Year)** |
| --- | --- | --- | --- | --- |
| A new ED was built |  | Old ED  New ED |  |  |
| New bed spaces were made available to the existing ED |  |  |  |  |
| A Short Stay ward was built in ED |  |  |  |  |
| Existing ED beds were redesignated Short Stay |  |  |  |  |
| Existing Other beds were redesignated Short Stay |  |  |  |  |

- 1. **Did the physical structure change in ED for any other reason than to meet the SSED Target?**

Yes No Go to A.3

- - 1. If Yes, please indicate why the physical structure of your ED changed

______________________________________________________________________

- - 1. If Yes, please indicate how the physical structure of your ED changed

______________________________________________________________________

- - 1. If Yes, how many new beds were created? ________________________
  1. **Please Comment on Any other Physical Changes made to the ED help meet the SSED Target**
  2. **Were more Staff employed in ED to help meet the SSED Target?**

Yes Please fill in the following table No Go to A.5

| **Role** | **Full Time Equivalent (FTE)** | **Date Employed**  **(month/year)** |
| --- | --- | --- |
| Senior Doctors |  |  |
| Medical Officers Special Scale |  |  |
| Registrars |  |  |
| House Officers |  |  |
| Clinical Nurse Specialists |  |  |
| Emergency Nurse Practitioners |  |  |
| Physicians Assistants |  |  |
| Senior Nurses |  |  |
| Nurses |  |  |
| Health Care Assistants (Non Clinical) |  |  |
| Orderlies |  |  |
| Clerical Staff |  |  |
| Managers |  |  |
| External Consultants |  |  |
| Other (state) |  |  |
| Other (state) |  |  |
| Other (state) |  |  |

- 1. **Were new roles created for ED staff to help meet the SSED Target?**

Yes Please fill in the following table No Go to A.6

| **Title** | **Role** | **FTE** | **Previous Role**  **(this person)** | **Previous FTE**  **(this person)** | **Date of Change**  **(month/year)** |
| --- | --- | --- | --- | --- | --- |
| Bed Coordinator |  |  |  |  |  |
| Flow Manager |  |  |  |  |  |
| Breach Avoidance Facilitator |  |  |  |  |  |
| Other (State Title) |  |  |  |  |  |
| Other (State Title) |  |  |  |  |  |

- 1. **Were staff changes in ED made for any other reason than to meet the SSED Target?**

Yes No Go to A.7

- - 1. If Yes, please indicate why the staff numbers/roles in your ED changed
    2. If Yes, please indicate how the staff numbers/roles in your ED changed
  1. **If you have any other comment on ED Staff Changes that occurred around the time of the SSED target introduction please make them here.**
  2. **Process Changes in the ED**

Please indicate the process changes that occurred in ED in order to help meet the SSED Target

| **ED Processes** | **Tick if this process changed in your ED** | **FTE Used on this change** | **Estimated Cost ($NZ)** | **Date of Change**  **(month/year)** |
| --- | --- | --- | --- | --- |
| Triage |  |  |  |  |
| Primary Nurse Assessment |  |  |  |  |
| Nurse Treatment / Protocol |  |  |  |  |
| Doctor Assessment / Protocol |  |  |  |  |
| Allied Health Assessment / Protocol |  |  |  |  |
| Availability of Investigations |  |  |  |  |
| Admission Process |  |  |  |  |
| Discharge Process |  |  |  |  |
| Other (state) |  |  |  |  |
| Other (state) |  |  |  |  |

- 1. **Please describe how the ED processes changed and/or make any comments on process changes in the ED that occurred in order to help meet the SSED target.**
  2. **If you feel someone else in your DHB is best equipped to answer Section A please state their name / contact details here:**

1. **CHANGES in the HOSPITAL**
   1. **Did the Physical Structure of your Hospital Change in order to help meet the SSED Target?**

Yes No Go to B.2

- - 1. **If Yes, how did the physical structure of your hospital change? (tick all that apply)**

| **Physical Change** | **Tick if Applies** | **Type of Bed**  **(Medical, Surgical, Rehabilitation etc)** | **Number**  **of Beds** | **Estimated**  **Cost ($NZ)** | **Date Change Occurred**  **(Month / Year)** |
| --- | --- | --- | --- | --- | --- |
| More Short Stay bed spaces were built |  |  |  |  |  |
| More Long Stay bed spaces were built |  |  |  |  |  |
| More Rehabilitation bed spaces were built |  |  |  |  |  |
| Other Physical Change (State) |  |  |  |  |  |

- 1. **Did the physical structure change in your hospital for any other reason than to meet the SSED Target?**

Yes No Go to B.3

- - 1. If Yes, please indicate why the physical structure of your hospital changed

______________________________________________________________________

- - 1. If Yes, please indicate how the physical structure of your hospital changed

______________________________________________________________________

- 1. **Please Comment on Any other Physical Changes made to the Hospital help meet the SSED Target**
  2. **Were more Staff employed in the Hospital to help meet the SSED Target?**

Yes Please fill in the following table No Go to B.5

| **Role** | **Full Time Equivalent (FTE)** | **Department(s)** | **Date Started**  **(month/year)** |
| --- | --- | --- | --- |
| Senior Doctors |  |  |  |
| Medical Officers Special Scale |  |  |  |
| Registrars |  |  |  |
| House Officers |  |  |  |
| Clinical Nurse Specialists |  |  |  |
| Emergency Nurse Practitioners |  |  |  |
| Physicians Assistants |  |  |  |
| Senior Nurses |  |  |  |
| Nurses |  |  |  |
| Health Care Assistants (Non Clinical) |  |  |  |
| Orderlies |  |  |  |
| Clerical Staff |  |  |  |
| Managers |  |  |  |
| External Consultants |  |  |  |
| Other (state) |  |  |  |
| Other (state) |  |  |  |
| Other (state) |  |  |  |

- 1. **Were new roles created for Hospital staff to help meet the SSED Target?**

Yes Please fill in the following table No Go to B.6

| **Title** | **Role** | **FTE** | **Date Created (month/year)** | **Previous Role**  **(this person)** | **Previous FTE**  **(this person)** |
| --- | --- | --- | --- | --- | --- |
| Bed Coordinator |  |  |  |  |  |
| Flow Manager |  |  |  |  |  |
| Breach Avoidance Facilitator |  |  |  |  |  |
| Other (State Title) |  |  |  |  |  |
| Other (State Title) |  |  |  |  |  |
| Other (State Title) |  |  |  |  |  |

- 1. **Were staff changes in Hospital made for any other reason than to meet the SSED Target?**

Yes No Go to B.7

- - 1. If Yes, please indicate why the staff numbers/roles in your Hospital changed
    2. If Yes, please indicate how the staff numbers/roles in your Hospital changed
  1. **If you have any other comment on Hospital Staff Changes that occurred around the time of the SSED target introduction please make them here.**
  2. **Process Changes in the Hospital**

**Please indicate the process changes that occurred in the hospital in order to help meet the SSED Target**

| **Hospital Processes** | **Tick if this process changed in your Hospital** | **FTE Used on this change** | **Estimated Cost ($NZ)** | **Date of Change**  **(month/year)** |
| --- | --- | --- | --- | --- |
| Admission Process |  |  |  |  |
| Primary Nurse Assessment |  |  |  |  |
| Nurse Treatment / Protocol |  |  |  |  |
| Doctor Assessment / Protocol |  |  |  |  |
| Allied Health Assessment / Protocol |  |  |  |  |
| Availability of Investigations |  |  |  |  |
| Discharge Process |  |  |  |  |
| Other (state) |  |  |  |  |
| Other (state) |  |  |  |  |

- 1. **Please describe how the processes changed in your hospital and/or make any comments on process changes in the hospital that occurred in order to help meet the SSED target.**
  2. **If you feel someone else in your DHB is best equipped to answer Section B please state their name / contact details here:**

1. **CHANGES in the COMMUNITY**
   1. **Did the Physical Structure of Community Healthcare change in order to help meet the SSED Target?**

Yes Please fill in table No Go to C.2 Unknown

- - 1. **If Yes, how did the physical structure of community healthcare change?**

| **Physical Change** | **Tick if Applies** | **Type of Beds / Intervention / Service** | **Number of Beds / Services** | **Estimated**  **Cost ($NZ)** | **Date Change Occurred**  **(Month / Year)** |
| --- | --- | --- | --- | --- | --- |
| More Rest Home Long Stay Residential Care bed spaces were built / opened |  |  |  |  |  |
| More Accident and Medical Clinics were built / opened |  |  |  |  |  |
| More General Practices were built / opened |  |  |  |  |  |
| Interventions to prevent ED attendances were made |  |  |  |  |  |
| Availability of Investigations |  |  |  |  |  |
| Other Physical Change (State) |  |  |  |  |  |

- 1. **Did the physical structure of community healthcare change for any other reason than to meet the SSED Target?**

Yes No Go to C.3 Unknown

- - 1. If Yes, please indicate why the physical structure of community healthcare changed

______________________________________________________________________

- - 1. If Yes, please indicate how the physical structure of community healthcare changed

______________________________________________________________________

- 1. **Please comment on any other Physical Changes made to community healthcare to** **help meet the SSED Target**
  2. **Were more Staff employed in the Community to help meet the SSED Target?**

Yes Please fill in table No Go to C.5 Unknown

| **Role** | **Full Time Equivalent (FTE)** | **Site / Role** | **Date Started**  **(month/year)** |
| --- | --- | --- | --- |
| General Practitioners |  |  |  |
| Medical Officers Special Scale |  |  |  |
| Geriatricians |  |  |  |
| Clinical Nurse Specialists |  |  |  |
| Emergency Nurse Practitioners |  |  |  |
| Physicians Assistants |  |  |  |
| Senior Nurses |  |  |  |
| Nurses |  |  |  |
| Health Care Assistants (Non Clinical) |  |  |  |
| Clerical Staff |  |  |  |
| Managers |  |  |  |
| External Consultants |  |  |  |
| Other (state) |  |  |  |
| Other (state) |  |  |  |
| Other (state) |  |  |  |

- 1. **Were new roles created for Community staff to help meet the SSED Target?**

Yes Please fill in the following table No Go to C.6

| **Title** | **Role** | **FTE** | **Date Created (month/year)** | **Previous Role**  **(this person)** | **Previous FTE**  **(this person)** |
| --- | --- | --- | --- | --- | --- |
|  |  |  |  |  |  |
|  |  |  |  |  |  |
|  |  |  |  |  |  |
|  |  |  |  |  |  |
|  |  |  |  |  |  |
|  |  |  |  |  |  |

- 1. **Were staff changes in the Community made for any other reason than to meet the SSED Target?**

Yes No Go to C.7 Unknown

- - 1. If Yes, please indicate why the staff numbers/roles in your Community changed
    2. If Yes, please indicate how the staff numbers/roles in your Community changed
  1. **If you have any other comment on Community Staff Changes that occurred around the time of the SSED target introduction please make them here.**
  2. **Process Changes in the Community**

**Please indicate the process changes that occurred in the Community in order to help meet the SSED Target**

| **Community Processes** | **Tick if this process changed in your Community** | **FTE Used on this change** | **Estimated Cost ($NZ)** | **Date of Change**  **(month/year)** |
| --- | --- | --- | --- | --- |
| Admission Process |  |  |  |  |
| Nurse Treatment / Protocol |  |  |  |  |
| Doctor Assessment / Protocol |  |  |  |  |
| Allied Health Assessment / Protocol |  |  |  |  |
| Availability of Investigations |  |  |  |  |
| Discharge Process |  |  |  |  |
| Other (state) |  |  |  |  |
| Other (state) |  |  |  |  |

- 1. **Please describe how the processes in the Community changed and/or make any comments on process changes in the community that occurred in order to help meet the SSED target.**
  2. **If you feel someone else in your DHB is best equipped to answer Section C please state their name / contact details here:**

1. **Funding Changes**
   1. **Was your DHB budget allocation altered as a result of the SSED Target**

Yes No Go to D.2 Unknown

- - 1. **Please state how the budget allocation changed**

**Increased Amount ($NZ) _____________________**

**Decreased Amount ($NZ) _____________________**

- 1. **Did your DHB reallocate funding as a result of the SSED Target (2008-2010)?**

Yes please fill in table No Go to D.3 Unknown

| Site of Care | Budget Increased  Yes/No | Amount Increased  $NZ | Budget Decreased  Yes / No | Amount Decreased  $NZ |
| --- | --- | --- | --- | --- |
| ED |  |  |  |  |
| Hospital |  |  |  |  |
| Primary Care |  |  |  |  |
| Long Term Residential Care |  |  |  |  |
| Other |  |  |  |  |
| Other |  |  |  |  |

- 1. **Please make any other comments related to funding and budgets you think may be relevant to the SSED target**
  2. **If you feel someone else in your DHB is best equipped to answer Section D please state their name / contact details here:**
