# Supplementary material for: Implementing performance improvement in New Zealand emergency departments: the six hour time target policy national research project protocol
Source: BMC Health Serv Res. 2012 Feb 21;12:45. doi: 10.1186/1472-6963-12-45 (PMC3311075; doi:10.1186/1472-6963-12-45)
Supplement: Additional file 2 — Interview Schedule for Qualitative Interviews (Stream Three). [file 1472-6963-12-45-S2.DOC]

**Appendix 2**
