# Supplementary material for: Implementing performance improvement in New Zealand emergency departments: the six hour time target policy national research project protocol
Source: BMC Health Serv Res. 2012 Feb 21;12:45. doi: 10.1186/1472-6963-12-45 (PMC3311075; doi:10.1186/1472-6963-12-45)
Supplement: Additional file 3 — Data Dictionary for Quantitative Outcomes (Stream Two). [file 1472-6963-12-45-S3.PDF]

SHORTER STAYS IN EMERGENCY DEPARTMENTS NATIONAL RESEARCH PROJECT

-RAW DATA DEFINITIONS-

STUDY SITES

Dr Peter Jones – Co Principal Investigator
Director Emergency Medicine Research
Auckland District Health Board

Dr Alana Harper – Research Fellow,
Emergency Medicine
Auckland District Health Board

Mr Denish Kumar – Data Manager
SSED NRP Project Team

Preface:

This data dictionary has been developed to provide a systematic protocol for quality indicator data collection, at the 4 study sites, in stream two of the Shorter Stays in Emergency Departments National Research Project.

A further dictionary defining derived values will be developed following data collection and before data analysis begins.

General Resources:

- College of Emergency Medicine (CEM UK): Emergency Medicine Minimum Dataset V0.7[1]
- Department of Health (DH UK): A&E Quality Indicators Data Definitions 2010[2].
- Australian Council on Healthcare Standards (ACHS): Australasian Clinical Indicator Report 2001-2009[3] and draft Emergency Medicine Indicators 2011[4]
- The Good Indicators Guide (NHS UK)[5]
- Measuring and Improving Quality in Emergency Medicine (Graff 2002)[6]
- Emergency Department Performance Measures and Benchmarking Summit (Definitions of Terms)[7]
- Development of a Consensus on Evidence-Based Quality of Care Indicators for Canadian Emergency Departments: ICES Investigative Report[8]
- Quality, Performance and Performance Indictors: ACEM Quality Sub-Committee Meeting Sept 2010[9]
- Ministry of Health NZ (MOH): Ethnicity Data Protocols for the Health and Disability Sector, Wellington 2004[10].

Contents:

General Resources: 2

Contents: 3

1.0 Terminology 11

Definitions: 11

Abbreviations: 13

Beds: 14

Procedures for collecting Process Indicator variables: 15

Procedures for extracting data for Clinical Indicator Data Collection forms: 17

2.0 Event Identifiers 18

2.1 NHI 18

2.2 DHB Identifier 19

2.3 Hospital Identifier 20

2.4 Event Identifier 21

2.5 Diagnostic (ICD) Code 22

2.6 Procedure Code 23

3.0 Event Demographic Values 24

3.1 Patient DOB 24

3.2 Patient DOD 25

3.3 Patient Age 26

3.4 Patient Gender 27

3.5 Patient Ethnicity Level 1 28

3.6 Patient Ethnicity Level 2 29

3.7 Patient Ethnicity Level 3 30

3.8 Patient Ethnicity Level 4 31

3.9 Domicile Code 34

3.10 Deprivation Scale Code 35

3.11 NZ Residency Status 36

3.12 Treatment Eligibility 37

4.0 ED Event Arrival and Discharge Values 38

4.1 ED Arrival Mode 38

4.2 Referral Type 39

4.3 Referral Reason 40

4.4 Admission Type 41

4.5 ACC Status ... 42
4.6 Triage Category ... 43
4.7 Triage to (Location) ... 44
4.8 ED Disposition ... 45
4.9 ED Discharge Type ... 46
4.10 ED Discharge Destination Type ... 47
4.11 Ward Discharge Type ... 48
4.12 Ward Discharge Destination Type ... 49
4.13 Discharge Ward Type ... 50
5.0 Event Time Stamp Values ... 51
5.1 Presentation Time ... 52
5.2 Triage Time ... 53
5.3 Assessment Time ... 54
5.4 Referral Time ... 55
5.5 Inpatient Team Time ... 56
5.6 Time of Bed Request ... 57
5.7 Time of Bed Allocation ... 58
5.8 ED Departure Time ... 59
5.9 ED SSU Admit / Assign Time ... 60
5.10 Ward Admission Time ... 61
5.11 Ward Departure Time ... 62
5.12 Ward Leave Start Time ... 63
5.13 Ward Leave End Time ... 64
6.0 Emergency Department General Data ... 65
6.1 ED FTE: Medical ... 65
6.2 ED FTE: Nursing ... 66
6.3 ED FTE: HCA (Hospital Aid or similar) ... 67
6.4 ED FTE: Orderlies ... 68
6.5 ED FTE: Ward Clerk ... 69
6.6 ED FTE: Managers ... 70
6.7 Bed Occupancy (at Midnight) - Hospital (%) ... 71
6.8 Bed Occupancy (at Midnight) – APU / ADU (%) ... 72
7.0 Outcomes ... 73
7.1 Primary Outcomes ... 73

7.1.1 Length of Stay ED (ED LOS – DHB Value) 73
7.1.2 Total Length of Stay (TLOS - DHB) 74
7.1.3 ED Re-Attendance < 48 Hours (Derived) 75
7.1.4 ED Re-Attendance < 72 Hours (Derived) 76
7.1.5 Re-Attendance Inpatients < 72 hours (Derived) 77
7.1.6 Access Block (Derived) 78
7.2 Secondary Outcomes: 79
7.2.1 Mortality 79
7.2.2 Hospital Mortality 80
7.2.3 ED "Did Not Wait" (DNW) 81
8.0 Data Recording (Data Collection Forms) 82
9.0 Clinical Confounder Variables 85
9.1 ADULT CONFOUNDERS 86
9.1.1 Chronic Renal Failure 86
9.1.2 Chronic Obstructive Pulmonary Disease 87
9.1.3 Diabetes Mellitus 88
9.1.4 Smoking (Tobacco) 89
9.1.5 Dementia 90
9.1.6 Stroke 91
9.1.7 Hypertension 92
9.1.8 Ischaemic Heart Disease 93
9.1.9 Congestive Heart Failure 94
9.1.10 Anticoagulation 95
9.1.11 Immunosuppression 96
9.1.12 ASA Status (Pre-Operatively) 97
9.1.13 Independence 98
9.2 PAEDIATRIC CONFOUNDERS 99
9.2.1 Pre-Term Birth 99
9.2.2 Pre-Term Birth Weight 100
9.2.3 Congenital Heart Disease 101
9.2.4 Chronic Lung Disease 102
9.2.5 Smoking (Tobacco) in the Home 103
9.2.6 Anticoagulation 104
9.2.7 Immunosuppression 105

9.2.8 ASA Status (Pre-Operatively) ... 106
9.2.9 Independence ... 107
10.0 Clinical Indicators ... 108
10.1 Time to Reperfusion in Acute Myocardial Infarction (AMI) ... 109
10.1.1 First ECG Time ... 116
10.1.2 Diagnostic ECG Time ... 117
10.1.3 Thrombolytic Time ... 118
10.1.4 PCI Time ... 119
10.1.5 Failed Thrombolysis ... 120
10.1.6 Rescue PCI ... 121
10.1.7 Primary CABG Surgery ... 122
10.1.8 Primary CABG Time ... 123
10.2 Time to Surgery for Fracture Neck of Femur (#NOF) ... 124
10.2.1 Diagnostic X-Ray Location ... 127
10.2.2 Diagnostic X-Ray Time ... 128
10.2.3 # NOF Surgery Start Time ... 129
10.3 Time to Surgery for Appendectomy in Appendicitis ... 130
10.3.1 Serum WCC ... 132
10.3.2 Serum CRP ... 133
10.3.3 Scan Type Pre-Op ... 134
10.3.4 Scan Time ... 135
10.3.5 Result Scan Pre-Op ... 136
10.3.6 Surgery Start Time ... 137
10.3.7 Appendix Appearance OR ... 138
10.3.8 Appendiceal Histology ... 139
10.4 Time to Antibiotics in Severe Sepsis ... 140
10.4.1 Primary Site of Infection ... 154
10.4.2 Ix: Peripheral Blood Cultures ... 155
10.4.3 Number of Peripheral Blood Cultures ... 156
10.4.4 Ix: Urine Culture ... 157
10.4.5 Ix: Pus Swab ... 158
10.4.6 Ix: CSF Specimen ... 159
10.4.7 Ix: Catheter Tip Culture ... 160
10.4.8 Ix: Sputum Culture ... 161

10.4.9 Ix: Faecal Culture ... 162
10.4.10 Ix: Aspirated Pus Culture ... 163
10.4.11 Ix: CXR ... 164
10.4.12 Ix: US Scan ... 165
10.4.13 Ix: CT Scan ... 166
10.4.14 Ix: MRI Scan ... 167
10.4.15 Ix: ERCP ... 168
10.4.16 Ix: TTE ... 169
10.4.17 Ix: TOE ... 170
10.4.18 ED First Antibiotic Given (3 fields) ... 171
10.4.19 ED First Antibiotic Route (3 fields) ... 172
10.4.20 ED First Antibiotic Time (3 fields) ... 173
10.4.21 Predominant Culture Growth ... 174
10.4.22 ED Antibiotic Sensitivity (3 fields) ... 175
10.4.23 ED ANY Antibiotic Appropriate ... 176
10.4.24 ED Best Guess Antibiotic Appropriate ... 177
10.5 Time to Analgesia for ED Patients ... 178
10.5.1 Pre-Hospital Analgesia Admin ... 182
10.5.2 Type of Pre-Hospital Analgesia ... 183
10.5.3 Route of Pre-Hospital Analgesia ... 184
10.5.4 Arrival Pain Score ED ... 185
10.5.5 Type of Pain Score Arrival ED ... 186
10.5.6 Raw Pain Score Arrival ED ... 187
10.5.7 Pain Score Categorical Arrival ED ... 188
10.5.8 First Assessment Pain Score ED ... 189
10.5.9 Type of Pain Score First Assessment ED ... 190
10.5.10 Raw Pain Score First Assessment ED ... 191
10.5.11 Pain Score Categorical First Assessment ED ... 192
10.5.12 First Analgesia Prescribe Time ... 193
10.5.13 First Analgesia Time (4 entries) ... 194
10.5.14 Type of Analgesia (4 Entries) ... 195
10.5.15 Route of Analgesia (4 Entries) ... 196
10.5.16 Time First IV Opiate Analgesia ... 197
10.5.17 Number IV Opiate Doses ... 198

10.5.18 Total IV Opiate Dose 199
10.5.19 Pain Score Reassessment: First Post-Analgesia 200
10.5.20 Time Pain Score Re-Assessed: First Post- Analgesia 201
10.5.21 Type of Pain Score Reassessment: First Post-Analgesia 202
10.5.22 Raw Pain Score Reassessment: First Post-Analgesia 203
10.5.23 Pain Score Categorical Reassessment: First Post-Analgesia 204
10.5.24 Other Subjective Pain Reassessment: Post-Analgesia 205
10.5.25 Raw Subjective Pain Reassessment: Post-Analgesia 206
10.5.26 Type of Pain Score: Lowest ED 207
10.5.27 Raw Pain Score: Lowest ED 208
10.5.28 Pain Score Categorical: Lowest ED 209
10.6 Time to Treatment in Acute Asthma for ED Patients 210
10.6.1 Pre-Hospital Treatment Given 218
10.6.2 Pre-Hospital Treatment Admin 219
10.6.3 Pre-Hospital Medications 220
10.6.4 ABG Taken 221
10.6.5 CXR Taken 222
10.6.6 CXR Time 223
10.6.7 Steroids Given in ED 224
10.6.8 Route of Steroids 225
10.6.9 First Steroid Time in ED 226
10.6.10 Salbutamol Given in ED 227
10.6.11 Route of Salbutamol 228
10.6.12 First Salbutamol Time in ED 229
10.6.13 Ipratropium Given in ED 230
10.6.14 Route of Ipratropium 231
10.6.15 First Ipratropium Time in ED 232
10.6.16 Adrenaline Given (at any time) 233
10.6.17 Oxygen Therapy Start Time in ED 234
10.6.18 Discharge Prescription Steroids 235
10.7 CT Head and Time to Operating Room in Acute Head Injury 236
10.7.1 Time of Injury 248
10.7.2 Ambulance Arrival Time @ Scene 249
10.7.3 Pre-Hospital GCS (lowest) 250

10.7.4 Pre-Hospital Motor Score Adults (lowest) 251
10.7.5 Pre-Hospital Motor Score Paeds (lowest) 252
10.7.6 Pre-Hospital Pupils (worst) 253
10.7.7 Pre-Hospital CNS Depressants Medications given. 254
10.7.8 Pre-Hospital Medication Route 255
10.7.9 Intubation Pre-Hospital 256
10.7.10 ED GCS Arrival and 2 Hours post-injury 257
10.7.11 ED Motor Score Adults Arrival and 2 Hours post-injury 258
10.7.12 ED Motor Score Paeds Arrival and 2 Hours post-injury 259
10.7.13 ED Pupils Arrival and 2 Hours post-injury 260
10.7.14 Intubation in ED 261
10.7.15 Duration Loss of Consciousness 262
10.7.16 Amnesia 263
10.7.17 Duration Amnesia 264
10.7.18 Headache since TBI 265
10.7.19 Seizure since TBI 266
10.7.20 Any Focal Neurological Deficit since TBI 267
10.7.21 Number of Vomits 268
10.7.22 Mechanism of Injury 269
10.7.23 Intoxication with ETOH 270
10.7.24 Serum ETOH Level 271
10.7.25 Intoxication with Other Recreational Substances 272
10.7.26 Vault Skull Fracture 273
10.7.27 Base of Skull Fracture Signs 274
10.7.28 Deterioration 275
10.7.29 Paeds: TBI < 1 years 276
10.7.30 Paeds: Specialist Referral? NAI 277
10.7.31 CT Head Scan Request Time 278
10.7.32 CT Head Scan Time 279
10.7.33 CT Scan Result (5 Fields) 280
10.7.34 C-Spine Imaged 281
10.7.35 C-Spine Imaging Time 282
10.7.36 ICP Monitor Placed 283
10.7.37 ICP Monitor Time 284

10.7.38 Neurosurgical Consultation 285
10.7.39 Neurosurgical Referral or Transfer 286
10.7.40 To OR 287
10.7.41 Surgery Start Time 288
10.7.42 Type of OR Procedure 289
10.8 Discharge Information 290
10.8.1 Discharge Letter Done 293
10.8.2 Discharge Letter Date 294
10.8.3 Discharge Diagnosis 295
10.8.4 Discharge Treatment Information 296
10.8.5 Treatment Complications Information 297
10.8.6 Procedures Information 298
10.8.7 Procedure Complications Information 299
10.8.8 GP-Specific Ongoing Care Information 300
10.8.9 Patient-Specific Ongoing Care Information 301
10.8.10 Discharge Medication Information 302
10.8.11 Review Information 303
10.8.12 Overall Adequacy Discharge Information 304
10.8.13 Patient-Specific Information – Clinical Notes 305
11.0 Appendices 306
11.1 Appendix 1: Example Data Source Chart – for greater detail see DHB’s Document 306
11.2 Appendix 2: ICD-10-AM Classification of Diseases Codes 308
11.3 Appendix 3: ACHI Classification of Procedure Codes 315
12.0 References 320

1.0 Terminology

Definitions:

Emergency Department (ED): ACEM (Australasian College for Emergency Medicine) definition[11]. "The Emergency Department (ED) is the dedicated area in a hospital that is organised and administered to provide a high standard of emergency care to those in the community who perceive the need for, or are in need of acute or urgent care, including hospital admission". Emergency Department includes all adult and paediatric events, for all DHB's.

ED Short Stay Unit (SSU): ARCHI (Australian Resource Centre for Healthcare Innovations) definition[12, 13]. "ED Short Stay Units (SSU's) have been developed to provide a short period of assessment, course of therapy or observations for a group of patients who no longer require active ED care. In the past these patients would have just remained in the ED. These units are designed to provide short-term (<24 hours) assessment and/or therapy for select conditions in order to streamline the episode of care. SSU front loads resources to provide an intensive period of evaluation, treatment and supervision. The emphasis is on enhancing patient flow through ED by allowing for early transfer out and improving ED bed access"

Admission and Planning Unit (APU), Acute Diagnostic Unit (ADU) or similar: Referrals from Primary Care, other hospital specialists or outpatient clinics, to inpatient specialties may be directed here, bypassing the ED. The APU / ADU patients do not have a formal LOS time target. This may not be applicable to all hospitals.

Inpatient Ward: An area in the Hospital where secondary qualified medical care is provided on an ongoing basis by a named medical specialist. Most patients are admitted to an inpatient wards after transfer from an Emergency Medicine Speciality or ED or APU.

Ethnicity: is ethnicity as collected and recorded for the patient at event and is current at the time of data collection for period of study. Ethnicity data can be sourced from:

- NHI (at event) = NMDS (Ministry of Health National Minimum Data Set) prioritized
- NHI ethnicity data (updated for changes along a continuum).
- NNAPC (National Non-Admitted Patients Collection). These people do not have a hospital event created. They spent less than 3 hours in hospital; therefore do not meet the criteria for admission and thus a coded diagnosis. Prior to 2010 reporting was intermittent.

Ethnicity is the ethnic group or groups that people identify with or feel they belong to. Ethnicity is a measure of cultural affiliation, as opposed to biological, race, ancestry, nationality or citizenship. Ethnicity is self perceived and people can belong to more than one ethnic group[14]:

- NZ Ministry of Health: "Ethnicity is self-identified and can change over time".
- "The best method of collecting ethnicity data is to allow people to complete the ethnicity question themselves[15]"

For this reason we are using ethnicity as described by the person at the time of their health-event, within the study time period. Ethnicity Data will be obtained from Levels 1-4 in order to facilitate multiple analyses within the study (decisions will be made regarding the appropriate use of ethnicity data level or prioritised ethnicity data depending on the analysis required).

Ethnicity data has not always been collected accurately in NZ[15]. Hauora IV (2007)[16] estimates an undercount of 5-15% of people identifying with Māori ethnicity, by census collected ethnicity data between 1991 and 2006 due to collection methods:

> "There are a number of ways in which Māori are defined in official statistics. Having an overview of these ways helps us to interpret health statistics and understand something more about the context of health status and disparities in health experiences and outcomes[16]".
>
> "Accurate ethnicity data is important to enable this comparison (between Māori and non-Māori). Previously however, official health data have been shown to undercount Māori. This leads to a mismatch between numerators and denominators that can bias results when population census denominator data are used to calculate rates[16]".
>
> "Hospitalisations and cancer registrations continue to undercount Māori. This undercount was estimated by linkage to other datasets with more reliable ethnicity data From these estimates, Māori adjusters were created and applied to hospital and cancer registration data to 'adjust' for the undercount of Māori in these datasets. Hospitalisation rates were calculated from 2003 to 2005[16, 17]".

The SSED NRP held discussions regarding whether to adjust the ethnicity data or not (in order to account for the undercount of Māori in hospital datasets). The SSED NRP research team, including University of Auckland Te Kupenga Hauora Māori researchers (Associate Professor Papaarangi Reid and Dr Elana Curtis, Te Kupenga Hauora Māori, Faculty of Medical and Health Sciences), in conjunction with Te Rōpū Rangahau Hauora a Eru Pōmare researchers Dr Donna Cormack and Dr Ricci Harris (Eru Pōmare Māori Health Research Centre, Wellington) agreed that adjustment is NOT required for hospital ethnicity data in the following contexts:

- Where the ethnicity collected for the numerator is the same as the ethnicity collected for the denominator.
- Where the calculation of population rates using Census ethnicity as the denominator is not required (as there will not be numerator/denominator bias present within the dataset).

Ethnicity Data will be collected where possible at Level 4 (during hand-data extractions). Electronic data provided by NZHIS and DHB's will be to Level 2 and Level 2 prioritised (they are unable to extract to Level 4 data).

Diagnostic and Procedure coding data: collected as a separate extract referenced to the NHI of the patient and the Event Identifier in tabular or extract form (see Appendix 2 11.2 and Appendix 3 11.3).

ED FTE Resource: captured from the Hospital Financial Forecasting and Reporting System (FFARS) or the Human Resource Management system (HRMS) for study years 1996 to present in an extract or tabular form.

Abbreviations:

Time Abbreviations:

- HH: Hour range from 00 to 23
- MM: Minute range from 00 to 59
- SS: seconds range from 00 to 59

A 24 hour time period is from 00:00:00 to 23:59:59

For time values Midnight is 00:00:00

Date Abbreviations

- DD: Day range from 01 to 31
- MM: Month range from 01 to 12
- CC: Century Range 18,19 or 20
- YY: Year range 00 to 99

Layout Abbreviations

- N = Numeric value
- A = Alpha value

Beds:

Inpatient Bed: is a Bed, not a trolley. It is *in* a bed space which is set up to provide safe care to the patient, i.e.: oxygen and suctioning equipment available.

- Excludes Clinic and Day stay Beds
- Should NOT include Procedure Rooms and Whānau Rooms on the Ward.
- Beds in the ED are *not* counted as Inpatient Beds and have a separate census from the Wards.
- ED and APU Procedure Rooms and Whānau Rooms are NOT counted as defined ED or APU beds, as prolonged care is not delivered there.
- ED Short Stay Units Beds are not counted as inpatient beds by the Ministry of Health (MOH) and for the purposes of the SSED NRP these will be counted separately.

Resourced Beds: A Resourced Bed is one which is functional in a bed capacity (as above) being an "Inpatient Bed" but it is also staffed by Nursing Staff with the appropriate skill mix.

Beds: may be available, but may not be able to be used if it is not "resourced" correctly (e.g. for outliers). The "bed" in this context represents not simply a place for the patient to sleep, but the services that go with being cared for by the medical facility: admission processing, physician time, nursing care, necessary diagnostic work, appropriate treatment, and so on.

Funded Resourced Beds: these are staffed as above and funded with allocation of monies from the patients designated CBU (for example for outliers). An outlier is defined as: "a patient who is being cared for on a ward whose specialty alignment is not that of the patient's home ward". The Home ward is "The ward that provides the specialised medical and nursing care required by the patient". Therefore if a patient is a surgical team patient, but on a medicine based ward they are a surgical outlier there – the bed is funded by Surgery but resourced by Medicine (but only if the nurse has an appropriate skill mix to cover the bed – if not, it may not be feasible to use the bed).

Procedures for collecting Process Indicator variables:

1. Electronic request by SSED research team to all 21 DHB Decision Support for variables as defined.
2. Electronic request by SSED research team to NZHIS for variables as defined.
3. Raw data checked and cleaned.
4. Cleaned data entered into final study table.
5. Calculated fields completed.

DHB Data Collection Diagram

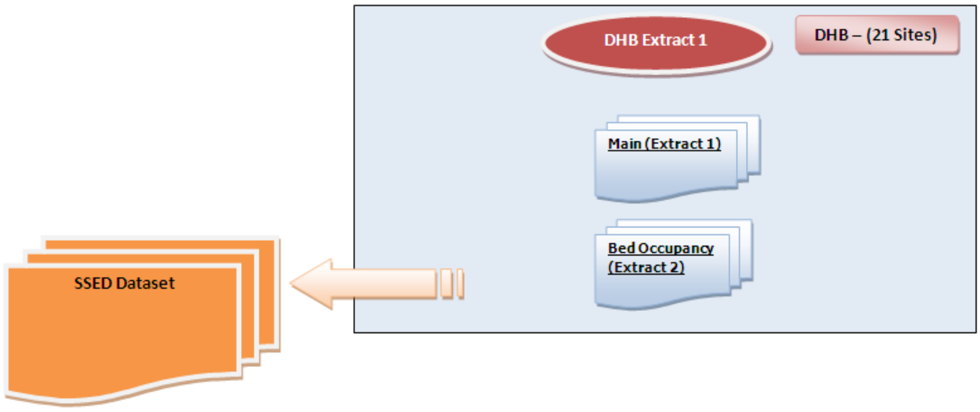

NZHIS Data Collection Diagram

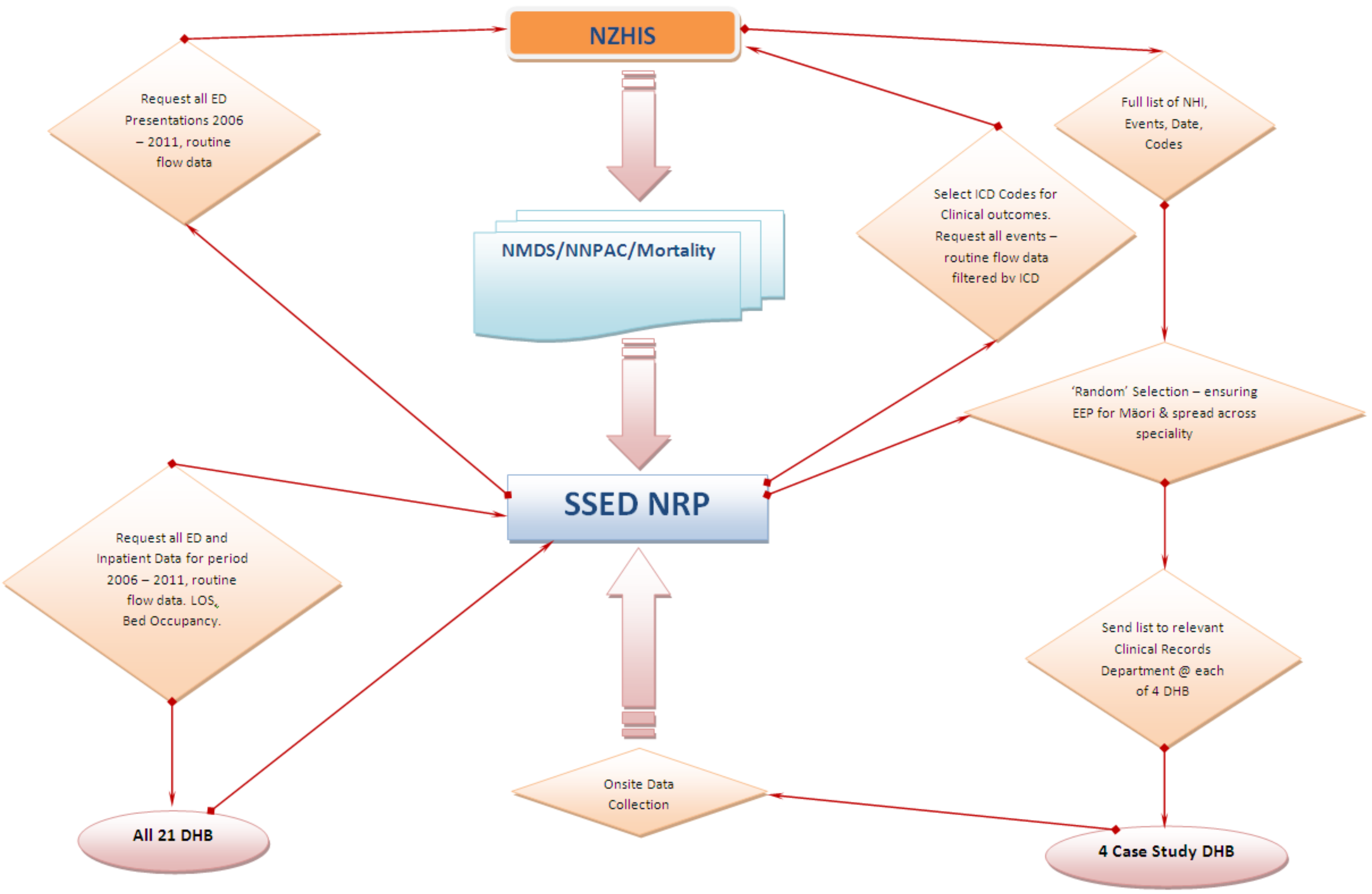

See Appendix 1 for complete Data Collection Diagram.

Procedures for extracting data for Clinical Indicator Data Collection forms:

"Random selection" of cases will be by ICD-10 Code groups within the study time period for Myocardial Infarction, Fracture Neck of Femur, CT Head for Traumatic Brain Injury, Asthma Treatment, Antibiotic in Severe Infection, Appendicectomy. For Pain Relief this will be a random selection from all **acute** hospital presentations within the study time period and for Discharge Summary this will be random selection from all ED presentations that are discharged from ED during the study time period.

The Data extracts to all clinical indicator data collection forms (both NZHIS and DHB Electronic Data) should be pulled in the following order for ease of transfer.

A - ICD Code (as provided to NZHIS)
B - Event Identifier
C - NHI
D - DOB
E - Age
F - DOD
G - Gender
H - Ethnicity (NZHIS reported)
I - Domicile Code
J - Deprivation Score
K - NZ Residential Status
L - Treatment Eligibility
M - Arrival Mode
N - ACC Status (only for Fracture Femur, CT Head and Pain extracts)
O - Triage Category
P - Triage Destination
Q - Presentation Time
R - Triage Time
S - Assessment Time
T - ED LOS (reported)
U - ED Discharge Type
V - ED Departure Time
W - Ward Admission Time (not for ED Discharge Extract)
X - Ward Discharge Type (not for ED Discharge Extract)
Y - Ward Departure Time (not for ED Discharge Extract)
Z - Hospital LOS (not for ED Discharge Extract - reported)
AA - Referral Reason (for Pain and Discharge extracts only)

2.0 Event Identifiers

2.1 NHI

Definition	National Health Index number (NHI Number). The unique identification number assigned to a healthcare user by the National Health Index (NHI) database.
Layout	AAANNNN (Alphanumeric: 3 letters 4 numbers – 7 characters)
Reported For	All Events
Description	The NHI number is the cornerstone of MOH data collections. It is a unique 7 character identification number assigned to a healthcare user by the National Health Index (NHI) database. It is stored in the NMDS in an encrypted form.<br><br>The NHI Number is a unique number given to every New Zealander at birth or first registration with health or disability services. The NHI holds the following information: name (including alternative names such as maiden names), NHI number, address, date of birth, sex, New Zealand resident status, ethnicity, and if appropriate, date of death, or flags indicating any medical warnings or donor information. Clinical information is not recorded on the NHI. Individual patients can be positively and uniquely identified for the purposes of treatment and care, and for maintaining medical records. It allows safe and secure identification of an individual to attempt to minimize the risk of wrong information[18].<br><br>A Patient must be registered on the NHI before any Health related episode.
Numerator (If Applicable)	n/a
Denominator (If Applicable)	n/a
Expressed As	String

National

2.2 DHB Identifier

Definition	Unique Code Identifying Individual DHB sites. This is the DHB the event has happened at – not the domicile DHB of the patient.
Layout	NNNN (Number: 4 Characters) or ANNN(1 Letter, 3 Numbers- 4 Characters)

Codeset

D011	Northland District Health Board	1011	Northland DHB
D021	Waitemata District Health Board	1021	Waitemata DHB
D022	Auckland District Health Board	1022	Auckland DHB
D023	Counties Manukau District Health Board	1023	Counties Manukau DHB
D031	Waikato District Health Board	2031	Waikato DHB
D042	Lakes District Health Board	2042	Lakes DHB
D047	Bay of Plenty District Health Board	2047	Bay of Plenty DHB
D051	Tairawhiti District Health Board	2051	Tairawhiti DHB
D061	Hawkes Bay District Health Board	2071	Taranaki DHB
D071	Taranaki District Health Board	3061	Hawke's Bay DHB
D081	Mid Central District Health Board	3081	Mid Central DHB
D082	Whanganui District Health Board	3082	Whanganui DHB
D091	Capital and Coast District Health Board	3091	Capital & Coast DHB
D092	Hutt Valley District Health Board	3092	Hutt Valley DHB
D093	Wairarapa District Health Board	3093	Wairarapa DHB
D101	Nelson Marlborough District Health Board	3101	Nelson-Marlborough DHB
D111	West Coast District Health Board	4111	West Coast DHB
D121	Canterbury District Health Board	4121	Canterbury DHB
D123	South Canterbury District Health Board	4123	South Canterbury DHB
D131	Otago District Health Board	4131	Otago DHB
D141	Southland District Health Board	4137	Otago Dental School
		4141	Southland DHB
		8559	Venturo
		8630	Queen Elizabeth Hospital
		8656	Mobile Surgical Bus

Reported For	All Events
Description	Ministry of Health assigned DHB codes. Classified as 'Agency Code' with Agency type code of '01' in NMDS
Numerator (If Applicable)	n/a
Denominator (If Applicable)	n/a
Expressed As	Categorical

National

2.3 Hospital Identifier

Definition	Unique Code identifying individual Hospital Sites used as Study Sites (for the collection of the quality indicator data).
Layout	AAA (3 Characters)
Codeset	ACH – Auckland City Hospital<br>NSH - North Shore Hospital (WDHB)<br>WTH – Waitakere Hospital (WDHB)<br>MMH – Middlemore Hospital (CMDHB)<br>WKH – Waikato Hospital<br>HBH – Hawkes Bay Hospital
Reported For	All Events
Description	Code assigned by SSED Study Group that uniquely identifies a healthcare facility.<br><br>A healthcare facility is a place, which may be a permanent, temporary, or mobile structure, which healthcare users attend or are resident in for the primary purpose of receiving healthcare or disability support services.
Numerator (If Applicable)	n/a
Denominator (If Applicable)	n/a
Expressed As	Categorical

**National**	*All hospitals that are not study sites listed above please reference to **DHB Identifier** and a unique code for each of your hospitals. Please add a lookup file together with the extract (Hospital Names/ Identifiers).*

2.4 Event Identifier

Definition	Hospital Event number as assigned by hospital "Patient Information Management System" (PIMS).
Layout	N (Number: X Character)
Codeset (If Applicable)	
Reported For	All Events
Description	Classified as "Event local identifier" in NMDS.<br><br>Event Identifier is a local, system-generated identifier to distinguish separately two or more events linked to an NHI.<br><br>'PIMS' in itself can relate to <u>any</u> generic Hospital based database that allows entry of new data, modification of data and stores data on health event information.
Numerator (If Applicable)	n/a
Denominator (If Applicable)	n/a
Expressed As	String

National

2.5 Diagnostic (ICD) Code

Definition	Diagnosis Identifier: from ICD10 codebooks
Layout	N (Number: X Character)
Codeset (If Applicable)	ICD-10-AM 6th Edition: See Appendix 2 11.2
Reported For	All Events
Description	ICD-10-AM and ACHI 6th Edition<br>Myocardial Infarction<br>Fracture Neck of Femur<br>Appendicitis<br>Severe Sepsis and Septic Shock<br>Asthma<br>Traumatic Brain Injury<br><br>The identification of events or cases for the study will be by ICD-Code (diagnosis codes and procedural codes) for the above conditions – each of which is being studied as a marker of quality care in the ED and wider hospital system. Each event associated with an ICD-Code will be the event included for data gathering – collecting all the raw data variables presented in this dictionary.
Numerator (If Applicable)	n/a
Denominator (If Applicable)	n/a
Expressed As	Categorical

National

2.6 Procedure Code

Definition	Procedure Identifier: from ICD10 codebooks (as a separate extract)
Layout	(X Character)
Codeset (If Applicable)	ICD-10-AM and ACHI 6th Edition: See Appendix 3 11.3
Reported For	All presentations
Description	See individual outcomes for ICD codes pertaining to that outcome.
Numerator (If Applicable)	n/a
Denominator (If Applicable)	n/a
Expressed As	Categorical

**National**	*Please submit as a separate extract.*

3.0 Event Demographic Values

3.1 Patient DOB

Definition	Patients Date of Birth
Layout	Date (DD/MM/CCYY – 10 Characters)
Reported For	All Events
Description	The date on which the person was born is required with full four-digit years and should be input in English date format.<br>Data stored under NHI.<br>This will be used to calculate Patient Age AT THE TIME OF EVENT
Numerator (If Applicable)	n/a
Denominator (If Applicable)	n/a
Expressed As	Numeric (Time)

National

3.2 Patient DOD

Definition	Patients Date of Death (if associated with event)
Layout	Date (DD/MM/CCYY – 10 Characters)
Reported For	All Events
Description	The date on which the person has died (if appropriate for event) is required with full four-digit years and should be input in English date format.<br>Data stored under NHI.<br>This will also be used to calculate age at death.
Numerator (If Applicable)	n/a
Denominator (If Applicable)	n/a
Expressed As	Numeric (Time)

National

3.3 Patient Age

Definition	Age of the Patient
Layout	NNN (Number: 3 characters)
Reported For	All Events
Description	The duration of a persons' life, or existence to date<br>How old the patient is in years as expressed in Ministry of Health Data.<br>If the patient is younger than 2 years old this will be expressed in months (where possible).<br>The Age is the patient's age at the time of the event, not at the time of data collection.<br>*Date of Event (DD/MM/CCYY) - Date of Birth (DD/MM/CCYY)*
Numerator (If Applicable)	n/a
Denominator (If Applicable)	n/a
Expressed As	Numeric (Year)

National

3.4 Patient Gender

Definition	Gender of the Patient
Layout	N (Number: 1 Character)
Codeset	DHB / NZHIS Codes as:<br>0 – Unknown<br>1 - Male<br>2 – Female<br>9 – Not Specified<br><br>NHI Codes As:<br>F = female<br>M = male<br>U = unknown
Reported For	All Events
Description	NHI stores data on Gender<br>NZHIS stores data on Gender Changes.<br>Gender is the genotypic and phenotypic distinction between Male and Female. It should be recorded as inferred by the patient and what gender they identify with; as is the case with gender reassignment (sex change) or intersex cases[1]. However gender changes are not relevant for the SSED NRP.
Numerator (If Applicable)	n/a
Denominator (If Applicable)	n/a
Expressed As	Categorical

National

3.5 Patient Ethnicity Level 1

Definition: Ethnic Group with which the Patient identifies

Layout: N (Number 1 Character)

Codeset (If Applicable):

Code	Description
1	European
2	Māori
3	Pacific Island
4	As�an
5	Middle Eastern/Latin American/African (was Other)
6	Other Ethnicity
9	Residual Categories*

Reported For: All Events

Description: The above are MOH defined sets[10, 19]– these are Ethnicities captured under a patient’s NHI for each discrete health-related event. Ethnicity data can be garnered from:

- NHI (at event)
- NMDS (Ministry of Health National Minimum Data Set: prioritized NHI ethnicity data, updated for changes along a continuum).
- NNAPC (National Non-Admitted Patients collection). These people do not have a hospital event created as they spent less than 3 hours in hospital and therefore do not meet the criteria for admission. Prior to 2010 reporting was intermittent.

Ethnicity is the ethnic group or groups that people identify with or feel they belong to. Ethnicity is a measure of cultural affiliation, as opposed to race, ancestry, nationality or citizenship. Ethnicity is self perceived and people can belong to more than one ethnic group.[14] NZ MOH: “Ethnicity is self-identified and can change over time”. For this reason we are using Ethnicity as described by the person at the time of their health-event within the study time period.

*The Ministry of Health currently uses Code 9 at Level 1 in National Data Collection reporting but Code 9 is not in the Ethnicity Data Protocols. These codes incorporate the changes as of July 2009[19] (in brackets on tables)

Expressed As: Categorical

National

3.6 Patient Ethnicity Level 2

Definition	Ethnic Group with which the Patient identifies
Layout	NN (Number: 2 Characters)
Codeset (If Applicable)	

Code	Description
10	European NFD
11	New Zealand European / Päkehä
12	Other European
21	Māori
30	Pacific peoples NFD
31	Samoan
32	Cook Island Maori
33	Tongan
34	Niuean
35	Tokelauan
36	Fijian
37	Other Pacific peoples
40	Asian NFD
41	Southeast Asian
42	Chinese
43	Indian
44	Other Asian
51	Middle Eastern
5	Latin American / Hispani[illegible]
53	African, or cultural group of African origin
61	Other Ethnicity (was 54 – Other)
94	Don't Know (addition)
95	Refused to Answer (addition)
96	Repeated value * not used
97	Response unidentifiable (now used)
98	Response outside scope * not used
99	Not stated

Reported For	All Events
Description	The above are MOH defined sets– these are Ethnicities reconciled under a patients' NHI.<br><br>These codes incorporate the changes as of July 2009 (in brackets in table)
Expressed As	Categorical

National

3.7 Patient Ethnicity Level 3

Definition: Ethnic Group with which the Patient identifies

Layout: NNN (Number: 3 Characters)

Codeset (If Applicable):

Code	Description
100	European NFD
111	New Zealand European / Päkehä
121	British and Irish (120 Removed)
122	Dutch
123	Greek (including Greek Cypriot)
124	Polish
125	South Slav (formerly Yugoslav)
126	Italian
127	German
128	Australian
129	Other European
211	Māori
300	Pacific peoples NFD
311	Samoan
321	Cook Island Maori
331	Tongan
341	Niuean
351	Tokelauan
361	Fijian
371	Other Pacific peoples
400	Asian NFD
410	Southeast Asian NFD
411	Filipino
412	Khmer / Kampuchean / Cambodian
413	Vietnamese
414	Other Southeast Asian
421	Chinese
431	Indian
441	Sri Lankan
442	Japanese
443	Korean
444	Other Asian
511	Middle Eastern
521	Latin American / Hispanic
531	African (or cultural group of African origin)
611	Other Ethnicity (was 541)
944	Don't Know (addition)
955	Refused to Answer (addition)
966	Repeated value *not used
977	Response unidentifiable (now used)
988	Response outside scope *not used
999	Not stated

Reported For: All Events

Description: The above are MOH defined sets.
These codes incorporate the changes as of July 2009 (in brackets in table)

Expressed As: Categorical

National

3.8 Patient Ethnicity Level 4

Definition: Ethnic Group with which the Patient identifies

Layout: NNNNN (Number: 5 Characters)

Codeset (If Applicable)

Code	Description
10000	European NFD
11111	New Zealand European / Päkehä
12000	Other European NFD
12111	Celtic NFD (addition of NFD)
12112	Channel Islander
12113	Cornish
12114	English
12115	Gaelic
12116	Irish
12117	Manx
12118	Orkney Islander
12119	Scottish (Scots)
12120	Shetland Islander
12121	Welsh
12199	British NEC
12211	Dutch / Netherlands
12311	Greek (including Greek Cypriot)
12411	Polish
12500	South Slav (formerly Yugoslav groups) NFD
12511	Croat / Croatian
12512	Dalmatian
12513	Macedonian
12514	Serb / Serbian
12515	Slovene / Slovenian
12516	Bosnian (addition)
12599	South Slav (formerly Yugoslav) NEC
12611	Italian
12711	German
12811	Australian
12911	Albanian
12912	Armenian
12913	Austrian
12914	Belgian
12915	Bulgarian
12916	Byelorussian
12917	Corsican
12918	Cypriot Unspecified
12919	Czech
12920	Danish
12921	Estonian
12922	Finnish
12923	Flemish
12924	French

Code	Description
37136	Phoenix Islander
37137	Pitcairn Islander
37138	Rotuman / Rotuman Islander
37139	Santa Cruz Islander
37140	Society Islander (including Tahitian)
37141	Solomon Islander
37142	Torres Strait Islander / Thursday Islander
37143	Tuamotu Islander
37144	Tuvalu Islander / Ellice Islander
37145	Vanuatu Islander / New Hebridean
37146	Wake Islander
37147	Wallis Islander
37148	Yap Islander
37199	Other Pacific peoples NEC
40000	Asian NFD
41000	Southeast Asian NFD
41111	Filipino
41211	Khmer / Kampuchean / Cambodian
41311	Vietnamese
41411	Burmese
41412	Indonesian (including Javanese / Sundanese / Sumatran)
41413	Lao / Laotian
41414	Malay / Malayan
41415	Thai / Tai / Siamese
41499	Other Southeast Asian NEC
42100	Chinese NFD
42111	Hong Kong Chinese
42112	Kampuchean Chinese
42113	Malaysian Chinese
42114	Singaporean Chinese
42115	Vietnamese Chinese
42116	Taiwanese Chinese
42199	Chinese NEC
43100	Indian NFD
43111	Bengali
43112	Fijian Indian / Indo-Fijian
43113	Gujarati
43114	Tamil
43115	Punjabi
43116	Sikh
43117	Anglo Indian (addition)
43199	Indian NEC

12925	Greenlander
12926	Hungarian
12927	Icelander
12928	Latvian
12929	Lithuanian
12930	Maltese
12931	Norwegian
12932	Portuguese
12933	Romanian / Rumanian
12934	Romany / Gypsy
12935	Russian
12936	Sardinian
12937	Slavic / Slav
12938	Slovak
12939	Spanish
12940	Swedish
12941	Swiss
12942	Ukrainian
12943	American (US)
12944	Burgher
12945	Canadian
12946	Falkland Islander / Kelper
12947	New Caledonian
12948	South African
12949	Afrikaner (addition)
12950	Zimbabwean (addition)
12999	European NEC
21111	Māori
30000	Pacific peoples NFD
31111	Samoan
32100	Cook Island Maori NFD
32111	Aitutaki Islander
32112	Atiu Islander
32113	Mangaia Islander
32114	Manihiki Islander
32115	Mauke Islander
32116	Mitiaro Islander
32117	Palmerston Islander
32118	Penrhyn Islander
32119	Pukapuka Islander
32120	Rakahanga Islander
32121	Rarotongan
33111	Tongan
34111	Niuean
35111	Tokelauan
36111	Fijian (except Fiji Indian / Indo-Fijian)
37111	Admiralty Islander
37112	Australian Aboriginal
37113	Austral Islander
37114	Belau / Palau Islander
37115	Bismark Archipelagoan
37116	Bougainvillean
37117	Caroline Islander
37118	Easter Islander

44100	Sri Lankan NFD
44111	Sinhalese
44112	Sri Lankan Tamil
44199	Sri Lankan NEC
44211	Japanese
44311	Korean
44411	Afghani
44412	Bangladeshi
44413	Nepalese
44414	Pakistani
44415<br>44416	Tibetan<br>Eurasian (addition)
44499	Other Asian NEC
51100	Middle Eastern NFD
51111	Algerian
51112	Arab
51113	Assyrian
51114	Egyptian
51115	Iranian / Persian
51116	Iraqi
51117	Israeli / Jewish / Hebrew
51118	Jordanian
51119	Kurd
51120	Lebanese
51121	Libyan
51122	Moroccan
51123	Omani
51124	Palestinian
51125	Syrian
51126	Tunisian
51127	Turkish (including Turkish Cypriot)
51128	Yemeni
51199	Middle Eastern NEC
52100	Latin American / Hispanic NFD
52111	Argentinian
52112	Bolivian
52113	Brazilian
52114	Chilean
52115	Colombian
52116	Costa Rican
52117	Creole (Latin America)
52118	Ecuadorian
52119	Guatemalan
52120	Guyanese
52121	Honduran
52122	Malvinian (Spanish-speaking Falkland Islander)
52123	Mexican
52124	Nicaraguan
52125	Panamanian
52126	Paraguayan
52127	Peruvian
52128	Puerto Rican

37119	Gambier Islander
37120	Guadalcanalian
37121	Guam Islander / Chamorro
37122	Hawaiian
37123	Kanaka / Kanak
37124	I-Kiribati / Gilbertese
37125	Malaitian
37126	Manus Islander
37127	Marianas Islander
37128	Marquesas Islander
37129	Marshall Islander
37130	Nauru Islander
37131	New Britain Islander
37132	New Georgian
37133	New Irelander
37134	Ocean Islander / Banaban
37135	Papuan / New Guinean / Irian Jayan

52129	Uruguayan
52130	Venezuelan
52199	Latin American / Hispanic NEC
53100	African NFD
53112	Creole (US)
53113	Jamaican
53114	Kenyan
53115	Nigerian
53116	African American
53117	Ugandan
53118	West Indian / Caribbean
53119	Somali
53120	Eritrean (addition)
53121	Ethiopian (addition)
53122	Ghanian (addition)
53199	Other African NEC (addition)
61111	Central American Indian (addition)
61112	Inuit / Eskimo(addition)
61113	North American Indian (addition)
61114	South American Indian (addition)
61115	Mauritian (addition)
61116	Seychelles Islander (addition)
61117	South African Coloured (addition)
61118	New Zealander (addition)
61199	Other Ethnicity NEC (addition)
94444	Don't Know (addition)
95555	Refused to Answer
96666	Repeated value *not used
97777	Response unidentifiable (now in use)
98888	Response outside scope *not used
99999	Not stated

Reported For	All Events
Description	The above are MOH defined sets. These codes incorporate the changes as of July 2009 (in brackets in table)
Numerator (If Applicable)	n/a
Denominator (If Applicable)	n/a
Expressed As	Categorical

National

3.9 Domicile Code

Definition	Domicile code is a Geographic Information System (GIS) code for the physical location of the person's registered address
Layout	NNNN (Number: 4 characters)
Codeset	See Appendix 9.3 in DHB's Data Definitions Dictionary for all up to date codes
Reported For	All Events
Description	Numeric code assigned to the physical location of the patient's address<br>Rural addresses without a locality (e.g. RD 9 Wanganui) will not result in an accurate domicile code<br>If a domicile code cannot be assigned automatically, data entry clerks have the ability to manually assign a domicile code
Numerator (If Applicable)	n/a
Denominator (If Applicable)	n/a
Expressed As	Categorical

National

3.10 Deprivation Scale Code

Definition	New Zealand Deprivation Scale: 1-10
Layout	NN (Number: 2 characters)
Reported For	All Events
Description	

The NZDep2006 scale of deprivation scores geographical areas in New Zealand from 1 to 10. **Scores apply to areas not the individual**.

The scores are comprised from a weighted sum of 9 variables[20]:

Dimension of deprivation	*Variable description (in order of decreasing weight)*
Income	People aged 18-64 receiving a means tested benefit
Income	People living in equivalised* households with income below an income threshold
Owned home	People not living in own home
Support	People aged <65 living in a single parent family
Employment	People aged 18-64 unemployed
Qualifications	People aged 18-64 without any qualifications
Living space	People living in equivalised* households below a bedroom occupancy threshold
Communication	People with no access to a telephone
Transport	People with no access to a car

This divides New Zealand into tenths of the distribution of the first principal component scores.

1 = least deprived, 10 = most deprived

Scale is **ordinal** not interval.

Deprivation has been defined as a state of observable and demonstrable disadvantage relative to the local community, wider society or nation to which an individual, family or group belongs. Deprivation is not just material deprivation; it can mean social deprivation also (derived from Atlas of Socioeconomic Deprivation in New Zealand[21]).

Deprivation Scores are calculated by Mesh-Block (group of addresses). These are then combined into areas of interest. For our purposes these will be suburbs, identified by Domicile Code. Each Domicile Code will have an associated deprivation score. Deprivation scores are linked to domicile code (suburb) using a population weighted average of the deprivation scores of the constituent Mesh blocks.

This will be provided for each person per event by NZHIS.

Numerator (If Applicable)	n/a
Denominator (If Applicable)	n/a
Expressed As	Ordinal

National

3.11 NZ Residency Status

Definition	Residency Status is the Immigration status of the patient
Layout	Alpha (A – 1 character)
Codeset	Y = Permanent Resident<br>(New Zealand citizen or classified as 'ordinarily resident in New Zealand')<br><br>N = Temporary<br>(not a New Zealand citizen, does not have New Zealand 'ordinarily resident' status)<br><br>U = Unknown
Reported For	All Events
Description	Patients who are of non-resident status or who are visiting NZ are charged for public health care. This may affect decisions made as the timeliness of choosing health care etc. This is captured by DHBs under NHI data.
Numerator (If Applicable)	n/a
Denominator (If Applicable)	n/a
Expressed As	Categorical

National

3.12 Treatment Eligibility

Definition	Eligibility for fully funded care in the New Zealand public hospital system.
Layout	Alpha (A – 1 character)
Codeset	Y = Eligible for Treatment<br><br>N = Not Eligible for Treatment<br><br>U = Unknown
Reported For	All Events
Description	Patients who are of non-resident status or who are visiting NZ are charged for public health care. This is captured by DHB Revenue Offices.
Numerator (If Applicable)	n/a
Denominator (If Applicable)	n/a
Expressed As	Categorical

National

4.0 ED Event Arrival and Discharge Values

4.1 ED Arrival Mode

Definition	Transport Mode by which the Patient arrives in the Emergency Department
Layout	N (Number: 1 Character)
Codeset (If Applicable)	1 Ambulance<br>2 Helicopter<br>3 Self Presentation<br>4 Police<br>5 Other
Reported For	All Events
Description	For journeys involving more than one mode of transport the mode in which the greater distance of the journey was undertaken will be recorded (i.e. Fixed Wing transfer, with airport to ED by Ambulance)
Numerator (If Applicable)	n/a
Denominator (If Applicable)	n/a
Expressed As	Categorical
**National**	*(If your DHB uses a different list for this variable please provide the list you use as a lookup table)*

4.2 Referral Type

Definition	Type of referral to hospital.
Layout	See code set below
Codeset (If Applicable)	Self — Self Referral<br>Clinic — Accident Clinic<br>GP — General Practitioner<br>Hospital — Hospital Transfer<br>OtherHP — Other Health Practitioner<br>Unknown — Referral type not recorded
Reported For	All Presentations
Description	Type of referral made and recorded for admission to hospital for the patient. Categorised as a self referral i.e. walk-in or ambulance (patient transported in directly without consulting a medical professional), referred from a general practitioner, a medical professional, and/or transfer from another hospital.
Numerator (If Applicable)	n/a
Denominator (If Applicable)	n/a
Expressed As	Categorical

**National**	*(If your DHB uses a different list for this variable please provide the list you use as a lookup table)*

4.3 Referral Reason

Definition	Reason for referral, usually recorded in free text.
Layout	(Free text)
Codeset (If Applicable)	Free text.
Reported For	All Presentations
Description	Referral reason as recorded in free text by triage nurse or ward clerk at triage.
Numerator (If Applicable)	n/a
Denominator (If Applicable)	n/a
Expressed As	String

National

4.4 Admission Type

Definition	Admission type for patient as recorded at time of triage.
Layout	AA (2 Character)
Codeset (If Applicable)	AA Acute<br>AC Arranged Admission<br>WN Waiting List
Reported For	All Presentations
Description	Patient can either be admitted to hospital from waiting list, a arrange admission or an acute admission. Most admissions to ED, if not all, are acute admissions. Patients that are admitted directly to a ward can fall under arranged admissions or waiting list.
Numerator (If Applicable)	n/a
Denominator (If Applicable)	n/a
Expressed As	Categorical

National

4.5 ACC Status

Definition	Accident Compensation Corporation status
Layout	A (Letter: 1 character)
Codeset (If Applicable)	Y Yes<br>N No<br>U Unknown
Reported For	All Events
Description	The Accident Compensation Corporation provides personal injury cover to all New Zealanders and visitors to New Zealand. An ACC referral is made by the attending health professional if the patients presentation is related to an "Accident"; in other words an unforeseen incident causing Trauma.
Numerator (If Applicable)	n/a
Denominator (If Applicable)	n/a
Expressed As	Categorical

National

4.6 Triage Category

Definition	Patients' medical urgency category according to Australasian Triage Scale [22]. The patient needs to be seen within the time allocated to the triage category.
Layout	N (Number: 1 Character)
Codeset (If Applicable)	

1	Immediate simultaneous triage and treatment
2	10 minutes
3	30 minutes
4	60 minutes
5	120 minutes

Reported For	All Events
Description	The maximum length of time someone should wait for Health professional assessment and treatment as determined by the reason for coming to hospital.<br><br>**Triage Cat 1**: Immediately life-threatening,<br>**Triage Cat 2**: Imminently life-threatening, or important time-critical treatment, or very Severe Pain.<br>**Triage Cat 3**: Potentially life-threatening condition, potential adverse outcomes from delay > 30 min, or severe discomfort or distress (situational urgency)<br>**Triage Cat 4**: Potentially serious (condition may deteriorate), or potential adverse outcomes from delay > 60 min, or significant complexity or severity of patients condition, or discomfort or distress (situational urgency)<br>**Triage Cat 5**: Less urgent (chronic or minor conditions), or dealing with administrative issues only
Numerator (If Applicable)	n/a
Denominator (If Applicable)	n/a
Expressed As	Categorical

National

4.7 Triage to (Location)

Definition	Triaged in ED to care under the Emergency Department or another Acute Care Ward (ADU / APU) **(i.e. First Ward the patient is seen on or triaged to be seen on at presentation to hospital).**
Layout	N (Number: 1 character)
Codeset (If Applicable)	0 Unknown<br>1 Triage to ED<br>2 Triage to APU / ADU / Similar<br>3 Triage to Other
Reported For	All Events
Description	In New Zealand most patients including self-presenters and those referred to inpatient specialties are triaged in the ED to the ED or APU / ADU. Possibilities following triage include:<br>1. Triaged to care under the Emergency Department (ED) – either as an ED patient or other inpatient service patient.<br>2. Triaged to care under another Acute Care Ward APU / ADU (I.e. Primary Care referral to General Surgery), meaning no time spent in ED.<br>3. Patients that did not go through ED but direct to an Inpatient Ward would be classified as 'Other'
Numerator (If Applicable)	n/a
Denominator (If Applicable)	n/a
Expressed As	Categorical

National

4.8 ED Disposition

Definition	Admitted to Hospital as an Inpatient, discharged or Transferred to another Hospital
Layout	N (Number: 1 character)
Codeset (If Applicable)	

0 = Discharged from ED - by ED Clinician

1 = Discharged from ED - by Inpatient Team Clinician

2 = Admitted to Ward

3 = Admitted to Intensive Care Unit

4 = Admitted to Coronary Care Unit

5 = Transfer to Other Hospital

6 = Not Available

7 = Not Recorded

Reported For	All Events
Description	Assigned by Research team for Study Sites during manual data collection.
Numerator (If Applicable)	n/a
Denominator (If Applicable)	n/a
Expressed As	Categorical

Study Sites

4.9 ED Discharge Type

Definition	Type of discharge from the ED: Event End type (NMDS).	
Layout	AAAAA (Alpha: 5 Characters)	
Codeset (If Applicable)	DIED	Deceased
	ORGAN	Discharge for organ donation
	ACUTE	Discharge to Acute Care Facility
	EDED	ED - Deceased
	EDEA	ED - Discharge to Acute Care Facility
	EDER	ED - Routine Discharge
	EDEI	ED - Self Discharge, Indemnity Signed
	EDES	ED - Self Discharge, No Indemnity
	EDET	ED - Transfer to Non-Acute Care Facility
	DNW	Patient did not wait
	RD	Routine discharge
	SDI	Self Discharge, Indemnity signed
	SD	Self Discharge, No Indemnity
	FUND	Stat'l dsch for change in funder
	ITRAN	Stat'l dsch for transfer b/n specified care types
	XFER	Transfer to Non-Acute Care Facility
Reported For	Includes: All Events	
Description	Used to identify the **immediate** departure status of the patient upon leaving the ED. This flags patients who did not wait for treatment and those who have self-discharged. The discharge destination may not always be to the patients' usual place of residence.	
Numerator (If Applicable)	n/a	
Denominator (If Applicable)	n/a	
Expressed As	Categorical	

**National**	*(Please provide lookup table to explain the Discharge Types). Blank field for patients that are transferred to ward instead of discharge from ED*

4.10 ED Discharge Destination Type

Definition	Site or place of discharge in the community following ED treatment.
Layout	N (Number: 1 Character)
Codeset (If Applicable)	1 Healthcare Facility - Private<br>2 Healthcare Facility - Public<br>3 Home<br>4 Other<br>5 Overseas<br>6 Prison<br>7 Rest Home
Reported For	All Events
Description	Includes information on the immediate discharge destination of the patient on departure from the ED. The list included here are in the summarized format for the reports to the Ministry of Health.<br><br>A private healthcare provider is one who is paid by the patient for care.<br><br>A public healthcare provider is one who provides treatment which is fully funded by the state.
Numerator (If Applicable)	n/a
Denominator (If Applicable)	n/a
Expressed As	Categorical
**National**	*(Please provide lookup table to explain the Discharge Destination Types). Blank field for patients that are transferred to ward instead of discharge from ED*

4.11 Ward Discharge Type

Definition	Type or Status of discharge following departure from the ward: Event End type (NMDS)
Layout	AAAAA (Alpha: 5 Characters)
Codeset (NMDA = MOH)	

DA	Discharge to acute specialist facility
DC	Psychiatric patient discharged to community care
DD	Died
DF	Change of funder
DI	Self discharge from hospital, indemnity signed
DL	Committed psychiatric patient discharged to leave of more than 14 days
DN	Psychiatric remand patient discharged without committal
DO	Discharge of a patient kept sustainable for organ donation
DP	Psychiatric patient transferred for further psychiatric care
DR	Ended routinely
DS	Self discharge from hospital (no indemnity signed)
DT	Discharge of patient to another healthcare facility
DW	Discharge to other service within same facility

Reported For	Includes: All Admissions
Description	Above are MOH NMDA codes for Event End Type. The DHB's internal system may catch many other codes, but there may not be inter-hospital validity with this method. Used to identify the **immediate** status of the patient upon departure from the ward. The discharge destination may not always be to the patients' usual place of residence, or the same residence they were admitted from.
Numerator (If Applicable)	n/a
Denominator (If Applicable)	n/a
Expressed As	Categorical

National *(Please provide lookup table to explain the Discharge Types). Blank field for patients that were not admitted to ward and discharged from ED*

4.12 Ward Discharge Destination Type

Definition	Site or place of discharge in the community following Ward Admission
Layout	N (Number: 1 Character)
Codeset (If Applicable)	1 Healthcare Facility - Private<br>2 Healthcare Facility - Public<br>3 Home<br>4 Other<br>5 Overseas<br>6 Prison<br>7 Rest Home
Reported For	Includes: All Admissions
Description	Includes information on the immediate discharge destination of the patient on departure from the ward. The list included here are in the summarized format for the reports to the Ministry of Health<br><br>A private healthcare provider is one who is paid by the patient for care.<br><br>A public healthcare provider is one who provides treatment which is fully funded by the state.
Numerator (If Applicable)	n/a
Denominator (If Applicable)	n/a
Expressed As	Categorical
**National**	*(Please provide lookup table to explain the Discharge Destination Types). Blank field for patients that were not admitted to ward and discharge from ED.*

4.13 Discharge Ward Type

Definition	Ward or location in which patient was last admitted to or seen at and finally discharged from, to home, another DHB hospital, a long term residential care location or deceased.<br>Either discharged from Emergency Department or Acute Care Ward APU / ADU or Inpatient Specialty.
Layout	N (Number: 1 character)
Codeset (If Applicable)	0 Unknown<br>1 ED, ED SSU<br>2 SSU (Short Stay Unit) e.g. APU, ADU<br>3 Inpatient Ward<br><br>**I.e. Last ward location the patient was at and discharged from. (At time of discharge from hospital).**
Reported For	All Discharges
Description	Patients presenting to ED are either admitted to an ED Short Stay Unit, an Inpatient Specialty Short Stay Unit (APU / ADU), an Inpatient Ward or are discharged following review. Patients admitted to ED SSU are reviewed further and finally discharged. Patients admitted into Inpatient specialty care (either APU/ADU or Ward) are reviewed, possibly spend some time in hospital and finally discharged.
Numerator (If Applicable)	n/a
Denominator (If Applicable)	n/a
Expressed As	Categorical

National

5.0 Event Time Stamp Values

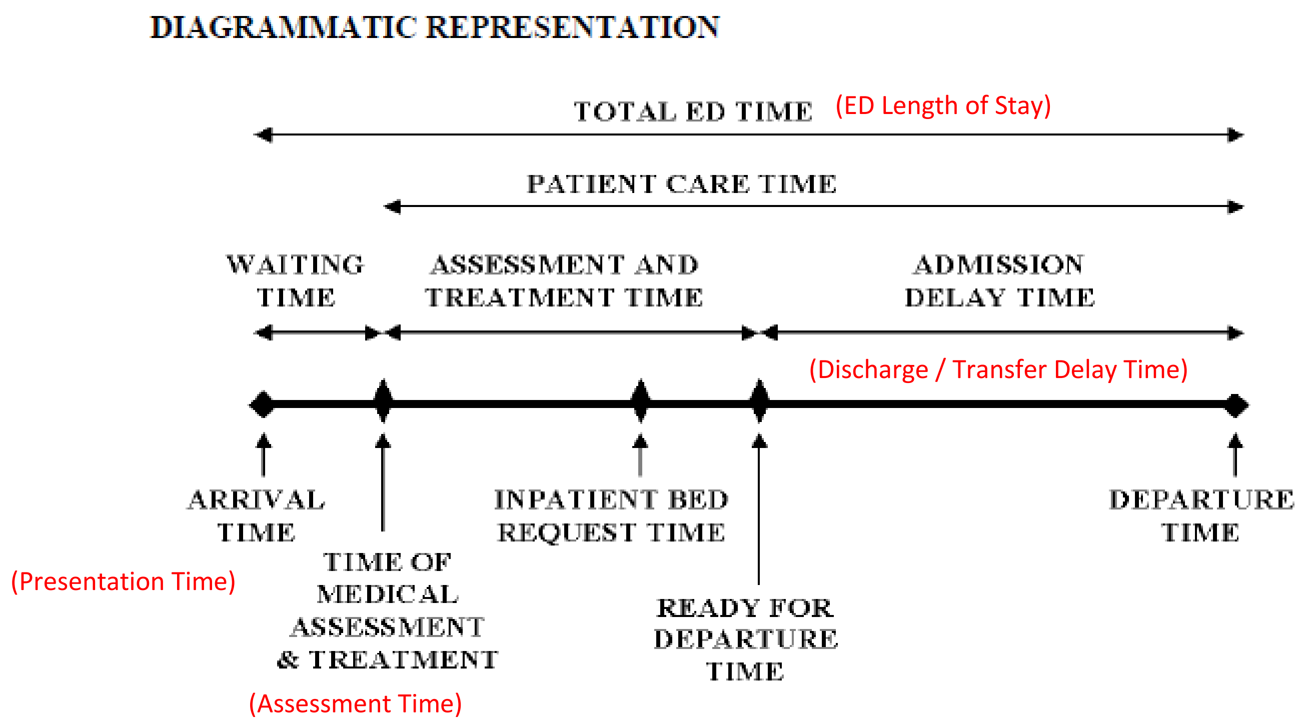

Figure 1. **ACEM Time Stamp Graphic**[11]

5.1 Presentation Time

Definition	The Time (and Date) the patient arrives at the Emergency Department during the time period (study period) of interest.
Layout	DD/MM/CCYY HH:MM:SS (Time: 18 characters with a space between date and time values)
Codeset (If Applicable)	n/a
Reported For	All Events
Description	Is ‘**Event Start Date’** (NMDS)<br>The time of initial contact between the patient and the triage nurse or clerical staff, whoever they see first.<br>A recording accuracy to within the nearest minute is appropriate. There should be no delay between the physical arrival in the ED of a patient who is seeking care and their first contact with staff[11].<br>Midnight is 00:00:00
Numerator (If Applicable)	n/a
Denominator (If Applicable)	n/a
Expressed As	Numeric (Time)

National

5.2 Triage Time

Definition	The time (and date) the patient is assessed by the Medical Triage Staff (either nurse or doctor).
Layout	DD/MM/CCYY HH:MM:SS (Time: 18 characters with a space between date and time values)
Codeset (If Applicable)	n/a
Reported For	All Events
Description	The point in time that the patient is triaged by a health professional trained in such, into an urgency category as per the Australasian Triage scale. If triage occurs on arrival, Triage Time will equal Presentation Time
Numerator (If Applicable)	n/a
Denominator (If Applicable)	n/a
Expressed As	Numeric (Time)

National

5.3 Assessment Time

Definition	Time first attended to by an ED Physician
Layout	DD/MM/CCYY HH:MM:SS (Time: 18 characters with a space between date and time values)
Codeset (If Applicable)	n/a
Reported For	All Events<br>Exclude: Did Not Waits (DNW), Dead on Arrival (DOA), Triage Nurse assessment
Description	Time first attended to by an Emergency Department Health Professional or Clinical Decision Maker (doctor, nurse specialist / nurse practitioner or nurse using clinical pathway). Also known as "sign-on time", this can be electronically captured when a Health Professional takes responsibility for the patient, or documents 'assessment time' in the clinical notes.
Numerator (If Applicable)	n/a
Denominator (If Applicable)	n/a
Expressed As	Numeric (Time)

National

5.4 Referral Time

Definition	The Time a request is made to an Inpatient Specialties service for review or admission of a Patient
Layout	DD/MM/CCYY HH:MM:SS (Time: 18 characters with a space between date and time values)
Codeset (If Applicable)	n/a
Reported For	Includes: All Events referred for Inpatient Care
Description	When a patient needs to be admitted to hospital the Emergency Department Practitioner must refer this patient to the relevant inpatient specialty. The time the transfer of care happens should be either captured electronically (by change of CBU on the computer system – i.e. electronic referral) or documented by the physician in the notes.
Numerator (If Applicable)	n/a
Denominator (If Applicable)	n/a
Expressed As	Numeric (Time)

**National**	**Referral Time is not something that is routinely reported on.**

5.5 Inpatient Team Time

Definition	The time a patient is seen by an Inpatient Registrar or other team Member.
Layout	DD/MM/CCYY HH:MM:SS (Time: 18 characters with a space between date and time values)
Codeset (If Applicable)	n/a
Reported For	Includes: All events referred for Inpatient Care<br>Excludes: Transfers to other hospitals
Description	Can be captured electronically with Inpatient Physician "sign-on" time or on the clinical notes as documented by the Physician
Numerator (If Applicable)	n/a
Denominator (If Applicable)	n/a
Expressed As	Numeric (Time)

**National**	**Inpatient team Time is not something that is routinely reported on.**

5.6 Time of Bed Request

Definition	Time an Inpatient Bed is booked for the patient
Layout	DD/MM/CCYY HH:MM:SS (Time: 18 characters with a space between date and time values)
Codeset (If Applicable)	n/a
Reported For	Includes: All Events where the patient is admitted
Description	ACEM: This represents the time when a formal request is made to obtain an inpatient bed for a patient requiring admission to hospital. This time is significantly more subjective than arrival time or departure time[11]<br><br>Bed Request usually follows completion of inpatient specialty review of the patient. Can be captured electronically where a bed is requested over a PIMS or Bed Management system or via a Bed Manager and documented by hand.
Numerator (If Applicable)	n/a
Denominator (If Applicable)	n/a
Expressed As	Numeric (Time)
**National**	**Time of bed request is not something that is routinely reported on.**

5.7 Time of Bed Allocation

Definition	Time when requested inpatient bed is allocated to a particular patient
Layout	DD/MM/CCYY HH:MM:SS (Time: 18 characters with a space between date and time values)
Codeset (If Applicable)	n/a
Reported For	Includes: All Events where the patient is admitted
Description	Beds are allocated as is appropriate for the patients' need to be in hospital (i.e.: Surgical Ward for a surgical problem). Therefore it may be easier to allocate certain patients beds as compared to others.<br><br>A 'Home Ward' is the ward that provides the specialised medical and nursing care required by the patient.<br><br>Outlier: Those patients allocated a bed in a ward whose specialty alignment is not that of the patient's 'Home Ward'.<br><br>Bed Allocation data is captured electronically or manually by the Clerical / Bed manager Staff when an appropriate inpatient bed has been found
Numerator (If Applicable)	n/a
Denominator (If Applicable)	n/a
Expressed As	Numeric (Time)
**National**	**Time of bed allocation is not something that is routinely reported on.**

5.8 ED Departure Time

Definition	The time when the patient physically leaves the department
Layout	DD/MM/CCYY HH:MM:SS (Time: 18 characters with a space between date and time values)
Codeset (If Applicable)	n/a
Reported For	All Events
Description	ED Visit End Time: the time at which the patient physically leaves the ED: either admitted to an Inpatient ward, transferred to another hospital facility or discharged to home.<br>Captured electronically<br>○ Admission: is the time at which the patient is physically moved from the ED to an inpatient ward, or the time at which a patient begins a period of formal observation, whether in ED SSU observation beds (see below), an observation unit, or similar. Inpatient wards include Inpatient short stay units<br>○ ED SSU Admission: allows a period of formal observation under the care of Emergency medicine, not inpatient specialties<br>○ Transfer: is the time a patient physically leaves the ED after being assessed as needing treatment at another health facility, or they are resident in area serviced by another DHB and are therefore transferred to their 'Home Hospital'.<br>○ Discharge: is the time at which a patient being discharged from the ED to the community physically leaves the ED. If treatment is finished and patient is waiting in the ED facility for transport they can be treated as discharged[23].<br>○ Death: transfer to mortuary
Numerator (If Applicable)	n/a
Denominator (If Applicable)	n/a
Expressed As	Numeric (Time)

National

5.9 ED SSU Admit / Assign Time

Definition	The time a patient is admitted to the ED Short Stay Unit
Layout	DD/MM/CCYY HH:MM:SS (Time: 18 characters with a space between date and time values)
Codeset (If Applicable)	n/a
Reported For	Includes: All ED patients admitted to the ED SSU
Description	Electronic Capture (SSU “Flag” or time of arrival in SSU) or Documentation in the clinical notes.<br>Clinicians may admit a patient to an ED SSU if it is felt that they need a further short period of time for observation, or they need treatment that is usually less than 12 hours duration.<br>This ‘stops the clock’ for the ED LOS – however these patients are still under the care of the ED team – the total LOS of the patient including their ED SSU LOS can be captured by adding ED LOS to SSU LOS (ED Discharge Time – ED SSU Admit / Assign Time).
Numerator (If Applicable)	n/a
Denominator (If Applicable)	n/a
Expressed As	Numeric (Time)

National

5.10 Ward Admission Time

Definition	The time a patient is admitted to an Inpatient Ward
Layout	DD/MM/CCYY HH:MM:SS (Time: 18 characters with a space between date and time values)
Codeset (If Applicable)	n/a
Reported For	Includes: All ED patients admitted to an Inpatient Ward
Description	Electronic Capture<br>Clinicians may admit a patient to a ward if it is felt that they need ongoing treatment or investigation in the Hospital setting. Patients may be admitted to the ward from the emergency department, from another ward (for example ICU), from another hospital or from the community. We want to capture new ward admissions from outside the hospital (i.e. ED, other Hospital or Community) not ward to ward transfers.<br><br>The time may be equivalent to the ED discharge time.
Numerator (If Applicable)	n/a
Denominator (If Applicable)	n/a
Expressed As	Numeric (Time)

National

5.11 Ward Departure Time

Definition	The time at which the patient physically leaves an Inpatient Ward
Layout	DD/MM/CCYY HH:MM:SS (Time: 18 characters with a space between date and time values)
Codeset (If Applicable)	n/a
Reported For	Includes: All Ward Discharges<br>Excludes: Those discharged on Ward Leave, or returning after a procedure at another facility.
Description	Ward Visit end time: the time at which the patient physically leaves the Ward: either Discharged to home or they can be transferred to another hospital facility.
Numerator (If Applicable)	n/a
Denominator (If Applicable)	n/a
Expressed As	Numeric (Time)

National

5.12 Ward Leave Start Time

Definition	The time at which the patient is assigned ward leave from an Inpatient ward.
Layout	DD/MM/CCYY HH:MM:SS (Date and Time: 18 characters separated by a space between year and hour values)
Codeset (If Applicable)	n/a
Reported For	Includes: Inpatient or Day Patient leaving the ward to attend:<br>• Home under prior arrangement with the healthcare team.<br>• An outpatient/ambulatory clinic, *whether at the same or a different facility* (Same DHB), and placed on **ward leave** for the duration of their absence.<br>• An inpatient location at a *different facility* (different DHB) and put on **ward leave** for the duration of their absence.<br>Excludes: n/a
Description	The start time on planned absence of an inpatient from the healthcare facility to which they were most recently admitted. Leave is counted only where that patient is absent at midnight and has a planned return within three nights of going on leave, for the continuation of their treatment or care.
Numerator (If Applicable)	n/a
Denominator (If Applicable)	n/a
Expressed As	Numeric (Time)

National

5.13 Ward Leave End Time

Definition	The time at which the patient returns from ward leave back to an inpatient facility and is assigned back on the ward.
Layout	DD/MM/CCYY HH:MM:SS (Date and Time: 18 characters separated by a space between year and hour values)
Codeset (If Applicable)	n/a
Reported For	Includes: Patients that had been on ward leave and returning back for continuation of their care.<br><br>Excludes: n/a
Description	The return time on planned absence of an inpatient from the healthcare facility to which they were most recently admitted. Leave is counted only where that patient is absent at midnight and has a planned return within three nights of going on leave, for the continuation of their treatment or care.
Numerator (If Applicable)	n/a
Denominator (If Applicable)	n/a
Expressed As	Numeric (Time)

National

6.0 Emergency Department General Data

6.1 ED FTE: Medical

Definition	Number of Full Time Equivalent Medical Staff per each DHB Emergency Department over study time period (yearly blocks 1st July 2006 – 30th June 2011).
Layout	NNNNNN (Number: 6 Characters)
Codeset (If Applicable)	n/a
Reported For	Includes: All ED Medical Staff, Inclusive of Physician Assistant.<br><br>Excludes:
Description	An FTE of 1.0 means that the person is equivalent to a full-time worker; while an FTE of 0.5 signals that the worker is only half-time.
Numerator (If Applicable)	n/a
Denominator (If Applicable)	n/a
Expressed As	Number

National

6.2 ED FTE: Nursing

Definition	Number of Full Time Equivalent Nursing Staff per each DHB Emergency Department over study time period (yearly blocks 1st July 2006 – 30th June 2011).
Layout	NNNNNN (Number: 6 Characters)
Codeset (If Applicable)	n/a
Reported For	Includes: All ED Nursing Staff<br>Excludes:
Description	An FTE of 1.0 means that the person is equivalent to a full-time worker; while an FTE of 0.5 signals that the worker is only half-time.
Numerator (If Applicable)	n/a
Denominator (If Applicable)	n/a
Expressed As	Number

National

6.3 ED FTE: HCA (Hospital Aid or similar)

Definition	Number of Full Time Equivalent Health Care Assistants (HCA) or Hospital Aid per each DHB Emergency Department over study time period (yearly blocks 1st July 2006 – 30th June 2011).
Layout	NNNNNN (Number: 6 Characters)
Codeset (If Applicable)	n/a
Reported For	Includes: All ED Healthcare Assistants<br>Excludes:
Description	HCA or Hospital Aid or similar staffs that are to aid and assist the nursing team.<br>An FTE of 1.0 means that the person is equivalent to a full-time worker; while an FTE of 0.5 signals that the worker is only half-time.
Numerator (If Applicable)	n/a
Denominator (If Applicable)	n/a
Expressed As	Number

National

6.4 ED FTE: Orderlies

Definition	Number of Full Time Equivalent Orderly Staff per each DHB Emergency Department over study time period (yearly blocks 1st July 2006 – 30th June 2011).
Layout	NNNNNN (Number: 6 Characters)
Codeset (If Applicable)	n/a
Reported For	Includes: All ED Orderly Staff<br>Excludes:
Description	An FTE of 1.0 means that the person is equivalent to a full-time worker; while an FTE of 0.5 signals that the worker is only half-time.
Numerator (If Applicable)	n/a
Denominator (If Applicable)	n/a
Expressed As	Number

National

6.5 ED FTE: Ward Clerk

Definition	Number of Full Time Equivalent ED Ward Clerks or Clerical staff per each DHB Emergency Department over study time period (yearly blocks 1st July 2006 – 30th June 2011).
Layout	NNNNNN (Number: 6 Characters)
Codeset (If Applicable)	n/a
Reported For	Includes: All ED Ward Clerk Staff inclusive of Ward Clerk Team Lead or Manager.<br>Excludes:
Description	An FTE of 1.0 means that the person is equivalent to a full-time worker; while an FTE of 0.5 signals that the worker is only half-time.
Numerator (If Applicable)	n/a
Denominator (If Applicable)	n/a
Expressed As	Number

National

6.6 ED FTE: Managers

Definition	Number of Full Time Equivalent ED Managers per each DHB, over study time period (yearly blocks 1st July 2006 – 30th June 2011).
Layout	NNNNNN (Number: 6 Characters)
Codeset (If Applicable)	n/a
Reported For	Includes: All ED Management Staff.<br>Excludes:
Description	An FTE of 1.0 means that the person is equivalent to a full-time worker; while an FTE of 0.5 signals that the worker is only half-time.
Numerator (If Applicable)	n/a
Denominator (If Applicable)	n/a
Expressed As	Number

National

6.7 Bed Occupancy (at Midnight) - Hospital (%)

Definition	Daily bed occupancy rate as calculated by the DHB, for the whole hospital over the study time period (1st July 2006 – 30th June 2011).
Layout	NNN (Number: 3 Characters)
Codeset (If Applicable)	n/a
Reported For	Includes: Inpatient Wards<br>Excludes: APU, SSU. ADU, ED Wards, Day Stay wards
Description	Bed occupancy as recorded at midnight by the Bed Manager for the entire hospital expressed in percentage. This is as reported by the DHB, not as a derived value. It will not be possible to gather all the information necessary to independently calculate daily hospital occupancy for all sites.
Numerator (If Applicable)	n/a
Denominator (If Applicable)	n/a
Expressed As	Numeric (Percentage)

National

6.8 Bed Occupancy (at Midnight) – APU / ADU (%)

Definition	Daily bed occupancy rate as calculated by the DHB, for Hospital Short Stay Unit over the study time period (1st July 2006 – 30th June 2011).
Layout	NNN (Number: 3 Characters)
Codeset (If Applicable)	n/a
Reported For	Includes: ADU, APU or Short Stay Unit<br>Excludes: ED, Inpatient Wards, Day Stay Wards
Description	Bed occupancy as recorded at midnight for the Hospital Short Stay Unit (ADU, APU) expressed in percentage. This is the occupancy as provided by the DHB. It will not be possible to gather all the information necessary to independently calculate daily hospital occupancy for all sites.
Numerator (If Applicable)	n/a
Denominator (If Applicable)	n/a
Expressed As	Numeric (Percentage)

National

7.0 Outcomes

These include the main outcomes of the study for all 21 DHB's (including the study sites). These measures have been included in the Raw Data Dictionary for an overall picture of the outcome measures.

7.1 Primary Outcomes

7.1.1 Length of Stay ED (ED LOS – DHB Value)

Definition	Interval between Presentation Time and ED Departure Time as reported by the DHB.
Layout	MMMM (Time in Minutes: 4 characters)
Codeset (If Applicable)	n/a
Reported For	Includes: All Events (Include ED SSU patients)<br>Excludes:
Description	*ED Departure Time – Presentation Time*<br>Length of stay for all patients presenting to the ED during time period X (midnight to midnight) who are subsequently admitted to the Hospital, transferred or discharged from the Emergency Department.<br>Each Study Site will be able to provide data for ED LOS (as per ministry guidelines). We will also gather data for each hospital on the background times based on our definitions to ensure uniformity of Data between sites. This definition will be in the derived data manual for the SSED NRP.
Numerator (If Applicable)	n/a
Denominator (If Applicable)	n/a
Expressed As	Numeric
Subset	Broken Down into Number of Patients with ED LOS <6 hours Subset, > 6 hours Subset
**National**	*Calculated field as stored in the Patient Information Management (PMS) system by each DHB for all presentations/ admission to ED*

7.1.2 Total Length of Stay (TLOS - DHB)

Definition	Interval between ED Presentation Time and Ward Departure Time as reported by the DHB.
Layout	MMMM (Time in Minutes: 4 characters)
Codeset (If Applicable)	n/a
Reported For	Includes: All Events<br>Excludes:
Description	*Ward Departure Time – ED presentation Time*<br>Total length of stay for all individual patients in Hospital (which includes ED LOS). An individual patients' length of stay is the total time they have spent in hospital as a patient.<br>Each Study Site will be able to provide data for LOS (as per ministry guidelines). We will also gather the data for each hospital on the background times based on our definitions to ensure uniformity. This definition will be in the derived data manual for the SSED NRP.
Numerator (If Applicable)	n/a
Denominator (If Applicable)	n/a
Expressed As	Numeric
Subset	Broken down into Total LOS < 24 hours Subset and > 24 hours Subset
**National**	*Calculated field as stored in the Patient Information Management (PMS) system by each DHB on patient total length of stay in hospital*

7.1.3 ED Re-Attendance < 48 Hours (Derived)

Definition	Number of people over study time period (monthly blocks 1st July 2006 – 30th June 2011), who have returned to the ED within 48 hours of discharge, with the same medical problem.
Layout	NNNNNN (Number: 6 Characters)
Codeset (If Applicable)	For National Sites<br>0 = No<br>1 = Yes<br><br>For Case Study Sites:<br>0 = No Re-attendance<br>1 = Advised Re-attendance<br>2 = Planned Re-attendance<br>3 = Unplanned Re-attendance
Reported For	Includes: All ED Presentations (Includes DNW's)<br>Excludes:
Description	Re-Attendees are those people returning to the ED with issues relating to a medical condition they were assessed for in the preceding 48 hours. These can either be advised, planned or unplanned re-attendances. These are usually flagged electronically by Triage staff in the Triage Diagnosis column – but not always.<br>**Advised Re-Attendance** – advice on discharge from ED is to come back to the ED, should the medical condition worsen.<br>**Planned Re-Attendance** – Patients are told to come back at a certain time for further assessment in the ED for their medical condition. On discharge some are flagged electronically as a planned ED Re-Attendance within a certain period of time.<br>**Unplanned Re-Attendance** – Patients come back to the ED with the same medical condition they were discharged with previously. The concern here is risk of higher mortality and the issue of possible initial low quality care, or poor discharge methods.<br><br>Data collected via NZHIS – searching for NHI with presentation to any acute care facility in NZ within 48 hours of Indicator (primary) event.
Numerator (If Applicable)	n/a
Denominator (If Applicable)	n/a
Expressed As	Categorical
**National**	**Not all DHBs flag re-attendances, we will be able to flag with data set. Potential for sub-study at case study sites.**

7.1.4 ED Re-Attendance < 72 Hours (Derived)

Definition	Number of people over study time period (monthly blocks 1st July 2006 – 30th June 2011), who have returned to the ED within 72 hours of discharge, with the same medical problem.
Layout	NNNNNN (Number: 6 Characters)
Codeset (If Applicable)	For National Sites<br>0 = No<br>1 = Yes<br><br>For Case Study Sites:<br>0 = No Re-attendance<br>1 = Advised Re-attendance<br>2 = Planned Re-attendance<br>3 = Unplanned Re-attendance
Reported For	Includes: All ED Presentations (Includes DNW's)<br>Excludes:
Description	Re-Attendees coming back to the ED with issues relating to a medical condition they were assessed for in the preceding 72 hours. These can either be advised, planned or unplanned re-attendances. These are usually flagged electronically by Triage staff in the Triage Diagnosis column – but not always.<br>**Advised Re-Attendance** – advice on discharge is to come back to the ED, should the medical condition worsen.<br>**Planned Re-Attendance** – Patients are told to come back at a certain time for further assessment in the ED for their medical condition. On discharge some are flagged electronically as a planned ED Re-Attendance within a certain period of time.<br>**Unplanned Re-Attendance** – Patients come back to the ED with the same medical condition they were discharged with previously. The concern here is risk of higher mortality and the issue of possible initial low quality care, or poor discharge methods.<br><br>Data collected via NZHIS – searching for NHI with presentation to any acute care facility in NZ within 48 hours of Indicator (primary) event.
Numerator (If Applicable)	n/a
Denominator (If Applicable)	n/a
Expressed As	Categorical
**National**	NOTE: May be scope to look at specific types of DNW at case study sites and ACH.

7.1.5 Re-Attendance Inpatients < 72 hours (Derived)

Definition	Number of people over study time period (monthly blocks 1st July 2006 – 30th June 2011), who have returned to the Hospital within 72 hours of discharge from an inpatient ward, with the same medical problem.
Layout	NNNNNN (Number: 6 Characters)
Codeset (If Applicable)	For National Sites<br>0 = No<br>1 = Yes<br><br>For Case Study Sites:<br>0 = No Re-attendance<br>1 = Advised Re-attendance<br>2 = Planned Re-attendance<br>3 = Unplanned Re-attendance
Reported For	Includes: All Hospital Inpatient Discharges<br>Excludes:
Description	Re-Attendees coming back to the hospital (Or ED) with issues relating to a medical condition they were assessed for in the preceding 72 hours.<br><br>Discharges for the month in question where:<br>2nd Admission:<br>• Admission Type is Acute<br>• Visit Type is Inpatient, Day Patient (Intended), Short Stay or Newborn<br><br>1st Admission:<br>• Is <72 hours prior to 2nd admission<br>• Has the same Last CBU as the 2nd admission<br>• Is not a transfer to another facility<br><br>Neither Admission:<br>• Has Last CBU of Maternity*, A+ Links*, Buchanan Rehabilitation Centre, Fraser McDonald Unit, Emergency Medicine, Child Emergency Dept (CED), Rehab (ADHB)<br><br>Re-admission may mean discharge was too early, before patient had finished an adequate course of treatment etc.
Numerator (If Applicable)	n/a
Denominator (If Applicable)	n/a
Expressed As	Categorical

**National**	**NOTE: May be scope to look at specific types of DNW at case study sites and ACH.**

7.1.6 Access Block (Derived)

Definition	Percentage of patients in ED requiring hospital admission spending >8 hours waiting in the ED for an Inpatient Bed, over the study time period (monthly blocks between 1st July 2006 – 30th June 2011).
Layout	NNN (Number: 3 characters)
Codeset (If Applicable)	n/a
Reported For	Includes: ED to Ward Admissions Total<br>Excludes:
Description	*(Number of Admitted Patients > 8 Hours ED LOS / ED to Ward Admissions Total) x 100*<br>The percentage of the ED to Ward admissions total whose ED LOS was greater than 8 hours over the study time period.
Numerator (If Applicable)	Number of patients who were admitted or planned for admission whose total ED time exceeded 8 hours
Denominator (If Applicable)	Number of patients who were admitted or planned for admission
Expressed As	Numeric (Percentage)

National

7.2 Secondary Outcomes:

7.2.1 Mortality

Definition	Number of Deaths in patients presenting to Emergency Departments within the study time period (monthly blocks 1st July 2006 – 30th June 2011).
Layout	NNNNNN (Number: 6 Characters)
Codeset (If Applicable)	Discharge Type:<br>DIED Deceased
Reported For	Includes: All ED Presentations<br>Excludes:
Description	Number of deaths in ED attendees at event, 10 days, 30 days and 90 days following treatment in an ED whether discharged, admitted or transferred.<br>Check Standardized mortality against NHI's, or check NHI's against death register.
Numerator (If Applicable)	n/a
Denominator (If Applicable)	n/a
Expressed As	Numeric

National

7.2.2 Hospital Mortality

Definition	Number of Inpatients Deaths over study time period (monthly blocks 1st July 2006 – 30th June 2011).
Layout	NNNNNN (Number: 6 Characters)
Codeset (If Applicable)	Discharge Type:<br>DIED Deceased
Reported For	Includes: All Hospital Inpatient Admissions<br>Excludes:
Description	Number of deaths in Hospital Inpatients at any time during their Hospitalisation per event.
Numerator (If Applicable)	n/a
Denominator (If Applicable)	n/a
Expressed As	Numeric

National

7.2.3 ED "Did Not Wait" (DNW)

Definition	Number of attendances where a patient registered at triage, but left without being seen by an ED Health Professional over study time period (yearly blocks 1st July 2006 – 30th June 2011).
Layout	NNNNNN (number: 6 Characters)
Codeset (If Applicable)	ED Discharge Type: DNW<br>0 = No<br>1 = Yes<br><br>Case Study Sites:<br>0 = Waited and was seen<br>1 = Left before Registration<br>2 = Left before Triage<br>3 = Left before treating clinician
Reported For	Includes: All ED Presentations<br>Excludes:
Description	Also called "**Renege Rate**" (from Queuing Theory) and "**Left Without Being Seen**" (LWBS).<br>The patient does not wait to be seen by any Emergency Department Health Professional (i.e. Doctor, Nurse Practitioner, Nurse Specialist or a Nurse Initiated Pathway). The patient arrives in the department and leaves 1) before registration, 2) after registration and before seeing triage nurse, 3) after registration and seeing triage nurse but never, at any of these times, seeing a treating health professional.<br><br>ACEM Def[24]: the number and percentage of ED patients who, for various reasons, decide to leave the ED before they are seen by an Emergency Medicine Physician<br><br>People who do not wait for assessment and treatment are a concern due to the perceived morbidity and mortality (serious adverse events) from a non-assessed medical condition. However recent studies have challenged this theory[25].<br><br>Captured on ED End Visit
Numerator (If Applicable)	n/a
Denominator (If Applicable)	n/a
Expressed As	Categorical
**National**	NOTE: May be scope to look at specific types of DNW at case study sites and ACH.

8.0 Data Recording (Data Collection Forms)

In the data collection forms for hand extracted data from clinical notes, there are choices from drop-down boxes (which correspond to categorical labels for variables in the data dictionary). This is to keep raw data collection consistent, so it can be compiled easily.

There are four common, recurring choices – how these are to be interpreted is as follows for each of the parts of the consultation:

- **No** – The absence of something
  - CONFOUNDERS from Past medical History and Clinical Notes:
    - If mention of any past medical history made and particular confounder not mentioned = NO (for the reason that it would likely have been documented if it was there)
    - Above and no objective evidence on investigations of presence of confounder = NO
    - 'Nil' or 'Nil PMHx' or 'No Past Medical History' written (or such like) = NO
    - Giving clinician benefit of the doubt
  - SYMPTOMS / CLNICAL HISTORY
    - 'Nil' or 'No' or 'Ø documented in reference to symptom or clinical history recording in notes = NO
  - OBSERVATIONS
    - Will usually be recorded as a number so not applicable – if not there will be a blank box on data collection from usually, unless you want to make it clearer why it wasn't present if there is a reason (see last bullet point in this section)
    - Only will be recorded as a 'No' if it is written in the notes 'Nil', 'No', 'Ǿ' blood pressure taken.
    - Observations generally will not be recorded as a no – they are either present, or they have been done and not recorded, or they haven't been done and not recorded – it will be impossible to tell the difference retrospectively.
    - If no recording documented it is a 'not recorded' (see below) unless it is specifically mentioned 'unrecordable observation' in which case it will be classed as 'unrecordable', or 'No Observation taken' in which case this is a 'no'.
  - CLINICAL FINDINGS / EVENTS
    - If a finding or event related to clinical presentation is not documented in notes = NO (for the reason that it would likely have been documented if it was there). For example respiratory arrest – should have been documented if it happened, therefore if not documented it didn't happen = 'No'.
    - 'Nil' or 'No' or 'Ø documented in reference to clinical findings in notes = NO
  - MEDICATIONS
    - "No analgesia given at home" = NO
    - "No antibiotics given due to inpatient team request" = NO
    - No intention to give a treatment and not recorded in Medication Sheet = NO
    - Can assume no if a particular medication (i.e. Warfarin) not documented on medication list

        - ‘Nil’ or ‘No’ or ‘Ø documented in reference to current medication history recording in notes = NO
    - “Nil” in reference to anything being recorded as variable
    - Circle with a strike through (Ø) in reference to anything being recorded as a variable

- **Yes** – clearly documented evidence in the clinical notes of the presence of something:
    - CONFOUNDERS from Past medical History and Clinical Notes:
        - If mention of any past medical history made and particular confounder mentioned = YES
        - “Co-morbidities include Diabetes Mellitus”
        - Clear objective evidence of confounders on investigations, even if presence of confounder not documented in notes, or documented as no confounder. For example documented “No history of COPD” however has CXR and spirometry findings consistent with COPD, then should be documented has a ‘Yes’ for COPD.
    - SYMPTOMS / CLNICAL HISTORY
        - ‘Yes’ or ‘Present’ or ‘Positive’ documented in reference to symptom or clinical history recording in notes = YES
    - OBSERVATIONS
        - If recorded, usually documented in data collection sheet as the number recorded in the notes
    - CLINICAL FINDINGS / EVENTS
        - If a finding or event related to clinical presentation is documented in notes = YES
        - ‘Yes’ or ‘Present’ or ‘Positive’ documented in reference to clinical findings in notes = YES
    - MEDICATIONS
        - “Given morphine at the GP surgery prior to hospital”
        - Charted evidence of medication on the medication chart **and** signed off by staff who gave them.
        - Can assume yes if a particular medication (i.e. Warfarin) documented on medication list
        - ‘Documented in current medication history recording in notes = YES

- **Not Recorded** – nothing written or recorded in the notes
    - CONFOUNDERS from Past medical History and Clinical Notes:
        - If no mention of any past medical history made at all in the notes, or in previous notes to the event and no investigations to the positive, or negative for the confounder = NOT RECORDED. For example, if you wanted to know if a patient had Diabetes Mellitus and there is nothing recorded for that event or previous events and there is no HBa1c or blood sugar recorded.
    - SYMPTOMS / CLNICAL HISTORY
        - ‘Nil’ or ‘No’ or ‘Ø documented in reference to symptom or clinical history recording in notes = NO
    - OBSERVATIONS
        - If no recording documented, recording box is blank = NOT RECORDED
        - For example Pain Score – if not documented on the chart in the relevant area this is not recorded

- CLINICAL FINDINGS / EVENTS
  - If a finding or event related to clinical presentation is not documented in notes = NO (for the reason that it would likely have been documented if it was there). For example respiratory arrest – should have been documented if it happened, therefore if not documented it didn't happen = 'No'.
  - 'Nil' or 'No' or 'Ǿ documented in reference to clinical findings in notes = NO
- MEDICATIONS
  - No information recorded; if you wanted to see if a patient had had pre-hospital analgesia and there was nothing written to the positive or negative to this (no information about this recorded)
  - "No antibiotics given due to inpatient team request" = NO
  - Intention to give a treatment, recorded on chart but NOT signed off by staff = NOT RECORDED

- **Not Available** – notes are not available

9.0 Clinical Confounder Variables

The measuring of clinical confounders is considered here. Confounder variables are those extraneous variables that correlate (positively or negatively) with both the dependent variable and the independent variables and therefore will have an effect on outcomes. We need to control for these factors to avoid a type 1 error, an erroneous conclusion the dependent variables are in a causal relationship with the independent variable. Confounding is a major threat to the validity of inferences made about cause and effect (internal validity).

The variables we have chosen below reflect, in the main, elements of chronic disease that will have an impact both on the person’s propensity to become sick (background ‘risk’) and also effect the illness outcome (ability and physiological reserve) to fight disease.

The confounders measured will be slightly different for each clinical indicator, dependant on them being relevant to the indicator or not. Confounders have been divided into Adults and Paediatric Confounders.

Documentation of Confounders: If no mention of any past history anywhere in notes then NR. If mention of any past medical history and particular confounder not mentioned or 'nil', or such like then 'no' (giving clinician benefit of the doubt). If written ‘no COPD’ but clearly has objective evidence of COPD (old notes/ FEVI/ CXR etc) then 'yes'. The point is to document co-morbidities, not comment on the quality of the medical notes. This applies to all in principle

9.1 ADULT CONFOUNDERS

9.1.1 Chronic Renal Failure

Definition	Co-morbid condition of Chronic Renal Failure (organ = kidneys)
Layout	NN (Number: 2 Characters)
Codeset (If Applicable)	0 = No<br>1 = Yes<br>2 = Not Recorded<br>3 = Not Available
Reported For	Includes: Appendicectomy, CT Head, Asthma, # NOF, MI, Pain, Antibiotics<br>Excludes: Discharge Summary

Description

Collection will be co-morbidities prior to event.

Check Renal Function prior to event

Renal Failure is defined for the purposes of the study as:

- GFR available in notes or with investigations within the previous 6 months (in adults only)
  - **>= 80 ml/min/1.73m^2:** no evidence kidney disease
  - **60 - 80 ml/min/1.73m^2:** Does not exclude renal disease, especially if proteinuria, abnormal urinary sediment, or hypertension is present.
  - **30 - 59 ml/min/1.73m^2:** Indicates moderate renal disease. Mild reductions in eGFR (50 - 59 mL/min/1.73m2) are not associated with increased mortality in people over 65 years of age.
  - **< 30 ml/min/1.73m^2:** Severe to end stage renal failure.
- Baseline Creatinine greater than 105 in men and greater than 90 in women. These criteria can be used if eGFR not available.

We are unable to retrospectively calculate the GFR consistently, as this is not reported for all patients and we will not have access to weight measures for all patients. The same issues arise with proteinuria and microalbuminuria for diagnosis of stages of CKD as this will not be consistently available for all patients for comparison. Renal function is acutely a marker of renal insult / acute kidney injury (hypoperfusion and ischaemia). If this insult is on top of compromised kidneys, their ability to do their job is severely compromised – leading to a buildup of toxic metabolites in the body and renal failure.

Numerator (If Applicable)	n/a
Denominator (If Applicable)	n/a
Expressed As	Categorical

Study Sites

9.1.2 Chronic Obstructive Pulmonary Disease

Definition	Co-morbid condition of Chronic Obstructive Pulmonary Disease (organ = lungs)
Layout	NN (Number: 2 Characters)
Codeset (If Applicable)	0 = No<br>1 = Yes<br>2 = Not Recorded<br>3 = Not Available
Reported For	Includes: Appendicectomy, CT Head, Asthma, # NOF, MI, Pain, Antibiotics<br>Excludes: Discharge Summary
Description	Collection will be co-morbidities prior to event.<br>**Check Spirometry, CXR prior to event**<br>COPD is defined for the purposes of the study as a baseline as<br>• Spirometry showing FEV1/FVC of less than 70%.<br>• CXR showing characteristics pathognomic of COPD (hyperinflation, emphysematous bullae, pulmonary hypertension)<br>• Documented COPD in clinical notes<br>The pulmonary component of COPD is characterized by airflow limitation that is not fully reversible. It is usually progressive and associated with an abnormal inflammatory response of the lung to noxious substances (i.e. cigarette smoking). COPD leads to a chronic hypoxic state and the development of respiratory failure (inability of respiratory gases to cross alveolar membrane). The chronic hypoxia will cause deterioration of end organ function and reduce reserve to inter-current illness; respiratory failure reduces physiological reserve to deal with acute illness and increases risk of morbidity and mortality.
Numerator (If Applicable)	n/a
Denominator (If Applicable)	n/a
Expressed As	Categorical

Study Sites

9.1.3 Diabetes Mellitus

Definition	Co-morbid condition of Diabetes Mellitus (all types, organ = pancreas)
Layout	NN (Number: 2 Characters)
Codeset (If Applicable)	0 = No<br>1 = Yes<br>2 = Not Recorded<br>3 = Not Available
Reported For	Includes: Appendicectomy, CT Head, Asthma, # NOF, MI, Pain, Antibiotics<br>Excludes: Discharge Summary
Description	Collection will be co-morbidities prior to event.<br>**Check BSL, HBa1c, Medications prior to event, BSL at event**<br>Diabetes is defined for the purposes of the study as baseline:<br>• Fasting plasma glucose level ≥ 7.0 mmol/L<br>• Casual plasma glucose ≥ 11.1 mmol/L<br>• Or glycated haemoglobin (Hb A1C) ≥ 6.5%<br>• Or current treatment with oral hypoglycaemic or insulin<br>A normal BSL on arrival in someone who is not on hypoglycaemic or insulin treatment for diabetes suggests the person does not have the condition.<br>Diabetes mellitus is a group of metabolic diseases in which a person has high blood sugar because the body either does not produce enough insulin or does not respond to the insulin that is produced. The high blood sugar has knock-on effects to all the body organ systems causing nephropathy, vasculopathy, retinopathy and neuropathy. For all these reasons DM is considered a confounder as it causes a gradual deterioration in end-organ function leading to a higher risk of inter-current illness and reduced capacity to respond to that illness.
Numerator (If Applicable)	n/a
Denominator (If Applicable)	n/a
Expressed As	Categorical

Study Sites

9.1.4 Smoking (Tobacco)

Definition	Co-morbid condition of Smoking (organ = all)
Layout	NN (Number: 2 Characters)
Codeset (If Applicable)	0 = Never Smoked<br>1 = Current Smoker<br>2 = Ex-Smoker<br>3 = Not Recorded<br>4 = Not Available
Reported For	Includes: Appendicectomy, CT Head, Asthma, # NOF, MI, Pain, Antibiotics<br>Excludes: Discharge Summary
Description	Collection will be prior to event.<br><br>Smoking is defined for the purposes of the study as baseline current smoker, ex-smoker, never smoked.<br>Smoking commonly leads to diseases that affect the heart and the lungs – being a major risk factor for AMI, Systemic Atherosclerosis, Stroke, COPD, Emphysema and Cancer (lung Cancer, cancers of the larynx, throat, mouth, bladder and pancreas). Therefore smoking can be directly related to many of our other confounding variables. Smokers may have an unhealthier, restricted lifestyle in general, which again will have an effect on the response to acute illness. Smoking causes micro-circulation injury – compounding the effects of end-organ ischaemia from acute illness.<br>**Never Smoked** = has never smoked<br>**Current Smoker** = currently smoking<br>**Ex-Smoker** = used to smoke and has now stopped (>28 days)<br>**Not Recorded** = no smoking status recorded at event. Status can be taken prior to event, but note it could have changed in the interim period. It is more likely we would over-pick up smokers this way, as people are more likely to stop smoking than begin smoking<br>**Not Available** = notes not available.
Numerator (If Applicable)	n/a
Denominator (If Applicable)	n/a
Expressed As	Categorical

Study Sites

9.1.5 Dementia

Definition	Co-morbid condition of Dementia (organ = brain)
Layout	NN (Number: 2 Characters)
Codeset (If Applicable)	0 = No<br>1 = Yes<br>2 = Not Recorded<br>3 = Not Available
Reported For	Includes: Appendicectomy, CT Head, Asthma, # NOF, MI, Pain, Antibiotics<br>Excludes: Discharge Summary
Description	Collection will be co-morbidities prior to event.<br><br>Dementia documented as past medical history during the index episode or prior to this.<br>We will be unable to retrospectively apply mini-mental state exams etc. Dementia is a confounder as memory of symptoms or inability to answer questions will impact on the time-to-treatment markers. It may also delay presentation to hospital, cause multiple re-attendances to hospital and predisposes to trauma by falling, causing injury. Those with dementia are likely to be less independent therefore having reduced physiological reserve to cope with acute illness. Delirium from acute illness can worsen dementia, making it more difficult in some cases to provide care without concurrent sedation (which comes with its own problems).
Numerator (If Applicable)	n/a
Denominator (If Applicable)	n/a
Expressed As	Categorical

Study Sites

9.1.6 Stroke

Definition	Co-morbid condition of Stroke (organ = brain)
Layout	NN (Number: 2 Characters)
Codeset (If Applicable)	0 = No<br>1 = Yes<br>2 = Not Recorded<br>3 = Not Available
Reported For	Includes: Appendicectomy, CT Head, Asthma, # NOF, MI, Pain, Antibiotics<br>Excludes: Discharge Summary
Description	Collection will be co-morbidities prior to event **(either Stroke, CVA or TIA)**<br><br>Stroke (sometimes termed CVA) is defined for the purposes of the study as a documented stroke episode, as past medical history, during the index episode. We will be unable to retrospectively apply neurological exams etc. Stroke is a confounder of memory, speech, movement and independence. It also stems from underlying vascular disease (meaning end-organ disease should be considered elsewhere). Inability to articulate symptoms or inability to answer questions will impact on the time-to-treatment markers. It may also delay presentation to hospital, cause multiple re-attendances to hospital and predisposes to trauma by falling, causing injury. Those with ongoing neurological issues are likely to be less independent therefore having reduced physiological reserve to cope with acute illness.
Numerator (If Applicable)	n/a
Denominator (If Applicable)	n/a
Expressed As	Categorical

Study Sites

9.1.7 Hypertension

Definition	Co-morbid condition of Hypertension (organ = vascular system)
Layout	NN (Number: 2 Characters)
Codeset (If Applicable)	0 = No<br>1 = Yes<br>2 = Not Recorded<br>3 = Not Available
Reported For	Includes: Appendicectomy, CT Head, Asthma, # NOF, MI, Pain, Antibiotics<br>Excludes: Discharge Summary and transient hypertension during ED visit in absence of known hypertension
Description	Collection will be co-morbidities prior to event.<br><br>Hypertension (HTN) or high blood pressure is defined for the purposes of the study as<br>• Documented known hypertension (treated or untreated) during the index episode.<br>• Blood pressure 200 systolic or 110 diastolic during ED event<br>• Being on anti-hypertensive medication will not be enough for a positive documentation of HTN, as they can also be used in other pathologies for rate-control etc.<br>HTN stems from underlying vascular disease (atherosclerosis) and results in increased systemic arterial blood pressure. It is associated with sedentary lifestyle, smoking, stress, obesity, salt and ETOH intake. It is a risk factor for end-organ vascular disease; stroke, AMI, Congestive Heart Failure, arterial aneurysm and chronic kidney disease. Reduced systemic vascular compliance and increased resistance will decrease the capacity of the vascular system to cope with acute illness. If acute illness is associated with lowering of the BP, this can be extremely bad for end-organ function, being used to a much higher baseline perfusion pressure.
Numerator (If Applicable)	n/a
Denominator (If Applicable)	n/a
Expressed As	Categorical

Study Sites

9.1.8 Ischaemic Heart Disease

Definition	Co-morbid condition of Ischaemic Heart Disease (organ = heart)
Layout	NN (Number: 2 Characters)
Codeset (If Applicable)	0 = No<br>1 = Yes<br>2 = Not Recorded<br>3 = Not Available
Reported For	Includes: Appendicectomy, CT Head, Asthma, # NOF, Pain, Antibiotics<br>Excludes: Discharge Summary, MI
Description	Collection will be co-morbidities prior to event.<br>**Check Coronary Angio report and/or Echo report and/or Exercise treadmill test prior to event, past medical history of MI, NSTEMI, Angina**<br>Ischaemic Heart Disease (IHD) also falls into the spectrum of disorders caused by vascular disease (stroke, dementia, chronic renal failure etc). IHD is defined for the purposes of the study as a documented known IHD, coronary heart disease or LVF, current Angina or previous AMI / NSTEMI, as past medical history, during the index episode. IHD is a confounder. It stems from underlying vascular disease (atherosclerosis) and results from reduced blood supply to the myocardium of the heart. The risk increase with age, smoking, hypercholesterolemia, diabetes and hypertension and genetics. End-organ disease should be considered elsewhere. Reduced myocardial blood supply (ischaemia) means inability of the heart to compensate in acute illness or stress to the body (I.e. surgery), thus increasing the risk of morbidity and mortality. It may also increase LOS (post-op AMI) and time-to treatment factors (acute AMI causes the fall and #NOF, therefore delaying surgery - in this case however, the delay would be appropriate.)<br>This co-morbidity is excluded from the MI dataset, as by definition the majority people who have had an MI will have ischaemic heart disease.
Numerator (If Applicable)	n/a
Denominator (If Applicable)	n/a
Expressed As	Categorical

Study Sites

9.1.9 Congestive Heart Failure

Definition	Co-morbid condition of Congestive Heart Failure (organ = heart)
Layout	NN (Number: 2 Characters)
Codeset (If Applicable)	0 = No<br>1 = Yes<br>2 = Not Recorded<br>3 = Not Available
Reported For	Includes: Appendicectomy, CT Head, Asthma, # NOF, Pain, Antibiotics, MI<br>Excludes: Discharge Summary
Description	Collection will be co-morbidities prior to event.<br>**Look up ECHO result and CXR prior to event**<br>Congestive Heart Failure (CHF) is defined for the purposes of the study as<br>• Documented known CHF as past medical history, during the index episode.<br>• Documented L ventricular ejection fraction less that 50% and/or R ventricular failure and/or diastolic dysfunction on Echocardiogram<br>CHF is the inability of the heart to supply sufficient blood flow to meet the body's needs, as myocardium is damaged, contractility is reduced and the stroke volume / cardiac output is also reduced. It usually stems from underlying IHD or HTN and results from reduced blood supply to the myocardium of the heart. The reduced cardiac output leads to blood vessel congestion in the lungs and pulmonary oedema / hypoxia and also causes congestion elsewhere in the body leading to end-organ damage. Reduced cardiac output means inability of the heart to compensate in acute illness or stress to the body (I.e. surgery), thus increasing the risk of morbidity and mortality. It may also increase LOS (post-op complications) and time-to treatment factors (unfeasible to ventilate a patient for operation if they have acute pulmonary oedema).<br><br>**No** = none of the above for definition<br>**Yes** = any of the above for definition.<br>**Not Recorded** = no mention of any PMHx or CHF in clinical notes, no mention in old notes of CHF and no previous ECHO's.
Numerator (If Applicable)	n/a
Denominator (If Applicable)	n/a
Expressed As	Categorical

Study Sites

9.1.10 Anticoagulation

Definition	Co-morbid condition of Anticoagulation on Warfarin or Dabigatran
Layout	NN (Number: 2 Characters)
Codeset (If Applicable)	0 = No<br>1 = Yes<br>2 = Not Recorded<br>3 = Not Available
Reported For	Includes: Appendicectomy, CT Head, Asthma, # NOF, Antibiotics, MI<br>Excludes: Discharge Summary, Pain
Description	Collection will be co-morbidities prior event.<br><br>Anticoagulation is defined for the purposes of the study as<br>• Documented as taking Warfarin or Dabigatran as part of regular medications.<br>Both can lead to worse outcomes in trauma due to bleeding. They can also act as confounder in sepsis, worsening the effects of DIC etc.
Numerator (If Applicable)	n/a
Denominator (If Applicable)	n/a
Expressed As	Categorical

Study Sites

9.1.11 Immunosuppression

Definition	Co-morbid condition of Immunosuppression.
Layout	NN (Number: 2 Characters)
Codeset (If Applicable)	0 = No<br>1 = Yes – Long Term Prednisone Use<br>2 = Yes – Azathioprine use<br>3 = Yes – Methotrexate use<br>4 = Yes – Sulphasalazine use<br>5 = Yes – On Current Chemotherapy<br>6 = Yes – Organ Transplant<br>7 = Yes – Other Immunosuppressant Medication use<br>8 = Yes – Neutrophils <=0.5<br>9 = Yes – Multiple reasons for Immunosuppression<br>10 = Not Recorded<br>11 = Not Available
Reported For	Includes: Appendicectomy, CT Head, Asthma, # NOF, Antibiotics, MI, Pain<br>Excludes: Discharge Summary
Description	Collection will be co-morbidities prior to event.<br><br>Immunosuppression is defined for the terms of the study as reduced efficacy of the immune system. This can happen in many ways and is often induced by medications. This predisposes to infection and more so to serious, opportunistic and nosocomial infection as the body's natural defence systems are weakened.
Numerator (If Applicable)	n/a
Denominator (If Applicable)	n/a
Expressed As	Categorical

Study Sites

9.1.12 ASA Status (Pre-Operatively)

Definition	A grading system used by anaesthetists to assess the fitness of patient before surgery.
Layout	NN (Number: 2 Characters)
Codeset (If Applicable)	1. 1= A normal healthy patient.<br>2. 2= A patient with mild systemic disease.<br>3. 3= A patient with severe systemic disease.<br>4. 4= A patient with severe systemic disease that is a constant threat to life.<br>5. 5= A moribund patient who is not expected to survive without the operation.<br>6. 6= A declared brain-dead patient whose organs are being removed for donor purposes<br>7. 1e =The suffix ‘e’ is added for emergency surgery<br>8. 2e<br>9. 3e<br>10. 4e<br>11. 5e<br>12. Not Recorded<br>13. Not Applicable<br>14. Not Available
Reported For	Includes: Appendicectomy, CT Head, # NOF, MI, Antibiotics<br>Excludes: Discharge Summary, Asthma, Pain
Description	Collection will be co-morbidities prior to event.<br>Anesthesia providers use this scale to indicate the patient's overall physical health or "sickness" preoperatively. A person with a higher ASA score will have a higher risk of adverse outcome from either the anaesthetic or the surgery itself.<br>ASA score is recorded as written in the Operation notes<br>Otherwise:<br>Not Recorded = not recorded on anaesthesia or surgical charts if the patient had surgery<br>Not Applicable = the patient did not have surgery and therefore did not need an ASA score<br>Not Available = clinical notes not available
Numerator (If Applicable)	n/a
Denominator (If Applicable)	n/a
Expressed As	Categorical

Study Sites

9.1.13 Independence

Definition	Co-morbid condition of Independence.
Layout	NN (Number: 2 Characters)
Codeset (If Applicable)	0 = Fully Independent Living<br>1 = Partially Assisted Living (own home)<br>2 = Residential (partially assisted) level of care<br>3 = Hospital (fully assisted) level of care<br>4 = Not Recorded<br>5 = Not Available
Reported For	Includes: Appendicectomy, CT Head, Asthma, # NOF, Pain, Antibiotics, Discharge Summary, MI<br>Excludes
Description	Collection will be data available at event and co-morbidities will be present prior to event.<br><br>Independence is a strong confounder. Reduced independence and living at home means more difficulties accessing medical care, delays and also increased risk of trauma. Needing fully assisted care (including being bed-bound) is associated with a high mortality due to decreased functional reserve and capacity to cope with acute illness. Assisted living can be associated with older age, and more medical co-morbidities as a cause of needing assistance. This includes help from family members, friends or co-coordinated services for help with ADLs one or more times a week. Chronic co-morbidities will reduce capacity for dealing with acute illness.
Numerator (If Applicable)	n/a
Denominator (If Applicable)	n/a
Expressed As	Categorical

Study Sites

9.2 PAEDIATRIC CONFOUNDERS

9.2.1 Pre-Term Birth

Definition	Birth before 37 weeks of gestational age.
Layout	NN (Number: 2 Characters)
Codeset (If Applicable)	0 = No<br>1 = Yes before 37 weeks, but after 32 weeks<br>2 = Yes before 32 weeks<br>2 = Not Recorded<br>3 = Not Available
Reported For	Includes: Appendicectomy, CT Head, Asthma, Antibiotics, Pain<br>Excludes: Discharge Summary
Description	Collection will be co-morbidities prior to event.<br>**Look at neonatal record if available**<br><br>Preterm birth is the leading cause of neonatal death and infant mortality, often as a result of respiratory distress syndrome due to immature lung development. Serious morbidity however, is uncommon after 32 weeks gestation, mortality and disability seem to be concentrated in those infants with birth weights of less than 1000g. Pre-term and extremely low-birth-weight children who survive are also at high risk of neurological disability, chronic lung disease and other chronic health disorders.<br>If age <5 and not mentioned = not recorded.<br>If 'nil PMHx' or such like then = 'no'.<br>If >5 and not mentioned then assume not present = 'no' - should been picked up by now.
Numerator (If Applicable)	n/a
Denominator (If Applicable)	n/a
Expressed As	Categorical

Study Sites

9.2.2 Pre-Term Birth Weight

Definition	Extremely –low birth weight (ELBW) is less than 1000g.<br>Very-Low Birth Weight(VLBW) is less than 1500g<br>Low Birth Weight (LBW) is less than 2500g
Layout	NN (Number: 2 Characters)
Codeset (If Applicable)	0 = No<br>1 = Yes ELBW<br>2 = Yes VLBW<br>3 = Yes LBW<br>4 = Not Recorded<br>5 = Not Available
Reported For	Includes: Appendicectomy, CT Head, Asthma, Antibiotics, Pain<br>Excludes: Discharge Summary
Description	Collection will be co-morbidities prior to event.<br>**Look at neonatal record if available**<br><br>Child mortality and disability are often concentrated in those with birth weights of less than 1000g. Pre-term and extremely low-birth-weight children who survive are also at high risk of neurological disability, chronic lung disease and other chronic health disorders (such as blindness and deafness).<br><br>"In 2002, the first-year survival rate was 13.8% for infants with birth weights less than 500 g, 51% for infants with birth weights of 500-749 g, 84.5% for infants with birth weights of 750-1000 g. Infants with extremely low birth weights (ELBWs) are more susceptible to all of the possible complications of premature birth, both in the immediate neonatal period and after discharge from the nursery"<br><br>(http://emedicine.medscape.com/article/979717-overview#aw2aab6b3)<br><br>If age <5 and not mentioned = not recorded.<br>If 'nil PMHx' or such like then = 'no'.<br>If >5 and not mentioned then assume not present = 'no' - should been picked up by now.
Numerator (If Applicable)	n/a
Denominator (If Applicable)	n/a
Expressed As	Categorical

Study Sites

9.2.3 Congenital Heart Disease

Definition	Refers to any of the spectrum of heart and/or great vessel defects a child can be born with
Layout	NN (Number: 2 Characters)
Codeset (If Applicable)	0 = No<br>1 = Yes<br>2 = Not Recorded<br>3 = Not Available
Reported For	Includes: Appendicectomy, CT Head, Asthma, Antibiotics, Pain<br>Excludes: Discharge Summary
Description	Collection will be co-morbidities prior to event.<br>**Look at neonatal record if available**<br><br>These children can be more susceptible to severe illness and infection.<br>If age <5 and not mentioned = not recorded.<br>If 'nil PMHx' or such like then = 'no'.<br>If >5 and not mentioned then assume not present = 'no' - should been picked up by now.
Numerator (If Applicable)	n/a
Denominator (If Applicable)	n/a
Expressed As	Categorical

Study Sites

9.2.4 Chronic Lung Disease

Definition	Refers to any of the spectrum of childhood chronic lung disease
Layout	NN (Number: 2 Characters)
Codeset (If Applicable)	0 = No<br>1 = Yes<br>2 = Not Recorded<br>3 = Not Available
Reported For	Includes: Appendicectomy, CT Head, Asthma, Antibiotics, Pain<br>Excludes: Discharge Summary
Description	Collection will be co-morbidities prior to event.<br>**Look at neonatal record if available (prem)**<br><br>Chronic lung disease in children refers to bronchiectasis, cystic fibrosis, recurrent aspiration (for example in cerebral palsy), bronchopulmonary dysplasia (secondary to prematurity), connective tissue disorders and congenital lung disorders (Congenital Fibrosing Alveolitis).<br><br>These children are more susceptible to severe illness and infection, as well as multi-resistant organisms in the case of those children getting recurrent infections.<br><br>If age <5 and not mentioned = not recorded.<br>If 'nil PMHx' or such like then = 'no'.<br>If >5 and not mentioned then assume not present = 'no' - should been picked up by now.
Numerator (If Applicable)	n/a
Denominator (If Applicable)	n/a
Expressed As	Categorical

Study Sites

9.2.5 Smoking (Tobacco) in the Home

Definition	Co-morbid condition of Smoking (organ = all)
Layout	NN (Number: 2 Characters)
Codeset (If Applicable)	0 = Yes - Inside<br>1 = Yes - Outside<br>2 = Occasional - Visitors<br>3 = Not Recorded<br>4 = Not Available
Reported For	Includes: Asthma<br>Excludes: Discharge Summary, Appendicectomy, CT Head, Pain, Antibiotics
Description	Collection will be co-morbidities prior to event.<br><br>Smoking in the home is associated with the development of asthma in children and exacerbations of established asthma.<br>Yes – Inside = one or more family members who normally reside in the house with the child will smoke in the house or car in the presence of the child in a consistent fashion.<br>Yes – Outside = one or more family members who normally live with the child, but restrict their smoking activities to outside the house or confined spaces they may be in with the child<br>Occasional – Visitors = may mean extended family, friends or people who visit the house (babysitters) may smoke in a confined area with the child for short periods of time. Not consistent.<br>Not Recorded = household smoking status not recorded at event.<br>Not Available = notes not available.<br><br>Smoking will also be captured as a confounder for children (see adult definition) – it is not that uncommon to meet young children and teens who smoke, this should be captured.
Numerator (If Applicable)	n/a
Denominator (If Applicable)	n/a
Expressed As	Categorical

Study Sites

9.2.6 Anticoagulation

Definition	Co-morbid condition of Anticoagulation on Warfarin or Dabigatran
Layout	NN (Number: 2 Characters)
Codeset (If Applicable)	0 = No<br>1 = Yes<br>2 = Not Recorded<br>3 = Not Available
Reported For	Includes: Appendicectomy, CT Head, Antibiotics > 1 month<br>Excludes: Discharge Summary, Asthma, Pain, Antibiotics <= 1 month
Description	Collection will be co-morbidities prior event.<br><br>Anticoagulation is defined for the purposes of the study as documented as taking Warfarin or Dabigatran as part of regular medications. Both can lead to worse outcomes in trauma due to bleeding. They can also act as confounder in sepsis, worsening the effects of DIC etc.
Numerator (If Applicable)	n/a
Denominator (If Applicable)	n/a
Expressed As	Categorical

Study Sites

9.2.7 Immunosuppression

Definition	Co-morbid condition of Immunosuppression.
Layout	NN (Number: 2 Characters)
Codeset (If Applicable)	0 = No<br>1 = Yes – Long Term Prednisone Use<br>2 = Yes – Azathioprine use<br>3 = Yes – Methotrexate use<br>4 = Yes – Sulphasalazine use<br>5 = Yes – On Current Chemotherapy<br>6 = Yes – Organ Transplant<br>7 = Yes – Other Immunosuppressant Medication use<br>8 = Yes – Neutrophils <=0.5<br>9 = Yes – Multiple reasons for Immunosuppression<br>10 = Not Recorded<br>11 = Not Available
Reported For	Includes: Antibiotics, Appendicitis, Pain, Asthma<br>Excludes: CT Head, Discharge Summary
Description	Collection will be co-morbidities prior to event.<br><br>Immunosuppression is defined for the terms of the study as reduced efficacy of the immune system. This can happen in many ways and is often induced by medications. This predisposes to infection and more so to serious, opportunistic and nosocomial infection as the body's natural defence systems are weakened.
Numerator (If Applicable)	n/a
Denominator (If Applicable)	n/a
Expressed As	Categorical

Study Sites

9.2.8 ASA Status (Pre-Operatively)

Definition	A grading system used by anaesthetists to assess the fitness of patient before surgery.
Layout	NN (Number: 2 Characters)
Codeset (If Applicable)	15. 1= A normal healthy patient.<br>16. 2= A patient with mild systemic disease.<br>17. 3= A patient with severe systemic disease.<br>18. 4= A patient with severe systemic disease that is a constant threat to life.<br>19. 5= A moribund patient who is not expected to survive without the operation.<br>20. 6= A declared brain-dead patient whose organs are being removed for donor purposes<br>21. 1e =The suffix 'e' is added for emergency surgery<br>22. 2e<br>23. 3e<br>24. 4e<br>25. 5e<br>26. Not Recorded<br>27. Not Applicable<br>28. Not Available
Reported For	Includes: Appendicectomy, CT Head, Antibiotics<br>Excludes: Discharge Summary, Asthma, Pain
Description	Collection will be co-morbidities prior to event.<br>Anesthesia providers use this scale to indicate the patient's overall physical health or "sickness" preoperatively. A person with a higher ASA score will have a higher risk of adverse outcome from either the anaesthetic or the surgery itself.<br>ASA score is recorded as written in the Operation notes<br>Otherwise:<br>Not Recorded = not recorded on anaesthesia or surgical charts if the patient had surgery<br>Not Applicable = the patient did not have surgery and therefore did not need an ASA score<br>Not Available = clinical notes not available
Numerator (If Applicable)	n/a
Denominator (If Applicable)	n/a
Expressed As	Categorical

Study Sites

9.2.9 Independence

Definition	Co-morbid condition of Independence.
Layout	NN (Number: 2 Characters)
Codeset (If Applicable)	0 = Fully Independent Living<br>1 = Independence Child with Caregiver<br>3 = Partially Assisted Living (own home)<br>4 = Residential (partially assisted) level of care<br>5 = Hospital (fully assisted) level of care<br>6 = Not Recorded<br>7 = Not Available
Reported For	Includes: Asthma, Pain, Appendectomy, Antibiotics (> 1 month old), CT Head, Discharge Summary<br>Excludes: Antibiotic Indicator <= 1 month old.
Description	Collection will be data available at event and co-morbidities will be present prior to event.<br>Independence is a strong confounder. Reduced independence and living at home means more difficulties accessing medical care, delays and also increased risk of trauma. Needing fully assisted care (including being bed-bound) is associated with a high mortality due to decreased functional reserve and capacity to cope with acute illness. Assisted living can be associated with more medical co-morbidities as a cause of needing assistance. This includes help from family members, friends or co-coordinated services for help with ADLs one or more times a week. Chronic co-morbidities will reduce capacity for dealing with acute illness.<br>**Fully Independent** = live independently, no caregiver<br>**Independent Child with Caregiver** = live independently (with no aids etc, however as all children do, they rely on a caregiver to assist them with daily living – transport, food preparation etc.<br>**Partially Assisted Living (own home)** – child has disability or impairment where they need aids and caregivers to help with mobility, speech, sight etc, but still live in their own home.<br>**Residential (partially assisted) Level of Care** = child lives in a specially staffed residential home (i.e. IHC home), level of care unable to be delivered in patients own home. Able to do some things for themselves.<br>**Hospital (fully assisted) Level of Care** = child unable to care for themselves and need help with all ADL's, by specially trained staff, that cannot be carried out in the patient's own home (i.e. Ventilation in some cases)
Numerator (If Applicable)	n/a
Denominator (If Applicable)	n/a
Expressed As	Categorical

Study Sites

10.0 Clinical Indicators

Proposed Data Collection Process

1) SSED team requests all patients with ICD-10 Code related to the quality indicator we are studying, during the study period, filtered by the 4 Study Sites, from NZHIS.

2) NZHIS provides list of NHIs and Event Numbers and similar demographic detail to that supplied for National Data collection for each event.

3) SSED request clinical records at each study site to pull files by NHI, supplementary electronic information stored by DHB will also be provided per NHI and event number (will subsequently be matched to NZHIS Data).

4) Records Pulled by Clinical Records at study site and SSED Team informed that records are now ready to view.

5) Data Collection for Quality Indicators commences.

Data collection for all quality indicators will have all of the previous variables in Units 2.0 to 6.0 and 8.0 recorded for each of them.

10.1 Time to Reperfusion in Acute Myocardial Infarction (AMI)

Acute Myocardial Infarction (from Thygesen et al[26]) is: "evidence of myocardial necrosis, in a clinical setting consistent with myocardial ischaemia". For our study purposes, the criteria below define myocardial infarction to be eligible for analysis:

- Detection of typical rise and/or gradual fall of cardiac biomarkers (troponin)
- *and* clinical evidence of myocardial ischaemia
- *and* at least one of the following is required:
    - Ischemic symptoms
    - ECG changes indicative of ischemia:
        - New >1mm ST elevation in 2 contiguous lead groups
        - New > 2mm ST elevation in 2 contiguous lead groups
        - New Left Bundle Branch Block.
        - Development of pathologic Q waves on the ECG
    - Imaging evidence of new loss of viable myocardium or a new regional wall motion abnormality.

The table below, from the same clinical paper[26] defines all the various types of myocardial infarction:

Table 1 Clinical classification of different types of myocardial infarction

**Type 1**	Spontaneous myocardial infarction related to ischaemia due to a primary coronary event such as plaque erosion and/or rupture, fissuring, or dissection
**Type 2**	Myocardial infarction secondary to ischaemia due to either increased oxygen demand or decreased supply, e.g. coronary artery spasm, coronary embolism, anaemia, arrhythmias, hypertension, or hypotension
**Type 3**	Sudden unexpected cardiac death, including cardiac arrest, often with symptoms suggestive of myocardial ischaemia, accompanied by presumably new ST elevation, or new LBBB, or evidence of fresh thrombus in a coronary artery by angiography and/or at autopsy, but death occurring before blood samples could be obtained, or at a time before the appearance of cardiac biomarkers in the blood
**Type 4a**	Myocardial infarction associated with PCI
**Type 4b**	Myocardial infarction associated with stent thrombosis as documented by angiography or at autopsy
**Type 5**	Myocardial infarction associated with CABG

Figure 2 (Thygesen et al)

The 2008 ACC/AHA STEMI/NSTEMI Performance Measures Definitions[27] are:

9. Reperfusion therapy	AMI patients with ST-segment elevation or LBBB on the ECG performed closest to arrival receiving either fibrinolysis or primary PCI or who are transferred to another facility for primary PCI
10. Time from ED arrival at STEMI referral facility to ED discharge from STEMI referral facility in patients transferred for primary PCI†	Median time from ED arrival at STEMI referral facility to ED discharge from STEMI referral facility for AMI patients with ST-segment elevation or LBBB on the ECG performed closest to hospital arrival time who are transferred to a STEMI receiving facility for primary PCI
11. Time from ED arrival at STEMI referral facility to primary PCI at STEMI receiving facility among transferred patients†	Median time from patient arrival at a STEMI referral facility's ED to time of primary PCI at a STEMI receiving facility for AMI patients presenting with ST-segment elevation or LBBB on the ECG performed closest to first hospital arrival time who are transferred to a STEMI receiving facility for primary PCI

Figure 3 (Krumholtz et al)

Reperfusion:

Prompt restoration of myocardial blood flow is essential to optimize myocardial salvage and to reduce mortality. A decision must be made as soon as possible by the treating clinician as to whether reperfusion will be achieved with thrombolytic agents, or primary (direct) percutaneous coronary intervention (PCI), depending on what is locally available. Reperfusion therapy for AMI aims to open compromised coronary arteries and to diminish the damage caused by reduced blood flow to the myocardium (the 'heart attack'). It is intuitive that the sooner this happens, the less myocardial damage there will be.

Multiple randomized clinical trials have shown that reperfusion therapy provided to eligible patients presenting with AMI reduces the risk of death due to all causes. The timeliness of reperfusion therapy is of central importance, because the benefits of therapy diminish rapidly with delays in treatment [28, 29].

Thrombolysis:

Not all patients having a heart attack are suitable for thrombolytic treatment. Patients are **eligible** for thrombolytic treatment if:

- They have definite signs and symptoms of a heart attack including typical evidence on the electrocardiogram (ECG) as detailed above.
- They present for treatment within 12 hours of symptoms onset
- There is no reason why thrombolytic treatment might be harmful to them, and
- There is no good reason to delay giving thrombolytic treatment (i.e. work-up for alternative causes of chest pain where thrombolysis treatment would not be indicated – Aortic Dissection, Pericarditis or active haemorrhage).
- The facility does not have the capability of expert, prompt intervention with primary PCI within 90 minutes of first medical contact.
- Patients who present to a facility in which the relative delay necessary to perform primary PCI (the expected door-to-balloon time minus the expected door-to-needle time) is greater than one hour.

The time interval from first patient contact to initiation of thrombolytic drug infusion should be within 30 minutes of first medical contact (arrival at hospital) (US ACC/AHA Guidelines[30], UK National Service Framework for Coronary Heart Disease: Chapter 5[31]) or within 60 minutes of calling professional help (UK[31]) or within 60 minutes of presentation to hospital (Aus, NZ)

- Contra-Indications to Thrombolysis include[32]:
  - **Absolute**: Current active haemorrhage, significant closed head or facial trauma < 3 months ago, suspected aortic dissection, any prior ICH, Ischaemic Stroke < 3 months, known structural cerebral vascular lesion, known malignant intracranial neoplasm.
  - **Relative**: Current anticoagulants, non-compressible vascular punctures, recent major surgery < 3 weeks, traumatic or prolonged (>10 mins) CPR, recent (< 4 weeks) GI bleed, severe HTN, Previous streptokinase allergy, proven strep throat infection, pregnancy.

Percutaneous coronary intervention:

If high-quality PCI is available, multiple randomized trials have shown enhanced survival after the first hour of symptoms, compared to thrombolysis, with a lower rate of intracranial haemorrhage and recurrent MI. 2009 ACC/AHA Guidelines for the Management of Patients with STEMI[30] recommends the use of primary PCI as a first option, for any patient with an acute STEMI who can undergo the procedure within 90 minutes of first medical contact, by persons skilled in the procedure. Australia / NZ guidelines[32] are as figure 4 below:

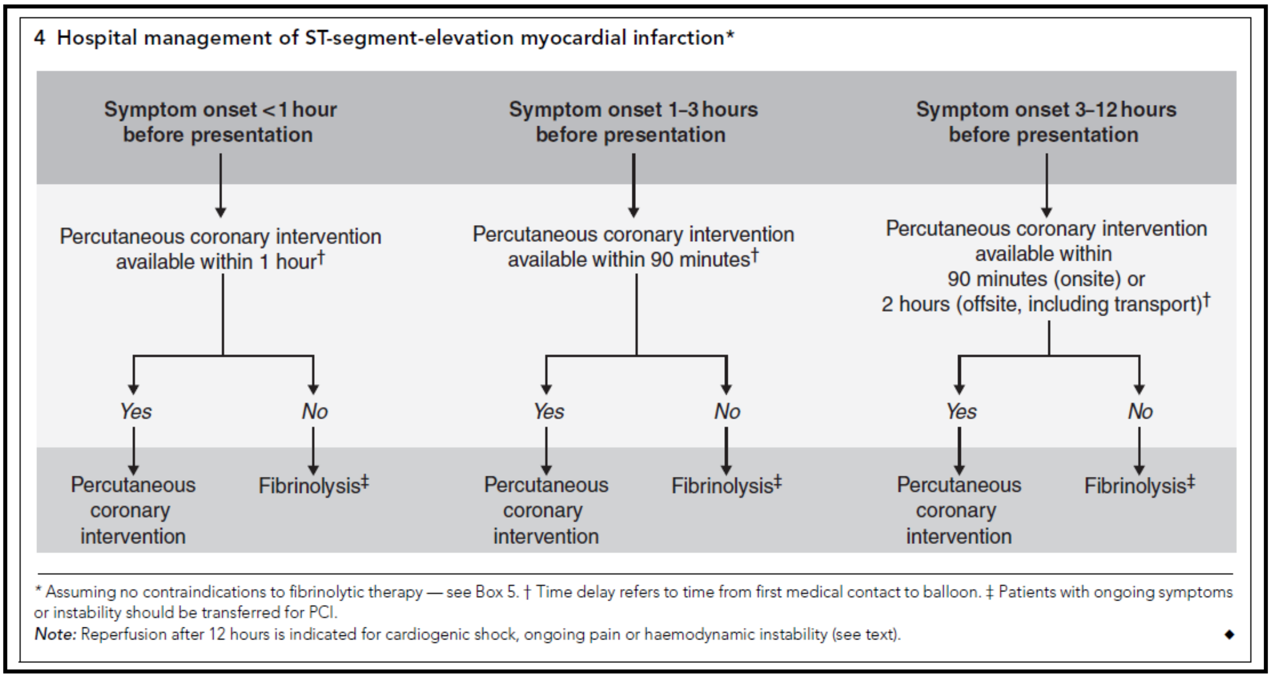

Figure 4 (National Heart Foundation Australia et al)

Time from patient arrival in the ED to initiation of reperfusion therapy for eligible cases [3, 30, 32-34]:

1) Door to Needle Time in Thrombolysis < 30 minutes (US, UK, Canada)
2) Door to Needle time in Thrombolysis <60 minutes (NZ, Aus)
3) Call to Needle Time in Thrombolysis < 60 minutes (UK)
4) Door to First Balloon Inflation Time for Primary PCI < 90 minutes (US, UK, Canada, Aus, NZ)
5) Call to First Balloon Inflation Time for Primary PCI < 150 minutes (UK)

Key Performance Indicators:

- ACC/AHA guidelines recommend that thrombolysis be provided within 30 minutes of first medical system contact and that primary PCI be provided within 90 minutes of first medical system contact for patients presenting with STEMI[28]
- ACHS KPI's measure thrombolysis within 60 minutes of presentation for AMI and also for Primary PCI [3]
- NHS (UK) KPI is 30 minutes for Thrombolysis and 60 minutes for PCI (used in MINAP)

ED Crowding and Hospital Access Block have been associated with adverse outcomes to time to reperfusion in AMI. Schull et al[35] carried out a retrospective study looking at ED crowding and delay to thrombolysis in AMI. They defined crowding as (hospital) network ambulance diversion – diversion of over 60% was high crowding, < 60% moderate crowding, 0% was no crowding. During times of moderate crowding there was an increased median door-to-needle time of 3 minutes and during times of high crowding there was a significant increase in door to needle times of 5.8 minutes.

There is level one evidence to the fact that the earlier the reperfusion the better the outcome.

Time to Reperfusion:

- Is the interval between time of presentation and time of definitive reperfusion treatment (both thrombolysis and PCI)
- Time to Thrombolysis for the SSED NRP will be < 60 minutes
- Time to PCI for the SSED NRP will be < 90 minutes

ELIGIBILITY CRITERIA: The following variables will be recorded as part of assessing eligibility for inclusion into the data collection (also will assess appropriateness of treatment given as well as timeliness). Eligibility for reperfusion is defined by the discussion at the beginning of this unit:

First Abnormal ECG: Description of Changes

• > 1mm ST Segment elevation in 2 contiguous limb leads	X
• > 2mm ST segment elevation in 2 contiguous chest leads	X
• New Left Bundle Branch Block	X
• New ST Depression in V1-V3 (Inferobasal MI)	X
• None of the Above	
• Not Recorded	
• Not Available	

Acute Trans-thoracic Echocardiogram Result

• Imaging evidence of new loss of viable myocardium	X
• New regional wall motion abnormality.	X
• None of the Above	
• Not Recorded	
• Not Available	

Ischaemic Symptoms (Rest Chest Pain > 20 mins or Angina Equivalent)

• No	
• Yes	X
• Not recorded	
• Not Available	

Length of Ischaemic Symptoms pre-hospital

• Less than 3 hours	X
• Less than 12 hours	X
• More than 12 hours	
• No symptoms	
• Not Recorded	
• Not Available	

Troponin (on arrival)

NNN (units depending on Troponin used – will be determined for each site when data collection starts)

Troponin Type (used on arrival)

• Troponin-**T** Roche
• HS Troponin-**T** Roche
• Not Recorded
• Not Available

Troponin at 6 to 9 hours (from pain)

NNN (units depending on Troponin used – will be determined for each site when data collection starts)

Troponin Type (used at 6-9 hours from pain)

• Troponin-**T** Roche
• HS Troponin-**T** Roche
• Not Recorded
• Not Available

Absolute Contraindications to Thrombolysis

• None	X
• Any prior ICH	
• CVA <= 3 months	
• Closed HI <= 3months	
• Closed Facial Injury <= 3 months	
• Suspected Aortic Dissection	
• Active Bleeding	
• Known structural cerebrovascular lesion	
• Known malignant intracranial neoplasm	
• Current anticoagulation	
• Non-compressible vascular puncture	
• Major surgery <= 3 weeks	
• CPR traumatic or prolonged >10 mins	
• GI Bleed in the last <= 4 weeks	
• Severe HTN – systolic >=180	
• Streptokinase allergy	
• Pregnancy	
• Proven Strep throat infection	
• Not Recorded	
• Not Available	

Acute PCI Available (at same Institution)

• No
• Yes

Contraindications to PCI

• None	X
• Diffusely diseased small calibre artery	
• Diffusely diseased small calibre vein graft	
• Other Coronary Anatomy not feasible to PCI	
• Anatomy unfeasible to catheter insertion	
• Not Recorded	
• Not Available	

Received Reperfusion Treatment (see below)

• No	
• Thrombolysis	X
• PCI	X
• CABG	X
• Not Available	

If No Reperfusion and Eligible (reason – free text)

10.1.1 First ECG Time

Definition	Time of first ECG on arrival to ED
Layout	DD/MM/CCYY HH:MM:SS (Date and Time: 18 characters separated by a space between year and hour values)
Codeset (If Applicable)	n/a
Reported For	Includes: All ED Presentations with AMI<br>Excludes:
Description	The time the first ECG was taken on presentation to hospital
Numerator (If Applicable)	n/a
Denominator (If Applicable)	n/a
Expressed As	Numeric (Time)

Study Sites

10.1.2 Diagnostic ECG Time

Definition	Time of first ECG fitting reperfusion criteria
Layout	DD/MM/CCYY HH:MM:SS (Date and Time: 18 characters separated by a space between year and hour values)
Codeset (If Applicable)	n/a
Reported For	Includes: All Patients with AMI<br>Excludes:
Description	Some patients may not fulfill reperfusion criteria on their first ECG, however if they do develop ECG changes consistent with reperfusion, the time to therapy should still be emergent. If the first ECG is diagnostic this time will be the same as the first ECG time. A diagnostic ECG shows the characteristics of acute myocardial infarction:<br>New >1mm ST elevation in 2 contiguous lead groups<br>New > 2mm ST elevation in 2 contiguous lead groups<br>New Left Bundle Branch Block.<br>New ST Depression in V1-V3 (Inferobasal MI)
Numerator (If Applicable)	n/a
Denominator (If Applicable)	n/a
Expressed As	Numeric (Time)

Study Sites

10.1.3 Thrombolytic Time

Definition	Documented time in notes of thrombolytic therapy delivery
Layout	DD/MM/CCYY HH:MM:SS (Date and Time: 18 characters separated by a space between year and hour values)
Codeset (If Applicable)	n/a
Reported For	Includes: All Patients with AMI who get Thrombolysis<br>Excludes:
Description	The time thrombolytic medication was administered to the patient (whether this is in the Emergency Department, CCU or elsewhere).
Numerator (If Applicable)	n/a
Denominator (If Applicable)	n/a
Expressed As	Numeric (Time)

Study Sites

10.1.4 PCI Time

Definition	Documented time in notes of first PCI reperfusion device used in those patients undergoing PCI
Layout	DD/MM/CCYY HH:MM:SS (Date and Time: 18 characters separated by a space between year and hour values)
Codeset (If Applicable)	n/a
Reported For	Includes: All Patients with AMI who get PCI<br>Excludes:
Description	The time the first reperfusion device (angioplasty balloon, stent clot evacuation or other) deployed to obtain coronary artery flow again.
Numerator (If Applicable)	n/a
Denominator (If Applicable)	n/a
Expressed As	Numeric (Time)

Study Sites

10.1.5 Failed Thrombolysis

Definition	Patients who present with AMI who receive acute thrombolysis treatment which fails.
Layout	N (Number: 1 Characters)
Codeset (If Applicable)	0 = No<br>1 = No Thrombolysis<br>2 = Yes<br>3 = Not Recorded<br>4 = Not Available
Reported For	Includes: All Patients with AMI acute (i.e. Non-Elective)<br>Excludes:
Description	Those who are given thrombolysis which fails to adequately reperfuse the myocardium. This may necessitate referral for “rescue” PCI.
Numerator (If Applicable)	n/a
Denominator (If Applicable)	n/a
Expressed As	Categorical

Study Sites

10.1.6 Rescue PCI

Definition	Patients who present with AMI who receive acute thrombolysis treatment which fails and necessitates rescue PCI
Layout	N (Number: 1 Characters)
Codeset (If Applicable)	0 = No<br>1 = Yes – Same Institution<br>3 = Yes - Transferred<br>4 = Not Recorded<br>5 = Not Available
Reported For	Includes: All Patients with AMI acute (i.e. Non-Elective)<br>Excludes:
Description	
Numerator (If Applicable)	n/a
Denominator (If Applicable)	n/a
Expressed As	Categorical

Study Sites

10.1.7 Primary CABG Surgery

Definition	Patients who present with AMI who require primary CABG for reperfusion
Layout	N (Number: 1 Characters)
Codeset (If Applicable)	0 = No<br>1 = Yes – Same Institution<br>3 = Yes - Transferred<br>4 = Not Recorded<br>5 = Not Available
Reported For	Includes: All Patients with AMI acute (i.e. Non-Elective)<br>Excludes:
Description	
Numerator (If Applicable)	n/a
Denominator (If Applicable)	n/a
Expressed As	Categorical

Study Sites

10.1.8 Primary CABG Time

Definition	Documented time in notes of primary coronary artery bypass grafting for primary reperfusion of myocardium
Layout	DD/MM/CCYY HH:MM:SS (Date and Time: 18 characters separated by a space between year and hour values)
Codeset (If Applicable)	n/a
Reported For	Includes: All Patients with AMI who get primary CABG (non-elective)<br>Excludes:
Description	The surgery start time of primary coronary artery bypass grafting.
Numerator (If Applicable)	n/a
Denominator (If Applicable)	n/a
Expressed As	Numeric (Time)

Study Sites

10.2 Time to Surgery for Fracture Neck of Femur (#NOF)

The British Orthopaedic Association and British Geriatric Society state: "All patients with hip fracture who are medically fit should have surgery within 48 hours of admission, and during normal working hours[36]". 'Early' surgery for fracture neck of femur has been defined as within 48 hours by the Scottish Intercollegiate Guidelines Network[37] and within 24 hours by the New Zealand Guidelines Group[38].

Hip fractures are associated with high mortality rates; ("about 10% of people with a hip fracture die within 1 month, and about 30% within 12 months, although only around a third of these will be due to the fracture[37]") and temporary (sometimes permanent) disability. Current UK guidelines[36] suggest surgery within 48 hours; earlier surgery is associated with avoidance of unnecessary discomfort, earlier mobilisation and better functional outcomes. It also reduces the chances of complications associated with delay to surgery (urinary tract infections, venous thromboembolic disease and pressure sores). On the other hand people with neck of femur fractures often have multiple associated co-morbidities and potential medical causes for the fall, causing the fracture. In this case it is in their interests to be medically 'optimised' prior to theatre to reduce the chance of an adverse event under anaesthesia.

There are 2 recent meta-analyses [39, 40] and 2 recent literature reviews [41, 42] on the effect on mortality, morbidity and hospital LOS with delay to theatre for hip fracture. **There is little current consensus on exactly what constitutes a delay to surgery and if delay has an effect on mortality. There is consensus that earlier surgery reduces hospital LOS** [41, 42].

1. Simuovic et al 2001[41]: Literature review of delay to surgery for #NOF of more than 24 hours. Outcomes looked at: Reasons for Delay, Mortality, Post-Operative Complications, Duration of Hospital Stay and The Economic Burden of Surgical Delay.
    a. Common reasons for surgical delay were unavailability of the theatre or staff and stabilisation of the patient's pre-operative condition.
    b. Mortality – all studies underpowered and observational. "**Current evidence suggests while a delay of more than 24 hours may not unequivocally impact on mortality, there is no theoretical benefit for stable patients to wait for surgery**. In the case of medically unfit patients it is less clear."
    c. Post-operative complications: "surgical timing does not appear to have a significant effect on the number of post-operative complications". The studies looked at were retrospective, therefore associations but not causality could be drawn. "It is difficult to know if surgical delay adversely affects outcomes directly or if delay in surgery is simply a reflection of underlying co-morbidities that affect these complications".
    d. Duration of Hospital Stay: "Early surgical treatment is associated with a shorter hospital LOS".
    e. Economic Burden: "Prompt surgical intervention is not only good for the patient but it also reduces healthcare costs".

2. Simunovic et al 2010: Meta-Analysis and Systematic Review; effect of early surgery after hip fracture looking at <24 hours, < 48 hours, < 72 hours. Outcomes assessed were mortality and complications. 16 studies were included for analysis, 14,171. Mortality data available on 13,478.
    a. Mortality: RR assessed for all cause mortality in hospital, at 30 days, 3-6 months and one year. In pooled estimates in five studies (adjusted for confounding pre-operative factors) (n=4208), early surgery was associated with a 19% risk-reduction in all cause mortality (RR 0.81, CI 0.68-0.96, p=0.01), irrespective of the time of mortality. Unadjusted estimates of 16 studies also suggested that early surgery significantly reduced the risk of one-year mortality (RR0.55 CI 0.40-0.75, p=<0.001)
    b. Complications: Four studies reported on post-op complications for 5377 patients. Unadjusted data for confounders. This suggested early surgery reduced the risk of post-op pneumonia (RR 0.59 CI 0.37-0.93 p=0.02) and pressure sores (RR 0.48 CI 0.34-0.69, p<0.001). **Surgery conducted before 24-72 hours is associated with lower mortality**.

3. Shiga et al: Meta-Analysis and Systematic Review, effect of early surgery after hip fracture looking at <48 hours. Outcomes reported were 30 day mortality, one year mortality. 16 studies were included for analysis, 257,367 patients. "When a cut-off of 48 hours from time of admission was used to define operative delay, the pooled OR for 30 day mortality was 1.41 (CI 1.29-1.54, p<0.001) and for one-year mortality pooled OR 1.32 (CI 1.21-1.43, p<0.001)". **Operative delay beyond 48 hours may increase odds of short-term and long-term mortality.**

4. As regards ED access block and hip fractures: Richardson et al[43] found that the more inpatient borders there were in a hospital ED, the longer the wait for surgery for those with hip fractures.
   "In terms of patient care, fractured neck of femur is not a condition that improves with conservative management: it is clearly in the interests of hospital function to start the postoperative care as soon as possible".

Current time-based guidelines from Presentation to Surgery are:

- Canada: CIHI[44] Health Indicator is "Waiting Time for Inpatient Hip Fracture Surgery", **<48 hours and < 72 hours**
- UK: BOA +BGS[36] and SIGN Guideline on Hip Fractures[37] (DOH) **< 48 hours**. The SIGN guideline also advocates that the volume of evidence for reduction of mortality if operating within 48 hours is low (as mentioned above) and "there is no consistent evidence of an improvement in mortality from early surgery for hip" It advocates operating within 24 "safe" hours of "medical stabilisation".

- New Zealand Guidelines Group 2003[38]: Recommend "Early operation (within 24 hours) for people aged 65 years and over with hip fracture is associated with shorter hospital stay and decreased mortality/morbidity".
- The College of Emergency Medicine (UK) uses Time to X-Ray (<60 minutes) and Time to Admission (<4 hours) as quality of care indicators for Fracture neck of Femur in ED's.

"The true mortality as a result of the hip fracture or complications from related treatment is unknown, since it is often impossible to determine the extent of contribution from hip fracture to the eventual death"[42].

Time to Surgery for #NOF:

- The time interval between arriving at hospital and first skin incision in theatre (operation start time) for patients who have a fractured Neck of Femur. Data collection will also be included for Time to Analgesia.
- The time periods used will be < 24 hours, < 48 hours and < 72 hours.

Eligibility screening (for inclusion into study):

Fracture Neck of Femur

• No	
• Yes	X
• Not Recorded	
• Not Available	

Surgery

• No	
• Yes	X
• Not Recorded	
• Not Available	

10.2.1 Diagnostic X-Ray Location

Definition	Those patients who had a definitive diagnosis of #NOF from an X-ray that was done in ED (as opposed to in the Community)
Layout	N (number: 1 Character)
Codeset (If Applicable)	0 = X-Ray in ED<br>1 = X-Ray in Community prior to ED<br>2 = Not Recorded<br>3 = Not Available
Reported For	Includes: All Patients with #NOF in ED<br>Excludes: X-Ray in Community prior to ED
Description	The time recorded on the first X-Ray taken, which is diagnostic of a fractured femoral neck.
Numerator (If Applicable)	n/a
Denominator (If Applicable)	n/a
Expressed As	Categorical

Study Sites

10.2.2 Diagnostic X-Ray Time

Definition	Time of X-Ray giving definitive diagnosis of #NOF
Layout	DD/MM/CCYY HH:MM:SS (Date and Time: 18 characters separated by a space between year and hour values)
Codeset (If Applicable)	n/a
Reported For	Includes: All Patients with #NOF<br>Excludes:
Description	The time recorded on the fist X-Ray taken that is diagnostic of a fractured femoral neck.
Numerator (If Applicable)	n/a
Denominator (If Applicable)	n/a
Expressed As	Numeric (Time)

Study Sites

10.2.3 # NOF Surgery Start Time

Definition	Time of Surgery Start
Layout	DD/MM/CCYY HH:MM:SS (Date and Time: 18 characters separated by a space between year and hour values)
Codeset (If Applicable)	n/a
Reported For	Includes: All patients in OR for #NOF<br>Excludes:
Description	Usually recorded electronically (Theatre Manager – PIMS) or documented in patients notes in the operating theatre. Denotes the time when first skin incision made.
Numerator (If Applicable)	n/a
Denominator (If Applicable)	n/a
Expressed As	Numeric (Time)

Study Sites

10.3 Time to Surgery for Appendectomy in Appendicitis

A brief literature review of adverse outcome associated with delay to appendectomy in appendicitis was undertaken. There were little in the way of systematic reviews or meta-analyses. However several studies were of interest.

1) Von Titte et al 1995[45] – small retrospective analysis concentrated on those patients over a 4 year period who had a delay to operation of over 72 hours. Of these 40 patients: mean hospital LOS 9 days, incidence of perforation was 90 %(!) and major complications in 60%. Delay to diagnosis and treatment was accounted for by the physician in 62.5% cases and patients in 27.5% cases.

2) Whyte et al 2007[46] - Another small prospective study in children this time – 58 patients with CT proven appendicitis were treated according to a non-operative protocol. These patients were 'less ill' – none developing septic shock or being admitted to ICU. 62% responded and 32% failed (who then went on to operative management) – the failure group had a mean hospital LOS of 17 v 9 days and a 46% complication rate as compared to a 0% complication rate. This suggested nearly half of those who fail non-operative treatment have complications and therefore it is clearly important to make an early decision regarding appendectomy.

3) Ingraham et al 2008[47] – Examined the effect of delay from surgical admission to induction of anaesthesia on outcomes after appendectomy (30 day overall morbidity). Large (32,782 cases) retrospective cohort with principal exposure being time to operation. 75% had surgery within 6 hours, 155 at 2-12 hours and 9.8% more than 12 hours. Length of post-op stay was longer (statistically significant) in the >12 hour group of half a day. They found that delay to surgery does not adversely affect 30 day outcomes – however they had 3/4 of their cases operated on within 6 hours, which may skew the outcomes. The discussion posits treating acute appendicitis urgently rather than emergently (within 12-24 hours rather than in less than 6 hours) as the outcomes are unlikely to be different.

4) Omundsen et al (New Zealand)[48]– retrospective review of 345 histologically proven appendicitis (of 480) appendectomies. Mean wait time from admission to operation was 14.5 hours. 21% normal appendectomy rate (18% in <24 hour group and 35% in > 24 hour group). They found no difference in the complication rate or time to discharge among patients with pathologically confirmed appendicitis undergoing appendectomy within 12 hours of admission (58%) vs. 12 through 24 hours (30%) but observed higher rates of complications ($p = 0.01$) and increased postoperative lengths of stay ($p = <0.0001$) among those having surgery after 24 hours of admission (12%) compared with less than 24 hours after admission. The complications in the delayed group were more likely to be cardiovascular ($p = 0.02$) and pulmonary. Again small number in the delayed group.

5) Ditillo et al[49] retrospectively reviewed 1081 patients with appendicitis (pathological diagnosis). The risk of advanced pathology increased with wait to surgery ($p < 0.001$). The odds for progressive pathology was 13 times higher for the total interval >71 hours group (OR 13) compared with total interval <12 hours group (OR 1)

Quoting Ditillo et al "In adult patients with acute appendicitis, the risk of developing advanced pathology and postoperative complications increases with time; therefore, delayed appendectomy is unsafe. As delays in seeking medical help are difficult to control, prompt appendectomy is mandatory"

Even 100 years ago in the Lancet the appeal was made to not delay operation in appendicitis (albeit they were talking about days as opposed to hours!): Owen (Lancet, 1913)"Appendicitis: A Plea for Immediate Operation"[50]. This paper was read before the medical society of London Feb 10th 1913 to open a debate upon the Early Operation for Appendicitis.

For the purposes of this study 24 hours seems to be a cut off where operating after this time is associated with a higher rate of complications and increased length of hospital stay – therefore we shall use 24 hours as a cut off for analysis. Data collection will also be included for Time to Analgesia.

Time to Surgery for Appendectomy:

- The time interval between arriving at hospital and first skin incision in theatre (operation start time) for patients who have appendicitis (either clinically or radiologically proven).
- Less than 24 hours for time to Appendectomy

Eligibility Criteria:

Appendectomy

• No	
• Yes	X
• Not Recorded	
• Not Available	

Appendix Histology

• Appendicitis	X
• Normal Appendix	
• No Surgical Procedure	
• Other	
• Not Recorded	
• Not Available	

10.3.1 Serum WCC

Definition	The absolute serum white cell count from first blood tests done in ED (X10E+9/L) if taken
Layout	NNN.NN (Number: 6 Characters)
Codeset (If Applicable)	
Reported For	Includes: All Patients with Appendicitis<br>Excludes:
Description	
Numerator (If Applicable)	n/a
Denominator (If Applicable)	n/a
Expressed As	Numeric

Study Sites

10.3.2 Serum CRP

Definition	The C-Reactive Protein result from first blood tests done in ED (mg/L) if taken
Layout	NNN.NN (Number: 6 Characters)
Codeset (If Applicable)	
Reported For	Includes: All Patients with Appendicitis<br>Excludes:
Description	
Numerator (If Applicable)	n/a
Denominator (If Applicable)	n/a
Expressed As	Numeric

Study Sites

10.3.3 Scan Type Pre-Op

Definition	Patients with appendicitis who do, or do not undergo a CT Scan for a diagnosis of Appendicitis pre-operatively
Layout	N (Number: 1 Character)
Codeset (If Applicable)	0 = No Scan<br>1 = USS Abdomen<br>2 = CT Abdomen<br>3 = Other Scan Type Abdomen<br>4 = Not Recorded<br>5 = Not Available
Reported For	Includes: All Patients with Appendicitis<br>Excludes:
Description	
Numerator (If Applicable)	n/a
Denominator (If Applicable)	n/a
Expressed As	Categorical

Study Sites

10.3.4 Scan Time

Definition	Time of Abdominal Scan for Appendicitis
Layout	DD/MM/CCYY HH:MM:SS (Date and Time: 18 characters separated by a space between year and hour values)
Codeset (If Applicable)	n/a
Reported For	Includes: All Patients with Referred Appendicitis<br>Excludes:
Description	The time recorded on the first abdominal scan done for confirmation of diagnosis of Appendicitis (or other considered pathology – but proves appendicitis)
Numerator (If Applicable)	n/a
Denominator (If Applicable)	n/a
Expressed As	Numeric (Time)

Study Sites

10.3.5 Result Scan Pre-Op

Definition	The results of Pre-Op CT Scan (CT Scan pre-op = ‘1’)
Layout	N (Number: 1 Character)
Codeset (If Applicable)	0 = No Scan<br>1= Non-Diagnostic<br>2 = Confirms Appendicitis<br>3 = Confirms other Diagnosis<br>4 = Appendicitis AND other pathology<br>5 = Not Recorded<br>6 = Not Available
Reported For	Includes: All Patients with Appendicitis<br>Excludes:
Description	Patients with appendicitis who do undergo any abdominal scan for a diagnosis of Appendicitis pre-operatively, who have either a positive confirmation of appendicitis diagnosis, a non-diagnostic scan, or other cause of symptoms found on scan, that is not appendicitis.
Numerator (If Applicable)	n/a
Denominator (If Applicable)	n/a
Expressed As	Categorical

Study Sites

10.3.6 Surgery Start Time

Definition	Time of Surgery Start – time of skin incision
Layout	DD/MM/CCYY HH:MM:SS (Date and Time: 18 characters separated by a space between year and hour values)
Codeset (If Applicable)	n/a
Reported For	Includes: All patients in OR for Appendicitis<br>Excludes:
Description	Usually recorded electronically (Theatre Manager – PIMS) or documented in patients notes in the operating theatre. Denotes the time when first skin incision made.
Numerator (If Applicable)	n/a
Denominator (If Applicable)	n/a
Expressed As	Numeric (Time)

Study Sites

10.3.7 Appendix Appearance OR

Definition	The intra-operative appearance of the appendix
Layout	N (Number: 1 Character)
Codeset (If Applicable)	0 = Normal Appendix (no Appendiceal inflammation)<br>1 = Acute Appendicitis (Inflamed Appendix)<br>2 = Gangrenous Appendicitis<br>3 = Perforated Appendicitis<br>4 = Perforated AND gangrenous Appendicitis<br>5 = Appendicitis with Abscess<br>6 = Perforated Appendicitis with Abscess<br>7 = Other<br>8 = Not Recorded<br>9 = Not Available
Reported For	Includes: All Presentations to Hospital<br>Excludes:
Description	The surgeon's description of how the appendix looked intra-operatively. This can be used to compare against histology findings.
Numerator (If Applicable)	n/a
Denominator (If Applicable)	n/a
Expressed As	Categorical

Study Sites

10.3.8 Appendiceal Histology

Definition	The Histological Diagnosis of removed Appendices
Layout	N (Number: 1 Character)
Codeset (If Applicable)	0 = Normal Appendix (no Appendiceal inflammation)<br>1 = Acute Appendicitis (Inflamed Appendix)<br>2 = Acute Supperative Appendicitis<br>3 = Gangrenous Appendix<br>4 = Supperative Appendicitis with Perforation<br>5 = Perforated and Gangrenous Appendicitis<br>6 = Peri-Appendiceal Abscess<br>7 = Serosal Chronic Inflammation<br>8 = Parasitic Infestation Appendix with Appendicitis<br>9 = Parasitic Infestation Appendix without Appendicitis<br>10 = Other<br>11 = Not Recorded<br>12 = Not Available
Reported For	Includes: All Presentations to Hospital<br>Excludes:
Description	The pathologists report of Appendiceal histology.
Numerator (If Applicable)	n/a
Denominator (If Applicable)	n/a
Expressed As	Categorical

Study Sites

10.4 Time to Antibiotics in Severe Sepsis

The effects of severe sepsis are a combination of the effect of an infecting organism on the body and the body's response both to this effect and the organism itself. It makes sense that as severe sepsis and septic shock can be devastating with a high mortality, you need to address the initial cause (the organism), as well as the body's response. Intuitively the timelier this is, the better chance of recovery. However there is little consensus on what constitutes 'too late'.

Sepsis Definitions[51-53]: Developed from referenced papers – documented are the IHI parameters which are also derived from and follow Levy et al and Dellinger et al's (Surviving Sepsis Campaign) definitions.

1. **ADULTS**

Systemic Inflammatory Response Syndrome (SIRS) Definition: is the body's response to systemic activation of the innate immune response, regardless of the cause.

Sepsis Definition: Sepsis is an exaggeration of the body's normal response to infection. There must be **clinical evidence of infection and SIRS.**

Characterised by:

- History Suggestive of New Infection (in ED)
    - Pneumonia, Empyema
    - UTI
    - Acute Abdominal Infection
    - Meningitis
    - Skin / Soft Tissue Infection
    - Bone / Joint Infection
    - Wound Infection
    - Blood Stream Catheter Infection
    - Endocarditis
- <u>Plus</u> more than one of the following present AND SIRS new (in ED)
- General Variables (can be measured for this study in **BOLD)**
    - **Fever: Temp > 38.3**
    - **Hypothermia: Temp <36**
    - **Tachycardia: HR > 90**
    - **Tachypnoea: RR > 20 OR $PaCO_2$ <32mmHg**
    - **Acutely altered mental status (GCS <15)**
    - Significant oedema – positive fluid balance > 20ml/kg (won't be able to measure, don't capture weight)
    - **Hyperglycaemia (not diabetic) Glucose >7.7mmol/l in the absence of diabetes**
- Inflammatory variables
    - **WCC > 12,000**
    - **WCC <4,000**
    - **Normal WCC with >10% immature forms, segmented neutrophils**
    - Plasma CRP > 2 sd above normal value
    - Plasma Procalcitonin > 2 sd above the normal value

Severe Sepsis Definition:

- **Characterised by above PLUS evidence of End-Organ Dysfunction, hypoperfusion or hypotension**
- Any of the following organ dysfunction criteria at a site REMOTE from infection
- Organ Dysfunction Variables
    - Arterial Hypoxaemia **PaO2/FiO2 ratio <300**
    - **Creatinine > 176mmol/l** or 2.0mg/dL
    - Urine Output <45ml/hr for > 2 hours or <0.5ml/kg/hr for 2 hours (can't measure as won't have weight)
    - **Bilirubin > 35 umol/l (or 34.2 – Dellinger)** or 2.0mg/dL
    - **Platelet Count < 100,000**
    - **Coagulopathy (INR > 1.5, aPTT > 60 secs)**
- Tissue Perfusion Variables
    - **Lactate > 2 mmol/l**
- Haemodynamic variables
    - **SBP <90 or MAP < 70 or SBP decrease >40mmhg**

Septic Shock Definition: Characterised by the above PLUS shock non-responsive to adequate fluid resuscitation.

2. **Paediatric Population**

For the purposes of looking at Sepsis in this study we will separate the Paediatric population into clinically and physiologically meaningful age groups, as described by Goldstein et al[54] from the International Consensus Conference of Paediatric Sepsis:

Newborn: 0 days to 7 days
Neonate: 8 days to 30 days
Infant: 1 month (31 days) to 12 months
Toddler: 13 months to 5 years
School Age: 6 years to 12 years
Adolescent / Young Adult: 13 years to < 18 years
Adult: >= 18 years old

Paediatric sepsis has been touched on by Levy et al[51], however we will be using the guidelines developed specifically for the paediatric population by Goldstein et al[54]. The following definitions and tables on pages 132-134 of this document have all been copied and extracted from the Goldstein paper.

Consensus Definition of Infection in Children: A suspected or proven (by positive culture, tissue stain, or polymerase chain reaction test) infection caused by any pathogen OR a clinical syndrome associated with a high probability of infection.

Consensus Definition of SIRS in Children:

The presence of at least two of the following four criteria, **one of which must be abnormal temperature or leukocyte count:**

- Core temperature of _38.5°C or _36°C.
- Tachycardia, defined as a mean heart rate _2 SD above normal for age in the absence of external stimulus, chronic drugs, or painful stimuli; or otherwise unexplained persistent elevation over a 0.5- to 4-hr time period **OR for children <1 yr old: Bradycardia, defined as a mean heart rate <10th percentile for age in the absence of external vagal stimulus, _-blocker drugs, or congenital heart disease; or otherwise unexplained persistent depression over a 0.5-hr time period.**
- Mean respiratory rate _2 SD above normal for age or mechanical ventilation for an acute process not related to underlying neuromuscular disease or the receipt of general anaesthesia.
- Leukocyte count elevated or depressed for age (not secondary to chemotherapy-induced leucopoenia) or _10% immature neutrophils.

Table 3. Age-specific vital signs and laboratory variables (lower values for heart rate, leukocyte count, and systolic blood pressure are for the 5th and upper values for heart rate, respiration rate, or leukocyte count for the 95th percentile)

Age Group[a]	Heart Rate, Beats/Min[b,c]		Respiratory Rate, Breaths/Min[d]	Leukocyte Count, Leukocytes × 10^3/mm[3b,c]	Systolic Blood Pressure, mm Hg[b,c,e,f]
	Tachycardia	Bradycardia			
0 days to 1 wk	>180	<100	>50	>34	<65
1 wk to 1 mo	>180	<100	>40	>19.5 or <5	<75
1 mo to 1 yr	>180	<90	>34	>17.5 or <5	<100
2–5 yrs	>140	NA	>22	>15.5 or <6	<94
6–12 yrs	>130	NA	>18	>13.5 or <4.5	<105
13 to <18 yrs	>110	NA	>14	>11 or <4.5	<117

NA, not applicable.

Table taken from Goldstein et al[54]

Consensus Definition of Sepsis in Children:

SIRS in the presence of or as a result of, suspected, or proven infection.

Consensus Definition of Severe Sepsis in Children:

Sepsis plus one of the following: cardiovascular organ dysfunction OR acute respiratory distress syndrome OR two or more other organ dysfunctions.

Cardiovascular dysfunction: Despite administration of isotonic intravenous fluid bolus >=40 mL/kg in 1 hr

- Decrease in BP (hypotension) <5th percentile for age or systolic BP <2 SD below normal for age

OR

- Need for vasoactive drug to maintain BP in normal range (dopamine >5 microg/kg/min or dobutamine, epinephrine, or norepinephrine at any dose)

OR Two of the following

- Unexplained metabolic acidosis: base deficit >-5.0 mEq/L
- Increased arterial lactate >2 times upper limit of normal (upper limit normal 1.6, therefore high lactate is >=3.2)
- Oliguria: urine output _0.5 mL/kg/hr (not collected)
- Prolonged capillary refill: >5 secs
- Core to peripheral temperature gap >3°C (not collected)

Respiratory

- PaO2/FIO2 <300 in absence of cyanotic heart disease or pre-existing lung disease

OR

- PaCO2 >65 mmHg or 20 mmHg over baseline PaCO2

OR

- Proven need or >50% FIO2 to maintain saturation >=92%

OR

- Need for non-elective invasive or non-invasive mechanical ventilation

Neurologic

- Glasgow Coma Score <=11

OR

- Acute change in mental status with a decrease in Glasgow Coma Score _3 points from abnormal baseline

Hematologic

- Platelet count <80,000/mm3 or a decline of 50% in platelet count from highest value recorded over the past 3 days (for chronic haematology/oncology patients)

OR

- International normalized ratio >2

Renal

- Serum Creatinine >=2 times upper limit of normal for age or 2-fold increase in baseline Creatinine.
    - 0 – 30 days; >= 120 umol/l
    - 31 days to 24 months: >= 100 umol/l
    - 24 months to 4 years: >= 120 umol/l
    - 4 to 6 years: >=130 umol/l
    - 6 to 10 years: >=140 umol/l
    - 10 to 15 years: >= 160 umol/l
    - >15 years: >=176 umol/l

Hepatic

- Total Bilirubin >4 mg/dL or 70 umol/l (not applicable for newborn)

OR

- ALT 2 times upper limit of normal for age (0-2 months >= 156 u/L and > 2 months >=72 u/L)

(Acute respiratory distress syndrome must include a PaO2/FIO2 ratio <=200 mm Hg, bilateral infiltrates, acute onset, and no evidence of left heart failure (Refs. 58 and 59). Acute lung injury is defined identically except the PaO2/FIO2 ratio must be <=300 mm Hg)

Consensus Definition of Septic Shock in Children:

Sepsis and cardiovascular organ dysfunction above.

Established Literature:

EGDT therapy – Rivers 2001: This landmark trial compared "Early Goal Directed Therapy" (n=130) for sepsis against standard treatment (n=133). The outcomes of this trial were the forerunner for the surviving sepsis campaign. In-Hospital mortality was the primary efficacy endpoint. 'Administered treatments' was one of the secondary endpoints. 92.4% of patients on standard therapy received antibiotics within the first 6 hours of severe sepsis being recognised, compared with only 86.8% in the EGDT group. These were adequate in 94% and 96% of cases respectively. Other than this mention, no other discussion was had regarding time to antibiotics. One important point to make is the unusually high mortality in the "standard care" group.

Bochud 2004: in his evidence based review stated that "**antibiotic therapy should be started within the first hour of recognition of severe sepsis** (after appropriate cultures)." He recommends this on Grade E evidence, but does not present the evidence explicitly in the paper.

When the Surviving Sepsis Campaign (http://www.survivingsepsis.org) was launched, it recommended care be delivered in 'bundles' stratified to the first 6 hours and the following 24 hours. The aim is to reduce mortality and prevent deterioration from sepsis by earlier recognition and earlier management, driven by a guidelines-based package of care. **It was recommended antibiotics be given within the first hour of arrival to ED** in the 2001 guidelines.

The Surviving Sepsis Campaign now recommends "that intravenous antibiotic therapy be started **as early as possible and within the first hour of recognition of septic shock** (GRADE 1B) and severe sepsis without septic shock (1D). Appropriate cultures should be obtained before initiating antibiotic therapy, but should not prevent prompt administration of antimicrobial therapy (1D)". They suggest that **antibiotics be given within 3 hours for ED patients with severe sepsis** and **within 1 hour within recognition of septic shock** or for ED patients expected to go to ICU.

Kumar 2006 – 2731 patients. Retrospective cohort determining: Impact on mortality of delays in initiation of effective antimicrobial therapy, from initial onset of recurrent or persistent hypotension. 2154 (78.9%) of patients got antibiotics after onset of persistent hypotension. A relationship between this delay and in-hospital mortality noted: OR 1.110. Administration of antibiotics in the first hour of documented hypotension was associated with a survival rate of 79.9%. **By the second hour after onset of persistent, recurrent hypotension, the in-hospital mortality rate significantly increased relative to that in the first hour (OR 1.67)**. In a multivariate analysis (including APACHE II Score) time to initiation of effective antimicrobial therapy was single strongest predictor of outcome. They surmised that "**duration of hypotension before initiation of effective antimicrobial therapy is the critical determinant of survival in septic shock"**.

Gaieski 2010: Retrospective Analysis 261 patients, single centre study. They studied the association between time to antibiotic admin and survival, in patients with severe sepsis or septic shock undergoing EGDT. No association between antibiotic and survival when assessed at different hourly cut-offs. When time from triage to **appropriate** antibiotics was analysed there was a significant reduction in mortality at less than 1 hour (OR 0.3, p 0.02) and also in time from qualification for EGDT to **appropriate** antibiotics at less than 1 hour (OR 0.5, p0.03)

The priority given to antibiotic delivery remains unclear from the literature. The length of time someone is hypotensive (in septic shock) appears to be more of a survival determinant than time to antibiotic. And the appropriateness of the antibiotics is important too – which is difficult in an ED when the diagnosis may be unclear and 'best-guess' antimicrobial therapy is used.

In America time to first antibiotic dose for community acquired pneumonia has become a pay-for-performance quality indicator (antibiotics to be given in less than 4 hours from arrival to the ED). This has led to an increase of misdiagnosis and antibiotic overuse. A recent review by Pines (AAEM 2009) led the American Academy of Emergency medicine to assign a class C recommendation to the measurement of this quality indicator and suggested it be withdrawn as it was not appropriate for the ED, due to conflicting evidence and unintended outcomes.

The College of Emergency Medicine UK Clinical Standards Guidelines[55] suggests that in the management of severe sepsis and septic shock:
"There should be documented evidence that antibiotics were administered:
• In 50% of cases within 1 hour of arrival
• In 90% of cases within 2 hours of arrival
• In 100% cases prior to leaving the ED"

Time to Antibiotics for Severe Infections: The time taken to give someone antibiotics for their severe infection from first presentation to the ED.

- **For SSED NRP study purposes we will be looking at clinically relevant time to antibiotics in:**
- **Severe Sepsis within 1, 2 and 3 hours.**
- **Septic Shock (or patients going to ICU) within 1 hour.**

Best Guess Antibiotics Therapy (adapted from ADHB clinical guidelines and Royal Children's Hospital Melbourne / Starship Children's Hospital clinical guidelines).

Adult:

**Abdomen**	**Peritonitis**	**Metronidazole + Gentamicin**
		**Metronidazole + Cefuroxime**
		**Metronidazole + Amoxycillin + Gentamicin**
		**Cefoxitin**
		**Augmentin**
	**Gallbladder**	**Augmentin + Gentamicin (Cefoxitin + Amoxycillin)**
	**Liver**	**Metronidazole + Gentamicin (+ Amoxycillin)**
		**Metronidazole + Cefuroxime**
		**Cefoxitin**
**Blood**	**Bacteraemia**	**Cefuroxime + Gentamicin (Aztreonam + Fluclox)**
	**Normal**	**Cefoxitin + Gentamicin**
	**Immunocompromised**	**Cefuroxime + Gentamicin (Ceftriaxone + Gent)**
	**Immunocompromised**	**Augmentin + Gentamicin (Aztreonam + Fluclox)**
	**Neutropoenic**	**Cefuroxime + Gentamicin (Ceftriaxone + Gent)**
		**Cefepime + Gentamicin**
	**Typhoid**	**Ciprofloxacin (3rd gen Ceph, Amoxycillin, Co-Trimoxazole)**
**Bone**	**Osteomyelitis**	**Flucloxacillin**
		**Cephazolin or Benzylpenicillin or Clindamycin**
	**MRSA**	**Vancomycin**
	**MRSA**	**Clindamycin or Co-Trimoxazole or Fusidic Acid**
**Skin**	**Mastitis**	**Flucloxacillin (Cephazolin)**
	**Mastitis NOT postpartum**	**Augmentin (Flucloxacillin and Metronidazole)**
	**Bites**	**Augmentin (Cefoxitin or Clindamycin)**
	**Cellulitis**	**Flucloxacillin (Cephazolin or Macrolide)**
		**BenPen (Flucloxacillin or Cephazolin or Macrolide)**

	Diabetic Foot<br><br><br><br>Impetigo	Augmentin or Flucloxacillin (Cephazolin or Clindamycin)<br><br>Cefuroxime + Metronidazole (Cefoxitin / Gentamicin + Metronidazole)<br><br>Flucloxacillin (BenPen / Cephazolin / Macrolide)
CNS	Brain Abscess<br><br>Mastoiditis<br><br>Trauma / Post-op<br><br>Meningitis	Amoxycillin + Metronidazole (BenPen and Met)<br><br>Ceftriaxone + Metronidazole<br><br>Ceftriaxone + Metronidazole + Flucloxacillin<br><br>BenPen (Ceftriaxone)<br><br>BenPen + Vancomycin (if increased risk *S. Pneumoniae*)<br><br>Ceftriaxone + Ben Pen (*Listeria*)
Ear	Mastoiditis	Augmentin (Cefuroxime)
Joint	Arthritis	Flucloxacillin (Cephazolin / BenPen / Clindamycin)<br><br>BenPen<br><br>Ceftriaxone then Augmentin
UroGenital	PID<br><br><br>Cystitis<br><br>Perinephric Abscess	Ciprofloxacin / Ceftriaxone and Doxycycline (Cefoxitin and Doxycycline) or erythromycin and Augmentin<br><br>Trimethoprim / Nitrofurantoin / Co-Trimoxazole / Cefaclor / Augmentin (Norfloxacin / Doxycycline)<br><br>Flucloxacillin (*staph bacteraemia*)<br><br>Gentamicin (then to cystitis as above) (Cefuroxime / Aztreonam)<br><br>Gentamicin / Cefuroxime / Aztreonem
Cardiac	Endocarditis	Flucloxacillin + Gentamicin (Cephazolin + Gentamicin)<br><br>BenPen (Ceftriaxone)<br><br>Ben Pen + Gentamicin (Amoxycillin + Gent or Vancomycin + Gent)
Mouth / Sinus	Vincent's Angina<br><br>Dental Sepsis<br><br>Candidiasis	BenPen (Metronidazole)<br><br>Metronidazole (BenPen)<br><br>Topical nystatin (or Amphotericin)

	Epiglottitis<br><br>Sinusitis	Oral ketoconazole (immunocompromised)<br><br>Augmentin (Cefuroxime)<br><br>Amoxycillin / Doxycycline / Augmentin (Macrolide / Co-Trimoxazole + Metronidazole if chronic)
Respiratory	Chronic Bronchitis<br><br>Pneumonia: Atypical<br><br>Lobar<br><br>Broncho-pneumonia	Amoxycillin / Doxycycline / Augmentin / Co-Trimoxazole (Cefuroxime / Roxithromycin)<br><br>Roxithromycin / Erythromycin / Doxycycline<br><br>BenPen (Roxithromycin / Erythromycin / Amoxycillin)<br><br>Augmentin + Roxithromycin / Cefuroxime + Roxithromycin<br><br>Augmentin + Roxithromycin / Cefuroxime + Roxithromycin

Paediatrics:

Central Nervous System / Eye

Encephalitis

Acyclovir *IV*

Meningitis (suspected or proven)

Cefotaxime *IV*

If <2 mths
add Benzylpenicillin *IV* ***and*** Gentamicin *IV* ***and if possibility of penicillin resistant pneumococcus, add*** Vancomycin *IV*

Orbital cellulitis

Flucloxacillin *IV* and Cefotaxime *IV*

Periorbital cellulitis

Mild: Augmentin *ORAL*

Moderate: Flucloxacillin *IV*

Severe, or under 5yr + not Hib immunised: as for orbital cellulitis.

Gastrointestinal Tract

Peritonitis

Benzylpenicillin *IV* or Amoxycillin *IV* ***and*** Gentamicin *IV* and Metronidazole *IV*

Giardiasis

Metronidazole *ORAL*

Genitourinary Tract

Urinary Tract Infection

Sick, or under 6 mths, or acute pyelonephritis:
Benzylpenicillin *IV:* 50 mg/kg (max 3 g) 6 hrly and Gentamicin *IV:* 7.5 (6 if >10 yr) mg/kg (max 360 mg) daily or

Under 3 months – CNS sepsis NOT excluded = Amoxycillin AND Cefotaxime

Under 3 months – CNS sepsis excluded = Amoxycillin AND Gentamicin

Over 3 months – Gentamicin OR Cefuroxime

Over 6 mths + not sick: (in order of preference) Augmentin *ORAL* or Co-Trimoxazole *ORAL or* Cephalexin *ORAL*

Respiratory

Tonsillitis

Consider no antibiotics ***or*** Penicillin V, *ORAL*

Otitis Media

Consider no antibiotics for 48 hrs (if over 2 yrs) ***or*** Amoxycillin *ORAL*

Pertussis

Clarithromycin *ORAL*

Pneumonia

Mild: Amoxycillin *ORAL*

Moderate: Benzylpenicillin *IV or*

Under 3 = Cefotaxime *IV* AND Amoxycillin *IV*

Over 3 = Amoxycillin *IV*

Severe, or pneumatocoele or large pleural effusion: Flucloxacillin *IV* ***and*** Gentamicin *IV OR*

Under 3 = Cefotaxime *IV* and Amoxycillin *IV*

Over 3 and not immunised = Augmentin *IV* OR Cefuroxime *IV*

Over 5 yr-consider *Mycoplasma*; ***add*** Erythromycin *ORAL* or Roxithromycin *ORAL*

Skin / Soft Tissue / Bone

Adenitis

Flucloxacillin *IV*

Bites (animal /human)

Augmentin *ORAL*

Severe: Cefotaxime *IV* ***and*** Metronidazole *IV, ORAL* OR Augmentin *IV*

Cellulitis

Mild: Penicillin V, *ORAL or if bite or injury not responding, substitute* Flucloxacillin *ORAL*

Moderate/Severe: Benzylpenicillin *IV* or *if bite/injury or not responding substitute* Flucloxacillin *IV*

Facial + under 5 yr + not Hib immunised: As for <u>orbital cellulitis</u>

Erythromycin if penicillin allergic

Impetigo

Mupirocin 2% ointment if localised ***or*** Flucloxacillin *ORAL*

Osteomyelitis/Septic arthritis

Flucloxacillin *IV*

Under 5 yr + not Hib immunised ***add*** Cefotaxime *IV*

Bacteraemia (i.e. sick child)

-with normal CSF
Flucloxacillin *IV* ***and*** Gentamicin *IV*

-with unknown CSF
Flucloxacillin *IV* ***and*** Cefotaxime IV

Or Cefotaxime *IV* and Amoxycillin *IV*

Eligibility Criteria and Sepsis Severity Stratification:

Temperature (1st Recorded ED) NN.NN (degrees C)

Heart Rate NNN (1st Recorded ED)

Systolic Blood Pressure NNN (mmHg) (1st Recorded ED)

Diastolic Blood Pressure NNN (mmHg) (1st Recorded ED)

Systolic Blood Pressure NNN (mmHg) (Lowest Recorded ED – 1st 6 hours)

Diastolic Blood Pressure NNN (mmHg) (Corresponding to above systolic)

Respiratory Rate NNN (1st Recorded ED)

Oxygen Saturations NNN (%) (1st Recorded ED)

Fraction Inspired Oxygen N.NN (1st Recorded ED) **See FiO2 Table in SS**

PaO2 (partial pressure oxygen in **arterial** blood) NNN.NN (mmHg) (1st Recorded ED)

PaCO2 (partial pressure carbon dioxide in **arterial** blood) NNN.NN (mmHg) (1st Recorded ED)

Conversion factor (kPa to mmHg): ***mmHg = 7.5 x kPa***

Glasgow Coma Scale NN (between 3 and 15)

Serum Creatinine NNNN (mmol/l)

Absolute serum White Cell Count NNN.NN (X10E9/L)

Segmented Neutrophils NNN.NN (X10E9/L)

Platelets NNNN (X10E9/L)

Glucose NNN.NN (mmol/l)

Diabetes

• Yes	
• No	
• Not Recorded	
• Not Available	

Lactate NNN.NN (mmol/l)

INR (International Normalised Ratio) NNN

Warfarin

• Yes	
• No	
• Not Recorded	
• Not Available	

aPTT (activated partial thromboplastin time) NNN (seconds)

Bilirubin NNN (umol/l)

Chest X-Ray

• No CXR indicated	
• Normal CXR	
• Unilateral pulmonary infiltrates	
• Bilateral pulmonary infiltrates	
• Empyema	
• Pulmonary Abscess	
• Abnormal – Other (non-infective) pathology	
• Not Available	
• Not Recorded	

Paeds Only: (plus previous)

Weight NNN (Kg)

Capillary Refill Time NNN (seconds)

Base Deficit NNN (mmol/l)

ALT NNNN (micromol/L)

Inotropic Support in ED

• Yes (any inotrope in ED)	
• No	
• Contraindicated	
• Medical Decision “No”	
• Not Recorded	
• Not Available	

Ventilation ED

• Yes _ Invasive	
• Yes – Non-Invasive	
• No	
• Contraindicated	
• Medical Decision “No”	
• Not Recorded	
• Not Available	

10.4.1 Primary Site of Infection

Definition	The site of infection causing symptoms
Layout	NN (Number: 2 Characters)
Codeset (If Applicable)	0 = No site of infection (other cause for symptoms)<br>1 = Blood<br>2 = Urinary Tract<br>3 = Respiratory Tract<br>4 = Abdominal Cavity<br>5 = Central Nervous System<br>6 = Bone<br>7 = Cardiac<br>8 = Genital<br>9 = Implant or Catheter related infection<br>10 = Sinus cavity<br>11 = Skin<br>12 = Neutropoenic Sepsis<br>13 = Multiple Sites of Infection<br>14 = Unknown<br>15 = Not Recorded<br>16 = Not Available
Reported For	Includes: All Presentations<br>Excludes:
Description	The site of infection as proposed by the ED clinician during the patients' ED stay. Sometimes this diagnosis is just postulated in the ED, as time may be needed before a source is found. We will be using site of infection as documented in the clinical notes from ED or if not documented there, the admission note from the admitting team.
Numerator (If Applicable)	n/a
Denominator (If Applicable)	n/a
Expressed As	Categorical

Study Sites

10.4.2 Ix: Peripheral Blood Cultures

Definition	Blood Cultures taken in the ED
Layout	NN (Number: 2 Characters)
Codeset (If Applicable)	0 = Indicated - Not done in ED<br>1 = Indicated - Growth (POSITIVE)<br>2 = Indicated - Growth (CONTAMINATED)<br>3 = Indicated - No growth (NEGATIVE)<br>4 = Not Indicated - not done in ED (temp <=38 degrees)<br>5 = Not Indicated - Growth (POSITIVE)<br>6 = Not indicated - Growth (CONTAMINATED)<br>7 = Not Indicated - No growth (NEGATIVE)<br>8 = Not Available<br>9 = Not Recorded
Reported For	Includes: All Presentations<br>Excludes:
Description	Inoculation of sealed bottled culture media when considering sepsis – or evidence of bacterial invasion of the blood stream.
Numerator (If Applicable)	n/a
Denominator (If Applicable)	n/a
Expressed As	Categorical

Study Sites

10.4.3 Number of Peripheral Blood Cultures

Definition	Blood Cultures taken in the ED
Layout	NN (Number: 2 Characters)
Codeset (If Applicable)	0 = None - BCs Not Done<br>1 = None - BCs Not Indicated<br>2 = One Set - BCs Indicated<br>3 = One Set - BCs Not Indicated<br>4 = Two Sets - BCs Indicated<br>4 = Two Sets -BCs Not Indicated<br>5 = Three or more sets - BCs Indicated<br>6 = Three or more sets - BCs Not Indicated<br>7 = Not Available<br>8 = Not Recorded
Reported For	Includes: All Presentations<br>Excludes:
Description	The number of sets (of 2 bottles in adult cases and 1 bottle in paediatric cases) of blood cultures taken in the Emergency Department.
Numerator (If Applicable)	n/a
Denominator (If Applicable)	n/a
Expressed As	Categorical

Study Sites

10.4.4 Ix: Urine Culture

Definition	Urine Cultures taken in the ED
Layout	NN (Number: 2 Characters)
Codeset (If Applicable)	0 = Indicated - Not done in ED<br>1 = Indicated - Growth (POSITIVE)<br>2 = Indicated - Growth (CONTAMINATED)<br>3 = Indicated - No Growth (NEGATIVE)<br>4 = Not indicated - Not done<br>5 = Not indicated - Growth (POSITIVE)<br>6 = Not indicated - Growth (CONTAMINATED)<br>7 = Not indicated - No growth (NEGATIVE)<br>8 = Not Available<br>9 = Not Recorded
Reported For	Includes: All Presentations<br>Excludes:
Description	Inoculation of culture media when considering urinary tract infection.
Numerator (If Applicable)	n/a
Denominator (If Applicable)	n/a
Expressed As	Categorical

Study Sites

10.4.5 Ix: Pus Swab

Definition	Pus Swab taken for cultures in the ED
Layout	NN (Number: 2 Characters)
Codeset (If Applicable)	0 = Indicated - Not done in ED<br>1 = Indicated - Growth (POSITIVE)<br>2 = Indicated - Growth (CONTAMINATED)<br>3 = Indicated - No Growth (NEGATIVE)<br>4 = Not indicated - Not done<br>5 = Not indicated - Growth (POSITIVE)<br>6 = Not indicated - Growth (CONTAMINATED)<br>7 = Not indicated - No growth (NEGATIVE)<br>8 = Not Available<br>9 = Not Recorded
Reported For	Includes: All Presentations<br>Excludes:
Description	Inoculation of culture media taken with a sterile swab when considering infection causing pus formation – for example from the abdomen, pleural cavity, genital tract, a joint or the skin.
Numerator (If Applicable)	n/a
Denominator (If Applicable)	n/a
Expressed As	Categorical

Study Sites

10.4.6 Ix: CSF Specimen

Definition	CSF (cerebrospinal fluid) specimen taken for cultures in the ED
Layout	NN (Number: 2 Characters)
Codeset (If Applicable)	0 = Indicated - Not done in ED<br>1 = Indicated - Growth (POSITIVE)<br>2 = Indicated - Growth (CONTAMINATED)<br>3 = Indicated - No Growth (NEGATIVE)<br>4 = Not indicated - Not done<br>5 = Not indicated - Growth (POSITIVE)<br>6 = Not indicated - Growth (CONTAMINATED)<br>7 = Not indicated - No growth (NEGATIVE)<br>8 = Not Available<br>9 = Not Recorded
Reported For	Includes: All Presentations<br>Excludes:
Description	Inoculation of culture media with a sample of cerebrospinal fluid – withdrawn during a lumbar puncture in the clinical suspicion of meningitis or encephalitis.
Numerator (If Applicable)	n/a
Denominator (If Applicable)	n/a
Expressed As	Categorical

Study Sites

10.4.7 Ix: Catheter Tip Culture

Definition	Catheter Tip sent away for cultures done in the ED
Layout	NN (Number: 2 Characters)
Codeset (If Applicable)	0 = Indicated - Not done in ED<br>1 = Indicated - Growth (POSITIVE)<br>2 = Indicated - Growth (CONTAMINATED)<br>3 = Indicated - No Growth (NEGATIVE)<br>4 = Not indicated - Not done<br>5 = Not indicated - Growth (POSITIVE)<br>6 = Not indicated - Growth (CONTAMINATED)<br>7 = Not indicated - No growth (NEGATIVE)<br>8 = Not Available<br>9 = Not Recorded
Reported For	Includes: All Presentations<br>Excludes:
Description	Inoculation of culture media with the tip of an indwelling catheter or other prosthesis when considering this as the source of the infection. It should usually match cultures from other sites (such as blood) if it is the cause of the infection.
Numerator (If Applicable)	n/a
Denominator (If Applicable)	n/a
Expressed As	Categorical

Study Sites

10.4.8 Ix: Sputum Culture

Definition	Expectorated Sputum taken for culture in the ED
Layout	NN (Number: 2 Characters)
Codeset (If Applicable)	0 = Indicated - Not done in ED<br>1 = Indicated - Growth (POSITIVE)<br>2 = Indicated - Growth (CONTAMINATED)<br>3 = Indicated - No Growth (NEGATIVE)<br>4 = Not indicated - Not done<br>5 = Not indicated - Growth (POSITIVE)<br>6 = Not indicated - Growth (CONTAMINATED)<br>7 = Not indicated - No growth (NEGATIVE)<br>8 = Not Available<br>9 = Not Recorded
Reported For	Includes: All Presentations<br>Excludes:
Description	Inoculation of culture media taken by sputum expectorated from the respiratory tract when considering infection causing respiratory infection– for example pneumonia.
Numerator (If Applicable)	n/a
Denominator (If Applicable)	n/a
Expressed As	Categorical

Study Sites

10.4.9 Ix: Faecal Culture

Definition	Faecal sample taken for culture in the ED
Layout	NN (Number: 2 Characters)
Codeset (If Applicable)	0 = Indicated - Not done in ED<br>1 = Indicated - Growth (POSITIVE)<br>2 = Indicated - Growth (CONTAMINATED)<br>3 = Indicated - No Growth (NEGATIVE)<br>4 = Not indicated - Not done<br>5 = Not indicated - Growth (POSITIVE)<br>6 = Not indicated - Growth (CONTAMINATED)<br>7 = Not indicated - No growth (NEGATIVE)<br>8 = Not Available<br>9 = Not Recorded
Reported For	Includes: All Presentations<br>Excludes:
Description	Inoculation of culture media of faecal material when considering gastrointestinal tract infection.
Numerator (If Applicable)	n/a
Denominator (If Applicable)	n/a
Expressed As	Categorical

Study Sites

10.4.10 Ix: Aspirated Pus Culture

Definition	Aspirated Pus taken for culture in the ED
Layout	NN (Number: 2 Characters)
Codeset (If Applicable)	0 = Indicated - Not done in ED<br>1 = Indicated - Growth (POSITIVE)<br>2 = Indicated - Growth (CONTAMINATED)<br>3 = Indicated - No Growth (NEGATIVE)<br>4 = Not indicated - Not done<br>5 = Not indicated - Growth (POSITIVE)<br>6 = Not indicated - Growth (CONTAMINATED)<br>7 = Not indicated - No growth (NEGATIVE)<br>8 = Not Available<br>9 = Not Recorded
Reported For	Includes: All Presentations<br>Excludes:
Description	Inoculation of culture media taken by the aspiration of a collection containing pus when considering infection causing pus formation – for example from the abdomen, peritoneal cavity (including in SBE or peritoneal dialysis peritonitis), pleural cavity, a joint or the skin.
Numerator (If Applicable)	n/a
Denominator (If Applicable)	n/a
Expressed As	Categorical

Study Sites

10.4.11 Ix: CXR

Definition	First CXR result during period of illness
Layout	NN (Number: 2 Characters)
Codeset (If Applicable)	0 = No CXR indicated<br>1 = Normal CXR<br>2 = Unilateral pulmonary infiltrates<br>3 = Bilateral pulmonary infiltrates<br>4 = Empyema<br>5 = Pulmonary Abscess<br>6 = Not Available<br>7 = Not Recorded
Reported For	Includes: All Presentations<br>Excludes:
Description	Duplicated for eligibility in data dictionary, but not in data collection form.
Numerator (If Applicable)	n/a
Denominator (If Applicable)	n/a
Expressed As	Categorical

Study Sites

10.4.12 Ix: US Scan

Definition	First Ultrasound Scan result during period of illness
Layout	NN (Number: 2 Characters)
Codeset (If Applicable)	0 = No US indicated<br>1 = Normal US Investigation<br>2 = US diagnosis of infection fitting with clinical symptoms<br>3 = US diagnosis of infection NOT fitting with clinical symptoms<br>4 = Procedure abandoned - unsuccessful<br>5 = Not Available<br>6 = Not Recorded
Reported For	Includes: All Presentations<br>Excludes:
Description	
Numerator (If Applicable)	n/a
Denominator (If Applicable)	n/a
Expressed As	Categorical

Study Sites

10.4.13 Ix: CT Scan

Definition	First CT result during period of illness
Layout	NN (Number: 2 Characters)
Codeset (If Applicable)	0 = No CT indicated<br>1 = Normal CT Investigation<br>2 = CT diagnosis of infection fitting with clinical symptoms<br>3 = CT diagnosis of infection NOT fitting with clinical symptoms<br>4 = Procedure abandoned - unsuccessful<br>5 = Not Available<br>6 = Not Recorded
Reported For	Includes: All Presentations<br>Excludes:
Description	
Numerator (If Applicable)	n/a
Denominator (If Applicable)	n/a
Expressed As	Categorical

Study Sites

10.4.14 Ix: MRI Scan

Definition	First MRI result during period of illness
Layout	NN (Number: 2 Characters)
Codeset (If Applicable)	0 = No MRI indicated<br>1 = Normal MRI Investigation<br>2 = MRI diagnosis of infection fitting with clinical symptoms<br>3 = MRI diagnosis of infection NOT fitting with clinical symptoms<br>4 = Procedure abandoned - unsuccessful<br>5 = Not Available<br>6 = Not Recorded
Reported For	Includes: All Presentations<br>Excludes:
Description	
Numerator (If Applicable)	n/a
Denominator (If Applicable)	n/a
Expressed As	Categorical

Study Sites

10.4.15 Ix: ERCP

Definition	First ERCP result during period of illness
Layout	NN (Number: 2 Characters)
Codeset (If Applicable)	0 = No ERCP indicated<br>1 = Normal ERCP Investigation<br>2 = ERCP confirmation of diagnosis of infection fitting symptoms<br>3 = ERCP diagnosis of infection NOT fitting with clinical symptoms<br>4 = Procedure abandoned - unsuccessful<br>5 = Not Available<br>6 = Not Recorded
Reported For	Includes: All Presentations<br>Excludes:
Description	
Numerator (If Applicable)	n/a
Denominator (If Applicable)	n/a
Expressed As	Categorical

Study Sites

10.4.16 Ix: TTE

Definition	First Trans-Thoracic Echocardiogram result during period of illness
Layout	NN (Number: 2 Characters)
Codeset (If Applicable)	0 = No TTE indicated<br>1 = Normal TTE Investigation<br>2 = TTE diagnosis of infection fitting with clinical symptoms<br>3 = TTE diagnosis of infection NOT fitting with clinical symptoms<br>4 = Procedure abandoned - unsuccessful<br>5 = Not Available<br>6 = Not Recorded
Reported For	Includes: All Presentations<br>Excludes:
Description	
Numerator (If Applicable)	n/a
Denominator (If Applicable)	n/a
Expressed As	Categorical

Study Sites

10.4.17 Ix: TOE

Definition	First Trans-Oesophageal Echocardiogram result during period of illness
Layout	NN (Number: 2 Characters)
Codeset (If Applicable)	0 = No TOE indicated<br>1 = Normal TOE Investigation<br>2 = TOE diagnosis of infection fitting with clinical symptoms<br>3 = TOE diagnosis of infection NOT fitting with clinical symptoms<br>4 = Procedure abandoned - unsuccessful<br>5 = Not Available<br>6 = Not Recorded
Reported For	Includes: All Presentations<br>Excludes:
Description	
Numerator (If Applicable)	n/a
Denominator (If Applicable)	n/a
Expressed As	Categorical

Study Sites

10.4.18 ED First Antibiotic Given (3 fields)

Definition	The name of the first dose of Antibiotics administered to patient
Layout	AAAAAAAAAA (Alpha – 12 Characters) Space for three columns
Codeset (If Applicable)	

0 = None Given
1 = Amoxicillin and Clavulinic Acid (Augmentin)
2 = Amoxicillin
3 = Penicillin (Benzyl penicillin)
4 = Flucloxacillin
5 = Cefuroxime
6 = Ceftriaxone
7 = Cefoxitin
8 = Cefepime
9 = Other Cephalosporin
10 = Erythromycin
11 = Roxithromycin
12 = Azithromycin
13 = Gentamicin
14 = Aztreonem
15 = Metronidazole
16 = Vancomycin
17 = Clindamycin
18 = Co-Trimoxazole
19 = Doxycycline
20 = Ciprofloxacin
21 = Norfloxacin
22 = Not Available
23 = Not Recorded

Reported For	Includes: All Sepsis Presentations<br>Excludes:
Description	Type name of antibiotics (best guess) given in ED here. Usually documented in patients notes. No electronic signature from Medications Room, no electronic capture. See Chapter 8.0 for definitions of not recorded and none given.
Numerator (If Applicable)	n/a
Denominator (If Applicable)	n/a
Expressed As	Categorical

Study Sites

10.4.19 ED First Antibiotic Route (3 fields)

Definition	The route of administration of the first dose of Antibiotics in ED
Layout	
Codeset (If Applicable)	0 = Not Recorded<br>1 = Oral<br>2 = Intravenous<br>3 = Intramuscular<br>4 = Not available
Reported For	Includes: All Sepsis Presentations<br>Excludes:
Description	Route of antibiotics (best guess) given in ED. Usually documented in patients notes. No electronic signature from Medications Room, no electronic capture. If record space for route blank – counted as not recorded.<br>Not available means notes for presentations not available (not the medication!)
Numerator (If Applicable)	n/a
Denominator (If Applicable)	n/a
Expressed As	Categorical

Study Sites

10.4.20 ED First Antibiotic Time (3 fields)

Definition	Time First dose of Antibiotics administered to patient
Layout	DD/MM/CCYY HH:MM:SS (Date and Time: 18 characters separated by a space between year and hour values)
Codeset (If Applicable)	n/a
Reported For	Includes: All Sepsis Presentations<br>Excludes:
Description	Usually documented in patients notes. Time of admin not an electronic dispensing signature from Medications Room.<br>All patients with severe sepsis should receive antibiotics in the ED. However as we see below, there is a difference between just 'any' antibiotics and the 'appropriate' antibiotic. Good practice dictates that "best Guess Therapy' be used for the suspected infection. The inappropriate choice of antibiotic may actually harm rather than help the patient.
Numerator (If Applicable)	n/a
Denominator (If Applicable)	n/a
Expressed As	Numeric (Time)

Study Sites

10.4.21 Predominant Culture Growth

Definition	The name of the bacterium PREDOMINENT growth in one or more sets of positive and non-contaminated body fluid culture from the patient.
Layout	AAAAAAAAAA (Alpha – 12 Characters)
Codeset (If Applicable)	0 = No Growth<br>1 = E-Coli<br>2 = Staphylococcus Aureus<br>3 = Staphylococcus Epidermidis<br>4 = Staphylococcus Saprophyticus<br>5 = Other Staph Species<br>6 = Streptococcus Pneumoniae<br>7 = Streptococcus Pyogenes<br>8 = Streptococcus Milleri<br>9 = Streptococcus Viridans<br>10 = Other Streptococcus Species<br>11 = Enterococcus Faecalis<br>12 = Bacteroides spp<br>13 = Neisseria Meningitides<br>14 = Pseudomonas<br>15 = Haemophilus Influenzae<br>16 = Proteus Mirabilis<br>17 = Listeria Monocytogenes<br>18 = Neisseria Gonorrhoeae<br>19 = Chlamydia Trachomatis<br>20 = Campylobacter Jujuni<br>21 = Yersinia Enterocolitica<br>22 = Salmonella<br>23 = Shigella<br>24 = Giardia<br>25 = Clostridium Difficile<br>26 = Moraxella Catarrhalis<br>27 = Legionella Pneumophilia<br>28 = Not available<br>29 = Not recorded
Reported For	Includes: All Sepsis Presentations<br>Excludes:
Description	These pathogens are based on the current “Best-Guess Antimicrobial Therapy” Guidelines for Auckland Hospital. It takes into account common pathogens in the surrounding populations. Predominant is the pathogen if only one cultured, or if more than one culture, the most common grown. If 2 are grown on one sample, it is the pathogen with the largest presence (i.e. large numbers of gram negative bacilli, small numbers of gram positive cocci – then e-coli would be recorded).
Expressed As	Categorical

Study Sites

10.4.22 ED Antibiotic Sensitivity (3 fields)

Definition	The sensitivity of the bacterium, yeast or other which is the PREDOMINENT growth in one or more sets of positive and non-contaminated body fluid culture from the patient to the first antibiotics (3 recorded) given to the patient in ED.
Layout	AAAAAAAAAA (Alpha – 12 Characters)
Codeset (If Applicable)	1 = Infecting agent sensitive to antibiotic<br>2 = Infecting agent intermediate to antibiotic<br>3 = Infecting agent resistant to antibiotic<br>4 = No Antibiotic given<br>5 = No culture growth<br>6 = Not Available<br>7 = Not Recorded
Reported For	Includes: All Sepsis Presentations<br>Excludes:
Description	This could be used to judge appropriateness of the first (best guess) antibiotics given in the ED.
Numerator (If Applicable)	n/a
Denominator (If Applicable)	n/a
Expressed As	Categorical

Study Sites

10.4.23 ED ANY Antibiotic Appropriate

Definition	Of those that were given antibiotics in ED, do the sensitivities of the predominant culture growth match any of the first antibiotics given in ED?
Layout	N (Number– 1 Character)
Codeset (If Applicable)	0 = No<br>1 = Yes
Reported For	Includes: All Sepsis Presentations with positive culture growth.<br>Excludes:
Description	
Numerator (If Applicable)	n/a
Denominator (If Applicable)	n/a
Expressed As	Binary

Study Sites

10.4.24 ED Best Guess Antibiotic Appropriate

Definition	Of those that were given antibiotics in ED; if there were no cultures done, or no culture growth, do the antibiotics given match best guess therapy for the postulated source of infection at the time in ED?
Layout	N (Number– 1 Character)
Codeset (If Applicable)	0 = Best Guess Not Appropriate<br>1 = Best Guess Appropriate<br>2 = No Antibiotic given<br>3 = Not Recorded<br>4 = Not Available
Reported For	Includes: All Sepsis Presentations with positive culture growth.<br>Excludes:
Description	Best Guess therapy is outlined earlier in the Data Dictionary pages 146 – 151. If the antibiotics given match any antibiotics in the best guess therapy tables (for the source of infection in 10.4.1) then this will be deemed either appropriate or inappropriate.
Numerator (If Applicable)	n/a
Denominator (If Applicable)	n/a
Expressed As	Binary

Study Sites

10.5 Time to Analgesia for ED Patients

Over half of all patients presenting to ED have pain[56, 57]. Pain, as defined by the Oxford English Dictionary, is a "highly unpleasant physical sensation caused by illness or injury" or "mental suffering or distress". It is also a highly subjective entity, and can be difficult to quantify. From the clinicians perspective severe pain should necessitate prompt relief, as it is ethical and humane practice to relieve suffering.

Timeliness of analgesia delivery in the ED is referenced through many of our daily working standards. Using the Australasian Triage Scale[58] (by which the acuity of every patient entering the ED is assessed) a triage category of 2, for example, is defined by needing assessment and treatment by a Doctor within 10 minutes as:

1. The patients' condition is *imminently Life threatening*
2. The patients' condition needs *important time-critical treatment*
3. ***The Patient has very severe pain of any cause-*** Humane practice mandates the relief of very severe pain or distress within 10 minutes

The Australasian College of Emergency Medicine who oversee the governance of ED in Australia and NZ have a policy on Acute Pain Management[59]. Point 3.3 states: "Emergency departments are responsible for regular monitoring of key clinical indicators related to best quality pain management (assessment, timeliness to intervention, and reassessment)".

The above policy is based on a consensus document (Acute Pain Management; Scientific Evidence[60]), released by the Australian National Health and Medical Research Council in collaboration with the Australasian College of Anaesthetists and the Faculty of Pain Medicine in 2010. This comprehensive document reiterates the need for timely relief of pain.

Studies have been done in many other countries in the world including Australia, Britain, France and America on the timeliness of analgesia delivery in those patients presenting in acute pain to ED. These have confirmed that pain relief is not adequate in ED and people can wait for a long time to receive any pain relief. Grant[61] showed a 68% non-compliance with suggested guidelines for pain relief, and a significant delay in pain relief delivery (mean waiting time 3 hours and 46 minutes for moderate pain).

There are many diverse factors affecting the delivery of analgesia, not just the fact it is a challenging subjective entity for the clinician at times! A recent literature review by Motov[62] looked at problems of and barriers to pain management in ED. They found problems included "a failure to acknowledge pain, a failure to assess initial pain, failure to implement pain management guidelines, failure to document pain and a failure to meet patient expectation". Barriers to pain management included "ethnic and racial bias, gender bias, age bias, inadequate knowledge and training of ED Physicians, opiophobia, ED environment and culture".

Arendts [63] showed there were multiple factors causing delay to opiate analgesia delivery, those being statistically significant were age, triage code, seniority of the treating doctor and ultimate disposition.

In the study we would like to look closer at ethnicity and health inequality. In the New Zealand 2006 Census, "European remained the largest of the major ethnic groups, with 67.6% of the population; the Maori ethnic group is the second largest 14.6%"[64]. Health inequalities in New Zealand are well documented[65], in particular, health inequities by ethnicity. When compared to American and Canadian Indigenous groups, Australian and NZ indigenous groups suffer much higher disease-specific mortality rates [66]. Inequities in analgesia delivery in other countries (especially America) are documented[67-69]. In a review of the literature in 2001, Todd et al[70] state "currently available research suggests that it is not the failure of physicians to adequately assess pain, but the failure to administer analgesics that is the principal contributor to oligoanalgesia among patients of minority ethnicity". In saying this there is some evidence exists to the contrary, that there is no disparity[71]. In relation to our study, ethnic disparity among analgesia delivery in EDs has not been studied before in NZ.

Only one Australasian study has compared ED overcrowding and time to analgesia [72]. Their conclusions were "No relationship between workload / overcrowding and Time to Analgesia was observed; however, there were delays to analgesia associated with age, non-English-speaking background and delay to pain assessment." However in other countries long waits in an overcrowded, overburdened ED for physician review, investigation and inpatient beds also correlates with delays for critical interventions and clinically orientated outcomes such as pain relief [73, 74], although this appears to be inconsistent in the literature.

Other international studies have looked at time to analgesia in ED for those who have a fracture neck of femur. Hwang et al[75] found older adults with hip fracture are at risk for underassessment of pain, considerable delays in analgesic administration after pain was identified and treatment with inappropriate medications. Over a third did not get any pain relief at all. When the ED is crowded (census levels greater than 120%) there was a significant association with poor or no pain documentation and longer times to pain assessment.

The Society for Academic Emergency Medicine (SAEM) Geriatric Task Force[76] noted analgesia delivery to be a quality gap in elderly people. They have derived 6 quality indicators for geriatric pain relief in ED. These include (relevant to our project)

- IF an older adult presents to the ED, then a formal assessment for the presence of acute pain should be documented within 1 hour of arrival to the ED.
- IF an older adult presents to the ED and has moderate to severe pain (i.e., a numeric rating scale score of 4 or higher out of 10), then pain treatment should be initiated (or the provider should document why treatment was not initiated).

This is why we have decided to look at time to analgesia in fracture neck of femur as well as time to operation, to capture this older population and time to analgesia in appendicitis to capture pain in a paediatric population.

The Clinical Standards for the College of Emergency Medicine (UK)[55] suggest the following standards for pain management:

Standards

1. Patients in severe pain (pain score 7 to 10) should receive appropriate analgesia, according to local guidelines,
   - 50% within 20 mins of arrival or triage whichever is the earliest
   - 75% within 30 mins of arrival or triage whichever is the earliest
   - 98% within 60 mins of arrival or triage whichever is the earliest
2. Patients with moderate pain (pain score 4 to 6) should be offered or receive analgesia, according to local guidelines,
   - 75% within 30 mins of arrival or triage whichever is the earliest
   - 90% within 60 mins of arrival or triage whichever is the earliest
3. 90% of patients with severe pain should have documented evidence of re-evaluation and action within 60 minutes of receiving the first dose of analgesic
4. 75% of patients with moderate pain should have documented evidence of re-evaluation and action within 60 minutes of receiving the first dose of analgesic
5. If analgesia is not prescribed and the patient has moderate or severe pain the reason should be documented in the notes.

Figure 5 (CEM UK Clinical Standards)

Time to Analgesia: The length of time it takes from presentation to the Emergency Department to receive analgesia for a painful condition. These have been chosen to cover all age groups.

- Adults >16 years with Renal Colic
- Adults >16 years with Fracture neck of Femur (covered in Fracture Neck of Femur data collection)
- All Presentations with Appendicitis (covered in Appendicitis data collection)

Time to analgesia for the purposes of the study will be:

- Less than 30 minutes in those with severe pain
- Less than 1 hour in those with moderate pain.

Adequacy of Analgesia:

Adequate analgesia should decrease a patient's pain by a clinically significant amount *and* to a level that is not more than mild. Kelly et al[77] found this minimum clinical difference to be 2mm on the VAS, or 2 points.

As defined by Jao et al[78]: "Reduction in the triage pain score by >= 2 points and to a level <4". This would mean moving from one severity category to the next lower and a severity category of mild.

Eligibility:

Pain on Arrival to ED

• No	
• Yes	X
• Not Recorded	
• Not Available	

Given Analgesia in ED

• No	
• Yes	
• Declined	
• Not Recorded	
• Not Available	

10.5.1 Pre-Hospital Analgesia Admin

Definition	The source of the pain relief before arrival to hospital
Layout	NN (Number: 2 Characters)
Codeset (If Applicable)	0 = None<br>1 = Ambulance Administered<br>2 = Primary Care Administered<br>3 = Self-Administered<br>4 = Hospital Administered<br>5 = Other Administered<br>6 = Not Recorded<br>4 = Not Available
Reported For	Includes: All ED Presentations<br>Excludes:
Description	Capturing those patients with the triage diagnosis of a painful condition, who have had Analgesia before arriving at the hospital (either Self, Ambulance, GP or Other). This is an attempt at having the severity of their pain settled with pain relief before coming to the hospital.
Numerator (If Applicable)	n/a
Denominator (If Applicable)	n/a
Expressed As	Categorical

Study Sites

10.5.2 Type of Pre-Hospital Analgesia

Definition	Type of analgesia the patient was given before hospital – either self administered, by paramedics or by Primary care health professionals.
Layout	NN (Number: 2 Characters)
Codeset (If Applicable)	0 = None<br>1 = Not Recorded<br>2 = Paracetamol<br>3 = Entonox<br>4 = Morphine<br>5 = Ketamine<br>6 = Penthrox<br>7 = Ibuprofen<br>8 = Diclofenac<br>9 = Codeine Phosphate<br>10 = Paracetamol and Codeine Combination<br>11 = Tramadol<br>12 = Mylanta or Gaviscon<br>13 = GTN<br>14 = Buscopan<br>15 = Other NSAID<br>16 = Other Opiate<br>17 = Multiple Medications given<br>18 = Declined<br>19 = Not Available
Reported For	Includes: All ED Presentations<br>Excludes: Pre-Hospital Analgesia
Description	Usually documented in patients notes, primary care notes (referral letter) or paramedic notes
Numerator (If Applicable)	n/a
Denominator (If Applicable)	n/a
Expressed As	Categorical

Study Sites

10.5.3 Route of Pre-Hospital Analgesia

Definition	Route of =pre-hospital analgesia given
Layout	NN (Number: 2 Characters)
Codeset (If Applicable)	0 = None<br>1 = Oral<br>2 = Intravenous<br>3 = Inhalational<br>4 = Intramuscular<br>5 = Subcutaneous<br>6 = Topical<br>7 = Intranasal<br>8 = Sublingual<br>9 = Rectal<br>10 = Nerve Block<br>11 = Declined<br>12 = Not Recorded<br>13 = Not Available
Reported For	Includes: All ED Presentations<br>Excludes: Pre-Hospital Analgesia
Description	Usually documented in patients notes.
Numerator (If Applicable)	n/a
Denominator (If Applicable)	n/a
Expressed As	Categorical

Study Sites

10.5.4 Arrival Pain Score ED

Definition	Those patients who have or have not had their pain score recorded at triage
Layout	NN (Number: 2 Characters)
Codeset (If Applicable)	1 = Yes<br>2 = Not Recorded<br>3 = Unable to Assess<br>4 = Declined Assessment<br>5 = Not Available
Reported For	Includes: All ED Presentations<br>Excludes:
Description	This is an attempt at having the severity of the patient's pain quantified, in order to assess urgency of review.
Numerator (If Applicable)	n/a
Denominator (If Applicable)	n/a
Expressed As	Categorical

Study Sites

10.5.5 Type of Pain Score Arrival ED

Definition	The pain score used to quantify pain
Layout	NN (Number: 2 Characters)
Codeset (If Applicable)	1 = Verbal Numeric Rating Pain Scale (1-10)<br>2 = Wong-Baker Faces Pain Scale<br>3 = Visual Analogue scale<br>4 = Categorical (Mild, Moderate, Severe)<br>5 = Not Recorded<br>6 = Unable to assess<br>7 = Declined assessment<br>8 = Not Available
Reported For	Includes: All ED Presentations<br>Excludes:
Description	This is an attempt at having the severity of pain quantified, in order to assess urgency of review and analgesia provision.
Numerator (If Applicable)	n/a
Denominator (If Applicable)	n/a
Expressed As	Categorical

Study Sites

10.5.6 Raw Pain Score Arrival ED

Definition	The severity of the pain recorded
Layout	NN (Number: 2 Characters)
Codeset (If Applicable)	Not Recorded (blank cell)
	Verbal Numeric Rating Pain Scale: Number 1-10 Figure 6

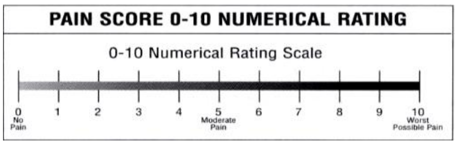

Wong-Baker Faces Pain Scale: Face number 1-6 Figure 7

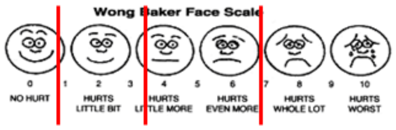

Face 1	Face 2	Faces 3 & 4	Faces 5 & 6
No Pain	Mild Pain	Moderate Pain	Severe Pain

Visual Analogue Scale: 1-10 centimetres Figure 8

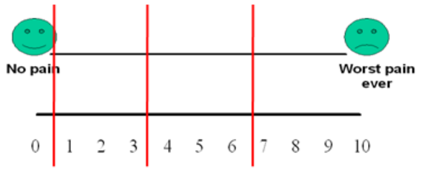

0	1-3	4-6	7-10
No Pain	Mild Pain	Moderate Pain	Severe Pain

Categorical: Mild, Moderate, Severe
No pain
Mild pain
Moderate Pain
Severe Pain

Reported For	Includes: All ED Presentations<br>Excludes:
Description	This is an attempt at having the severity of pain quantified, in order to assess urgency of review and analgesia provision. The raw score written down in the notes is a free text entry into the database.
Numerator (If Applicable)	n/a
Denominator (If Applicable)	n/a
Expressed As	String

Study Sites

10.5.7 Pain Score Categorical Arrival ED

Definition	A categorical score from the previous raw pain score data for the purposes of grouping people together for analysis.
Layout	NN (Number: 2 Characters)
Codeset (If Applicable)	0 = No pain<br>1 = Mild pain<br>2 = Moderate pain<br>3 = Severe pain<br>4 = Not Recorded<br>5 = Unable to Assess<br>6 = Declined Assessment<br>7 = Not Available
Reported For	Includes: All ED Presentations<br>Excludes:
Description	CEM UK and ACHS give guidelines on how promptly analgesia should be given in the emergency Department, and stratify into mild, moderate and severe pain. For purposes of analysis and comparison we will do the same here.
Numerator (If Applicable)	n/a
Denominator (If Applicable)	n/a
Expressed As	Categorical

Study Sites

10.5.8 First Assessment Pain Score ED

Definition	Those patients who have or have not had their pain score recorded at first nursing or medical assessment in the ED, pre-analgesia.
Layout	NN (Number: 2 Characters)
Codeset (If Applicable)	1 = Yes<br>2 = Not Recorded<br>3 = Unable to Assess<br>4 = Declined Assessment<br>5 = Not Available
Reported For	Includes: All ED Presentations<br>Excludes:
Description	This is an attempt at having the severity of the patient's pain quantified, in order to assess urgency of review.
Numerator (If Applicable)	n/a
Denominator (If Applicable)	n/a
Expressed As	Categorical

Study Sites

10.5.9 Type of Pain Score First Assessment ED

Definition	The pain score used to quantify pain during the first nursing or medical assessment in the ED, pre-analgesia.
Layout	NN (Number: 2 Characters)
Codeset (If Applicable)	1 = Verbal Numeric Rating Pain Scale (1-10)<br>2 = Wong-Baker Faces Pain Scale<br>3 = Visual Analogue scale<br>4 = Categorical (Mild, Moderate, Severe)<br>5 = Not Recorded<br>6 = Unable to assess<br>7 = Declined assessment<br>8 = Not Available
Reported For	Includes: All ED Presentations<br>Excludes:
Description	This is an attempt at having the severity of pain quantified, in order to assess urgency of review and analgesia provision.
Numerator (If Applicable)	n/a
Denominator (If Applicable)	n/a
Expressed As	Categorical

Study Sites

10.5.10 Raw Pain Score First Assessment ED

Definition
The severity of the pain recorded during the first nursing or medical assessment in the ED, pre-analgesia.

Layout
NN (Number: 2 Characters)

Codeset (If Applicable)
Not Recorded (blank cell)

Verbal Numeric Rating Pain Scale: Number 1-10 Figure 6

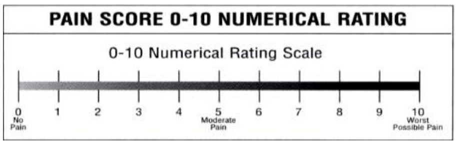

Wong-Baker Faces Pain Scale: Face number 1-6 Figure 7

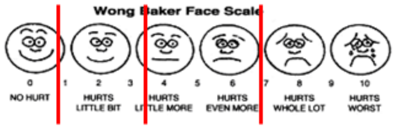

Face 1	Face 2	Faces 3 & 4	Faces 5 & 6
No Pain	Mild Pain	Moderate Pain	Severe Pain

Visual Analogue Scale: 1-10 centimetres Figure 8

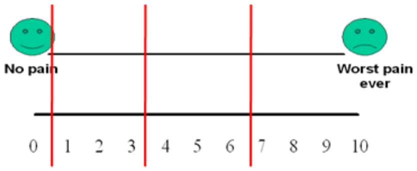

0	1-3	4-6	7-10
No Pain	Mild Pain	Moderate Pain	Severe Pain

Categorical: Mild, Moderate, Severe
No pain
Mild pain
Moderate Pain
Severe Pain

Reported For
Includes: All ED Presentations
Excludes:

Description
This is an attempt at having the severity of pain quantified, in order to assess urgency of review and analgesia provision. The raw score written down in the notes is a free text entry into the database.

Numerator (If Applicable) n/a

Denominator (If Applicable) n/a

Expressed As String

Study Sites

10.5.11 Pain Score Categorical First Assessment ED

Definition	A categorical score from the previous raw pain score data for the purposes of grouping people together for analysis. This is the score for the patients pain during the first nursing or medical assessment in the ED, pre-analgesia.
Layout	NN (Number: 2 Characters)
Codeset (If Applicable)	0 = No pain<br>1 = Mild pain<br>2 = Moderate pain<br>3 = Severe pain<br>4 = Not Recorded<br>5 = Unable to Assess<br>6 = Declined Assessment<br>7 = Not Available
Reported For	Includes: All ED Presentations<br>Excludes:
Description	CEM UK and AHCS give guidelines on how promptly analgesia should be given in the Emergency Department, and stratifies into moderate and severe pain. For purposes of analysis and comparison we will do the same here.
Numerator (If Applicable)	n/a
Denominator (If Applicable)	n/a
Expressed As	Categorical

Study Sites

10.5.12 First Analgesia Prescribe Time

Definition	The Time Analgesia first prescribed at
Layout	DD/MM/CCYY HH:MM:SS (Date and Time: 18 characters separated by a space between year and hour values)
Codeset (If Applicable)	n/a
Reported For	Includes: All ED Presentations<br>Excludes: Pre-Hospital Analgesia
Description	Usually documented in patients notes, or electronic signature from Medications Room / electronic capture.
Numerator (If Applicable)	n/a
Denominator (If Applicable)	n/a
Expressed As	Numeric (Time)

Study Sites

10.5.13 First Analgesia Time (4 entries)

Definition	The Time Analgesia first Administered at
Layout	DD/MM/CCYY HH:MM:SS (Date and Time: 18 characters separated by a space between year and hour values)
Codeset (If Applicable)	n/a
Reported For	Includes: All ED Presentations<br>Excludes: Pre-Hospital Analgesia
Description	Usually documented in patients notes, or electronic signature from Medications Room / electronic capture.
Numerator (If Applicable)	n/a
Denominator (If Applicable)	n/a
Expressed As	Numeric (Time)

Study Sites

10.5.14 Type of Analgesia (4 Entries)

Definition	Whether the patient was given analgesia or not
Layout	NN (Number: 2 Characters)
Codeset (If Applicable)	0 = None<br>1 = Not Recorded<br>2 = Paracetamol<br>3 = Morphine<br>4 = Ibuprofen<br>5 = Diclofenac<br>6 = Codeine Phosphate<br>7 = Paracetamol and Codeine Combination<br>8 = Tramadol<br>9 = Fentanyl<br>10 = Pethidine<br>11 = Buscopan<br>13 = Mylanta or Gaviscon<br>14 = GTN<br>15 = Other NSAID<br>16 = Other Opiate<br>17 = Entonox<br>18 = Nerve Block<br>19 = Declined<br>20 = Not Given<br>21 = Not Available
Reported For	Includes: All ED Presentations<br>Excludes: Pre-Hospital Analgesia
Description	Usually documented in patients notes as refused (R) or administered (timed and signed for by administrator).<br>Analgesia prescribed but no admin time = Not Recorded<br>No Analgesia prescribed = None
Numerator (If Applicable)	n/a
Denominator (If Applicable)	n/a
Expressed As	Categorical

Study Sites

10.5.15 Route of Analgesia (4 Entries)

Definition	Whether the patient was given analgesia or not
Layout	NN (Number: 2 Characters)
Codeset (If Applicable)	0 = None<br>1 = Oral<br>2 = Intravenous<br>3 = Inhalational<br>4 = Intramuscular<br>5 = Subcutaneous<br>6 = Topical<br>7 = Intranasal<br>8 = Sublingual<br>9 = Rectal<br>10 = Nerve Block<br>11 = Declined<br>12 = Not Recorded<br>13 = Not Available
Reported For	Includes: All ED Presentations<br>Excludes: Pre-Hospital Analgesia
Description	Usually documented in patients notes as refused (R) or administered (timed and signed for by administrator).
Numerator (If Applicable)	n/a
Denominator (If Applicable)	n/a
Expressed As	Categorical

Study Sites

10.5.16 Time First IV Opiate Analgesia

Definition	Time first IV opiate dose given
Layout	DD/MM/CCYY HH:MM:SS (Date and Time: 18 characters separated by a space between year and hour values)
Codeset (If Applicable)	n/a
Reported For	Includes: All ED Presentations<br>Excludes:
Description	Usually documented in patients notes.
Numerator (If Applicable)	n/a
Denominator (If Applicable)	n/a
Expressed As	Numeric (Time)

Study Sites

10.5.17 Number IV Opiate Doses

Definition	Whether the patient was given IV opiates or not and the number of doses they were given
Layout	NNN (Number: 3 Characters)
Codeset (If Applicable)	n/a
Reported For	Includes: All ED Presentations<br>Excludes:
Description	Usually documented in patients notes.
Numerator (If Applicable)	n/a
Denominator (If Applicable)	n/a
Expressed As	Numeric

Study Sites

10.5.18 Total IV Opiate Dose

Definition	The total amount of IV opiate the patient needed during their ED stay.
Layout	NNN (Number: 3 Characters)
Codeset (If Applicable)	n/a
Reported For	Includes: All ED Presentations<br>Excludes:
Description	Usually documented in patients notes. State the units of the dose on entry.
Numerator (If Applicable)	n/a
Denominator (If Applicable)	n/a
Expressed As	Numeric

Study Sites

10.5.19 Pain Score Reassessment: First Post-Analgesia

Definition	Those patients who have or have not had their pain score reassessed and recorded after analgesia. The first reassessment score post-analgesia will be recorded.
Layout	NN (Number: 2 Characters)
Codeset (If Applicable)	1 = Not Recorded<br>2 = Yes<br>3 = Unable to assess<br>4 = Declined assessment<br>5 = Not Available
Reported For	Includes: All ED Presentations<br>Excludes:
Description	This is an attempt at having the severity of pain quantified, in order to assess urgency of further review and further analgesia provision. Pain is a changing entity and it is important to be aware that for adequate analgesia re-assessment is needed.
Numerator (If Applicable)	n/a
Denominator (If Applicable)	n/a
Expressed As	Categorical

Study Sites

10.5.20 Time Pain Score Re-Assessed: First Post- Analgesia

Definition	The first time the patients pain score is first reassessed after analgesia is given
Layout	DD/MM/CCYY HH:MM:SS (Date and Time: 18 characters separated by a space between year and hour values)
Codeset (If Applicable)	n/a
Reported For	Includes: All ED Presentations<br>Excludes:
Description	Usually documented in patients notes. This will be difficult to capture. If the patient is on IV opiates, it is the time the first pain score re-assessment that is done from commencement of IV opiates. If not it will be the first documented entry in the notes detailing that some re-assessment of the patients pain has taken place.
Numerator (If Applicable)	n/a
Denominator (If Applicable)	n/a
Expressed As	Numeric (Time)

Study Sites

10.5.21 Type of Pain Score Reassessment: First Post-Analgesia

Definition	The pain score used to quantify pain during the first reassessment post-analgesia
Layout	NN (Number: 2 Characters)
Codeset (If Applicable)	1 = Verbal Numeric Rating Pain Scale (1-10)<br>2 = Wong-Baker Faces Pain Scale<br>3 = Visual Analogue scale<br>4 = Categorical (Mild, Moderate, Severe)<br>5 = Not Recorded<br>6 = Unable to assess<br>7 = Declined assessment<br>8 = Not Available
Reported For	Includes: All ED Presentations<br>Excludes:
Description	This is an attempt at having the severity of pain quantified, in order to assess urgency of further review and further analgesia provision.
Numerator (If Applicable)	n/a
Denominator (If Applicable)	n/a
Expressed As	Categorical

Study Sites

10.5.22 Raw Pain Score Reassessment: First Post-Analgesia

Definition	The severity of the pain recorded during the first reassessment of pain score post-analgesia
Layout	NN (Number: 2 Characters)
Codeset (If Applicable)	Not Recorded (blank cell)

Verbal Numeric Rating Pain Scale: Number 1-10 Figure 6

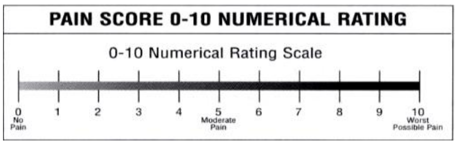

Wong-Baker Faces Pain Scale: Face number 1-6 Figure 7

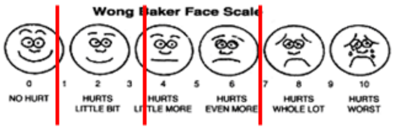

Face 1	Face 2	Faces 3 & 4	Faces 5 & 6
No Pain	Mild Pain	Moderate Pain	Severe Pain

Visual Analogue Scale: 1-10 centimetres Figure 8

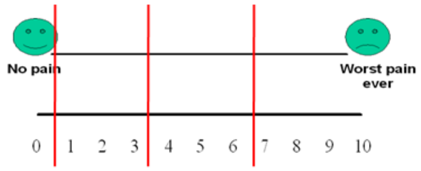

0	1-3	4-6	7-10
No Pain	Mild Pain	Moderate Pain	Severe Pain

Categorical: Mild, Moderate, Severe
No pain
Mild pain
Moderate Pain
Severe Pain

Reported For	Includes: All ED Presentations<br>Excludes:
Description	This is an attempt at having the severity of pain quantified, in order to assess urgency of further review and analgesia provision.
Numerator (If Applicable)	n/a
Denominator (If Applicable)	n/a
Expressed As	String

Study Sites

10.5.23 Pain Score Categorical Reassessment: First Post-Analgesia

Definition	A categorical score from the previous raw pain score data for the purposes of grouping people together for analysis. This will denote the severity of the patients' pain during the first assessment post-analgesia.
Layout	NN (Number: 2 Characters)
Codeset (If Applicable)	0 = No pain<br>1 = Mild pain<br>2 = Moderate pain<br>3 = Severe pain<br>4 = Not Recorded<br>5 = Unable to Assess<br>6 = Declined Assessment<br>7 = Not Available
Reported For	Includes: All ED Presentations<br>Excludes:
Description	CEM UK and AHCS give guidelines on how promptly analgesia should be given in the emergency Department, and stratifies into moderate and severe pain. For purposes of analysis and comparison we will do the same here.
Numerator (If Applicable)	n/a
Denominator (If Applicable)	n/a
Expressed As	Categorical

Study Sites

10.5.24 Other Subjective Pain Reassessment: Post-Analgesia

Definition	Those patients who have or have not had their pain score reassessed and recorded after analgesia in the ED. This could be any documented attempt at assessing the pain level of the patient, but not using any of the above pain scores. This will be a subjective assessment of the pain.
Layout	NN (Number: 2 Characters)
Codeset (If Applicable)	1 = Not Recorded<br>2 = Yes<br>3 = Unable to assess<br>4 = Declined assessment<br>5 = Not Available
Reported For	Includes: All ED Presentations<br>Excludes:
Description	This is an attempt at having the severity of pain quantified, in order to assess urgency of further review and further analgesia provision.
Numerator (If Applicable)	n/a
Denominator (If Applicable)	n/a
Expressed As	Categorical

Study Sites

10.5.25 Raw Subjective Pain Reassessment: Post-Analgesia

Definition	Those patients who have or have not had their pain score reassessed and recorded using a subjective means (plain language documentation in notes rather than the use of the validated pain scores), after analgesia in the ED.
Layout	NN (Number: 2 Characters)
Codeset (If Applicable)	N/A
Reported For	Includes: All ED Presentations<br>Excludes:
Description	This will be free text documentation of what has been recorded in the notes. This could be any documented attempt at assessing the pain level of the patient, but not using any of the above pain scores. This will be a subjective assessment of the pain.
Numerator (If Applicable)	n/a
Denominator (If Applicable)	n/a
Expressed As	String

Study Sites

10.5.26 Type of Pain Score: Lowest ED

Definition	The pain score used to quantify the lowest severity of pain during the patients stay in ED
Layout	NN (Number: 2 Characters)
Codeset (If Applicable)	1 = Verbal Numeric Rating Pain Scale (1-10)<br>2 = Wong-Baker Faces Pain Scale<br>3 = Visual Analogue scale<br>4 = Categorical (Mild, Moderate, Severe)<br>5 = Not Recorded<br>6 = Unable to assess<br>7 = Declined assessment<br>8 = Not Available
Reported For	Includes: All ED Presentations<br>Excludes:
Description	This is an attempt to quantify the adequacy of analgesia given
Numerator (If Applicable)	n/a
Denominator (If Applicable)	n/a
Expressed As	Categorical

Study Sites

10.5.27 Raw Pain Score: Lowest ED

Definition: The documented lowest severity pain recorded during the patients ED stay.

Layout: NN (Number: 2 Characters)

Codeset (If Applicable): Not Recorded (blank cell)

Verbal Numeric Rating Pain Scale: Number 1-10 Figure 6

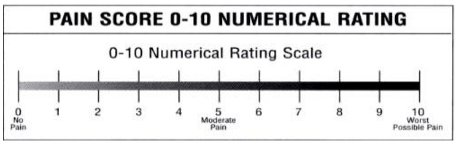

Wong-Baker Faces Pain Scale: Face number 1-6 Figure 7

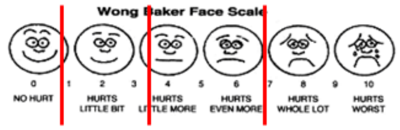

Face 1 No Pain	Face 2 Mild Pain	Faces 3 & 4 Moderate Pain	Faces 5 & 6 Severe Pain

Visual Analogue Scale: 1-10 centimetres Figure 8

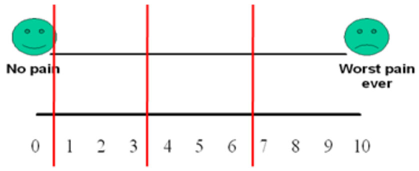

0 No Pain	1-3 Mild Pain	4-6 Moderate Pain	7-10 Severe Pain

Categorical: Mild, Moderate, Severe

No pain

Mild pain

Moderate Pain

Severe Pain

Reported For: Includes: All ED Presentations

Excludes:

Description: This is an attempt to quantify the adequacy of analgesia given

Numerator (If Applicable): n/a

Denominator (If Applicable): n/a

Expressed As: String

Study Sites

10.5.28 Pain Score Categorical: Lowest ED

Definition	A categorical score from the previous raw pain score data for the purposes of grouping people together for analysis. This will denote the lowest severity of the patients pain during their stay in ED
Layout	NN (Number: 2 Characters)
Codeset (If Applicable)	0 = No pain<br>1 = Mild pain<br>2 = Moderate pain<br>3 = Severe pain<br>4 = Not Recorded<br>5 = Unable to Assess<br>6 = Declined Assessment<br>7 = Not Available
Reported For	Includes: All ED Presentations<br>Excludes:
Description	This is an attempt to quantify the adequacy of analgesia given. For purposes of adequacy it is"Reduction in the triage pain score by >= 2 points and to a level <4". This would mean moving from one severity category to the next lower and a documented severity category of mild during the ED stay.
Numerator (If Applicable)	n/a
Denominator (If Applicable)	n/a
Expressed As	Categorical

Study Sites

10.6 Time to Treatment in Acute Asthma for ED Patients

Asthma is the most common chronic lung disease in the developed and developing world. Asthma is a chronic inflammatory disorder associated with airway hyper-responsiveness, reversible airflow limitation and respiratory symptoms (shortness of breath, cough and wheezing)[79]. The severity of acute exacerbation determines the treatment. Guidelines and articles used for this purpose include:

CEM (UK) Clinical Standards for Emergency Departments[55]

British Thoracic Society – Guideline on the Management of Asthma[80]: Chapter 6 & Annex 3

New Zealand Guidelines Group[81] – Treatment of Acute Severe Asthma

Review Article – Canadian Medical Journal: Management of Acute Asthma in Adults in the Emergency Department[82]

Review Article – Chest: Acute Asthma in Adults, a Review[79].

Starship Children's' Health Clinical Guideline: Management of Acute Asthma[83]

Acute Asthma Severity Tool (Children)– New Zealand Guidelines Group[84]

Cochrane Review: Early Emergency Department Treatment of Acute Asthma with Systemic Corticosteroids[85].

B-Agonists (Bronchodilator): Salbutamol, Albuterol, Terbutaline, Adrenaline. It has been shown that nebulised AND spaced B-agonists are as efficacious as each other so both these routes of administration will be considered in data collection. However if the presentation severity is life-threatening nebulised or IV delivery should be used[80]. All guidelines suggest that first treatment be done as initial nursing assessment is done (i.e. first set of observations)

Anticholinergic (Bronchodilator), Ipratropium: Meta-analysis of RCT's has shown that combining Ipratropium with B-Agonists results in greater improvement in lung function and a significant reduction in hospital admission, particularly in patients with severe to life-threatening air flow obstruction[82]. Suggested use in these Canadian guidelines[82] is "if poor response to first bolus of B-agonist in moderate and severe asthma and to be given immediately with first bolus Salbutamol in life-threatening asthma". In NZ Guidelines for Asthma management[81] they state "Ipratropium should be given within the first 90 minutes of arrival to ED. The addition of inhaled Anticholinergic medication (Ipratropium 0.5 mg via nebuliser or 80 µg via MDI) at the first presentation of acute asthma improves peak flow rate and symptoms over 90 minutes and reduces hospital admissions (NNT=18)[86]".

Steroids: A recent Cochrane review[85] on time to treatment with systemic corticosteroids (in all but the mildest cases) suggested they be given within 1 hour of arrival. It was shown that this significantly reduces the need for hospital admission and benefits of faster delivery were most marked in those with severe or life-threatening asthma or those who are not currently taking steroids. It also documented that children respond well to oral steroids. All the adults studies reviewed had parenteral steroids. BTS[80] guidelines suggest steroids should be given within 20-60 minutes and CEM Clinical practice guidelines[55] suggest within 30 minutes for moderate, severe and life-threatening asthma. Lougheed at al [87] described admission rates at adult hospital sites were positively associated with delay in receiving systemic steroids in the ED (p=0.004).

Adults and Children have the same treatment times according to CEM guidelines reviewed – there are just slightly different ways of scoring severity and drug dosing. As we are looking at time to treatment, both adults and children will be stratified to moderate, severe and life-threatening asthma on presentation. We will look at time to treatment overall, and within the stratified severity groups. Data collection and severity stratification will be done separately for adults and children. Times to treatment are as recommended in the Clinical Standards for Emergency Departments (College of Emergency Medicine UK). Excerpts relevant to our study are as follows:

"A) Life threatening asthma

1. Evidence in the notes that Oxygen was being given on arrival
2. Salbutamol 5mg ***or*** Terbutaline 5 – 10mg + Ipratropium 0.5mg by **nebuliser *or*** Salbutamol 250 microgram (5 mcg/kg) i**ntravenously** given **within 5 minutes of arrival in adults**.
3. Salbutamol 2.5mg or Terbutaline 5mg + Ipratropium 0.25mg given by **spacer or nebuliser within 5 minutes of arrival in children**
4. In 98% of cases documented evidence of pulse rate, respiratory rate and oxygen saturation measured on arrival
5. CXR performed
6. IV hydrocortisone 100mg or **oral prednisone** 40-50mg given **within 30 minutes of arrival in adults**.
7. **IV hydrocortisone** 100mg (50 mg if 2 – 5 years) or **oral prednisone** 30 - 40mg (20mg if 2 – 5 years) given **within 30 minutes of arrival in children"**

"B) Moderate / Severe asthma

1. Evidence in the notes that Oxygen was being given on arrival
2. Salbutamol 5mg or Terbutaline 5 - 10mg given **by nebuliser within 10 minutes of arrival in adults**.
3. Salbutamol 2.5mg or Terbutaline 5mg by **nebuliser** or Beta2 agonist 2 – 10 puffs via **spacer device** given **within 10 minutes of arrival in children**
4. 98% documented evidence of peak flow, pulse rate, respiratory rate and oxygen saturation measured on arrival
5. 90% of cases **IV hydrocortisone** 100mg or **oral prednisone** 30-50mg (20mg if 2 – 5 years) **given within 30 minutes of arrival adults and children**
6. 90% of discharged adult patients should have oral prednisolone 30 – 50mg for 5 days
7. 90% of discharged paediatric patients should have oral prednisolone 20mg (2 – 5 years) or 30 – 40 mg (over 5 years) for 3 days"

Figure 9: (CEM UK Clinical Standards)

Adults:

Severity (ADULT)	Moderate Acute Asthma	Severe Acute Asthma	Life-Threatening Asthma to Fatal
Clinical Signs	1.Increase in Symptoms **i.e. breathless, cough, wheeze**<br>2.Able to talk in sentences<br>3.Monophasic or Biphasic Wheeze	1.Increase in Symptoms<br>2.Inability to complete sentences in one breath<br>3.Biphasic Wheeze **(expiratory and inspiratory)**<br>Prolonged Exp Phase	1.Increase in Symptoms<br>2.One or two words per breath<br>Unable to Speak<br>3. Audible Wheeze from end of bed<br>Silent Chest<br>4.Cyanosis<br>5.Exhaustion<br>Coma<br>6.Decreased level of consciousness<br>7.Poor Resp effort<br>Needs ventilation<br>8.Respiratory Arrest<br>9.Hypotension<br>Cardiovascular Collapse<br>10.Arrhythmia - AF etc.<br>11.Cardiac Arrest
PEFR	50-75% Predicted	33-50% Predicted	<33% Predicted<br>Unable to Complete
PaO2 (ABG)	Normal	Normal	<8kPa
PaCO2 (ABG)	4.6 - 6.0 kpa	4.6 - 6.0 kpa	> 6.0 kpa
SpO2	>=92%	>=92%	<92%
RR	< 25	>25	any (extremes)
HR	< 110	>=110	any (extremes)
No features Severe	✓	X	X
No features Life-Threatening	✓	✓	X
No features Fatal	✓	✓	X
Treatment Time	Salbutamol via spacer within 5 mins<br>Steroids(PO) between 20 and 60 minutes	Salbutamol via nebuliser within 5 mins<br>Steroids (PO) between 20 and 60 minutes	Oxygen IMMEDIATELY<br>Salbutamol via nebuliser or IV IMMEDIATELY<br>Ipratropium via nebuliser IMMEDIATELY<br>Steroids (IV or PO) IMMEDIATELY

Figure 10: (NZGG and BTS Asthma Guidelines)

Children:

Severity (CHILD)	Moderate Acute Asthma	Severe Acute Asthma	Life-Threatening Asthma
Clinical Signs	1.Increase in Symptoms<br>2.Able to talk in sentences<br>3.Monophasic or Biphasic Wheeze<br>4.Mild Indrawing or Accessory Muscle use	1.Increase in Symptoms<br>2. Inability to complete sentences in one breath, or unable to talk or feed.<br>3.Biphasic Wheeze<br>Prolonged Exp Phase<br>Audible Wheeze<br>4.Moderate Indrawing / Accessory Muscle Use / Tracheal Tug evident	1.Increase in Symptoms<br>2. One or two words per breath, unable to feed.<br>3.Silent Chest<br>4.Severe Accessory Muscle Use or Indrawing<br>5.Decreased LOC<br>Coma<br>6.Exhaustion<br>7.Cyanosis<br>8.Poor Resp effort<br>Needs Mechanical Ventilation<br>Respiratory Arrest<br>9.Bradycardia<br>10. Pulsus Parodoxus<br>11.Cardiac Arrest
PEFR	>=50% Predicted	33-50% Predicted	<33% Predicted<br>Unable to complete
PaO2 (ABG)	Normal	Normal	<8 kPa
PaCO2 (ABG)	4.6 – 6.0 kPa	4.6 – 6.0 kPa	>6.0 kPa
SpO2	>=92%	>=92%	<92%
RR (per min)	>= 40 in 2-5 yrs<br>>= 20 in > 5 yrs	>= 50 in 2-5 yrs<br>>= 30 in > 5 yrs	Any (extremes)
HR (per min)	>= 110 in 2-5 yrs<br>>= 100 in >5 yrs	>= 130 in 2-5 yrs<br>>= 120 in >5 yrs	Any (extremes)
Asthma Severity Score ASS (below)	3 to 5	6	6 (with other features of life-threatening or fatal)
Treatment Time	Salbutamol via spacer within 5 mins<br>Steroids(PO) between 20 and 60 minutes	Salbutamol via spacer or nebuliser within 5 mins<br>Steroids (PO) between 20 and 60 minutes	Oxygen IMMEDIATELY<br>Salbutamol via nebuliser or IV IMMEDIATELY<br>Ipratropium nebulised together with Salbutamol<br>Steroids (IV) IMMEDIATELY

Figure 11: (NZGG and BTS Asthma Guidelines)

**Paediatric Asthma Severity Score (ASS)**<br>Add wheeze and muscle subtotals to give score	
Score	
**Wheeze (beware of silent chest*)**	
None (0)	
Expiratory (by auscultation) (1)	
Expiratory & inspiratory (2)	
Heard without stethoscope (3)	
**Sub Total**	
**Accessory muscle use / indrawing**	
None (0)	
Mild (1)	
Moderate (2)	
Severe (3)	
**Sub Total**	
**TOTAL**	
**0-2 Mild 3-5 Moderate 6 = Severe**	

Figure 12: Starship Hospital Clinical Guidelines on Asthma[83]

Asthma severity will be quantified into moderate and severe – this differentiation is due to the inherent clinical difference in management – usually mild and moderate asthma can be discharged home, whereas severe asthma needs admission, in nearly all cases, to hospital. Mild asthma is excluded as steroids are rarely given.

Steroids are given in moderate, severe and life-threatening asthma. The time to steroids as 60 minutes is being used as the standard in the SSED study. New Zealand guidelines do not have a recommended minimum time for steroid delivery; however there seems to be consensus among different guidelines (UK, Canada) and a Cochrane review to support this as an appropriate time to measure and to expect steroids to be given within. The NZGG Asthma[81] recommend bronchodilators are given immediately if clinically indicated. The draft ACHS Emergency Medicine Clinical Indicators[4] suggest salbutamol (for paediatric patients) within 30 minutes of arrival. NZGG suggest Ipratropium within 90 minutes for moderate--- to severe asthma which we will use for our study.

Time to Salbutamol

- **Immediately for Severe and Life-Threatening Asthma**

Time to Ipratropium

- **Within 90 minutes for Moderate and Severe Asthma**
- **Immediately for Life-Threatening Asthma**

Time to Corticosteroids

- **Within 60 minutes for Moderate and Severe Asthma**
- **Immediately for Life-Threatening Asthma**

Eligibility and Severity Stratification: Eligibility will be assessed on having moderate, severe or life-threatening asthma and having been given steroids in the Emergency Department.

Increase in Asthma Symptoms

• No	
• Yes	X
• Not recorded	
• Not available	

Talking in Sentences

• Able to talk in sentences	
• Inability to complete sentence in one breath	
• One or two words per breath	
• Unable to speak	
• Not Recorded	
• Not Available	

Talking in Sentences (**Paeds Only**)

• Able to talk in sentences / feeding normally	
• One or two words per breath / interrupted feeding	
• Unable to talk or feed	
• Not Recorded	
• Not Available	

Work of Breathing (**Paeds Only**)

• None	
• Mild in drawing / accessory muscle use	
• Moderate in drawing / accessory muscle use	
• Severe in drawing / accessory muscle use	
• Not Recorded	
• Not Available	

Presence of Wheeze

• No Wheeze, undistressed	
• Monophasic Wheeze or Biphasic Wheeze	
• Biphasic Wheeze, prolonged expiration	
• Audible wheeze (end of bed)	
• Silent Chest, distressed	
• Not Recorded	
• Not Available	

Presence of Wheeze (**Paeds Only**)

• No Wheeze, undistressed	
• Monophasic Wheeze	
• Biphasic Wheeze	
• Audible wheeze (end of bed)	
• Silent Chest, distressed	
• Not Recorded	
• Not Available	

Cyanosis

• No	
• Yes	
• Not recorded	
• Not available	

Exhaustion

• No	
• Yes	
• Not recorded	
• Not available	

Level of Consciousness (AVPU)

• Alert	
• Responsive to Voice	
• Responsive to Pain	
• Unresponsive	
• Not Recorded	
• Not Available	

Respiratory Arrest

• No	
• Yes	
• Not recorded	
• Not available	

Arrhythmia

• No	
• Yes	
• Not recorded	
• Not available	

Cardiac Arrest

• No	
• Yes	
• Not recorded	
• Not available	

PEFR Measured (First): this should only be in mild, moderate and severe asthma, not necessarily in life-threatening.

NNN

PEFR: Best as recorded in clinical notes or can be substituted, predicted from PEFR Tables (in data collection forms: substitute = mid-height predictor for age and gender)

NNN

Oxygen Saturations

NNN (%)

Systolic Blood Pressure (not Paeds)

NNN (mmHg)

Heart Rate

NNN (mmHg)

Respiratory Rate

NNN

10.6.1 Pre-Hospital Treatment Given

Definition	The total number of patients who present to the Emergency Department, with the discharge diagnosis of Acute Asthma – who have been treated with any asthma medication prior to arrival in ED within the previous 1 hour.
Layout	N (Number: 1 Character)
Codeset (If Applicable)	0= No<br>1 = Yes<br>2 = Declined<br>3 = Not Recorded<br>4 = Not Available
Reported For	Includes: All ED Presentations with Asthma<br>Excludes: Self-Presentations
Description	Capturing the number of patients who present to the Emergency Department suffering from an acute exacerbation of asthma who have been given treatment prior to arriving for their symptoms within the previous 1 hour.
Numerator (If Applicable)	n/a
Denominator (If Applicable)	n/a
Expressed As	Categorical

Study Sites

10.6.2 Pre-Hospital Treatment Admin

Definition	The source of pre-hospital treatment – within the previous 1 hour.
Layout	N (Number: 1 Character)
Codeset (If Applicable)	0 = None<br>1 = Self-Administered<br>2 = Primary Care Administered<br>3 = Ambulance Administered<br>4 = Hospital Administered<br>5 = Self and Primary Care Administered<br>6 = Self and Ambulance Administered<br>7 = Self and Hospital Administered<br>8 = Primary Care and Ambulance Administered<br>9 = Primary Care and Hospital Administered<br>10 = Ambulance and Hospital Administered<br>11 = Self, Primary Care and Ambulance Administered<br>12 = Self, Primary Care and Hospital Administered<br>13 = Self, Ambulance and Hospital Administered<br>14 = Primary Care, Ambulance and Hospital Administered<br>15 = Self, Primary Care, Ambulance and Hospital Administered<br>16 = Not Recorded<br>17 = Not Available
Reported For	Includes: All ED Presentations with Asthma<br>Excludes: Self-Presentations
Description	Capturing the number of patients who present to the Emergency Department suffering from an acute exacerbation of asthma who have been given treatment directly (i.e. within the previous 1 hour) prior to arriving for their symptoms.<br>If 10.6.1 is 'declined' answer 'none' here.
Numerator (If Applicable)	n/a
Denominator (If Applicable)	n/a
Expressed As	Categorical

Study Sites

10.6.3 Pre-Hospital Medications

Definition	The total number of patients who present to the Emergency Department, with the discharge diagnosis of Acute Asthma – who have been treated with any asthma medication prior to arrival in ED.
Layout	N (Number: 1 Character)
Codeset (If Applicable)	0 = None<br>1 = Inhaled Salbutamol<br>2 = Nebulised Salbutamol<br>3 = Intravenous Salbutamol<br>4 = Oral Salbutamol<br>5 = Combination Inhaled and Nebulised Salbutamol<br>6 = Combination Nebulised and Intravenous Salbutamol<br>7 = Combination Inhaled, Nebulised and Intravenous Salbutamol<br>8 = Inhaled Ipratropium<br>9 = Nebulised Ipratropium<br>10 = Combination Inhaled and Nebulised Ipratropium<br>11 = Hydrocortisone<br>12 = Prednisone<br>13 = Nebulised Steroids<br>14 = Combination Oral and Intravenous Steroids<br>15 = Combination Oral and Nebulised Steroids<br>16 = Combination Nebuliser and Intravenous Steroids<br>17 = Combination Oral, Nebuliser and Intravenous Steroids<br>18 = Adrenaline (any form)<br>19 = Multiple medications<br>20 = Not Recorded<br>21 = Not Available
Reported For	Includes: All ED Presentations with Asthma<br>Excludes: Self-Presentations
Description	Capturing the number of patients who present to the Emergency Department suffering from an acute exacerbation of asthma who have been given treatment prior to arriving for their symptoms.<br>If 10.6.1 is 'declined' answer 'none' here.
Numerator (If Applicable)	n/a
Denominator (If Applicable)	n/a
Expressed As	Categorical

Study Sites

10.6.4 ABG Taken

Definition	Those patients who present to the Emergency Department, with the diagnosis of Asthma, who have had an Arterial Blood Gas (ABG) taken
Layout	N (Number: 1 Character)
Codeset (If Applicable)	0 = No<br>1 = Yes<br>2 = Refused<br>3 = Failed attempts, abandoned<br>4 = Contraindicated<br>5 = Not Recorded<br>6 = Not Available
Reported For	Includes: All ED Presentations with Asthma<br>Excludes:
Description	Capturing the number of patients who have had quantification of arterial blood gas oxygen and carbon dioxide concentrations. In asthmatics low partial pressures of oxygen (PaO_2) and high partial pressures of carbon dioxide ($PaCO_2$) in arterial blood suggest respiratory failure and is a life-threatening situation. They are more rarely used in children as compared to adults as it is an invasive procedure. This is more commonly used as a quantification of the severity of life-threatening asthma.
Numerator (If Applicable)	n/a
Denominator (If Applicable)	n/a
Expressed As	Categorical

Study Sites

10.6.5 CXR Taken

Definition	Those patients who present to the Emergency Department, with the diagnosis of Asthma, who have had a Chest X-Ray (CXR) taken
Layout	N (Number: 1 Character)
Codeset (If Applicable)	0 = No<br>1 = Yes<br>2 = Refused<br>3 = Not Recorded<br>4 = Not Available
Reported For	Includes: All ED Presentations with Asthma<br>Excludes:
Description	Chest X-Rays are not a standard investigation in asthma and are not recommended. However in life-threatening asthma they are important to exclude another cause of severe sudden deterioration (such as a Pneumothorax). Children also frequently have an X-Ray taken to exclude foreign body inhalation or if a first episode of asthma. Although again this is not standard and chest x-rays are discouraged in children, due to radiation exposure.
Numerator (If Applicable)	n/a
Denominator (If Applicable)	n/a
Expressed As	Categorical

Study Sites

10.6.6 CXR Time

Definition	Time of Chest X-Ray
Layout	DD/MM/CCYY HH:MM:SS (Date and Time: 18 characters separated by a space between year and hour values)
Codeset (If Applicable)	n/a
Reported For	Includes: All Patients with Asthma<br>Excludes:
Description	The time recorded on the fist X-Ray taken during the ED admission.
Numerator (If Applicable)	n/a
Denominator (If Applicable)	n/a
Expressed As	Numeric (Time)

Study Sites

10.6.7 Steroids Given in ED

Definition	Those patients who present to the Emergency Department, with the diagnosis of Asthma, who were given steroid medication IN ED
Layout	N (Number: 1 Character)
Codeset (If Applicable)	0 = No<br>1 = Yes<br>2 = Pre-Hospital Administration<br>3 = Declined<br>4 = Contraindicated<br>5 = Not Recorded<br>6 = Not Available
Reported For	Includes: All ED Presentations with Asthma<br>Excludes:
Description	
Numerator (If Applicable)	n/a
Denominator (If Applicable)	n/a
Expressed As	Categorical

Study Sites

10.6.8 Route of Steroids

Definition	Route of steroid delivery used in the ED
Layout	NN (Number: 2 Characters)
Codeset (If Applicable)	0 = None<br>1 = Oral<br>2 = Inhaled<br>3 = Nebuliser<br>4 = Intravenous<br>5 = Combination Oral and Intravenous<br>6 = Combination Oral and Nebuliser<br>7 = Combination Nebuliser and Intravenous<br>8 = Combination Oral, Nebuliser and Intravenous<br>9 = Not Recorded<br>10 = Not Available
Reported For	Includes: All ED Presentations with asthma<br>Excludes:
Description	Usually documented in patients notes as refused (R) or administered (timed and signed for by administrator).<br>If 10.6.7 is 'declined', then answer 'none'.
Numerator (If Applicable)	n/a
Denominator (If Applicable)	n/a
Expressed As	Categorical

Study Sites

10.6.9 First Steroid Time in ED

Definition	The time first dose of Steroids given in the Emergency Department, of any route
Layout	DD/MM/CCYY HH:MM:SS (Date and Time: 18 characters separated by a space between year and hour values)
Codeset (If Applicable)	n/a
Reported For	Includes: All ED Presentations with Asthma<br>Excludes: Pre-Hospital Steroids
Description	Usually documented in patients notes, or electronic signature from Medications Room / electronic capture.
Numerator (If Applicable)	n/a
Denominator (If Applicable)	n/a
Expressed As	Numeric (Time)

Study Sites

10.6.10 Salbutamol Given in ED

Definition	Those patients who present to the Emergency Department, with the diagnosis of Asthma, who were given Salbutamol medication IN ED
Layout	N (Number: 1 Character)
Codeset (If Applicable)	0 = No<br>1 = Yes<br>2 = Pre-Hospital Administration<br>3 = Declined<br>4 = Contraindicated<br>5 = Not Recorded<br>6 = Not Available
Reported For	Includes: All ED Presentations with Asthma<br>Excludes:
Description	
Numerator (If Applicable)	n/a
Denominator (If Applicable)	n/a
Expressed As	Categorical

Study Sites

10.6.11 Route of Salbutamol

Definition	Route of salbutamol delivery used in the ED
Layout	NN (Number: 2 Characters)
Codeset (If Applicable)	0 = Not Given<br>1 = Oral<br>2 = Inhaled<br>3 = Nebuliser<br>4 = Intravenous<br>5 = Combination Inhaled and Nebuliser<br>6 = Combination Nebuliser and Intravenous<br>7 = Combination Inhaled, Nebuliser and Intravenous<br>8 = Not Recorded<br>9 = Not Available
Reported For	Includes: All ED Presentations with asthma<br>Excludes:
Description	Usually documented in patients notes as refused (R) or administered (timed and signed for by administrator).<br>If 10.6.10 is 'declined', then answer 'none'.
Numerator (If Applicable)	n/a
Denominator (If Applicable)	n/a
Expressed As	Categorical

Study Sites

10.6.12 First Salbutamol Time in ED

Definition	The time first dose of Salbutamol given in the Emergency Department, of any route
Layout	DD/MM/CCYY HH:MM:SS (Date and Time: 18 characters separated by a space between year and hour values)
Codeset (If Applicable)	n/a
Reported For	Includes: All ED Presentations with Asthma<br>Excludes: Pre-Hospital Salbutamol
Description	Usually documented in patients notes, or electronic signature from Medications Room / electronic capture.
Numerator (If Applicable)	n/a
Denominator (If Applicable)	n/a
Expressed As	Categorical

Study Sites

10.6.13 Ipratropium Given in ED

Definition	Those patients who present to the Emergency Department, with the diagnosis of Asthma, who were given Ipratropium medication IN ED
Layout	N (Number: 1 Character)
Codeset (If Applicable)	0 = No<br>1 = Yes<br>2 = Declined<br>3 = Contraindicated<br>4 = Not Recorded<br>5 = Not Available
Reported For	Includes: All ED Presentations with Asthma<br>Excludes:
Description	
Numerator (If Applicable)	n/a
Denominator (If Applicable)	n/a
Expressed As	Categorical

Study Sites

10.6.14 Route of Ipratropium

Definition	Route of Ipratropium delivery used in the ED
Layout	NN (Number: 2 Characters)
Codeset (If Applicable)	0 = None<br>1 = Inhaled<br>2 = Nebuliser<br>3 = Combination Inhaled and Nebuliser<br>4 = Not Recorded<br>5 = Not Available
Reported For	Includes: All ED Presentations with asthma<br>Excludes:
Description	Usually documented in patients notes as refused (R) or administered (timed and signed for by administrator).<br>If 10.6.13 is 'declined', then answer 'none'.
Numerator (If Applicable)	n/a
Denominator (If Applicable)	n/a
Expressed As	Categorical

Study Sites

10.6.15 First Ipratropium Time in ED

Definition	The time first dose of Ipratropium given in the Emergency Department, of any route
Layout	DD/MM/CCYY HH:MM:SS (Date and Time: 18 characters separated by a space between year and hour values)
Codeset (If Applicable)	n/a
Reported For	Includes: All ED Presentations with Asthma<br>Excludes:
Description	Usually documented in patients notes, or electronic signature from Medications Room / electronic capture.<br><br>In NZ Guidelines for Asthma management (58) they state Ipratropium should be given within the first 90 minutes of arrival to ED. "The addition of inhaled Anticholinergic medication (Ipratropium 0.5 mg via nebuliser or 80 µg via MDI) at the first presentation of acute asthma improves peak flow rate and symptoms over 90 minutes and reduces hospital admissions (NNT=18)[86, 88]".
Numerator (If Applicable)	n/a
Denominator (If Applicable)	n/a
Expressed As	Numeric (Time)

Study Sites

10.6.16 Adrenaline Given (at any time)

Definition	Number of patients who have Adrenaline administered at any time – either prior to ED or in ED
Layout	NNNNNNN (Number: 7 Characters)
Codeset (If Applicable)	0 = No<br>1 = Yes - ED<br>2 = Yes - Pre-Hospital Administration<br>3 = Declined<br>4 = Contraindicated<br>5 = Not Recorded<br>6 = Not Available
Reported For	Includes: All Patients in ED with Asthma<br>Excludes:
Description	Adrenaline is used only in severe to life-threatening cases of acute asthma. This is to quantify how many patients are given adrenaline at any point of their exacerbation.<br><br>Subset: those patients presenting with life-threatening asthma, severe asthma and paediatric patients
Numerator (If Applicable)	
Denominator (If Applicable)	
Expressed As	Categorical

Study Sites

10.6.17 Oxygen Therapy Start Time in ED

Definition	The time of first Oxygen Administration in the Emergency Department (and this has been a continuous treatment for at least 1 hour.)*
Layout	DD/MM/CCYY HH:MM:SS (Date and Time: 18 characters separated by a space between year and hour values)
Codeset (If Applicable)	n/a
Reported For	Includes: All ED Presentations with Asthma<br>Excludes: Pre-Hospital Oxygen, solely nebulizer driven by oxygen with breaks in therapy*
Description	Usually documented in patients notes, or electronic signature from Medications Room / electronic capture.<br>If a patient was on oxygen in the ambulance – the time may be equal to presentation time as it is most likely continued. If patient is placed on an oxygen driven nebulizer, but oxygen not continued after nebulizer has finished this is not considered as continuous oxygen therapy*. If however the patient is on continuous nebulised medications this is equivalent to oxygen therapy and start time is the same as continuous nebs start time.
Numerator (If Applicable)	n/a
Denominator (If Applicable)	n/a
Expressed As	Numeric (Time)

Study Sites

10.6.18 Discharge Prescription Steroids

Definition	Was a discharge prescription given for steroids – if discharged from ED
Layout	NN (Number: 2 Characters)
Codeset (If Applicable)	0 = No<br>1 = Yes<br>2 = Not Recorded<br>3 = Not Available
Reported For	Includes: All ED Presentations with asthma discharged from ED<br>Excludes: Those admitted to Hospital
Description	Usually documented in patients notes or on discharge summary
Numerator (If Applicable)	n/a
Denominator (If Applicable)	n/a
Expressed As	Categorical

Study Sites

10.7 CT Head and Time to Operating Room in Acute Head Injury

Head injury (HI): is defined as any trauma to the head, other than superficial injuries to the face[89].

Traumatic Brain Injury (TBI): refers specifically to an injury of the brain substance itself[90].

"TBI is a leading cause of disability in all regions of the globe: The global incidence rate of TBI is 200 per 100,000 people per year, however the rate is uncertain and likely underestimated"[91] (many people with mild TBI do not seek medical attention).

From the NZ Guideline for the Management of Traumatic Brain Injury[92]: "New Zealand data on hospital presentations for mild TBI found a rate of 437 per 100,000 per year for people aged 15 years and older, and 252 per 100,000 per year for those aged less than 15......A total TBI incidence figure for New Zealand, including those people with TBI who do not seek medical attention, is likely to be in the range of 20,000 to 30,000 cases per year".

Demographics of TBI in NZ Population – approx 2/3 male, incidence peaks in the 15-30 age group and again over 60 years of age. 14% of people with concussion identify as Maori and 5% as Pacific peoples.

It is appropriate in those patients with moderate and severe Traumatic Brain Injury that CT scanning be done as soon as possible after arrival in the ED to allow diagnosis, surgery and invasive monitoring to proceed expeditiously – to aim for the least primary and secondary brain injury and aim for a good neurological outcome following the injury.

Clinical Distinction of Severity of Head Injury: ACC New Zealand[92]

Criteria for classifying the severity of traumatic brain injury

SEVERITY OF INJURY	GLASGOW COMA SCALE SCORE	DURATION OF POST-TRAUMATIC AMNESIA
Mild	13–15	‹24 hours
Moderate	9–12	1–6 days
Severe	3–8	7 days or more

If there is a discrepancy between the severity level for the GCS score and post-traumatic amnesia, it is appropriate to use the more severe category (eg, GCS score of 14 but post-traumatic amnesia for 2 days = moderate TBI).

Figure 13: ACC Guideline, Traumatic Brain injury – Diagnosis, Acute Management and Rehabilitation[92]

Decision to CT

- **Any patient with moderate or severe TBI (blunt head trauma and/or LOC and/or amnesia and/or disorientation)**

1. Canadian CT Head Rules[93] (Minor Head Injury only)
Mild Head Injury and any of the following suggest high-risk for needing surgical intervention:
   - GCS <15 2 hours after injury
   - Suspected open or depressed skull fracture
   - Any sign of basal skull fracture
   - Two or more episodes of vomiting
   - Age > 65 years

Mild head injury and any of the following suggest moderate-risk for needing surgical intervention:
   - Amnesia before impact
   - Dangerous Mechanism

2. American College of Emergency Physicians (ACEP): Decision making in Adult Mild TBI (2008)[90]
Level A recommendation CT Head when-
Head Trauma **with** LOC or post-traumatic amnesia and one or more of the following:
Headache
Vomiting
Age> 60
Drug or ETOH intoxication
Deficit in Short Term Memory
Physical evidence of Trauma above Clavicle
Post-Traumatic Seizure
GCS < 15
Focal Neurological Deficit
Coagulopathy

Level B recommendation CT Head when –
Head Trauma with **no** LOC or post-traumatic amnesia and one or more of the following:
Focal Neurological Deficit
Vomiting
Severe Headache
Age>65
Clinical signs of basilar skull fracture
GCS <15
Coagulopathy
Dangerous mechanism of injury

3. NICE Guidelines (UK) Algorithm[89]

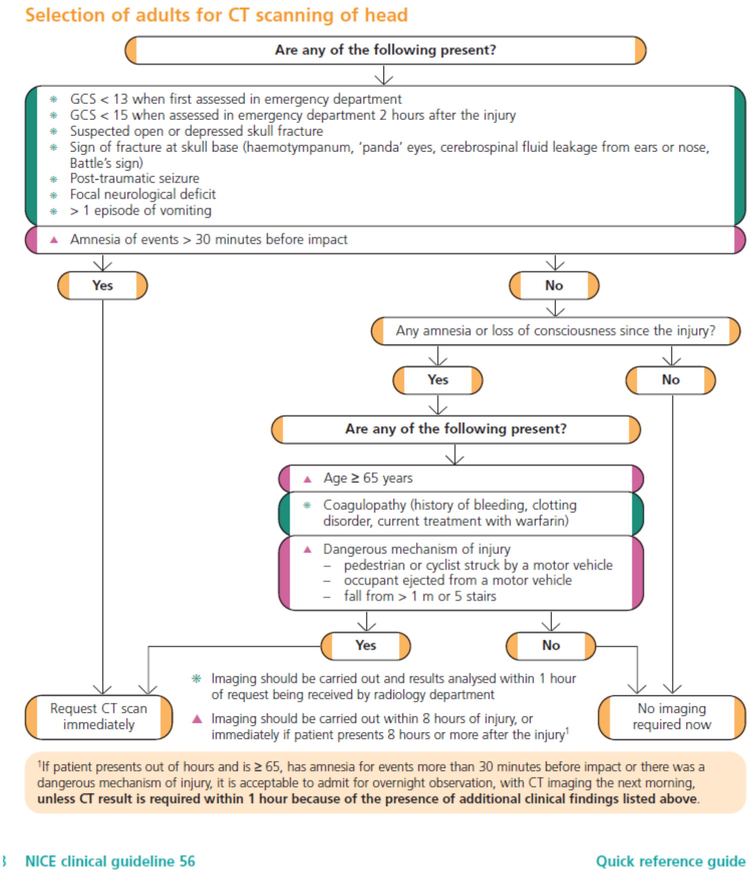

Both algorithms from NICE Guidelines UK[89]

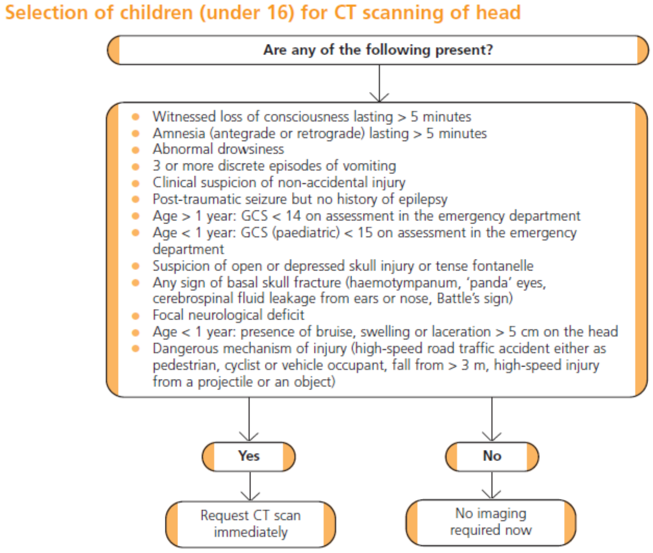

Figure 14: NICE Guidelines Acute Head Injury

Time to CT:

1. NICE[89]:

Adults:

CT Head to be performed within 1 hour of the request being received and confirmed by the radiology department, with any of the following risk factors:

- GCS < 13 when first assessed in emergency department
- GCS < 15 when assessed in emergency department 2 hours after the injury
- Suspected open or depressed skull fracture
- Sign of fracture at skull base
- Post-traumatic seizure
- Focal neurological deficit
- 1 episode of vomiting
- Coagulopathy (history of bleeding, clotting disorder, current treatment with Warfarin)

CT Head Imaging should be carried out within 8 hours of injury, or immediately if patient presents 8 hours or more after the injury when:

- None of the above
- Dangerous Mechanism of injury
- Age > 65 years
- Amnesia of events more than 30 minutes

Children (Under 16)
Any of the Following warrant CT imaging:

- Witnessed loss of consciousness lasting > 5 minutes
- Amnesia (antegrade or retrograde) lasting > 5 minutes
- Abnormal drowsiness
- 3 or more discrete episodes of vomiting
- Clinical suspicion of non-accidental injury
- Post-traumatic seizure, but no history of epilepsy
- Age > 1 year: GCS < 14 on assessment in the emergency department
- Age < 1 year: GCS (paediatric) < 15 on assessment in the emergency department
- Suspicion of open or depressed skull injury or tense fontanelle
- Any sign of basal skull fracture
- Focal neurological deficit
- Age < 1 year: presence of bruise, swelling or laceration > 5 cm on the head
- Dangerous mechanism of injury

2. College Emergency Medicine[55] (CEM: UK)

"**90% of CT imaging should be performed within 1 hour of the request having been received by the radiology department**."

3. Traumatic Brain Injury Guidelines NZ[92] (excerpt)

"Adults: **Immediate CT if**:

- Any deterioration in condition
- GCS <13 when assessed, irrespective of time elapsed since the injury
- GCS of 13 or 14 two hours after the injury
- Suspected open or depressed skull fracture
- Any sign of basal skull fracture
- Post-traumatic seizure
- Focal neurological deficit
- More than one episode of vomiting
- Amnesia for >30 minutes for events before the injury.

Request an **immediate CT scan** for adults who have sustained a head injury with some loss of consciousness or amnesia since the injury and ANY of these risk factors:

- Age ≥65 years
- Coagulopathy (history of bleeding, clotting disorder, current treatment with Warfarin)
- High-risk mechanism of injury (a pedestrian struck by a motor vehicle, an occupant ejected from a motor vehicle, or a fall from a height of >1 metre or >5 stairs)".

“The decision to CT scan should be applied regardless of the influence of intoxication”.

“In some situations (e.g. rural centres with limited access to CT), observation for 24 hours rather than CT scan is a reasonable option. Discuss the appropriateness of observation with the relevant neurosurgical centre.

People with the following factors **MUST** be referred for CT scan: (i.e. it is inappropriate if these patients do NOT have a CT scan).

• Any deterioration in condition

• GCS <13 at time of assessment irrespective of time elapsed since the injury or GCS of 13 to 14 two hours after injury

• Any sign of basal skull fracture

• Focal neurological deficit

“Children:

• Post-injury adverse events or signs, including focal neurological deficits and seizures (excepting immediate)

• A paediatric GCS of ≤13, particularly an initial or ‘field’ (pre-hospital) GCS of ≤13, or any decrease in GCS

• Skull fracture, either obvious or suspected on the basis of clinical signs

• injury resulting from a fall from 1 metre or 5 stairs, or less in the case of younger children non-accidental cause of injury

• Lethargy or irritability on examination.

• Soft tissue injury such as swelling or haematoma (if under 2)

• Occipital or temporal/parietal location of injury (if under 2)”

4. The Society of British Neurological Surgeons[94]

Recommended in 1998 (before CT scanners were as widely available as they are today) that “CT Scanning be performed urgently, within 2-4 hours of admission”. This Society has more recently had input and had guidelines released in collaboration with the NICE in the UK (detailed above).

5. Royal College of Radiology (RCR UK)[95] use the Canadian CT Head Rules for guidelines for CT Imaging in acute head injury.

Mild TBI:

CT should be performed within 1 hour of arrival if any high risk features

CT should be performed within 8 hours of arrival if only moderate-risk features and no high risk features

Moderate to Severe TBI:

CT should be performed within 1 hour of arrival

6. Scottish Intercollegiate Guidelines Group[96]:

Adults:

Indications and Time to CT similar to RCR Guidelines (follow Canadian CT Head Rules – immediate if severe TBI, moderate TBI or mild TBI with high risk features, within 8 hours if mild TBI with moderate-risk features).

Children:

Immediate CT scanning should be done in a child (<16 years) who has any of the following features:

- GCS≤13 on assessment in emergency department
- Witnessed loss of consciousness >5 minutes
- Suspicion of open or depressed skull injury or tense fontanelle
- Focal neurological deficit
- Any sign of basal skull fracture.

CT scanning should be considered within eight hours if any of the following features are present (excluding indications for an immediate scan):

- Presence of any bruise/swelling/laceration >5 cm on the head
- Post-traumatic seizure, but no history of epilepsy nor history suggestive of reflex anoxic seizure
- Amnesia (antegrade or retrograde) lasting >5 minutes
- Clinical suspicion of non-accidental head injury
- A significant fall age under one year: GCS<15 in emergency department assessed by personnel experienced in paediatric GCS monitoring
- Three or more discrete episodes of vomiting
- Abnormal drowsiness (slowness to respond)."

Time to Neurosurgery in Eligible Patients:

1. Royal College of Surgeons[97] (RCS UK)
   "The system of care should achieve surgical evacuation of a clinically significant Extradural haematoma within 4 hours of the onset of symptoms."

2. Brain Trauma Foundation[98] (US)

Extradural Haematoma

"Time from neurological deterioration, as defined by onset of coma, pupillary abnormalities or neurological deterioration to surgery, is more important than time between trauma and surgery. In these patients, surgical evacuation should be done as soon as possible since every hour delay in surgery is associated with progressively worse outcome."

- >300 mls, evacuate regardless of GCS
- < 300mls, < 15mm thickness **and** < 5 mm midline shift **and** GCS> 8 **and** no focal deficit can be managed non-operatively (serial CT and close neuro obs in specialist centre).
- Acute EDH with GCS < 9 and asymmetry of pupils (lesion causing compression therefore higher risk of poor outcome)= evacuation
- Surgical Evacuation (if indicated) = **ASAP**
- **Craniotomy**

Acute Subdural Haematoma

"The literature supports the statement that the length of time from clinical deterioration to operative treatment of a SDH is significantly related to outcome. In summary, there is evidence that patients who undergo surgery within 2-4[99] hours after clinical deterioration have a better outcome than those who undergo delayed surgery."

- Acute SDH >10mm, **or** > 5mm midline shift should be evacuated regardless of GCS
- All patients with acute SDH and GCS <9 should have ICP
- If GCS <9 **and/or** GCS decreased by 2 or more points pre-hospital (between time of injury and hospital presentation) **and/or** asymmetric/fixed pupils **and/or** ICP >20mmHg with acute SDH <10mm or <5mm midline shift should have surgical evacuation, otherwise can be treated non-operatively
- Surgical Evacuation (if indicated) = **within 2 to 4 hours**
- **Craniotomy +/- Bone Flap and Duraplasty**

Intra-Parenchymal Lesions (contusion and intra-cerebral haematoma)

- GCS <8 with frontal or temporal contusions greater than 200mls in volume **with** midline shift >5mm **and/or** cisternal compression should undergo evacuation
- Any patient with lesion greater than 500mls
- Parenchymal lesion with no neuro compromise, ICP, < 20mmHg no mass effect on CT can be managed non-operatively.
- Within 24 to 48 hours refractory high ICP **and/or** medically resistant cerebral oedema **and** impending herniation.
- Craniotomy, Decompressive Craniectomy

Acute Posterior Fossa Mass Lesions

- Patients with mass effect on CT scan **or** with neurological dysfunction **or** deterioration referable to the lesion should undergo operative intervention
- Evacuation = ASAP
- **Suboccipital Craniectomy**

Depressed Fractures of the Skull

- Patients with open (compound) skull fractures depressed greater than the thickness of the skull should undergo operative intervention to prevent infection.
- Early Operation
- Elevation and Debridement

Literature times to surgery from arrival to ED or injury are based on **2 hours, 3 hours, 4 hours and 6 hours (for EDH and SDH)**

Two NZ studies have been done using time to surgery within 4 hours as a benchmark, one looking at the transfer of intubated patients to Auckland Hospital with TBI[100] and the other looking at time to definitive care for patients with moderate to severe TBI[101] at Auckland Hospital**.**

Inequality and TBI in NZ: From ACC Guidelines for TBI (NZ): based on ACC entitlement claims by ethnicity.

In NZ there are significant ethnic disparities across many areas of healthcare – Maori have high incidence rates of TBI and it is under-reported. Maori are also at risk from poorer outcomes.

Pacific peoples are also most likely under-represented in TBI related claims, stigma can surround illness and disability. There are also multiple barriers of access for Pacific peoples to health services (i.e. cost and language).

Quality Indicators for Trauma Care:

The American College of Surgeons have a Trauma Quality Improvement Program that collects data from 111 centres, benchmarks trauma care, identifies structures and process of care. The ACSCOT indicators used are:

- **Patients with Glasgow Coma Scale <13 who do not receive a head CT within 2 h**
- Comatose trauma patient leaving ED before mechanical airway established
- Laparotomy not performed within 2 h of arrival at ED
- **Subdural or epidural hematoma receiving craniotomy more than 4 h after arrival**
- **Cranial surgery performed more than 24 h after arrival at ED**
- Thoracic surgery performed more than 24 h after arrival at ED
- Abdominal surgery performed more than 24 h after arrival at ED
- All patients developing deep vein thromboses, pulmonary emboli or decubitus ulcers
- Interval of more than 8 h between arrival and treatment of blunt compound tibial fracture
- Non-fixation of femoral diaphyseal fracture in adult trauma patient

There are 2 papers from Canada and Australia reviewing the evidence for quality indicators in Trauma Care.

The first Canadian paper is a scoping review[102] – the findings are re-iterated in a second paper (2011 - systematic review[103]). The QI's in this paper only relate to adults over the age of 18 years. 40 articles were included and of those 5 looked at quality indicators for Traumatic Brain Injury. The papers reviewed looked mostly at the ACSCOT indicators, other indicators used pertaining to quality care in TBI were "GCS <13 and no CT head in < 2 hours", "TBI and surgery < 4 hours", "Time from Injury to operative SDH evacuation", "GCS < 14 and no CT Head < 2 hours", "TBI and OR < 4 hours". These all seem to be based on ACSCOT indicators. The ranges of times looked at for CT head in the literature were GCS < 10, or < 13, or < 14 and CT Head < 30 mins, < 2 hours, < 4 hours. The ranges of times to craniotomy in **severe TBI** in the literature were < 60mins, < 2 hours, < 4 hours and anything more than 4 hours was 'delayed'. The review identified 115 quality indicators in the literature for trauma care. However the evidence is weak and as such a c ore group of indicators could not be recommended. "**Evidence for improved quality of care after QI implementation was strongest for depressed level of consciousness and no CT Head or delayed CT head**".

The Australian Study[104] investigated the construct validity of 14 trauma QI's (ACSCOT and the Victorian State Trauma Registry QI's), through their ability to identify patients at risk of poor outcomes (increased mortality, increased LOS and increased use of ICU). Data was taken from the Victorian State Trauma Registry, for which the QI's are:

- Failure to activate trauma team at major trauma service
- Patients with Glasgow Coma Scale <9 who are not intubated
- Head scan completed more than 2 h after arrival at the ED
- Patients with penetrating torso trauma taking more than 1 h to theatre

Related to head trauma the only indictor found to have a risk of poor outcome was increased LOS if cranial surgery was more than 24 hours after arrival at ED (OR 2.1 CI 1.1 – 3.8).

The Trauma Audit and Research Network in the UK has a UK-wide database (50% of NHS Trusts), with documented indicators to measure the quality of trauma care. This is developed from the RCS (UK) and BOA (UK) document "Better Care for the Severely Injured"[105]. There is no such nation-wide trauma network in NZ. They use the following as QI's for TBI:

- Pre-Hospital and In-Hospital Spine protection
- All trauma receiving hospitals much have 24-hour access to CT scanning and on-call radiology
- Patients with severe head injuries or focal signs should be transferred to a neurosurgical unit regardless of whether they need surgical intervention
- Measure Glasgow Coma Scale

Discharge Advice:

- Documentation if a head injury advice sheet and verbal advice was given to those patients discharged home. (NICE)
- Only discharged home if a suitable carer is at home (NICE)
- Anyone who has had CT imaging of head should receive GP follow-up within 1 week (NICE)
- Children GCS of 13 or less at anytime or who had CT should receive GP follow-up 1 week post injury (NZGG Head Injury)
- Letter to GP and school should be generated for all school-age children who have a head injury (NICE)
- Letter to GP and pre-school should be generated for all pre-school-age children who have a head injury (NICE)

For the purposes of the SSED NRP we will classify:

Severe Head Injury (Severe TBI): GCS on arrival to ED <=8/15
Moderate Head Injury (Moderate TBI): GCS on arrival to ED 9-12/15
Mild Head Injury (Mild TBI): GCS on arrival to ED 13-15/15

Time to CT within 1 hour (90%) for:
All head injuries
All severe TBI
All moderate TBI
All mild TBI with high-risk features from CCT Head Rules

Time to CT within 2 hours for:
All head injuries
All mild TBI
All mild TBI with moderate-risk features on CCT Head Rules (no high risk features)

Time to CT within 8 hours for:
All head injuries
All mild TBI
All mild TBI with moderate-risk features on CCT Head Rules (no high risk features)

Time to OR for Extra-Dural Haematoma (EDH)
Within 2 hours of arrival to ED
Within 4 hours of arrival to ED

Time to OR for Sub-Dural Haematoma (SDH)
Within 2 hours of arrival to ED
Within 4 hours of arrival to ED

May not be able to capture surgical time for those transferred for surgical management to another hospital – North Shore, Middlemore and Waitakere possibly from Auckland notes, Waikato yes (transfers to their NS Unit are excluded) but Hawkes Bay no.

Appropriateness of CT:
All Severe TBI = Yes
All Moderate TBI = Yes
Mild TBI = Yes if fulfils ACC guidelines for needing CT imaging.

Eligibility (for Appropriateness of CT Brain):

Traumatic Head Injury

• No	
• Yes	X
• Not Recorded	
• Not Available	

Eligibility (for Time to CT Brain):

CT Brain

• No	
• Yes	X
• Not Recorded	
• Not Available	

10.7.1 Time of Injury

Definition	The time of the injury.
Layout	DD/MM/CCYY HH:MM:SS (Date and Time: 18 characters separated by a space between year and hour values)
Codeset (If Applicable)	n/a
Reported For	Includes: All ED Presentations with Head Injury<br>Excludes:
Description	If time documented in the ambulance or patient notes use this, if not use ambulance dispatch time as a surrogate (if available).
Numerator (If Applicable)	n/a
Denominator (If Applicable)	n/a
Expressed As	Numeric (Time)

Study Sites

10.7.2 Ambulance Arrival Time @ Scene

Definition	The time of arrival of the first ambulance crew at the pre-hospital scene
Layout	DD/MM/CCYY HH:MM:SS (Date and Time: 18 characters separated by a space between year and hour values)
Codeset (If Applicable)	n/a
Reported For	Includes: All ED Presentations with Head Injury who arrive by ambulance / air ambulance<br>Excludes: Those who self-present
Description	Usually documented in ambulance patient notes. If the patient did not arrive by ambulance leave cell blank – this can be matched up with “arrival mode” from electronic data.
Numerator (If Applicable)	n/a
Denominator (If Applicable)	n/a
Expressed As	Numeric (Time)

Study Sites

10.7.3 Pre-Hospital GCS (lowest)

Definition	The lowest recorded Glasgow Coma Score pre-hospital
Layout	NN (numeric 2 characters)
Codeset (If Applicable)	n/a
Reported For	Includes: All ED Presentations with Head Injury who arrive by ambulance / air ambulance<br>Excludes: Those who self-present
Description	Usually documented in ambulance patient notes.<br>This is a discrete number out of 15 (minimum score 3, maximum score 15) given to people who have suffered a head injury. It looks at motor, verbal and eye responses to differing levels of stimulus.
Numerator (If Applicable)	n/a
Denominator (If Applicable)	n/a
Expressed As	Ordinal

Study Sites

10.7.4 Pre-Hospital Motor Score Adults (lowest)

Definition	The lowest recorded motor score of the patient pre-hospital
Layout	Alphanumeric
Codeset (If Applicable)	1 = Obeys Commands<br>2 = Localises to Pain<br>3 = Withdrawal from Pain<br>4 = Flexion to Pain (Decorticate)<br>5 = Extension to Pain (Decerebrate)<br>6 = No Motor Response<br>7 = Not Recorded<br>8 = Not Available
Reported For	Includes: All ED Presentations with Head Injury who arrive by ambulance / air ambulance<br>Excludes: Those who self-present
Description	Usually documented in ambulance patient notes.<br>This is the motor score from the GCS Score – however the motor score is a better predictor of outcome alone after traumatic brain injury[106].
Numerator (If Applicable)	n/a
Denominator (If Applicable)	n/a
Expressed As	Ordinal

Study Sites

10.7.5 Pre-Hospital Motor Score Paeds (lowest)

Definition	The lowest recorded motor score of the patient pre-hospital
Layout	Alphanumeric
Codeset (If Applicable)	1 =Obeys Commands or normal spontaneous movements<br>2 =Localises to painful stimulus or withdraws to touch<br>3 =Withdrawal to painful stimulus<br>4 =Abnormal flexion to pain (Decorticate)<br>5 =Abnormal extension to pain (Decerebrate)<br>6 =No motor response to pain<br>7 = Not Recorded<br>8 = Not Available
Reported For	Includes: All ED Presentations with Head Injury who arrive by ambulance / air ambulance<br>Excludes: Those who self-present
Description	Usually documented in ambulance patient notes.<br>This is the motor score from the GCS Score – however the motor score is a better predictor of outcome alone after traumatic brain injury.
Numerator (If Applicable)	n/a
Denominator (If Applicable)	n/a
Expressed As	Ordinal

Study Sites

10.7.6Pre-Hospital Pupils (worst)

Definition	The worst recorded pre-hospital papillary response of the patient
Layout	Alphanumeric
Codeset (If Applicable)	1 = Attempted but Unable to assess<br>2 = Both Pupils equal and reactive<br>3 = Left Pupil only dilated, unreactive<br>4 = Right Pupil only dilated, unreactive<br>5 = Both Pupils dilated and unreactive<br>6 = Not Recorded<br>7 = Not Available
Reported For	Includes: All ED Presentations with Head Injury who arrive by ambulance / air ambulance<br>Excludes: Those who self-present
Description	Usually documented in ambulance patient notes.<br>This is the papillary examination of the patient. If the pupil is dilated or unreactive this suggests a third nerve palsy which in itself suggests rising intracranial pressure and/or cerebral herniation. This can also be used for prognostication of outcome following TBI[106].
Numerator (If Applicable)	n/a
Denominator (If Applicable)	n/a
Expressed As	Ordinal

Study Sites

10.7.7 Pre-Hospital CNS Depressants Medications given.

Definition	Type of medication given to the patient at the pre-hospital scene
Layout	Alphanumeric
Codeset (If Applicable)	0 = None<br>1 = Opiate<br>2 = Benzodiazepine<br>3 = Ketamine<br>4 = Other CNS Depressant Medications<br>5 = Multiple Medications<br>6 = Not Recorded<br>7 = Not Available
Reported For	Includes: All ED Presentations with Head Injury who arrive by ambulance / air ambulance<br>Excludes: Those who self-present
Description	Usually documented in ambulance patient notes.
Numerator (If Applicable)	n/a
Denominator (If Applicable)	n/a
Expressed As	Categorical

Study Sites

10.7.8 Pre-Hospital Medication Route

Definition	Route of medication given to the patient at the pre-hospital scene
Layout	Alphanumeric
Codeset (If Applicable)	0 = None<br>1 = Oral<br>2 = Intravenous<br>3 = Inhalational<br>4 = Intramuscular<br>5 = Subcutaneous<br>6 = Topical<br>7 = Intranasal<br>8 = Sublingual<br>9 = Rectal<br>10 = Multiple Routes<br>11 = Not Recorded<br>12 = Not Available
Reported For	Includes: All ED Presentations with Head Injury who arrive by ambulance / air ambulance<br>Excludes: Those who self-present
Description	Usually documented in ambulance patient notes.
Numerator (If Applicable)	n/a
Denominator (If Applicable)	n/a
Expressed As	Categorical

Study Sites

10.7.9 Intubation Pre-Hospital

Definition	Whether the patient was intubated at the pre-hospital scene
Layout	Alphanumeric
Codeset (If Applicable)	0 = No<br>1 = Yes<br>2 = Not Recorded<br>3 = Not Available
Reported For	Includes: All ED Presentations with Head Injury who arrive by ambulance / air ambulance<br>Excludes: Those who self-present
Description	Usually documented in ambulance patient notes.<br>Intubation allows mechanical ventilation and support of an airway in coma.
Numerator (If Applicable)	n/a
Denominator (If Applicable)	n/a
Expressed As	Categorical

Study Sites

10.7.10 ED GCS Arrival and 2 Hours post-injury

Definition	The Glasgow Coma Score on arrival to ED and 2 hours following arrival to ED (if still in the ED at 2 hours)
Layout	NN (numeric 2 characters)
Codeset (If Applicable)	n/a
Reported For	Includes: All ED Presentations with Head Injury<br>Excludes:
Description	Usually documented in patient notes.<br>This is a discrete number out of 15 (minimum score 3, maximum score 15) given to people who have suffered a head injury. It looks at motor, verbal and eye responses to differing levels of stimulus.
Numerator (If Applicable)	n/a
Denominator (If Applicable)	n/a
Expressed As	Ordinal

Study Sites

10.7.11 ED Motor Score Adults Arrival and 2 Hours post-injury

Definition	The motor score of the patient on arrival to ED and 2 hours following arrival to ED (if still in the ED at 2 hours)
Layout	Alphanumeric
Codeset (If Applicable)	1 = Obeys Commands<br>2 = Localises to Pain<br>3 = Withdrawal from Pain<br>4 = Flexion to Pain (Decorticate)<br>5 = Extension to Pain (Decerebrate)<br>6 = No Motor Response<br>7 = Not in ED at 2 hours<br>8 = Not Recorded<br>9 = Not Available
Reported For	Includes: All ED Presentations with Head Injury<br>Excludes:
Description	Usually documented in patient notes.<br>This is the motor score from the GCS Score – however the motor score is a better predictor of outcome alone after traumatic brain injury[106].
Numerator (If Applicable)	n/a
Denominator (If Applicable)	n/a
Expressed As	Ordinal

Study Sites

10.7.12 ED Motor Score Paeds Arrival and 2 Hours post-injury

Definition	The motor score of the patient on arrival to ED and 2 hours following arrival to ED (if still in the ED at 2 hours)
Layout	Alphanumeric
Codeset (If Applicable)	1 =Obeys Commands or normal spontaneous movements<br>2 =Localises to painful stimulus or withdraws to touch<br>3 =Withdrawal to painful stimulus<br>4 =Abnormal flexion to pain (Decorticate)<br>5 =Abnormal extension to pain (Decerebrate)<br>6 =No motor response to pain<br>7 = Not in ED at 2 hours<br>8 = Not Recorded<br>9 = Not Available
Reported For	Includes: All ED Presentations with Head Injury<br>Excludes:
Description	Usually documented in patient notes.<br>This is the motor score from the GCS Score – however the motor score is a better predictor of outcome alone after traumatic brain injury.
Numerator (If Applicable)	n/a
Denominator (If Applicable)	n/a
Expressed As	Ordinal

Study Sites

10.7.13 ED Pupils Arrival and 2 Hours post-injury

Definition	The papillary examination of the patient on arrival to ED and 2 hours following arrival to ED (if still in the ED at 2 hours)
Layout	Alphanumeric
Codeset (If Applicable)	1 = Attempted but Unable to assess<br>2 = Both Pupils equal and reactive<br>3 = Pupils asymmetric but reactive<br>4 = Left Pupil dilated, unreactive<br>5 = Right Pupil dilated, unreactive<br>6 = Both Pupils dilated and unreactive<br>7 = Not in ED at 2 hours<br>8 = Not Recorded<br>9 = Not Available
Reported For	Includes: All ED Presentations with Head Injury<br>Excludes:
Description	Usually documented in patient notes.<br>This is the papillary examination of the patient. If the pupil is dilated or unreactive this suggests a third nerve palsy which in itself suggests rising intracranial pressure and/or cerebral herniation. This can also be used for prognostication of outcome following TBI[106].
Numerator (If Applicable)	n/a
Denominator (If Applicable)	n/a
Expressed As	Ordinal

Study Sites

10.7.14 Intubation in ED

Definition	Whether the patient was intubated in ED
Layout	Alphanumeric
Codeset (If Applicable)	0 = No<br>1 = Yes<br>2 = Not Recorded<br>3 = Not Available
Reported For	Includes: All ED Presentations with Head Injury<br>Excludes:
Description	Usually documented in patient notes.<br>Intubation allows mechanical ventilation and support of an airway in coma.
Numerator (If Applicable)	n/a
Denominator (If Applicable)	n/a
Expressed As	Categorical

Study Sites

10.7.15 Duration Loss of Consciousness

Definition	The length of time the patient was unconscious for.
Layout	MMM (Numeric – 3 characters)
Codeset (If Applicable)	n/a
Reported For	Includes: All ED Presentations with Head Injury<br>Excludes:
Description	Usually documented in patient notes by treating clinician.
Numerator (If Applicable)	n/a
Denominator (If Applicable)	n/a
Expressed As	Numeric

Study Sites

10.7.16 Amnesia

Definition	The presence of amnesia associated with the injury in the ED.
Layout	Alphanumeric
Codeset (If Applicable)	0 = No Amnesia<br>1= Unable to Assess<br>2 = Yes – Antegrade (of events after injury)<br>3 = Yes – Retrograde (of events before injury)<br>4 = Yes – Both Antegrade and Retrograde Amnesia<br>5 = Yes – For Event Only<br>6 = Yes – Unspecified Type<br>7 = Not Recorded<br>8 = Not Available
Reported For	Includes: All ED Presentations with Head Injury<br>Excludes:
Description	Usually documented in patient notes by treating clinician.<br>This includes retrograde amnesia (unable to remember before the incident) and antegrade amnesia (unable to remember what happened after the incident).
Numerator (If Applicable)	n/a
Denominator (If Applicable)	n/a
Expressed As	Categorical

Study Sites

10.7.17 Duration Amnesia

Definition	The length of time the patient is amnesic for.
Layout	MMM (Numeric – 3 characters)
Codeset (If Applicable)	n/a
Reported For	Includes: All ED Presentations with Head Injury<br>Excludes:
Description	Usually documented in patient notes by treating clinician.<br>This includes retrograde amnesia (unable to remember since the incident) and antegrade amnesia (unable to remember what happened before the incident).
Numerator (If Applicable)	n/a
Denominator (If Applicable)	n/a
Expressed As	Numeric

Study Sites

10.7.18 Headache since TBI

Definition	The presence of headache following the incident.
Layout	Alphanumeric
Codeset (If Applicable)	0 = No<br>1 = Unable to Assess<br>2 = Yes<br>3 = Not Recorded<br>4 = Not Available
Reported For	Includes: All ED Presentations with Head Injury<br>Excludes:
Description	Usually documented in patient notes by treating clinician.<br>If no mention of headache in the notes = “not documented”.
Numerator (If Applicable)	n/a
Denominator (If Applicable)	n/a
Expressed As	Categorical

Study Sites

10.7.19 Seizure since TBI

Definition	The occurrence of any seizure-type activity following the incident.
Layout	Alphanumeric
Codeset (If Applicable)	0 = No<br>1 = Yes<br>2 = Not Recorded<br>3 = Not Available
Reported For	Includes: All ED Presentations with Head Injury<br>Excludes:
Description	Usually documented in patient notes by treating clinician.<br>If not documented it is safe to assume a seizure hasn't happened and will therefore be recorded as a "no".
Numerator (If Applicable)	n/a
Denominator (If Applicable)	n/a
Expressed As	Categorical

Study Sites

10.7.20 Any Focal Neurological Deficit since TBI

Definition	The occurrence of any focal neurological deficit following the incident.
Layout	Alphanumeric
Codeset (If Applicable)	0 = No<br>1 = Unable to Assess<br>2 = Yes<br>3 = Not Recorded<br>4 = Not Available
Reported For	Includes: All ED Presentations with Head Injury<br>Excludes:
Description	Usually documented in patient notes by treating clinician.<br>This can mean any new neurological deficit for the patient – weakness or sensory disturbance in any limb, or deficit in any cranial nerve.
Numerator (If Applicable)	n/a
Denominator (If Applicable)	n/a
Expressed As	Categorical

Study Sites

10.7.21 Number of Vomits

Definition	The number of vomits following incident
Layout	NNNN (Numeric up to 4 characters)
Codeset (If Applicable)	
Reported For	Includes: All ED Presentations with Head Injury<br>Excludes:
Description	Usually documented in patient notes by treating / examining clinician.
Numerator (If Applicable)	n/a
Denominator (If Applicable)	n/a
Expressed As	Numeric

Study Sites

10.7.22 Mechanism of Injury

Definition	The way the patient sustained the TBI
Layout	Alphanumeric
Codeset (If Applicable)	1 = Pedestrian struck by a motor vehicle<br>2 = Occupant ejected from a motor vehicle<br>3 = Cyclist accident involving other Motor Vehicle<br>4 = Motorcyclist accident involving other Motor Vehicle<br>5 = Fall from a height of greater than one metre or five stairs<br>6 = Assault with blunt object<br>7 = High-speed motor vehicle collision<br>8 = Rollover motor vehicle accident<br>9 = Accident involving motorised recreational vehicles<br>10 = Motorcycle Accident not involving other Motor Vehicle<br>11 = Cyclist Accident not involving other Motor Vehicle<br>12 = Diving Accident<br>13 = Clinical Suspicion of NAI<br>14 = None of the above - Other<br>15 = Not Recorded<br>16 = Not Available
Reported For	Includes: All ED Presentations with Head Injury<br>Excludes:
Description	Usually documented in patient notes by treating / examining clinician.
Numerator (If Applicable)	n/a
Denominator (If Applicable)	n/a
Expressed As	Categorical

Study Sites

10.7.23 Intoxication with ETOH

Definition	If the patient was intoxicated with alcohol just prior to the incident.
Layout	Alphanumeric
Codeset (If Applicable)	0 = No<br>1 = Yes<br>2 = Not Recorded<br>3 = Not Available
Reported For	Includes: All ED Presentations with Head Injury<br>Excludes:
Description	Usually documented in patient notes by treating clinician. This means intoxication with alcohol. If there is nothing documented in the clinical notes, but there is an ETOH level taken, then of level <3mmol/L = "No", if >=3mmol/L then "Yes". If there is no mention in the notes and no ethanol level then "Not Recorded".
Numerator (If Applicable)	n/a
Denominator (If Applicable)	n/a
Expressed As	Categorical

Study Sites

10.7.24 Serum ETOH Level

Definition	The serum ethanol level for the patient on arrival to ED measured in mmol/l.
Layout	NNNN (Numeric up to 4 characters)
Codeset (If Applicable)	
Reported For	Includes: All ED Presentations with Head Injury<br>Excludes:
Description	Available from laboratory records.
Numerator (If Applicable)	n/a
Denominator (If Applicable)	n/a
Expressed As	Numeric

Study Sites

10.7.25 Intoxication with Other Recreational Substances

Definition	If the patient was intoxicated with other recreational drugs just prior to the incident.
Layout	Alphanumeric
Codeset (If Applicable)	0 = No<br>1 = Yes<br>2 = Not Recorded<br>3 = Not Available
Reported For	Includes: All ED Presentations with Head Injury<br>Excludes:
Description	Usually documented in patient notes by treating clinician.<br>This means intoxication with any recreational drug including heroin, cocaine, LSD, Speed, Ecstasy, Marijuana, GHB, “Party Pills” etc. This can be based on clinical documentation, but not urine specimens, as metabolites may be in urine from before traumatic event.
Numerator (If Applicable)	n/a
Denominator (If Applicable)	n/a
Expressed As	Categorical

Study Sites

10.7.26 Vault Skull Fracture

Definition	If the patient has a clinical skull fracture (based on clinical NOT radiological findings).
Layout	Alphanumeric
Codeset (If Applicable)	1 = Open Fracture<br>2 = Suspected Fracture<br>3 = No Obvious Fracture<br>4 = Not Recorded<br>5 = Not Available
Reported For	Includes: All ED Presentations with Head Injury<br>Excludes:
Description	Usually documented in patient notes by treating / examining clinician.<br>Documentation such as “brain tissue visible”, “fracture fragments visible to the eye” should be designated “open fracture”.<br>Documentation such as “boggy”, “boggy haematoma”, “step” or “? underlying fracture” should all be designated “suspected fracture”.<br>If minor scalp injury / contusion / haematoma (excepting Paeds), without red flags for suspected fracture then take as “no obvious fracture”.<br>If there is no mention of a head exam in the notes, then “not recorded”.
Numerator (If Applicable)	n/a
Denominator (If Applicable)	n/a
Expressed As	Categorical

Study Sites

10.7.27 Base of Skull Fracture Signs

Definition	If the patient has a clinical basal skull fracture (based on clinical NOT radiological findings).
Layout	Alphanumeric
Codeset (If Applicable)	1 = Battles Sign<br>2 = Racoon Eyes<br>3 = CSF Otorrhoea<br>4 = Haemotympanum<br>5 = CSF Rhinorrhoea<br>6 = Maxillary Shift<br>7 = Combination of Above<br>8 = None<br>9 = Not Recorded<br>10 = Not Available
Reported For	Includes: All ED Presentations with Head Injury<br>Excludes:
Description	Usually documented in patient notes by treating / examining clinician.
Numerator (If Applicable)	n/a
Denominator (If Applicable)	n/a
Expressed As	Categorical

Study Sites

10.7.28 Deterioration

Definition	Any deterioration in condition in the ED.
Layout	Alphanumeric
Codeset (If Applicable)	

0 = None
1 = GCS drop >=2 points
2 = New dilated unresponsive pupil
3 = New focal neurology
4 = Multiple reasons (related to TBI)
5 = Other
6 = Not Recorded
7 = Not Available

Reported For	Includes: All ED Presentations with Head Injury<br>Excludes:
Description	Usually documented in patient notes by treating clinician.<br>To determine appropriateness of CT scan – as it is warranted if there is any deterioration in condition.
Numerator (If Applicable)	n/a
Denominator (If Applicable)	n/a
Expressed As	Categorical

Study Sites

10.7.29 Paeds: TBI < 1 years

Definition	If the patient has any external signs of TBI (based on clinical NOT radiological findings).
Layout	Alphanumeric
Codeset (If Applicable)	0 = No external HI signs<br>1 = Presence of Bruise / Haematoma on Head<br>2= Presence of swelling on head<br>3 = Laceration >5cm on head<br>4 = Tense Fontanelle<br>5 = Not Recorded<br>6 = Not Available
Reported For	Includes: All ED Presentations with Head Injury<br>Excludes:
Description	Usually documented in patient notes by treating / examining clinician.
Numerator (If Applicable)	n/a
Denominator (If Applicable)	n/a
Expressed As	Categorical

Study Sites

10.7.30 Paeds: Specialist Referral? NAI

Definition	If the patient has been referred by ED to a Specialist Paediatrician for consideration of work-up for NAI
Layout	Alphanumeric
Codeset (If Applicable)	0 = No – Not Required<br>1 = No – Required, Not Done<br>2 = Yes<br>3 = Not Recorded<br>4 = Not Available
Reported For	Includes: All ED Presentations with Head Injury<br>Excludes:
Description	Usually documented in patient notes by treating clinician.<br>With suspicion of NAI, the NICE guidelines state these children need to have:<br>• A clinician involved with expertise in the area<br>• Skeletal survey (including skull x-ray)<br>• Ophthalmoscopic exam<br>• Exam of pallor, anaemia, tense fontanelle etc<br>These factors are not necessarily important for reducing stay in ED as these patients would usually be admitted anyway. Therefore we feel the time factor and that to determine appropriateness of care is involvement of someone with expertise
Numerator (If Applicable)	n/a
Denominator(If Applicable)	n/a
Expressed As	Categorical

Study Sites

10.7.31 CT Head Scan Request Time

Definition	The time of the first CT head Scan requested for the patient
Layout	DD/MM/CCYY HH:MM:SS (Date and Time: 18 characters separated by a space between year and hour values)
Codeset (If Applicable)	n/a
Reported For	Includes: All ED Presentations with Head Injury<br>Excludes:
Description	Usually documented in patient notes or documented on radiology ordering system (might be if electronic – this data may be hard to source).
Numerator (If Applicable)	n/a
Denominator (If Applicable)	n/a
Expressed As	Numeric (Time)

Study Sites

10.7.32 CT Head Scan Time

Definition	The time of the first CT head Scan for the patient
Layout	DD/MM/CCYY HH:MM:SS (Date and Time: 18 characters separated by a space between year and hour values)
Codeset (If Applicable)	n/a
Reported For	Includes: All ED Presentations with Head Injury<br>Excludes:
Description	Usually documented in patient notes or documented on radiological films if electronically stored.
Numerator (If Applicable)	n/a
Denominator (If Applicable)	n/a
Expressed As	Numeric (Time)

Study Sites

10.7.33 CT Scan Result (5 Fields)

Definition	The result of the CT Head Scan. There are 5 columns to record information if there is more than one finding on the CT.
Layout	Alphanumeric
Codeset (If Applicable)	0 = Normal Brain Parenchyma, No Intra or Extra Axial Injury<br>1 = Traumatic Subarachnoid Bleed<br>2 = Traumatic Subdural Haematoma - Acute, Unilateral<br>3 = Traumatic Subdural Haematoma - Acute - Bilateral<br>4 = Traumatic Subdural Haematoma - Chronic, Unilateral<br>5 = Traumatic Subdural Haematoma - Chronic, Bilateral<br>6 = Traumatic Subdural Haematoma - Mixed Age, Unilateral<br>7 = Traumatic Subdural Haematoma - Mixed Age, Bilateral<br>8 = Traumatic Extradural Haematoma<br>9 = Traumatic Intracerebral Haemorrhage<br>10 = Traumatic Intraventricular Haemorrhage<br>11 = Hydrocephalus and / or Midline Shift<br>12 = Few Cerebral Contusions<br>13 = Multiple Cerebral Contusions<br>14 = Diffuse Axonal Injury<br>15 = Skull Fracture<br>16 = Basal Skull Fracture<br>17 = None Further<br>18 = Not Done Patient Declined<br>19 = Not Recorded<br>20 = Not Available
Reported For	Includes: All ED Presentations with Head Injury<br>Excludes:
Description	Usually documented in patient notes by treating / examining clinician. Results available from radiology reports.
Numerator (If Applicable)	n/a
Denominator(If Applicable)	n/a
Expressed As	Categorical

Study Sites

10.7.34 C-Spine Imaged

Definition	If the patient has presented with TBI whether or not the C-Spine was also imaged (especially if sufficient energy in mechanism of injury or axial loading).
Layout	Alphanumeric
Codeset (If Applicable)	0 = No – Documented not clinically relevant<br>1 = No – Not documented why not<br>2 = Yes – Plain Films<br>3= Yes – CT Scan<br>4 = Not Recorded<br>5= Not Available
Reported For	Includes: All ED Presentations with Head Injury<br>Excludes:
Description	Usually documented in patient notes by treating / examining clinician.
Numerator (If Applicable)	n/a
Denominator (If Applicable)	n/a
Expressed As	Categorical

Study Sites

10.7.35 C-Spine Imaging Time

Definition	The time of the first C-Spine imaging for the patient
Layout	DD/MM/CCYY HH:MM:SS (Date and Time: 18 characters separated by a space between year and hour values)
Codeset (If Applicable)	n/a
Reported For	Includes: All ED Presentations with Head Injury<br>Excludes:
Description	Usually documented in patient notes or documented on radiological films if electronically stored.
Numerator (If Applicable)	n/a
Denominator (If Applicable)	n/a
Expressed As	Numeric (Time)

Study Sites

10.7.36 ICP Monitor Placed

Definition	If an Intracranial Pressure Monitor was placed WITHIN THE FIRST 24 HOURS.
Layout	Alphanumeric
Codeset (If Applicable)	0 = No<br>1 = Yes<br>2 = Not Recorded<br>3 = Not Available
Reported For	Includes: All ED Presentations with Head Injury<br>Excludes:
Description	Usually documented in patient notes by treating clinician.
Numerator (If Applicable)	n/a
Denominator(If Applicable)	n/a
Expressed As	Categorical

Study Sites

10.7.37 ICP Monitor Time

Definition	The time of the first Intracranial Pressure Monitor for the patient
Layout	DD/MM/CCYY HH:MM:SS (Date and Time: 18 characters separated by a space between year and hour values)
Codeset (If Applicable)	n/a
Reported For	Includes: All ED Presentations with Head Injury<br>Excludes:
Description	Usually documented in patient notes.
Numerator (If Applicable)	n/a
Denominator (If Applicable)	n/a
Expressed As	Numeric (Time)

Study Sites

10.7.38 Neurosurgical Consultation

Definition	If Neurosurgical consultation was undertaken at any stage during the ED stay.
Layout	Alphanumeric
Codeset (If Applicable)	0 = No<br>1 = Yes<br>2 = Not Recorded<br>3 = Not Available
Reported For	Includes: All ED Presentations with Head Injury<br>Excludes:
Description	Usually documented in patient notes by treating clinician.
Numerator (If Applicable)	n/a
Denominator(If Applicable)	n/a
Expressed As	Categorical

Study Sites

10.7.39 Neurosurgical Referral or Transfer

Definition: Reason given for transfer or referral to Neurosurgical services.
Layout: Alphanumeric
Codeset (If Applicable):

0 = No referral or transfer
1 = Severe TBI on arrival (GCS<=8)
2 = CT proven TBI (Non-operative Mx)
3 = CT proven TBI (Operative Mx)
4 = Isolated Skull Fracture
5 = CSF Leak
6 = Drop in GCS > 2 points while in ED
7 = Development of pupil dilatation while in ED
8 = Development of new neurological deficit while in ED
9 = Development of progressive neurological deficit in ED
10 = Unexplained confusion lasting more than 4 hours
11 = Seizure without full recovery
12 = Combination of Above
13 = Not Recorded
14 = Not Available

Reported For: Includes: All ED Presentations with Head Injury
Excludes:
Description: Usually documented in patient notes by treating clinician.

Numerator (If Applicable): n/a
Denominator(If Applicable): n/a
Expressed As: Categorical

Study Sites

10.7.40 To OR

Definition	If the patient proceeded to the Operating Room for a Neurosurgical procedure.
Layout	Alphanumeric
Codeset (If Applicable)	0 = No<br>1 = Yes - from ED<br>2 = Yes - transferred to other Neurosurgical Centre<br>3 = Yes - from Ward, following observation<br>4 = Not Recorded<br>5 = Not Available
Reported For	Includes: All ED Presentations with Head Injury<br>Excludes:
Description	Usually documented in patient notes by treating clinician.<br>We may not be able to capture surgical time for those transferred for surgical management to another hospital – North Shore, Middlemore and Waitakere possibly yes (from Auckland notes), Waikato yes (transfers to their NS Unit from outside the DHB are excluded) but Hawkes Bay no.
Numerator (If Applicable)	n/a
Denominator(If Applicable)	n/a
Expressed As	Categorical

Study Sites

10.7.41 Surgery Start Time

Definition	The time of the start of first emergency neurosurgery for the patient.
Layout	DD/MM/CCYY HH:MM:SS (Date and Time: 18 characters separated by a space between year and hour values)
Codeset (If Applicable)	n/a
Reported For	Includes: All ED Presentations with Head Injury<br>Excludes:
Description	Usually documented in patient notes.
Numerator (If Applicable)	n/a
Denominator (If Applicable)	n/a
Expressed As	Numeric (Time)

Study Sites

10.7.42 Type of OR Procedure

Definition	The Neurosurgical procedure carried out.
Layout	Alphanumeric
Codeset (If Applicable)	0 =No Procedure<br>1 = Burr Hole<br>2 = Craniotomy<br>3 = Craniectomy<br>4 = Not Recorded<br>5 = Not Available
Reported For	Includes: All ED Presentations with Head Injury<br>Excludes:
Description	Usually documented in patient notes by treating clinician.<br>We may not be able to capture surgical procedure for those transferred for surgical management to another hospital – North Shore, Middlemore and Waitakere possibly yes (from Auckland notes), Waikato yes (transfers to their NS Unit from outside the DHB are excluded) but Hawkes Bay no.
Numerator (If Applicable)	n/a
Denominator(If Applicable)	n/a
Expressed As	Categorical

Study Sites

10.8 Discharge Information

The decision was made to capture the quality of discharge information (ED to Primary Care physician). This was chosen to represent the theme of communication which arose during a forum to validate the quality markers being used on this project. It was felt this may is an important aspect for the quality of care of the patient and is part of appropriate handover to the physician taking over ongoing care.

The Joint Commission (US) requires that (generic) discharge summaries include the following elements:

- The reason for hospitalisation
- Significant findings
- Procedures performed
- Treatment provided
- Patients condition at discharge
- Information provided to the patient (and family)

The above is based on Standard 6.10: Hospital Accreditation Standards 2006[107] "the hospital has a complete and accurate medical record for patients assessed, cared for, treated or served".

A literature review by Kripalani et al[108] looked at observational studies investigating communication and information transfer at discharge from hospital. They surmised that Primary Care Physicians generally rate the following as most important discharge documentation to be able to provide adequate follow-up care:

- Main Diagnosis
- Pertinent Physical Findings
- Results of Procedures
- Results of Investigations
- Discharge Medications and reasons for change in medications if any
- Details of follow-up arrangements
- Information given to patients and family
- Test results pending at discharge (and who is to chase these): missing from 65% of discharge summaries in review.
- Specific follow-up needs

They also suggested the use of pre-formatted standardised electronic computer-generated discharge summaries that may facilitate timely and accurate transfer of discharge information. Currently most hospitals in New Zealand use an electronic format for discharge summaries.

The ACEM policy document on “Components of an Emergency Medicine Consultation[109]” states that for patients discharged from ED there is a process of:

- Pre-Discharge Screening
  - Suitability for Discharge
  - Safety for Discharge
- Discharge Medications :
  - An explanation of the discharge medications provided
  - Possible adverse effects discussed
- Discharge Instructions:
  - Measures to be taken to assist in treatment
  - Timing and Service involved in the scheduled review of their condition
  - Instructions as to when to seek unscheduled review
  - Written discharge instructions where relevant
  - Documentation of above in notes
- Discharge Communication of Diagnosis and Management plan to relevant care provider

Jansen et al[110] defined an Emergency Department “gold standard” discharge letter as containing the following:

- Accurate primary diagnosis
- Relevant secondary diagnosis
- Concise summary of management
  - Details of minor procedures if relevant to follow-up
- Hospital follow-up arrangements (if any)
- Any issues (including pending tests, social) requiring follow-up or action by GP

They have also defined the overall quality of correspondence:
“Satisfactory: All necessary information relevant to patient’s further care included
Lacking: Inaccurate Diagnosis, missing detail regarding management of follow-up
Unacceptable: Wrong diagnosis, dangerously misleading content”.
Nearly 50% of the 300 discharge summaries they reviewed had the wrong or an inaccurate diagnosis.

Taylor and Cameron[111] state “all patients discharged home from the emergency department should be given instructions for the ongoing management of their illness”. Their essential elements of a discharge summary (based on author consideration, patient and medico-legal requirements and a paucity of literature) are:

- General Features:
  - Patient Name
  - Physician Name

- Illness Related Features:
  - Diagnosis
  - Expected course of illness
  - Potential complications of illness

- Patient Instructions:
    - General instructions for the management of the illness
    - Medication Prescribed
        - Name
        - Dose
        - Frequency
        - Purpose
        - Complications or side effects
        - Any alteration in usual drug regimen
    - Advice on follow-up
        - Service (with whom)
        - Appropriate time
        - Advised review in ED in the event of serious complications
    - Medico-legal
        - Date and time on summary

In a survey of GP's, Wass et al[112] found the following information was important to GP's on the discharge summary for ED patients:

- Patients Presenting Complaint
- Investigations done
- Results of Investigations
- Diagnosis made in ED
- Treatment given in ED
- Follow-up Arrangements
- Speciality under which the patient was admitted
- Issue of a sick note

The above information has been used to develop the following components of the discharge summary to be measured, and ways of assessing the adequacy of information.

10.8.1 Discharge Letter Done

Definition	Whether or not the patient has a recorded discharge letter in the clinical notes.
Layout	N (Number: 1 Characters)
Codeset (If Applicable)	0 = No<br>1 = Yes<br>2 = Left Without Being Seen<br>3 = Not available
Reported For	Includes: All Patients discharged from ED or inpatient teams<br>Excludes:
Description	Should be accessible from electronic records and also paper patient records.
Numerator (If Applicable)	n/a
Denominator (If Applicable)	n/a
Expressed As	Categorical

Study Sites

10.8.2 Discharge Letter Date

Definition	The date (and time, if recorded) the discharge letter was written and finalized. If there is no date / time on the letter then this field will be blank.
Layout	DD/MM/CCYY HH:MM:SS (Date and Time: 18 characters separated by a space between year and hour values)
Codeset (If Applicable)	n/a
Reported For	Includes: All Events discharged from ED<br>Excludes: Those with no discharge letter, DNW, DOA
Description	Usually documented on the discharge letter when written electronically – this does not change of the letter has been modified, but the date and time of modification is recorded. This will be simple to capture of discharge letter in electronic format, however may be more difficult if written letters have been used.<br><br>**This is not the date and time the patient was discharged, but the date and time the letter was written.**
Numerator (If Applicable)	n/a
Denominator (If Applicable)	n/a
Expressed As	Numeric (Time)

Study Sites

10.8.3 Discharge Diagnosis

Definition	Whether or not the patient has Diagnosis Information recorded on the discharge letter.
Layout	N (Number: 1 Characters)
Codeset (If Applicable)	1 = Adequate<br>2 = Inadequate<br>3 = Unacceptable<br>4 = Not Available
Reported For	Includes: All Events discharged from ED or seen in ED and discharged after inpatient stay.<br>Excludes:
Description	Should be accessible from electronic records and also paper patient records. Adequacy based on:<br><br>Adequate = Correct Primary Diagnosis and some detail on relevant secondary diagnoses.<br><br>Inadequate = Inaccurate Primary Diagnosis (if it is a “non-specific” term or differs to clinical notes) and / or inaccurate, irrelevant or missing detail on secondary diagnosis information<br><br>Unacceptable = Wrong primary diagnosis or no primary diagnosis recorded on summary.<br><br>Not Available = notes for event not available.<br><br>This is applicable for all patient discharges. For self discharges if the patient has not been seen by a physician, no discharge summary may be adequate.
Numerator (If Applicable)	n/a
Denominator (If Applicable)	n/a
Expressed As	Categorical

Study Sites

10.8.4 Discharge Treatment Information

Definition	Whether or not the patient has ED or Hospital treatment information recorded on the discharge letter.
Layout	N (Number: 1 Characters)
Codeset (If Applicable)	1 = Adequate<br>2 = Inadequate<br>3 = Unacceptable<br>4 = Not Available
Reported For	Includes: All Events discharged from ED or seen in ED and discharged after inpatient stay.<br>Excludes:
Description	Should be accessible from electronic records and also paper patient records. Treatment involves non-surgical management of illness, including medications given to ameliorate or treat disease. Adequacy based on:<br><br>Adequate = Correct and concise documentation of all treatments delivered in ED or as an inpatient, or documentation of no need for treatment, or no documentation when no treatment given.<br><br>Inadequate = Incomplete treatment information from ED or inpatient stay (only some aspects of treatment documented – but not likely to be harmful)<br><br>Unacceptable = Wrong treatment information or missing treatment information when treatment given (likely to be harmful).<br><br>Not Available = notes for event not available.
Numerator (If Applicable)	n/a
Denominator (If Applicable)	n/a
Expressed As	Categorical

Study Sites

10.8.5 Treatment Complications Information

Definition	Whether the patient has ED or Hospital treatment complications information recorded on the discharge letter.
Layout	N (Number: 1 Characters)
Codeset (If Applicable)	1 = Adequate<br>2 = Inadequate<br>3 = Unacceptable<br>4 = Not Available
Reported For	Includes: All Events discharged from ED or seen in ED and discharged after inpatient stay.<br>Excludes:
Description	Should be accessible from electronic records and also paper patient records. Treatment involves non-surgical management of illness, including medications given to ameliorate or treat disease. Complications of these include allergic reactions, wrong medications or doses given and side-effects of treatment (for example GI Bleed or renal failure). Adequacy based on:<br><br>Adequate = Correct and concise documentation of most treatment complications in ED or as an inpatient, or documentation of no complications of treatment, or no complication occurred, therefore not necessary to document this in discharge summary, or no treatment given.<br><br>Inadequate = Incomplete treatment complication information from ED or inpatient stay (not likely to be harmful if treatment repeated)<br><br>Unacceptable = Wrong treatment complication information or not documented when a complication happened (likely to cause harm if treatment were subsequently repeated).<br><br>Not Available = notes for event not available.
Numerator (If Applicable)	n/a
Denominator (If Applicable)	n/a
Expressed As	Categorical

Study Sites

10.8.6 Procedures Information

Definition	Whether or not the patient has ED or Hospital procedures information recorded on the discharge letter.
Layout	N (Number: 1 Characters)
Codeset (If Applicable)	1 = Adequate<br>2 = Inadequate<br>3 = Unacceptable<br>4 = Not Available
Reported For	Includes: All Events discharged from ED or seen in ED and discharged after inpatient stay.<br>Excludes:
Description	Should be accessible from electronic records and also paper patient records. Procedures involve any invasive procedure carried out on the patient. Adequacy based on:<br><br>Adequate = Correct and concise documentation of all procedures carried out in ED or as an inpatient (does not need to include venepuncture or peripheral intravenous cannulation), or not recorded when no procedure done.<br><br>Inadequate = Inaccurate or incomplete procedure information from ED or inpatient stay (e.g. some but not all relevant procedures documented)<br><br>Unacceptable = Wrong procedure information, no procedure documented when procedure occurred.<br><br>Not Available = notes for event not available.
Numerator (If Applicable)	n/a
Denominator (If Applicable)	n/a
Expressed As	Categorical

Study Sites

10.8.7 Procedure Complications Information

Definition	Whether or not the patient has ED or Hospital procedure complications information recorded on the discharge letter.
Layout	N (Number: 1 Characters)
Codeset (If Applicable)	1 = Adequate<br>2 = Inadequate<br>3 = Unacceptable<br>4 = Not Available
Reported For	Includes: All Events discharged from ED or seen in ED and discharged after inpatient stay.<br>Excludes:
Description	Should be accessible from electronic records and also paper patient records. Procedures involve any invasive procedure carried out on the patient. Complications of these include IV site infection, wrong operative site, post-op wound infection, unscheduled return to OR, failure of procedure etc. Adequacy based on:<br><br>Adequate = Correct and concise documentation of most procedure complications in ED or as an inpatient, or documentation of no complications of procedures, or no procedures carried out, or not recorded if no complication of the procedure.<br><br>Inadequate = Inaccurate or incomplete procedure complication information from ED or inpatient stay (not likely to cause harm if procedure subsequently repeated)<br><br>Unacceptable = Wrong procedure complication information or no procedure complication information documented if a complication happened (likely to cause harm if subsequently repeated).<br><br>Not Available = notes for event not available.
Numerator (If Applicable)	n/a
Denominator (If Applicable)	n/a
Expressed As	Categorical

Study Sites

10.8.8 GP-Specific Ongoing Care Information

Definition	Whether or not the patient has a need for ongoing care (whilst in the community) and information relaying such to the GP recorded on the discharge letter.
Layout	N (Number: 1 Characters)
Codeset (If Applicable)	1 = Adequate<br>2 = Inadequate<br>3 = Unacceptable<br>4 = Not Available
Reported For	Includes: All Events discharged from ED or seen in ED and discharged after inpatient stay.<br>Excludes:
Description	Should be accessible from electronic records and also paper patient records. Ongoing care advice includes:<br>• Details of hospital follow-up arrangements (service and timing)<br>• Test results pending at discharge (and who is to chase these)<br>• Specific follow-up needs for GP to arrange (i.e. organizing further investigations)<br><br>Adequate = all of the points above recorded if applicable, or documentation that the patient does not need to be followed up, or no need for ongoing care.<br><br>Inadequate = inaccurate or incomplete (missing points above if they are applicable) ongoing care information, unlikely to cause harm if not documented.<br><br>Unacceptable = wrong or misleading information given to the GP regarding ongoing care or none documented when ongoing care required (likely to cause harm if not documented).<br><br>Not Available = notes for event not available.<br><br>If the patient has self discharged, adequacy will be based on discharge letter as per criteria above; as it is important the GP is aware of this, however if they have not been seen by a physician this may be adequate.
Numerator (If Applicable)	n/a
Denominator (If Applicable)	n/a
Expressed As	Categorical

Study Sites

10.8.9 Patient-Specific Ongoing Care Information

Definition	Whether or not the patient has discharge information recorded on the discharge letter.
Layout	N (Number: 1 Characters)
Codeset (If Applicable)	1 = Adequate<br>2 = Adequate - self D/C attempt to Contact<br>3 = Inadequate<br>4 = Inadequate – self D/C no attempt to Contact<br>5 = Unacceptable<br>6 = Not Available
Reported For	Includes: All Events discharged from ED<br>Excludes:
Description	Should be accessible from electronic records and also paper patient records. Discharge information should include:<br>• Advice on diagnosis<br>• Detail about expectations for course of recovery<br>• Potential Complications<br>• Guidelines for management of the illness<br><br>Adequate = all of above points covered on discharge summary. The information can be documented as either an instruction note in the discharge summary or as an information handout. Either of these is adequate as long as they include all of the above points. All patients should have some information.<br>Adequate, self discharge with attempt to contact = Patient has self-discharged without notifying healthcare professionals, and an attempt has been made to contact the patient and provide specific advice in information.<br>Inadequate = inaccurate or incomplete (missing points above if they are applicable) patient-specific information.<br>Inadequate, self discharge with no attempt to contact = Patient has self-discharged without notifying healthcare professionals and no attempt has been made to contact the patient and provide specific advice in information<br>Unacceptable = Patient-specific information wrong, not relevant to case or none documented.<br>Not Available = notes for event not available.
Numerator (If Applicable)	n/a
Denominator (If Applicable)	n/a
Expressed As	Categorical

Study Sites

10.8.10 Discharge Medication Information

Definition	Whether or not the patient has information recorded on the discharge letter in relation to their discharge medications.
Layout	N (Number: 1 Characters)
Codeset (If Applicable)	1 = Adequate<br>2 = Adequate - self D/C attempt to Contact<br>3 = Inadequate<br>4 = Inadequate – self D/C no attempt to Contact<br>5 = Unacceptable<br>6 = Not Available
Reported For	Includes: All Events discharged from ED<br>Excludes:
Description	Should be accessible from electronic records and also paper patient records. This information details information on the medications prescribed on discharge it should include:<br>• Name<br>• Dose<br>• Frequency<br>• Purpose – in relation to current issue<br>• Potential complications or side effects<br>• Any alteration in usual drug regimen<br>When no medication documented on d/c summary this shall be:<br>Adequate if no medication prescribed when not indicated (minor injury and illness, mild pain only, or patient declines).<br>Otherwise this could be regarded as inadequate or unacceptable as per the definitions below.<br>Adequate = all of above points covered on discharge summary (if applicable). The last point may not always be applicable in ED. The second last may be covered verbally. Medication appropriate and prescribed in relation to current illness and illness severity.<br>Adequate, self discharge with attempt to contact = Patient has self-discharged without notifying healthcare professionals and has not been given prescription as no opportunity to do so.<br>Inadequate = inaccurate or incomplete (missing points above if they are applicable) discharge medication information. Medication not prescribed in relation to current illness or illness severity, but unlikely to cause harm.<br>Unacceptable = wrong medication information (wrong dose, wrong medication), not prescribed in relation to current illness or illness severity and likely to cause harm.<br>Not Available = notes for event not available.<br>If patient self discharges and is given a prescription, the adequacy of the medication information is judged as per if they had not self-discharged.
Numerator (If Applicable)	n/a
Denominator (If Applicable)	n/a
Expressed As	Categorical

Study Sites

10.8.11 Review Information

Definition	Whether or not the patient has information recorded on the discharge letter as to when their next expected medical review should be.
Layout	N (Number: 1 Characters)
Codeset (If Applicable)	1 = Adequate<br>2 = Inadequate<br>3 = Unacceptable<br>4 = Not Available
Reported For	Includes: All Events discharged from ED<br>Excludes:
Description	Should be accessible from electronic records and also paper patient records. This information details when to seek medical advice again, it should include:<br>• Service (with whom)<br>• Specific timeline for follow-up<br>• Advised review with GP if needed<br>• Advised review in ED in the event of serious complications<br><br>Adequate = any of above points covered on discharge summary and documented if applicable.<br><br>Inadequate = Incomplete medical review information.<br><br>Unacceptable = wrong or unrecorded medical review information<br><br>Not Available = notes for event not available.
Numerator (If Applicable)	n/a
Denominator (If Applicable)	n/a
Expressed As	Categorical

Study Sites

10.8.12 Overall Adequacy Discharge Information

Definition	The overall adequacy of discharge information as compared to previous studies.
Layout	N (Number: 1 Characters)
Codeset (If Applicable)	1 = Adequate<br>2 = Inadequate<br>3 = Unacceptable<br>4 = Not Available
Reported For	Includes: All Events discharged from ED<br>Excludes:
Description	• Discharge Date<br>• Discharge Diagnosis<br>• Treatment Information<br>• Treatment Complications Information<br>• Procedure Information<br>• Procedure Complications Information<br>• Ongoing Care Information for GP<br>• Patient-Specific Information<br>• Discharge Medication Information<br>• Medical Review Information<br><br>Adequate = Discharge Diagnosis must be adequate. To be overall adequate the discharge summary must have scored adequate in all 10 of the components.<br><br>Inadequate = less than 10 out of 10 points<br><br>Unacceptable = any point rated as unacceptable – this is something that may have the potential to cause harm to the patient, or no discharge summary completed for event.<br><br>Not Available = notes for event not available.
Numerator (If Applicable)	n/a
Denominator (If Applicable)	n/a
Expressed As	Categorical

Study Sites

10.8.13 Patient-Specific Information – Clinical Notes

Definition	Whether or not the patient has discharge information recorded in the clinical notes prior to discharge.
Layout	N (Number: 1 Characters)
Codeset (If Applicable)	1 = Adequate<br>2 = Adequate - self D/C attempt to Contact<br>3 = Inadequate<br>4 = Inadequate – self D/C no attempt to Contact<br>5 = Unacceptable<br>6 = Not Available
Reported For	Includes: All Events discharged from ED<br>Excludes:
Description	Should be accessible from electronic records and also paper patient records. Discharge information recorded in the notes is important from a medico-legal point of view and is also covered by an ACEM policy document for ED discharges[109]. This should include:<br>• Advice on diagnosis<br>• Detail about expectations for course of recovery<br>• Potential Complications<br>• Guidelines for management of the illness<br>Adequate = all of above points covered in clinical notes. The information is documented as either verbal instructions (but must be documented what the patient was told), an instruction note or as an information handout. Either of these is adequate as long as they include all of the above points. All patients should have some information.<br>Adequate, self discharge with attempt to contact = Patient has self-discharged without notifying healthcare professionals, and an attempt has been made to contact the patient and provide specific advice in information over the phone, then this is documented in the clinical notes<br>Inadequate = inaccurate or incomplete (missing points above if they are applicable) patient-specific information.<br>Inadequate, self discharge with no attempt to contact = Patient has self-discharged without notifying healthcare professionals and no attempt has been made to contact the patient and provide specific advice in information.<br>Unacceptable = Patient-specific information wrong, not relevant to case or none documented.<br>Not Available = notes for event not available.
Numerator (If Applicable)	n/a
Denominator (If Applicable)	n/a
Expressed As	Categorical

Study Sites

11.0 Appendices

11.1 Appendix 1: Example Data Source Chart – for greater detail see DHB's Document

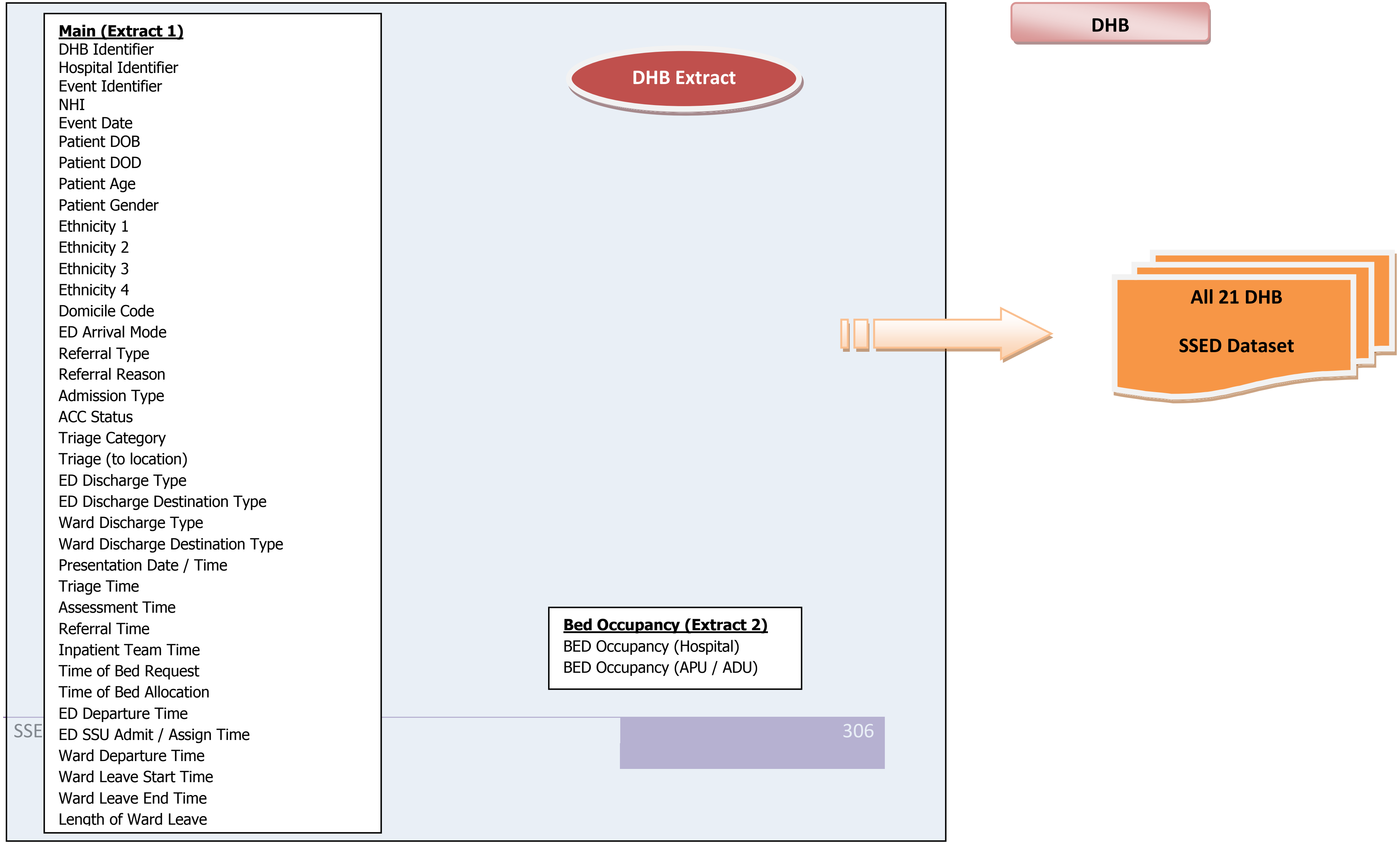

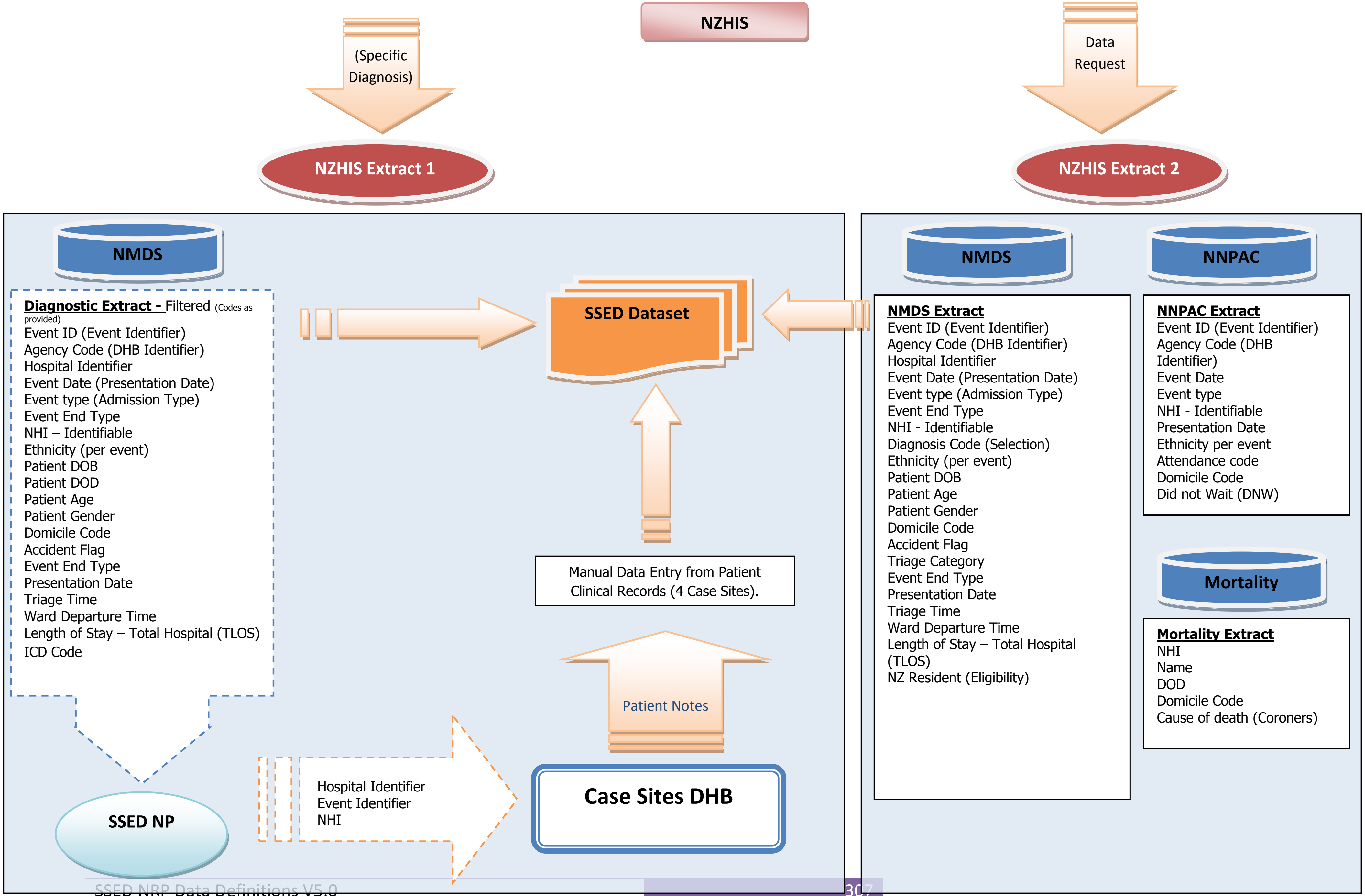
NZHIS
(Specific Diagnosis)
Data Request
NZHIS Extract 1
NZHIS Extract 2
NMDS
Diagnostic Extract - Filtered (Codes as provided)
Event ID (Event Identifier)
Agency Code (DHB Identifier)
Hospital Identifier
Event Date (Presentation Date)
Event type (Admission Type)
Event End Type
NHI – Identifiable
Ethnicity (per event)
Patient DOB
Patient DOD
Patient Age
Patient Gender
Domicile Code
Accident Flag
Event End Type
Presentation Date
Triage Time
Ward Departure Time
Length of Stay – Total Hospital (TLOS)
ICD Code
SSED NP
SSED Dataset
Manual Data Entry from Patient Clinical Records (4 Case Sites).
Patient Notes
Hospital Identifier
Event Identifier
NHI
Case Sites DHB
NMDS
NNPAC
NMDS Extract
Event ID (Event Identifier)
Agency Code (DHB Identifier)
Hospital Identifier
Event Date (Presentation Date)
Event type (Admission Type)
Event End Type
NHI - Identifiable
Diagnosis Code (Selection)
Ethnicity (per event)
Patient DOB
Patient Age
Patient Gender
Domicile Code
Accident Flag
Triage Category
Event End Type
Presentation Date
Triage Time
Ward Departure Time
Length of Stay – Total Hospital (TLOS)
NZ Resident (Eligibility)
NNPAC Extract
Event ID (Event Identifier)
Agency Code (DHB Identifier)
Event Date
Event type
NHI - Identifiable
Presentation Date
Ethnicity per event
Attendance code
Domicile Code
Did not Wait (DNW)
Mortality
Mortality Extract
NHI
Name
DOD
Domicile Code
Cause of death (Coroners)

11.2 Appendix 2: ICD-10-AM Classification of Diseases Codes

For Quality Indicators (Study Sites)

From: **"The International Statistical Classification of Diseases and Related Health Problems, Australian Modification (Tenth Revision). Sixth Edition July 2008"**

Myocardial Infarction*: Diagnosis Codes*

I21 Acute Myocardial Infarction

I21.0 Acute Transmural Myocardial Infarction of anterior wall (STEMI)

I21.1 Acute Transmural Myocardial Infarction of inferior wall (STEMI)

I21.2 Acute Transmural Myocardial Infarction of other sites (STEMI includes apical-lateral, basal-lateral, high lateral, lateral, posterior, posterobasal, posterolateral, posteroseptal, septal)

I21.3 Acute Transmural Myocardial Infarction of unspecified site (STEMI)

I21.9 Acute Myocardial Infarction, unspecified

Appendicitis*: Diagnosis Codes*

K35 Diseases of Appendix

K35.0 Acute Appendicitis with generalised peritonitis (Appendicitis, acute with perforation, rupture or peritonitis following perforation or rupture)

K35.1 Acute Appendicitis with peritoneal abscess (Appendiceal abscess)

K35.9 Acute Appendicitis, unspecified (acute appendicitis with localised peritonitis, acute appendicitis without generalised peritonitis, perforation, abscess or rupture)

K36 Other Appendicitis (chronic or recurrent)

K37 Unspecified Appendicitis

Fracture Neck of Femur*: Diagnosis Codes*

S72 Fracture Neck of Femur

S72.0 Fracture Neck of Femur

S72.00 Fracture of neck of femur, part unspecified

S72.01 Fracture of intracapsular section of femur

S72.02 Fracture of upper epiphysis (separation) of femur

S72.03 Fracture of subcapital section of femur

S72.04 Fracture of midcervical section of femur (transcervical)

S72.05 Fracture of base of neck of femur

S72.08 Fracture of other parts of neck of femur (fracture of hip NOS, head or femur)

S72.1 Pertrochanteric Fracture

S72.10 Fracture of trochanteric section of femur, unspecified

S72.11 Fracture of intertrochanteric section of femur

Asthma*: Diagnosis Codes*

J45 Asthma

J45.0 Predominantly allergic asthma

J45.1 Non-allergic asthma

J45.8 Mixed Asthma

J45.9 Asthma, unspecified

J46 Status Asthmaticus (severe asthma)

***Sepsis and Septic Shock**: Diagnosis Codes*

A39 Meningococcal Infection

- A39.0 Meningococcal Meningitis
- A39.1 Waterhouse-Friderichsen Syndrome
- A39.2 Acute Meningococcaemia
- A39.3 Chronic Meningococcaemia
- A39.4 Meningococcaemia, unspecified
- A39.5 Meningococcal Heart Disease
- A39.8 Other Meningococcal Infections
- A39.9 Meningococcal infection, unspecified

A40 Streptococcal Sepsis

- A40.0 Sepsis due to Streptococcus group A
- A40.1 Sepsis due to Streptococcus group B
- A40.2 Sepsis due to Streptococcus group D
- A40.3 Sepsis due to Streptococcus Pneumoniae
- A40.8 Other Streptococcal Sepsis
- A40.9 Streptococcal Sepsis, unspecified

A41 Other Sepsis

- A41.0 Sepsis due to Staphylococcus aureus
- A41.1 Sepsis due to other specified Staphylococcus
- A41.2 Sepsis due to unspecified Staphylococcus
- A41.3 Sepsis due to Haemophilus Influenzae
- A41.4 Sepsis due to anaerobes
- A41.5 Sepsis due to other and unspecified gram-negative organisms
    - A41.50 Sepsis due to Escherichia Coli (E-Coli)
    - A41.51 Sepsis due to Pseudomonas
    - A41.58 Sepsis due to other Gram-Negative organisms

A41.8 Other specified sepsis

A41.9 Sepsis, unspecified (includes septic shock and septicaemia)

A48 Other Bacterial Diseases

A48.0 Gas Gangrene (clostridia)

A48.3 Toxic Shock Syndrome

A49 Bacterial Infection unspecified site

A49.9 Bacterial Infection, unspecified (Bacteraemia)

A42 Actinomycosis

A42.7 Actinomycotic Sepsis

A22 Anthrax

A22.7 Anthrax Sepsis

B37 Candidal Infection

B37.7 Candidal Sepsis

A26 Erysipeloid

A26.7 Erysipelothrix Sepsis

A28 Other zoonotic bacterial disease, not elsewhere classified

A28.2 Extra intestinal yersiniosis

A54 Gonococcal Infection

A54.8 Other Gonococcal Infections (Sepsis)

B00 Herpesvirus Infection

B00.7 Disseminated herpesviral disease (herpesvirus sepsis)

A32 Listeria Infection

A32.7 Listerial Sepsis

P36 Bacterial Sepsis of Newborn (includes congenital septicaemia)

P36.0 Sepsis of newborn due to streptococcus group B

P32.1 Sepsis of newborn due to other and unspecified streptococci

P36.2 Sepsis of newborn due to staph aureus

P36.3 Sepsis of newborn due to other and unspecified staphylococci

P36.4 Sepsis of newborn due to E-Coli

P36.5 Sepsis of newborn due to anaerobes

P36.8 Other bacterial Sepsis of newborn

P36.9 Bacterial Sepsis of newborn, unspecified

P37 Other Congenital an infectious diseases newborn

P37.2 Neonatal Disseminated Listeriosis

P37.52 Invasive Neonatal candidiasis (generalised neonatal Candidal sepsis)

T81 Complications of procedures, not elsewhere classified

T81.1 Shock during or resulting from a procedure, not elsewhere classified (septic shock post-procedural)

T81.4 Infection following a procedure, not elsewhere classified

T81.42 Sepsis following a procedure

O85 Puerperal Sepsis

A21 Tularaemia

A21.7 Generalised Tularaemia (Tularaemic Sepsis)

A24 Glanders and Melioidosis

A24.1 Acute and fulminating melioidosis (pneumonia, sepsis, septicaemia)

A20 Plague

A20.7 Septicaemic Plague

R57 Shock, not elsewhere classified

R57.8 Other Shock (Endotoxic Shock)

***Traumatic Brain Injury**: Diagnosis Codes*

S02 Fracture of Skull and Facial Bones

- S02.0 Fracture of Vault of Skull
- S02.1 Fracture of Base of Skull
- S02.7 Multiple fractures involving skull and facial bones
- S02.9 Fracture of Skull and Facial bones, part unspecified.

S06 Intracranial Injury

- S06.0 Concussive Injury
  - S06.00 Concussion
  - S06.01 Loss of consciousness of unspecified duration
  - S06.02 Loss of consciousness of brief duration (less than 30 minutes)
  - S06.03 Loss of consciousness of moderate duration (30 minutes to 24 hours)
  - S06.04 Loss of consciousness of prolonged duration (> 24 hours), with return to pre-existing conscious level
  - S06.05 Loss of consciousness of prolonged duration (> 24 hours), without return to pre-existing conscious level
- S06.1 Traumatic Cerebral Oedema
- S06.2 Diffuse Brain Injury
  - S06.20 Diffuse cerebral and cerebellar brain injury, unspecified.
  - S06.21 Diffuse cerebral contusions
  - S06.22 Diffuse cerebellar contusions
  - S06.23 Multiple intracerebral and cerebellar haematomas
  - S06.28 Other diffuse cerebral and cerebellar injury
- S06.3 Focal Brain Injury
  - S06.30 Focal cerebral and cerebellar injury, unspecified
  - S06.31 Focal cerebral contusion
  - S06.32 Focal cerebellar contusion
  - S06.33 Focal cerebral haematoma

S06.34 Focal cerebellar haematoma

S06.38 Other focal cerebral and cerebellar injury

S06.4 Epidural Haemorrhage (Extradural Haemorrhage)

S06.5 Traumatic Subdural Haemorrhage

S06.6 Traumatic Subarachnoid Haemorrhage

S06.8 Other intracranial injuries

S06.9 Intracranial injury, unspecified

S09.7 Multiple injuries to the head

S09.8 Other specified injuries to the head

11.3 Appendix 3: ACHI Classification of Procedure Codes

Australian Classification of Health Interventions (Tabular List) – Sixth Edition July 2008

Myocardial Infarction: *Procedure Codes:*

667 Cardiac Catheterisation

38200-00 R heart catheterisation

38203-00 L heart catheterisation

38206-00 R and L heart catheterisation

668 Coronary Angiography

38215-00 Coronary Angiography

38215-00 Coronary Angiography L heart catheterisation

38218-01 Coronary Angiography R heart catheterisation

38218-02 Coronary Angiography R and L heart catheterisation

669 Excision Procedures on Coronary Arteries

38309-00 Percutaneous Transluminal coronary rotational atherectomy (PTCRA), 1 artery

38312-00 Percutaneous Transluminal coronary rotational atherectomy (PTCRA), 1 artery with insertion of 1 stent

38312-01 Percutaneous Transluminal coronary rotational atherectomy (PTCRA), 1 artery with insertion of >=2 stents

38315-00 Percutaneous Transluminal coronary rotational atherectomy (PTCRA), multiple arteries

38318-00 Percutaneous Transluminal coronary rotational atherectomy (PTCRA), multiple arteries with insertion of 1 stent

38318-01 Percutaneous Transluminal coronary rotational atherectomy (PTCRA), multiple arteries with insertion of >=2 stents

670 Transluminal Coronary Angioplasty

38300-00 Percutaneous Transluminal balloon angioplasty of 1 coronary artery

38303-00 Percutaneous Transluminal balloon angioplasty of >=2 coronary arteries

671 Transluminal Coronary Angioplasty with Stenting

38306-00 Percutaneous Insertion of one Transluminal stent into a single coronary artery

38306-01 Percutaneous Insertion of >=2 Transluminal stents into a single coronary artery

38306-02 Percutaneous Insertion of >=2 Transluminal stents into multiple coronary arteries

***Appendectomy**: Procedure Codes*

926 Appendicectomy

30572-00 Laparoscopic Appendicectomy

30571-00 Appendicectomy (includes Incidental Appendicectomy)

1974 Other radiography of chest

58500-00 Radiography of chest

1977 Radiography of abdomen or peritoneum

58900-00 Radiography of abdomen

1943 Ultrasound of abdomen or pelvis

55036-00 Ultrasound of abdomen

1963 Computerised Tomography of abdomen and pelvis

56501-00 CT of Abdomen and Pelvis

56507-00 CT of Abdomen and Pelvis with IV contrast

Fracture Neck of Femur: *Procedure Codes*

1479 Fixation of fracture of pelvis or femur

47519-00 Internal fixation of fracture of trochanteric or subcapital femur

1486 Reduction of fracture of pelvis or femur

47516-01 Closed reduction of fracture of femur

1488 Bone graft to pelvis or hip

48200-00 Bone graft to femur

48203-00 Bone graft to femur with internal fixation

1489 Arthroplasty of hip

47522-00 Hemi-arthroplasty of femur

49312-00 Excision arthroplasty of hip

49315-00 Partial arthroplasty of hip

49318-00 Total arthroplasty of hip, unilateral

1983 Radiography of lower limb

57518-00 Radiography of femur

Asthma: *Procedure Codes*

1889 Other therapeutic interventions on respiratory system

92043-00 Respiratory medication administered by nebuliser (mist therapy)

1974 Other radiography of chest

58500-00 Radiography of chest

570 Non-Invasive ventilatory support

92209-00 Management of non-invasive ventilatory support, <=24 hours

92209-01 Management of non-invasive ventilatory support, >24 and <96 hours

92209-02 Management of non-invasive ventilatory support, >=96 hours

Head Trauma (TBI): *Procedure Codes*

3 Insertion of Intracranial CSF devices

39015-00 Insertion of external ventricular drain

39015-02 Insertion of intracranial pressure monitoring device, with monitoring

8 Intracranial drainage

39600-00 Drainage of intracranial haemorrhage

9 Intracranial Decompression

40015-00 Subtemporal Decompression

40106-00 Hind-Brain decompression

40106-01 Posterior Cranial Fossa decompression

14 removal of Intracranial Haematoma or abscess

39603-01 Removal of intracranial haematoma via osteoplastic craniectomy

39603-01 Removal of intracranial haematoma with craniectomy

25 Procedures for skull fracture

39606-00 Elevation of closed skull fracture

39606-01 Reduction of closed skull fracture

39609-00 Debridement of compound skull fracture

39609-01 Elevation of compound skull fracture

39612-00 Elevation of compound skull fracture with repair of dura and brain

39609-02 Reduction of compound skull fracture

39612-01 Reduction of compound skull fracture with repair of dura and brain

1952 Computerised Tomography of Brain

56001-00 CT of Brain

56007-00 CT of Brain with IV contrast medium

1957 Computerised Tomography of Brain, Chest and Abdomen

57001-00 CT of Brain and Chest

57007-00 CT of Brain and Chest with IV contrast medium

57001-01 CT of Brain, Chest and Abdomen

57007-01 CT of Brain, Chest and Abdomen with IV contrast medium

***Sepsis**: Procedure Codes*

738 Venous Catheterisation

13815-00 Central vein catheterisation

30 Lumbar Puncture

39000-00 Lumbar Puncture

***General**: Procedure Codes*

1890 Therapeutic Interventions on cardiovascular system

92052-00 Cardiopulmonary resuscitation

13400-00 Cardioversion (includes defibrillation)

92053-00 Closed cardiac chest massage

568 Airway Management

22007-00 Endotracheal Intubation, single lumen

22007-01 Management of Endotracheal Intubation, single lumen

569 Ventilatory Support

13882-00 Management of continuous ventilatory support, <=24 hours

13882-01 Management of continuous ventilatory support, >24 and <96 hours

13882-02 Management of continuous ventilatory support, >=96 hours

12.0 References

[1.] College of Emergency Medicine (UK). Emergency Medicine Minimum Dataset v 0.72009.

[2.] Department of Health (UK). A&E Clinical Quality Indicators Data Definitions. Department of Health; 2010 [cited December 2010]; PDF Document]. Available from: http://www.dh.gov.uk/en/Publicationsandstatistics/Publications/index.htm.

[3.] Australian Council on Healthcare Standards. Australasian Clinical Indicator Report: 2001 – 2009. Determining the Potential to Improve Quality of Care: [serial on the Internet]. 2010.

[4.] Australasian Council of Healthcare Standards, Australasian College for Emergency Medicine. Draft Emergency Medicine Indicators - Clinical Indicators Users Manual V5.02011; 5.0.

[5.] NHS - Institute for Innovation and Improvement. The Good Indicators Guide - understanding how to use and choose Indicators.

[6.] Louis Graff, Carl Stevens, Daniel Spaite, Foody J. Measuring and Improving Quality in Emergency Medicine. Acad Emerg Med. 2002;9(11):1091 - 107.

[7.] Welch S, Augustine J, Camargo CA, Reese C. Emergency Department Performance Measures and Benchmarking Summit. Acad Emerg Med. 2006;13(10):1074-80.

[8.] Schull MJ, Hatcher, C.M., Guttman, A., Leaver, C.< Vermeulen, M,> Rowe, B.H., Anderson, G.M., Zwarenstein, M., . Development fo a Consensus on Evidence-Based Quality of Care Indicators for Canadian Emergency Departments: ICES Investigative Report2010.

[9.] Richardson P. Quality, Performance and Performance Indicators: Developing a Model for Quality, Peerformance and Performance Indicators2010.

[10.] Ministry of Health. Ethnicity Data Protocols for the Health and Disability Sector2004: Available from: www.moh.govt.nz.

[11.] Australasian College of Emergency Medicine. ACEM Policy on Standard Terminiology2009: Available from: http://www.acem.org.au/media/policies_and_guidelines/P02_-_Standard_Terminology_16.04.09.pdf.

[12.] Australian Resource Centre for Healthcare Innovations. Emergency Models of Care: about Short Stay Units2010: Available from: http://www.archi.net.au/resources/moc/emergency-moc/aboutssu.

[13.] NSW Department of Health. Clinical Sevices Re-Design Program - Models of Emergency care2006: Available from: www.health.nsw.gov.au.

[14.] Statistics New Zealand. Ethnicity - Definition (In Statistics NZ Home > Surveys and Methods > Methods > Classifications and standards > Classifications and related statistical standards > Ethnicity). [cited 2011 1st February]; Available from: http://www.stats.govt.nz/surveys_and_methods/methods/classifications-and-standards/classification-related-stats-standards/ethnicity/definition.aspx.

[15.] Cormack D. Making Ethnicity Data Count. Best Practice Journal (NZ). 2007;October(9):44-6.

[16.] Robson B, Harris R, (Editors). Hauora: Māori Standards of Health IV. A study of the years 2000-2005. 2007.

[17.] Harris R, Purdie G, Robson B, et al. Estimating Maori Hospitalisations and Cancer Registrations. In: Te Ropu Rangahau Hauora a Eru Pomare Hauora IV [serial on the Internet]. 2007.

[18.] Ministry of Health. NHI Frequently Asked Questions. Wellington [cited 2011 1st February]; Definition of National Health Identifier Number]. Available from: http://www.nzhis.govt.nz/moh.nsf/pagesns/265?Open#01a.

[19.] Ministry of Health. Ethnicity Data Protocols Key Message: 2004 Ethnicity Data Protocols - Supplementary Notes2009: Available from: www.moh.govt.nz.

[20.] Clare Salmond PC, June Atkinson,. NZDep2006 Index of Deprivation 2007: Available from: http://www.wnmeds.ac.nz/NZDep-info.html.

[21.] White P GJ, Salmond C, Atkinson J, Crampton P,. Atlas of Socioeconomic Deprivation in New Zealand NZDep2006.2008: Available from: http://www.moh.govt.nz.

[22.] Australasian College of Emergency Medicine. G24 - Guidelines on the implementation of the Australasian triage scale in emergency departments 2005: Available from: www.acem.org.au. .
[23.] Ministry of Health. SHORTER STAYS IN EMERGENCY DEPARTMENTS HEALTH TARGET GUIDANCE DOCUMENT FOR DHBS2009: Available from: http://www.moh.govt.nz/moh.nsf/indexmh/ed-tools-toolsbytopic.
[24.] Australasian College of Emergency Medicine. S-18: Statement on Responsibility of Care in Emergency Departments2005: Available from: http://www.acem.org.au/media/policies_and_guidelines/responsibility_care.pdf.
[25.] Guttmann A, Schull MJ, Vermeulen MJ, Stukel TA. Association between waiting times and short term mortality and hospital admission after departure from emergency department: population based cohort study from Ontario, Canada. Bmj. 2011 June 1, 2011;342.
[26.] Thygesen K, Alpert JS, White HD, on behalf of the Joint ESC/ACCF/AHA/WHF Task Force for the Redefinition of Myocardial Infarction, TASK FORCE MEMBERS: Chairpersons: Kristian Thygesen JSA, Harvey D. White ,, Biomarker Group: Allan S. Jaffe C, Fred S. Apple , Marcello Galvani , Hugo A. Katus , L. Kristin Newby , Jan Ravkilde ,, et al. Universal Definition of Myocardial Infarction. Circulation. 2007 November 27, 2007;116(22):2634-53.
[27.] WRITING COMMITTEE MEMBERS, Krumholz HM, Anderson JL, Bachelder BL, Fesmire FM, Fihn SD, et al. ACC/AHA 2008 Performance Measures for Adults With ST-Elevation and Non-ST-Elevation Myocardial Infarction: A Report of the American College of Cardiology/American Heart Association Task Force on Performance Measures (Writing Committee to Develop Performance Measures for ST-Elevation and Non-ST-Elevation Myocardial Infarction): Developed in Collaboration With the American Academy of Family Physicians and the American College of Emergency Physicians: Endorsed by the American Association of Cardiovascular and Pulmonary Rehabilitation, Society for Cardiovascular Angiography and Interventions, and Society of Hospital Medicine. Circulation. 2008 December 9, 2008;118(24):2596-648.
[28.] WRITING COMMITTEE MEMBERS, Masoudi FA, Bonow RO, Brindis RG, Cannon CP, DeBuhr J, et al. ACC/AHA 2008 Statement on Performance Measurement and Reperfusion Therapy: A Report of the ACC/AHA Task Force on Performance Measures (Work Group to Address the Challenges of Performance Measurement and Reperfusion Therapy). Circulation. 2008 December 9, 2008;118(24):2649-61.
[29.] Luepker RV. Delay in acute myocardial infarction: Why don't they come to the hospital more quickly and what can we do to reduce delay? American Heart Journal. 2005;150(3):368-70.
[30.] Kushner FGM, FACC, FAHA, FSCAI, Co-Chair; Hand, Mary MSPH, RN, FAHA, Co-Chair *; Smith, Sidney C. Jr MD, FACC, FAHA, Chair; King, Spencer B. III MD, MACC, FSCAI, Co-Chair; Anderson, Jeffrey L. MD, FACC, FAHA; Antman, Elliott M. MD, FACC, FAHA; Bailey, Steven R. MD, FACC, FSCAI; Bates, Eric R. MD, FACC, FAHA; Blankenship, James C. MD, FACC, FSCAI; Casey, Donald E. Jr MD, MPH, MBA; Green, Lee A. MD, MPH; Hochman, Judith S. MD, FACC, FAHA; Jacobs, Alice K. MD, FACC, FAHA, FSCAI; Krumholz, Harlan M. MD, SM, FACC, FAHA; Morrison, Douglass A. MD, PhD, FACC, FSCAI; Ornato, Joseph P. MD, FACC, FAHA; Pearle, David L. MD, FACC, FAHA; Peterson, Eric D. MD, MPH, FACC, FAHA; Sloan, Michael A. MD, MS, FACC, FAHA; Whitlow, Patrick L. MD, FACC, FAHA; Williams, David O. MD, FACC, FAHA, FSCAI. 2009 Focused Updates: ACC/AHA Guidelines for the Management of Patients With ST-Elevation Myocardial Infarction (Updating the 2004 Guideline and 2007 Focused Update) and ACC/AHA/SCAI Guidelines on Percutaneous Coronary Intervention (Updating the 2005 Guideline and 2007 Focused Update): A Report of the American College of Cardiology Foundation/American Heart Association Task Force on Practice Guidelines. . Circulation. 2009;120(22):2271-306.
[31.] Department of Health (UK). National Service Framework for Coronary Heart Disease: Modern Standards & Service Models. Executive Summary2000: Available from: http://www.dh.gov.uk/prod_consum_dh/groups/dh_digitalassets/@dh/@en/documents/digitalasset/dh_4057525.pdf.

[32.] National Heart Foundation of Australia, Cardiac Society of Australia and New Zealand. National Heart Foundation of Australia and Cardiac Society of Australia and New Zealand: Guidelines for the management of acute coronary syndromes 2006. Medical Journal Australia. 2006;184(8):Supplement.
[33.] Lindsay P, Schull M, Bronskill S, Anderson G. The Development of Indicators to Measure the Quality of Clinical Care in Emergency Departments Following a Modified-Delphi Approach. Academic Emergency Medicine. 2002;9(11):1131-9.
[34.] Myocardial Ischaemia National Audit Project [MINAP]. How the NHS cares for patients with heart attack2010: Available from: http://www.rcplondon.ac.uk/clinical-standards/organisation/partnership/Pages/MINAP-.aspx.
[35.] Schull MJ, Vermeulen M, Slaughter G, Morrison L, Daly P. Emergency department crowding and thrombolysis delays in acute myocardial infarction. Ann Emerg Med. 2004;44(6):577-85.
[36.] British Orthopaedic Association BGS. THE CARE OF PATIENTS WITH FRAGILITY FRACTURE2007.
[37.] Scottish Intercollegiate Guidelines Network. Management of hip fracture in older people: A National Clinical Guideline2009.
[38.] New Zealand Guidelines Group (NZGG). ACUTE MANAGEMENT AND IMMEDIATE REHABILITATION AFTER HIP FRACTURE AMONGST PEOPLE AGED 65 YEARS AND OVER: Best Practive Evidence-Based Guideline2003.
[39.] Shiga T, Wajima Zi, Ohe Y. Is operative delay associated with increased mortality of hip fracture patients? Systematic review, meta-analysis, and meta-regression. Canadian Journal of Anesthesia / Journal canadien d'anesthésie. 2008;55(3):146-54.
[40.] Nicole Simunovic PJD, Sheila Sprague, Gordon H. Guyatt, Emil Schemitsch, Justin DeBeer, and Mohit Bhandari. Effect of early surgery after hip fracture on mortality and complications: systematic review and meta-analysis. Canadian Medical Association Journal. 2010;182(15):1609–16.
[41.] Simunovic N, Devereaux P, Bhandari M. Surgery for hip fractures: Does surgical delay affect outcomes?.(Review Article). Indian Journal of Orthopaedics. 2011;45(1):27.
[42.] Leung F, Lau T, Kwan K, Chow S, Kung A. Does timing of surgery matter in fragility hip fractures? Osteoporosis International. 2010;21(0):529-34.
[43.] Richardson D, McMahon KLH. Emergency department access block occupancy predicts delay to surgery in patients with fractured neck of femur. Emerg Med Australas. 2009;21(4):304-8.
[44.] Canadian Institute for Health Information (CIHI). Health Indicators 20102010.
[45.] Von Titte SN, McCabe CJ, Ottinger LW. Delayed appendectomy for appendicitis: Causes and consequences. The American Journal of Emergency Medicine. 1996;14(7):620-2.
[46.] Whyte C, Levin T, Harris BH. Early decisions in perforated appendicitis in children: lessons from a study of nonoperative management. Journal of Pediatric Surgery. 2008;43(8):1459-63.
[47.] Ingraham AM, Cohen ME, Bilimoria KY, Ko CY, Hall BL, Russell TR, et al. Effect of delay to operation on outcomes in adults with acute appendicitis. Archives of Surgery. 2010;145(9):886-92.
[48.] Omundsen M, Dennett E. Delay to appendicectomy and associated morbidity: a retrospective review. ANZ J Surg. 2006 Mar;76(3):153-5.
[49.] Ditillo MF, Dziura JD, Rabinovici R. Is it safe to delay appendectomy in adults with acute appendicitis? Annals of Surgery. 2006;244(5):656-60.
[50.] Owen E. Appendicitis: A Plea for Immediate Operation. The Lancet. 1913;441.
[51.] Levy MMM, FCCP; Fink, Mitchell P. MD, FCCP; Marshall, John C. MD; Abraham, Edward MD; Angus, Derek MD, MPH, FCCP; Cook, Deborah MD, FCCP; Cohen, Jonathan MD; Opal, Steven M. MD; Vincent, Jean-Louis MD, FCCP, PhD; Ramsay, Graham MD; For the International Sepsis Definitions Conference,. 2001 SCCM/ESICM/ACCP/ATS/SIS International Sepsis Definitions Conference. . Critical Care Medicine. 2003;31(4):1250-6.
[52.] Dellinger RPMD, Levy MMMD, Carlet JMMD, Bion JMD, Parker MMMD, Jaeschke RMD, et al. Surviving Sepsis Campaign: International guidelines for management of severe sepsis and septic shock: 2008. [Article]. Critical Care Medicine January. 2008;36(1):296-327.

[53.] Institute for Healthcare Improvement (IHI). Sepsis Definitions. 2004 [May 2011]; Available from:
http://www.survivingsepsis.org/About_the_Campaign/Documents/sepsisdefinitionsihitool.pdf.
[54.] Goldstein BMD, Giroir BMD, Randolph AMD, Members of the International Consensus Conference on Pediatric S. International pediatric sepsis consensus conference: Definitions for sepsis and organ dysfunction in pediatrics*. [Article]. Pediatric Critical Care Medicine January. 2005;6(1):2-8.
[55.] The College of Emergency Medicine (UK). CLINICAL EFFECTIVENESS COMMITTEE: Clinical Standards for Emergency Departments2010: Available from: www.collemergencymed.ac.uk.
[56.] Not Just a Matter of Time: A reveiw of urgent and emergency care services in England: Commission for Healthcare Audit and Inspection2008 September.
[57.] ACEM/ACEN/CENA Draft Joint Policy Emergency Department Pain Management DP58 [College Policy]: Australasian College for Emergency Medicine; 2008 [updated 24/08/09]; Available from:
https://www.acem.org.au/media/member_docs/DP58_NICS_joint_FACEM_CENA_ACEN_ED_Pain_Policy.pdf.
[58.] G:24 Guidelines on the Implementation of the Australasian Triage Scale in Emergency Departments. [database on the Internet]. Australasian College of Emergency Medicine. 2000 [cited 25/10/10]. Available from:
http://www.acem.org.au/media/policies_and_guidelines/G24_Implementation__ATS.pdf.
[59.] P58 - Joint Policy Statement Emergency Department Pain Management [database on the Internet]. Australasian College of Emergency Medicine. 2009 [cited 25/10/10]. Available from:
http://www.acem.org.au/media/policies_and_guidelines/P58_Joint_Policy_on_Pain_Management.pdf.
[60.] Macintyre PE, Schug SA, Scott DA, Visser EJ, SM W. Acute Pain Management: Scientific Evidence (3rd edition). APM:SE Working Group of the Australian and New Zealand College of Anaesthetists and Faculty of Pain Medicine
http://wwwnhmrcgovau/guidelines/health_guidelineshtm [serial on the Internet]. 2010.
[61.] Grant PS. Analgesia Delivery in the ED. American Journal of Emergency Medicine. 2006(24):806-9.
[62.] Motov SM, Khan AN. Problems and Barriers of Pain Management in the Emergency Department: Are we ever going to get better? Journal of Pain Research. 2009(2):5-11.
[63.] Arendts G, Fry M. Factors associated with delay to opiate analgesia in emergency departments. J Pain. 2006 Sep;7(9):682-6.
[64.] Statistics New Zealand. QuickStats about Culture and Identity - Ethnic Groups in New Zealand2006: Available from:
http://www.stats.govt.nz/Census/2006CensusHomePage/QuickStats/quickstats-about-a-subject/culture-and-identity/ethnic-groups-in-new-zealand.aspx.
[65.] Rumball-Smith JML. Not in my hospital? Ethnic disparities in quality of hospital care in New Zealand: a narrative review of the evidence. N Z Med J. 2009;122(1297):68-83.
[66.] Bramley D, Hebert P, Jackson R, Chassin M. Indigenous disparities in disease-specific mortality, a cross-country comparison: New Zealand, Australia, Canada, and the United States. N Z Med J. 2004 Dec 17;117(1207):U1215.
[67.] Pletcher MJ, Kertesz SG, Kohn MA, Gonzales R. Trends in opioid prescribing by race/ethnicity for patients seeking care in US emergency departments. Jama. 2008 Jan 2;299(1):70-8.
[68.] Anderson KO, Green CR, Payne R. Racial and ethnic disparities in pain: causes and consequences of unequal care. J Pain. 2009 Dec;10(12):1187-204.
[69.] Green CR, Anderson KO, Baker TA, Campbell LC, Decker S, Fillingim RB, et al. The unequal burden of pain: confronting racial and ethnic disparities in pain. Pain Med. 2003 Sep;4(3):277-94.
[70.] Todd KH. Influence of ethnicity on emergency department pain management. Emergency Medicine. 2001;13(3):274-8.

[71.] Fuentes EF, Kohn MA, Neighbor ML. Lack of association between patient ethnicity or race and fracture analgesia. Acad Emerg Med. 2002;9(9):910-5.
[72.] Mitchell R, Kelly AM, Kerr D. Does emergency department workload adversely influence timely analgesia? Emerg Med Australas. 2009;21(1):52-8.
[73.] Hwang U, Richardson L, Livote E, Harris B, Spencer N, Sean Morrison R. Emergency department crowding and decreased quality of pain care. Acad Emerg Med. 2008 Dec;15(12):1248-55.
[74.] Forero R, Mohsin M, McCarthy S, Young L, Ieraci S, Hillman K, et al. Prevalence of morphine use and time to initial analgesia in an Australian emergency department. Emerg Med Australas. 2008 Apr;20(2):136-43.
[75.] Hwang U, Richardson LD, Sonuyi TO, Morrison RS. The effect of emergency department crowding on the management of pain in older adults with hip fracture. J Am Geriatr Soc. 2006 Feb;54(2):270-5.
[76.] Terrell KM, Hustey FM, Hwang U, Gerson LW, Wenger NS, Miller DK, et al. Quality indicators for geriatric emergency care. Acad Emerg Med. 2009 May;16(5):441-9.
[77.] Kelly A-M. Setting the benchmark for research in the management of acute pain in emergency departments. Emergency Medicine. 2001;13(1):57-60.
[78.] Jao K, McD Taylor D, Taylor SE, Khan M, Chae J. Simple clinical targets associated with a high level of patient satisfaction with their pain management. Emerg Med Australas. 2011;23(2):195-201.
[79.] Rodrigo GJ, Rodrigo C, Hall JB. Acute Asthma in Adults*. Chest. 2004 March 1, 2004;125(3):1081-102.
[80.] British Thoracic Society, Scottish Intercollegiate Guidelines Network. British Guideline on the Management of Asthma2009: Available from: www.brit-thoracic.org.uk.
[81.] New Zealand Guidelines Group (NZGG). Best Practice Evidence-based Guideline: The Disagnosis and Treatement of Adult Asthma2002: Available from: http://www.nzgg.org.nz/guidelines/0003/Full_text_Guideline.pdf.
[82.] Hodder RM, MSc, M. Diane Lougheed, MD, MSc, Brian H. Rowe, MD, MSc, J. Mark FitzGerald, MD, Alan G. Kaplan, MD, and R. Andrew McIvor, MD, MSc,. Management of acute asthma in adults in the emergency department: nonventilatory management. Canadian Medical Association Journal. 2010;182(2):E55:E67.
[83.] Raewyn Gavin and Colleagues. Starhsip Childrens Health Clinical Guideline: Management of Acute Asthma2009.
[84.] Paediatric Society of New Zealand. Best practice Evidence-Based Guideline: Management of Asthma in Children aged 1-15 years2005: Available from: http://www.nzgg.org.nz/guidelines/0104/Asthma_endorsed.pdf.
[85.] Rowe BH SC, Ducharme FM, Bretzlaff JA, Bota GW. Early emergency department treatment of acute asthma with systemic corticosteroids (Cochrane Review). Cochrane Database of Systematic Reviews [serial on the Internet]. 2008; (4).
[86.] Day P, Weir, R,. Effectiveness of Therapeutic agents in the treatment of asthma: a critical appraisal of the literature:2001.
[87.] Lougheed MD, Garvey N, Chapman KR, Cicutto L, Dales R, Day AG, et al. Variations and Gaps in Management of Acute Asthma in Ontario Emergency Departments. Chest. 2009 March 1, 2009;135(3):724-36.
[88.] What Is Health Promotion? [Web Article]: Health Promotion Agency Northern Ireland (now: Public Health Agency NI); 2009 [1st September 2010]; Available from: http://www.healthpromotionagency.org.uk/Healthpromotion/Health/section2.htm.
[89.] National Institute for Health and Clinical Excellence. Head Injury: Quick Reference Guide2007: Available from: www.nice.org.uk.
[90.] Jagoda AS, Bazarian JJ, Bruns Jr JJ, Cantrill SV, Gean AD, Howard PK, et al. Clinical Policy: Neuroimaging and Decisionmaking in Adult Mild Traumatic Brain Injury in the Acute Setting. Ann Emerg Med. 2008;52(6):714-48.

[91.] Bryan-Hancock C HJ, editor. The Global Burden of Traumatic Brain Injury; Preliminary results from the Global Burden of Disease Project. Injury Prevention Safety 2010; 2010. www.injuryprevention.bmj.com: Injury Prevention 2010.
[92.] Accident Compensation Corporation. Evidence Based Best Practice Guideline Summary: Traumatic Brain Injury - Diagnosis, Acute Management and Rehabilitation2007: Available from: http://www.nzgg.org.nz/guidelines/0129/pdf_final.pdf.
[93.] Stiell IG, Wells GA, Vandemheen K, Clement C, Lesiuk H, Laupacis A, et al. The Canadian CT Head Rule for patients with minor head injury. The Lancet. 2001;357(9266):1391-6.
[94.] J Bartlett RK-W, A D Mendelow, J D Miller, et al,. Guidelines for the initial management of head injuries: Recommendations from the Society of British Neurological Surgeons. British Journal of Neurosurgery. 1998;12(4):349-52.
[95.] Needham et al. Making the Best Use of a Department of Clinical Radiology: Guidelines for Doctors2003: Available from: http://qmc.nhs.uk/radguide/index.html.
[96.] Scottish Intercollegiate Guidelines Network. Early management of patients with a head injury: A National Clinical Guideline2009: Available from: http://www.sign.ac.uk/pdf/sign110.pdf.
[97.] Lloyd DA. The Royal College of Surgeons of England: A position paper on the acute management of patients with head injury. Annals of the Royal College of Surgeons. 2005;87:323-5.
[98.] Bullock et al. Surgical Management of Traumatic Brain InjuryUndated.
[99.] Seelig JM, Becker DP, Miller JD, Greenberg RP, Ward JD, Choi SC. Traumatic Acute Subdural Hematoma. New England Journal of Medicine. 1981;304(25):1511-8.
[100.] Lind CRP, Heppner PA, Robins TM, Mee EW. Transfer of intubated patients with traumatic brain injury to Auckland City Hospital. ANZ Journal of Surgery. 2005;75(10):858-62.
[101.] R. Kejriwal I, Civil,. Time to definitive care for patients with moderate and severe traumatic brain injury—does a trauma system matter? . The New Zealand Medical Journal. 2009;122 (11 September)(1302):40-6.
[102.] Stelfox HT, Bobranska-Artiuch B, Nathens A, Straus SE. Quality Indicators for Evaluating Trauma Care: A Scoping Review. Arch Surg. 2010 March 1, 2010;145(3):286-95.
[103.] Stelfox HTMDPF, Straus SEMDMF, Nathens AMDPF, Bobranska-Artiuch BMD. Evidence for quality indicators to evaluate adult trauma care: A systematic review *. [Review]. Critical Care Medicine April. 2011;39(4):846-59.
[104.] Willis C.D. et al. Interpreting Process Indicators in trauma care: Construct validity versus confounding by indication. Int J Qual Health Care. 2008;20(5):331-8.
[105.] The Royal College of Surgeons of England and British Orthopaedic Association. Better Care for the Severely Injured2000: Available from: http://www.rcseng.ac.uk/publications/docs/severely_injured.html.
[106.] Steyerberg EW, Mushkudiani N, Perel P, Butcher I, Lu J, McHugh GS, et al. Predicting Outcome after Traumatic Brain Injury: Development and International Validation of Prognostic Scores Based on Admission Characteristics. PLoS Med. 2008;5(8):e165.
[107.] Joint Commission on Accreditation of Healthcare Organisations. Hospital Accreditation Standards2006: Available from: http://books.google.co.nz/books?id=5hihoN2VuPoC&printsec=frontcover&dq=HOSPITAL+ACCREDITATION+STANDARDS&hl=en&ei=J73iTc2LPOLQiALRoK2TBg&sa=X&oi=book_result&ct=result&resnum=1&ved=0CCoQ6AEwAA#v=onepage&q&f=false.
[108.] Kripalani S, LeFevre F, Phillips CO, Williams MV, Basaviah P, Baker DW. Deficits in Communication and Information Transfer Between Hospital-Based and Primary Care Physicians. JAMA: The Journal of the American Medical Association. 2007 February 28, 2007;297(8):831-41.
[109.] Australasian College of Emergency Medicine. Policy on Components of an Emergency Medicine Consultation2008: Available from: www.acem.org.au.
[110.] Jansen JO, Grant IC. Communication with general practitioners after accident and emergency attendance: computer generated letters are often deficient. Emergency Medicine Journal. 2003 May 1, 2003;20(3):256-7.

[111.]   Taylor DM, Cameron PA. Discharge instructions for emergency department patients: what should we provide? Journal of Accident & Emergency Medicine. 2000 March 1, 2000;17(2):86-90.
[112.]   Wass AR, Illingworth R. N.,. What information do general practitioners want about accident and emergency patients? Jounral of Accident and Emergency Medicine. 1996;13:406-8.
